# 121st Annual Meeting Medical Library Association, Inc. May 10–27, 2021

**DOI:** 10.5195/jmla.2022.1438

**Published:** 2022-01-01

**Authors:** JJ Pionke, Ellen M. Aaronson

**Affiliations:** 1 pionke@illinois.edu, Proceedings Coeditor, Applied Health Sciences Librarian, University Library, University of Illinois at Urbana-Champaign, Urbana, IL; 2 aaronson.ellen@mayo.edu, Proceedings Coeditor, Librarian, Mayo Clinic Libraries, Rochester, MN

## INTRODUCTION

The Medical Library Association (MLA) held its 121st annual meeting virtually May 10-May 27, 2021, due to the COVID-19 pandemic. The meeting was entitled “MLA '21 vConference,” and the theme was “Transforming Our Diversifying Communities.” The 2021 conference was built on the success of the MLA '20 vConference and Exhibits with improvements based on feedback and lessons learned from last year's event. The virtual meeting was again broken into segments, all available using a variety of online platforms. The meeting had 1,203 registrations plus 290 exhibitor representatives. Additional meeting content—including the meeting program and various electronic presentations from the business meetings, plenary sessions, poster sessions, and program sessions—can be accessed by all meeting registrants via the MLA '21 website.

## OPENING SESSION (HELD VIRTUALLY DUE TO THE COVID-19 PANDEMIC)

Lisa Traditi: Good morning. I am Lisa Traditi, your MLA president and cohost for this MLA 2021 Annual Conference and Exhibits opening session. Thank you all for being with us today!

Kris Alpi: Greetings, everyone! I am Kris Alpi, your MLA president-elect, and cohost for today's session. Lisa—it is great to share this virtual stage with you for the kick-off session of the MLA 2021 Annual Conference and Exhibits. It feels like I just saw you cohost our annual conference celebration.

Lisa Traditi: It does feel that way, doesn't it? One year ago, we were hopeful that the COVID pandemic would be over quickly. We were in the midst of planning for an in-person conference in Portland that we had rescheduled from May to August. Well, we know what happened. COVID did not go away. Our lives got upended more than we could have imagined … and still no in-person meetings. So, we went back to the drawing board and created MLA's first virtual conference and exhibits in August 2020. More than 1,100 of you attended, and 9 months later, 955 of you so far are registered for this conference. Of those, more than 630 of you can attend thanks to an outstanding commitment of 65 institutions who have purchased group registrations. Yes, you can still register because you do not want to miss out on keynotes, contributed content, exhibits and vendor programing, and everything else the 2021 National Program Committee has planned. If you have financial hardship, you can apply to register for free.

Kris Alpi: Joining you from my office in Portland, Oregon, I have to say we really missed hosting MLA in 2020. Portland is a wonderful city, and everything is green and flowering beautifully in May. The good news is that we will be in Portland for MLA 2024. We are delighted that we were able to make that happen. Updating the restaurant guide will give us a great excuse to sample all the wonderful local and global cuisine.

Lisa Traditi: I had hoped to be welcoming you all to Washington, DC. We couldn't make it to our nation's capital this year as originally scheduled for MLA '21, and we are sorry to miss visiting DC as well. No doubt we will find a way back to DC for a future conference.

Kris Alpi: Next year we will meet in New Orleans in person for the MLA '22 conference. Let's keep our fingers crossed and plan to make the most of seeing each other and that fabulous city!

### Reflections

Lisa Traditi: Before we get started, we would like to take a moment and reflect on issues that touch us all: The COVID pandemic itself, with over 3 million deaths, including nearly 600,000 in the US alone. This pandemic has had a devastating effect on physical health, mental health, financial well-being, exclusion, disparities, and more, either personally, with family and friends, or through our work. As health information professionals, we are particularly upset by the sustained attacks on evidence-based medicine and research and by the undermining of our institutions, particularly those essential to the quality of health care. This is why the MLA Board of Directors just bestowed the MLA Award for Distinguished Public Service on Dr. Anthony S. Fauci, director of the National Institute of Allergy and Infectious Disease, for his leadership. Dr. Fauci was the Joseph Leiter MLA/NLM lecturer in 2006, on the topic of “Pandemic Influenza and Other Emerging Infectious Diseases: Public Health Threat and the Research Agenda.” I invite you to listen or relisten to his presentation, which, unfortunately, was so spot on in its predictions. Dr. Fauci could not be with us today, but he did send us his photo holding the award.

Kris Alpi: Just as we need to take collective action and share responsibility toward our global health, we must attend to attacks on our democracy such as the physical assaults on the Capitol and efforts to limit access to voting. Finally, and most importantly, we have individual and group responsibility to end systemic racism and fully embrace diversity, equity, and inclusion. We must create a just, civil, and sustainable society for today and for future generations.

Lisa Traditi: Thank you to all of you for being active participants in this journey. Thank you to our amazing caucuses for contributing to MLA's voice. Thank you for leaning forward and engaging in dialogue so we can all be better.

Kris Alpi: Let's get started with our exciting session. Lisa and I are your cohosts for this session. We will invite others to join us and share some videos as well. Coming up:

1. MLA Statement of Appropriate Conduct—Our objective is that this MLA experience be open, inclusive, collaborative, and enjoyable by all. We will review our rules and guidelines as the first agenda item.
2. Recognition of Sponsors—New this year, we will hear from our platinum, gold, and silver partners throughout this session with their recorded messages.
3. MLA'21 experience—Tara Douglas-Williams and Neville D. Prendergast, cochairs of the 2021 National Program Committee, will recognize those who made this meeting possible. They will also give us tips on how to best experience the next few weeks.
4. Presidential address—Lisa will share her thoughts and perspectives on her year as MLA president.
5. Awards, recognitions and in memoriam—MLA's cherished tradition of celebrating our colleagues through presenting this year's awards and recognitions, as well as honoring and remembering colleagues who passed away in this last year. You told us you enjoyed the videos, photos, and clips we assembled in 2021, so we hope that you will find this year's presentation inspiring as well.

### MLA Statement of Appropriate Conduct

Lisa Traditi: The MLA Board of Directors approved the MLA Statement of Appropriate Conduct before the August 2020 conference. It applies to all MLA activities including conferences, meetings, workshops, online forums, social media, continuing education, and all means of communication relating to MLA activities. It applies to all MLA members, nonmembers, invited guests, speakers, moderators, instructors, exhibitors, staff, and all others who participate in an MLA activity or event. Our objectives are:

- an open, inclusive, and collaborative environment within the association in all its activities and events, and outside the profession;
- diversity, equity, and inclusion in professional practice, leadership of health sciences libraries, and information professionals;
- advancement and support of accessibility for all stakeholder groups; and
- irreproachable ethical standards that call for health sciences librarians to conduct all professional relationships with courtesy and respect.

Please read the statement. It includes the dos and don'ts, how to report inappropriate behavior, and the resolution process. At the bottom of the statement, there is a link to an incident reporting form.

### Stronger together

Kris Alpi: Creating an open, inclusive, and collaborative environment starts with each of us. MLA exists because we want to connect with each other, grow our knowledge and skills, and amplify voices in our profession to achieve change. Visit “I Am MLA” to learn our stories and share your own as an “I Am MLA Ambassador.”

### Exhibitor and sponsor recognition

Lisa Traditi: Please join Kris and me in giving a heartfelt welcome and thank you to our sponsors and exhibitors who once again demonstrated their outstanding support for MLA and the value they see in health information professionals. MLA vendors partnered with us in the redesign of the exhibitor experience for MLA '21. New this year, we have dedicated time for you to meet with your exhibitors and to attend their education and product showcase sessions. They contribute to MLA in so many ways:

- In 2020, exhibitors, sponsors, and advertisers provided $781,000 or 31% of operating revenues. I don't want to imagine what membership dues, meeting registration, and other subscription costs would be without our partners' financial support.
- They provide content that is valuable to all of us.
- They engage and collaborate with librarians on initiatives that advance our profession.

### InSight Initiative

Kris Alpi: We just enjoyed one of those collaborations, the sixth summit of the MLA InSight Initiative. The InSight Initiative is a thought-leadership initiative that brings health sciences librarians and information providers together to engage in high-level, high-value dialogue on issues that matter to both our communities. Last week, 325 librarians and company representatives participated in three days of learning and small group discussions focused on moving toward equitable health sciences knowledge sharing. This timely issue is critically important to both our communities. Through this engagement we better understand the barriers that prevent us from making real progress toward equity in scholarly communication and are designing strategies for overcoming these barriers.

Lisa Traditi: Let's take a few moments to thank our annual conference sponsors who have contributed a minimum of $6,000 to more than $45,000 in support. We are grateful for their support and invite you to take time to personally thank them for their support throughout the meeting and afterward.

Kris Alpi: Our Bronze-level sponsors, in alphabetical order, are American Pharmacists Association, BMJ, Elsevier, Radiological Society of North America, Rittenhouse, and Springer Nature.

Lisa Traditi: Our Silver-level sponsors are EBSCO, JAMA, and Wiley.

Kris Alpi: Our Gold-level sponsors are Clarivate, Elsevier-Clinical Key, McGraw Hill, and *New England Journal of Medicine*.

Lisa Traditi: And I want to especially thank our Platinum-level sponsor, Wolters Kluwer, who is also sponsoring MLA's upcoming New Member First Time Attendee gathering on Wednesday afternoon.

Let's hear from Vikram Savkar on behalf of Wolters Kluwer experience.

*[Video.]*

Lisa Traditi: That was wonderful. We will hear from each of our four Gold sponsors throughout this session. Now, let's talk about this year's conference. We are energized by the enthusiasm of our members, organizers, presenters, MLA staff, and technology partners who have been so committed in preparing this meeting. We salute the hard work and vision of the organizers for their creativity, building on the success of last year's virtual conference to design a new and improved experience for all of us to enjoy. The word “awesome” comes to mind. Here to talk to you about the awesome virtual conference you are about to experience, please welcome your NPC '21 cochairs, Tara Douglas-Williams, AHIP, and Neville D. Prendergast.

Tara Douglas-Williams: Thank you Lisa. I am Tara Douglas-Williams, your NPC '21 cochair.

Neville Prendergast: And I am Neville Prendergast, your NPC '21 cochair.

Tara Douglas-Williams: On behalf of the entire National Program Committee, welcome to “MLA '21: Transforming Our Diversifying Communities.” And what a theme that is! If any of you have a clear view of how we'll be transforming over the next year, please let me know … It has certainly been interesting, and hard, work these last few months, with many unexpected twists and turns, and even more unknowns. It is a delight to be here today for this kick-off session. Thank you for your participation and for your trust in us.

Neville Prendergast: Tara, I have just received the count for this session: Over 500 are watching us live so far, with a steady flow coming online as this gathering continues!

Tara Douglas-Williams: Our NPC '21 team has been extraordinary. Please join Neville and me in recognizing them. Before we share with everyone what's in store for this vConference and Exhibits, let's hear from two of our Gold sponsors.

*[Video.]*

Neville Prendergast: Thank you to McGraw Hill and the *New England Journal of Medicine* for their support. Here is what is in store for this year's conference. Registered attendees have already been exploring the online planner. Before the end of this session, most of the on-demand content will be available for you to view. Tara and I will provide a few tips about the planner and how to best experience MLA '21. First, let's talk about some big changes for this conference. I want to thank our Accessibility and Disability Caucus for providing information to presenters on how to make this conference more accessible. I especially want to acknowledge NPC member Charlotte Beyer, AHIP, for her coordination of this effort. Our vendor team from CadmiumCD also updated their tools to enable captions for audio presentations—and editing captions—by all presenters. Our Cadmium team really listened to attendees from MLA '20 and staff. You have probably already noticed one of them, which is … time zone support! When you first login to the Online Planner, make sure to put in your time zone. All live sessions then show in your time zone so you can plan your time effectively. You also now have the opportunity for one-on-one networking. Every attendee has the option to visit and connect with each other by text chat, video chat, or both. Up to four people can gather and connect! The last big change I want to point out is the My Experience page, your one-stop-shop for all your favorites, questions, attendee network, system checks, favorite exhibitors, and more.

Tara Douglas-Williams: Neville, those are all game-changers for a virtual conference—I can see I need to set up some lunch dates!

Neville Prendergast: Tara, you're right—but now let's get into how our attendees can access all the great MLA '21 content. Listening to MLA '20 attendees again, we have designed this vConference and Exhibits into 4 phases—a little differently than last year. Phase 1 is much the same: the Live Kick-off! It includes the Opening Session, which is happening right now, followed by Awards & Recognition a bit later. Our first networking event will be on Wednesday. It is for first time attendees and new members. There will be random group networking through Zoom that will help new members meet each other and MLA leaders.

Tara Douglas-Williams: Neville, I also want to mention that we are leaving Fridays free throughout MLA '21.

Neville Prendergast: Tara, yes, we are—thanks for pointing that out. Our phase 2 is the Exploration phase. This is the two-week period today through May 20, where you can view on-demand content and interact with text Q&A. Pick the best time for you to view content and ask questions. Watch lightning talks and paper presentations—your presenters have recorded their slide presentations with voiceovers and captions synced to each slide or are adding transcripts. The poster viewing experience will be outstanding. Many presenters have recorded short audio presentations (with captions) to augment your experience. And in another enhancement, presenters will be notified when you ask your questions. When presenters answer in the system, you will get an email with the answer or can also view it online in your “My Experience” page. Tara, why don't you take us through phase 3, Dedicated Exhibitor Days?

Tara Douglas-Williams: Neville, thanks—you saved a really important phase for me! The third phase is our Dedicated Exhibitor Days on May 19 and 20. As you heard earlier, our company partners really help MLA put on this conference and provide other support throughout the year. They've also dedicated time to create educational content and (new for MLA '21) product demonstrations along with their virtual booths. Let me highlight a few items.

Attend our virtual ribbon cutting and opening of the Virtual Exhibit Hall on May 19 at 9:45 a.m., central time.

- From our exhibitors, expect twelve solution showcases (both live and recorded) and six product demonstrations on May 19 and 20.
- Exhibitors will also have video chats in their virtual booths, so you can meet with your representatives.
- In a further enhancement, you'll also be able to text-chat with exhibitors one-on-one.

Neville Prendergast: And Tara, I know that you, as an incoming board member of MLA, will definitely be spending time thanking our sponsors in the hall.

Tara Douglas-Williams: [Virtual nodding and thumbs up.]

Neville Prendergast: Now let me quickly run through phase 4. You know all those on-demand papers and lightning talks? Beginning the Friday before, staff will change those from on-demand to scheduled times and days on May 24 to 26 so you can drop in to video chat with those presenters. Those gatherings are limited to twenty-five minutes and twelve people to make sure you can actually have a conversation. As a reminder, these are NOT presentations: watch on demand ahead of time and then come and chat!

The seventeen immersion sessions are both live and effective virtual learning experiences. If you miss one, you can watch the recording of general portions later.

Do not miss the three keynotes! And be sure to save a little time after listening to Janet Doe Lecturer Sandra Franklin for a virtual reception and conversation outside of the live-cast event. And finally, please join our National Library of Medicine speakers for the NLM Update on Thursday—and join Patti Brennan in the evening for her signature trivia event!

Tara, that was a lot of information! Before we move on to tips and tricks on how to have the ultimate experience, let's hear from our remaining two Gold sponsors.

*[Video.]*

### Tips and tricks for MLA '21

Tara Douglas-Williams: Thank you to Clarivate and Elsevier-Clinical Key for their continuing support. Ready to hear some tips and tricks for this MLA '21 vConference? A good place to start is by making sure you're in the online planner. Select “Login” to connect with your MLANET username and password. And if you are already logged into MLANET, you won't need to log in again. Then, fill out your attendee profile with your picture, interests, virtual ribbons, and connection preferences. Use the star “favorite” feature to mark sessions you want to attend, such as solution showcases organized by vendors, plenary speaker sessions, and immersion sessions. Set aside time for the Virtual Exhibit Hall, Poster Gallery, and on-demand sessions.

Neville Prendergast: For exhibitors, you can favorite their live educational sessions May 19 and 20, and the Virtual Exhibit Hall will go live by our ribbon-cutting on May 19. You will start seeing more navigation items as the conference progresses. Don't miss the live plenary sessions with our featured speakers. Those include:

- Dr. Damon Tweedy on Monday, May 24, for the McGovern Lecture
- Mitzy Baum on Tuesday, May 25, for the NLM/MLA Leiter Lecture
- Sandra Franklin, AHIP, FMLA, on Wednesday, May 26, for the Janet Doe Lecture
- Dr. Patti Brennan, Diane Babski, and Dr. Olivier Bodenreider on Thursday, May 27, for the NLM update

All sessions will be live, with Q&A. Recordings will be open to a wider audience.

Neville Prendergast: We want to emphasize that starting today, you can access on-demand content. This includes all papers, lightning talks, and the virtual poster gallery. Q&A is enabled now. That means you can ask questions and participate in conversations with the presenter and other attendees by typing in questions. For papers and lightning talks, you will also be able to video chat live at set times from May 24 to 26. Check the schedule for exact times for each small group of presenters. At any time, you can connect with other attendees and presenters one-on-one directly through the website.

Tara Douglas-Williams: The seventeen immersion sessions will be presented live. You can also watch them later after they are recorded. Our presenters and facilitators have redesigned their sessions for the virtual experience. We opted to use Zoom for this, for a familiar look and feel, and many will use breakouts for group discussion.

Neville Prendergast: And if I could add one final tip, although the whole conference will be fun, our caucuses have created some one-hour midday topic sessions and networking gatherings, plus you can play MLA '21 Quest for prizes!

Tara Douglas-Williams: Neville, that's a lot! I can't wait to get started. Thanks to the speakers and contributed content presenters for being so flexible and for going the extra mile to make MLA '21 a success. Your participation is essential to strengthening our community. Let's welcome again our emcees, Kris and Lisa.

## MLA 2020/2021 OUTGOING PRESIDENTIAL ADDRESS (PLENARY SESSION)

### Monday, May 10, 2021

*The MLA 2020/2021 Outgoing Presidential Address was held prior to the vConference and virtually due to the COVID-19 pandemic*.

Kris Alpi: Thank you, Tara and Neville. As I think about the work Tara and Neville presented and the progress that we in MLA have made together this year, it's well summed up by a quote from Aisha Bowe, cofounder and CEO, STEMboard: “Don't spend life daydreaming about 'what could be.' Invest your energy in what is right in front of you and see how it can be cultivated into something meaningful.” We actually will ask you all to dream along with the group thinking about MLA's future as part of our 125th Anniversary planning. Now let's focus on the second part of the quote, which reflects how we as volunteers cultivate a meaningful present for our association. Lisa Traditi has invested her energy in being an inspiring and caring leader in her tenure as MLA president in this most challenging year. Please join me in congratulating Lisa Traditi, MLA's 2020–21 president. Lisa, thank you so much for your leadership and kindness.

Lisa Traditi: Thank you, Kris. I joked with friends that I wanted the entire text of this talk to simply be, “Phew!” Twenty twenty was definitely a year, wasn't it? Sometimes it felt like the year that would *never* end. Oh no, what the … ?! And finally, we welcomed 2021. Ah, yes, I remember the sweet, naive, summer youth I was when I thought that 2021 would be better than 2020. But no, wave after wave of continuing political upheaval, increasing COVID surges, and heartbreaking social injustices kept on coming. They hit and hit and hit. Sometimes, I felt like Bernie looked at the inauguration—I'm here, I'm ready for any weather, let's just get on with this.

And now we're here. Last May, my most fervent hope was that the association would not lose everything—our financial security or any of you! A year later and, for the most part, we, as individuals and as an association, are intact. I'm happy to report that the state of our association is good. MLA is in good financial shape. Our membership numbers are good. You'll hear more about that at the business meeting next Tuesday, May 18. We are indeed all MLA—I'm proud of all of you, who worked within the association and pushed us to embrace the challenges presented to really do hard things and advance the association. And yes, there were certainly times when we started to crack, to show our flaws. So, we tried to break things and rebuild in different ways—accelerating change where we could and slowing things down to thoughtfully recreate when needed. We had to take a good hard look at the association and how we do things. For example, as we worked to embrace diversity, equity, and inclusion, we made mistakes *and* we acknowledged them and made corrections. We had to change other ways of doing things, including our annual meeting, among so much more. All of our chapters had successful virtual meetings last year, too. I was sad to miss the opportunity to visit the chapters in person. One unexpected benefit was that I was able to present the MLA Update at every one of the fall and spring chapter meetings! I never would have been able to manage that in the before-times. Thinking about how things sometimes fall apart can give us the opportunity to put things back together in better ways—it reminds me of the ancient Japanese art of kintsugi or kintsukuroi. Thanks to Wikipedia, I learned that kintsugi 
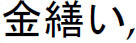
 “golden joinery”), also known as kintsukuroi 
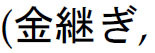
 “golden repair”), is the Japanese art of repairing broken pottery by mending the broken areas with lacquer dusted or mixed with powdered gold, silver, or platinum. As a philosophy, it treats breakage and repair as part of the history of an object, rather than something to disguise. I think that's what many of us in MLA have been doing, even if we didn't realize it. We have been finding what's not working and putting the broken parts together, adding in similar pieces to make something better and, hopefully, more beautiful than before.

These transformations are everywhere: the DEI committee is hitting its stride. Their recent Living Library event was wonderful, and I hope you'll make sure to attend the next one, scheduled for June 16. Our publications groups are working hard, too:

- *JMLA's* Diversity & Equity Committee started their work in 2020—like all of us, they are learning and making changes.
- The Oral History Committee is increasing the number of interviews with our colleagues of color to ensure that we preserve their key contributions to the profession and the association. At the same time, we have added members from all the identity caucuses and challenged the committee to rethink their organizational structure.
- *MLAConnect* is recruiting new editors—this is another great opportunity to make our diverse voices heard.
- The association's Government Relations & Scholarly Communications work is now streamlined with MLA headquarters staff coordinating and inviting expert volunteers from communities. This year, the Governmental Relations Committee and Joint MLA/AAHSL Legislation Committee planned the May 10–21 Capitol Hill Meeting Event. Since we aren't in DC in person, the meetings are being held by videoconference or phone. The planning group offered four Zoom orientation sessions and created a website with helpful resources. More than fifty members from twenty-six states have signed up to participate. With this year's vConference, free registrations are available to anyone who doesn't get funding from your employer, or is unemployed or furloughed, or you are unable to pay for the conference yourself. Please apply—last year some of the free registrations went unused!
- I want to give a shout-out to Charlotte Beyer and the folks on the NPC who worked hard to make changes in how abstracts are evaluated—with attention to more inclusive language and respect for authors' preferences.
- We now have closed captioning for our presentations!

And just think about all the ways our caucuses have continued to fill our cup by engaging with members. They have focused on creating content, highlighting and embracing diversity and equity, and transforming our communities. Our caucuses are speaking up, not just on health sciences librarianship, but on important social justice and equity issues in our society. All the MLA caucus statements are freely available on MLANET. We're also focusing on task forces for specific goals. For example, in planning for MLA's 125th Anniversary, the board approved a new strategic goal that uses the touchstone of our 125th year to focus on how we can build a better, more inclusive future for the association. I appointed three task forces who will work on the objectives of this important goal. I look forward to seeing the results of their work.

But wait! There's more! Please make sure to mark your calendars for Kris Alpi's Presidential Inaugural Address at the end of the MLA Business Meeting on Tuesday, May 18 at 1:00 p.m.-2:30 p.m., central time. Of course, researching about kintsugi led me happily down many rabbit holes, one of which was the artist and author Austin Kleon's blog. Kleon is the author of the book *Steal Like an Artist* and others. I recommend you spend some time there. Here's a quote from Kleon's blog about kintsugi and its relationship to wabi-sabi. *[Slide.]* The idea that there is beauty in unfinished and imperfect things resonates with me—since I feel unfinished and imperfect all the time. Our work and our association are unfinished and imperfect. We'll keep breaking things. And the things we think are perfect will be impermanent or incomplete. All of that is okay, because we're still learning.

Last year, I challenged us all to train for agility—to focus on our ability to quickly respond or even predict changes in our environment before they happen—perhaps even to learn to direct those changes. MLA members, caucuses, committees, editorial boards, and all our other groups have strengthened our agility and resilience this year—even when we did not believe we had the will to go on. Let's keep practicing and developing the skills and capacity within the association and our profession to anticipate change. And also remember to keep our heads up while doing all of this, in order to analyze the environment and anticipate movements when obstacles appear.

So, phew! We made it this far—not unmarked or unchanged—but hopefully stronger. Definitely more financially stable, and hopefully more agile and resilient. Thank you all for working to build and embrace a better version of MLA. An MLA that represents all of us. In a year where so many things needed to be rebuilt and repaired, I am forever grateful for the relationships that only grew stronger over this past year. Thank you:

- to the MLA headquarters staff who kept me on track. If at any point I looked organized, it was only due to their regular and gracious reminders and help.
- To my boss and direct reports, for keeping things going while I was distracted.
- To my parents, family, and friends, who love me no matter what.
- And, especially, to my husband Frank—seen here looking at the view from the foundation of our future vacation home in southern Colorado.

And to you, my friends. Many of you know that I am retiring at the end of June. I'm so lucky to have spent more than three decades in a profession and association that has given me so much joy and fulfillment. And to end my career as president of this association—well, I can hardly find words. I'll be around as past-president though, so you're not done with me yet! My heartfelt thanks to all of you for the great honor of your trust in selecting me to be president of our Medical Library Association. It has been the honor of my lifetime.

Kris Alpi: Thank you, Lisa. I look forward to continuing to learn from you as I step into the role of MLA president later this week.

## IN MEMORIAM

Kris Alpi: Before we get started with awards and fellowships, MLA has a tradition to pause and recognize our cherished members who passed away in the last year. Their counsel and friendship will be deeply missed. We have produced this video you will see today to honor their memory. The MLA Fellows and MLA members have generously provided in-depth profiles and photographs for this year's In Memoriam. We will share the In Memoriam on MLANET. We encourage you to view it and share it with others to learn more and be inspired.

*[Video.]*

Lisa Traditi: Truly inspiring and beautiful. Thank you. Let's get started with our recognitions and awards. New this year, we asked our award recipients to record their names using “say my name.” To up our game even more, and to pronounce those names correctly, please look forward to the “radio” voice of MLA's very own Darell Schmick! Darell—thank you! We are so grateful.

## RECOGNITIONS AND AWARDS

Kris Alpi: Donations to MLA by individuals, large and small, are a demonstration of your commitment to support MLA grants, scholarships, fellowships, awards, and prizes. Those contributions help build MLA's endowment, which, augmented by donations from foundations, institutions, sponsors, and MLA operations, provided over $90,000 of support to 130 individuals in 2020. Thank you to the 57 individuals who gave over $20,000 in calendar year 2020. We recognize them in the next 2 slides.

Lisa Traditi: We are fortunate and thankful to have talented members serve in various MLA editorial and coordinator positions. I would like to recognize them today.

- *MLAConnect* Editor: Christine Willis, AHIP
- *JMLA* Editor: Katherine Goold Akers
- MEDLIB-L Coordinator: Richard James
- Oral History Project Director: Carolyn Lipscomb, AHIP, FMLA

Thank you for all your efforts on behalf of the association. I will share more about Carolyn's contributions later in the program. MLA advances our mission through its programs and services. Those initiatives are successful thanks to the committed work of MLA volunteers in caucuses, committees, juries, task forces, and as allied representatives. We recognize our volunteers for your expertise and time to advance your association and the profession.

Kris Alpi: This membership year, 235 new members have joined MLA. New members bring fresh ideas and energy and are essential to MLA's future and diversity. We will be welcoming new-to-MLA members and first-time conference attendees at our annual new member/attendee networking event on Wednesday, May 12. This year's program features virtual speed networking and a keynote address by MLA Past-President Beverly Murphy.

Lisa Traditi: The Credentialing Committee evaluates and grants membership to the Academy of Health Information Professionals, known as AHIP. There are 1,008 AHIP members. This is an impressive 40% of MLA members who have earned the AHIP credential. Here are the names of new members in the Distinguished and Senior categories. *[Slide.]* Here are the names of the new members in the Member and Provisional categories. *[Slide.]* There were no new Emeritus members this year. Congratulations to all of you. Many MLA chapters provide funding for their members to receive AHIP. Your support is essential. Thank you.

Kris Alpi: From coast to coast, including Alaska, Hawaii, and several Canadian provinces, MLA's twelve chapters serve as an important home for members. They offer regional meetings, grants and scholarships, and other programs at the local and state level. The MLA Board of Directors recently approved the merger of the New York-New Jersey and Philadelphia Regional Chapters into the new Liberty Chapter. For nearly three years, the Chapter Merger Steering Committee combed through each chapter's structures to determine synergies and opportunities between both chapters to ensure a smooth transition for everyone into one cohesive group.

Lisa Traditi: Now I have the pleasure to present MLA's Chapter Project of the Year Award, which recognizes excellence, innovation, and contributions to the profession of health sciences librarianship. This year's recipient is the Northern California and Nevada Medical Library Group for its goals of promoting diversity in health sciences librarianship in a meaningful and sustainable way. Let's hear a few words from the chapter Past-President, Rachel Keiko Stark, AHIP, NCNMLG Chapter.

Kris Alpi: Enabling our members to pursue their professional development regardless of their financial ability is an important MLA inclusion and equity objective. This year, we have so far provided fifty no-charge MLA'21 vConference registrations to members. The $17,500 funding for this initiative comes from the proceeds of the Ysabel Bertolucci MLA Annual Meeting Grant and EBSCO/MLA Annual Meeting Grants endowments, augmented by MLA's operating budget.

Lisa Traditi: MLA's professional development program is the hallmark of our association. MLA grants offer members opportunities to pursue professional development courses that promote excellence in the field of health sciences librarianship. MLA offers several funding opportunities to assist qualified students in graduate library science programs. This enables members to further their careers and brand themselves as experts in their organizations. Here's Darell to announce the award recipients.

Darell Schmick: The Naomi C. Broering Latinx Heritage Grant provides a librarian with an interest in Latinx community information services the opportunity to pursue a professional activity in cutting-edge medical information services using the latest technical formats. This year's recipient is Hanni Nabahe. The grant will allow Hanni to promote a community-academic partnership that minimizes information silos in services critical to essential workers within the Spanish-speaking community and who have been particularly affected by the COVID-19 pandemic.

The Continuing Education Grant supports continuing education to develop applicants' knowledge of the theoretical, administrative, or technical aspects of librarianship. This year there are two recipients. Alanna Campbell will participate in the “Service Design: Toward a Holistic Assessment of Library Services” course, where she will learn about the various tools libraries and librarians can use to implement a service design approach to assessment. Alessia Zanin-Yost, AHIP, plans to advance her professional skills and gain new ideas on how to support students and faculty by participating in a series of continuing education courses.

The MLA Scholarships are awarded to students enrolled in or entering an ALA-accredited library school. This year's recipients are:

- Claudio Garcia, who shared that his interests in scholarly communication and open access have deepened as he's had the opportunity both in his job and coursework to explore the ways that the research ecosystem impacts real-world health care outcomes. Claudio wants to become a health sciences librarian so he can help people live healthier lives by connecting clinicians and consumers to actionable, quality health information.
- Laurier Lynette Cress has a professional objective to make the resources and materials within an institution as equitable as possible. This includes impartial and accurate metadata curation, designing and implementing an acquisitions policy built upon diversity and inclusion, and promoting the institution's collections to as many people as possible.

Kris Alpi: Thank you Darell. Now on to our international awards. MLA seeks to establish global partnerships and support the information needs of underserved individuals throughout the world. Today we are pleased to recognize members and colleagues who strive to educate and network with practitioners around the world. Their work makes a difference by advocating for access to information and helping to set standards for health information management.

Darell Schmick: The purpose of the Cunningham Memorial International Fellowship is to assist in the education and training of health science librarians from countries outside the United States and Canada. This year's recipient, Mercy Wamunyima Monde, looks forward to traveling next year. She plans to use the fellowship to enhance her leadership capabilities in health science librarianship in Zambia while sharpening her systematic review skills, evidence-based medicine skills, consumer health information skills, and knowledge translation skills. MLA members will have an opportunity to meet Mercy in person next year when we all gather in New Orleans.

Let's continue our world travels. The MLA Librarians without Borders/Elsevier Foundation/Research4Life Grants are an expansion of the Elsevier Foundation/Librarians without Borders/E-library Training Initiative. This program supports Hinari/Research for Life training activities that promote the use of the scientific resources in emerging low-income countries. Five institutions received 2019–2020 grants. The first four are Sir Albert Cook Library, Makerere University, Kampala, Uganda; Nelson Mandela African Institution of Science and Technology Library, Arusha, Tanzania; Egerton University, Kenya; and Craper Benin, Centre de Recherche Agricole et Promotion des Expertises Rurales, Montreal, QC, Canada. The fifth recipient is a joint team from Medical Library, Children's National Medical Center, Washington, DC, and Bahir Dar University, Bahir Dar, Ethiopia. Congratulations to all the Librarians Without Borders grant recipients.

The T. Mark Hodges International Service Award honors an outstanding individual achievement in promoting, enabling, and/or delivering improvements in the quality of health information internationally. The 2021 award is presented to Bethany S. McGowan, AHIP.

Lisa Traditi: I always appreciate hearing from our colleagues around the world. Next, our achievement awards. Time and time again, MLA members demonstrate what it means to be an outstanding practicing health sciences librarian or library educator. Today, we honor those that exhibit significant excellence in the areas of teaching, mentoring, and volunteering at the local, regional, and national levels. Here is Darell with the Achievement Award recipients.

Darell Schmick: The Virginia L. and William K. Beatty Volunteer Service Award recognizes a medical librarian who has demonstrated outstanding and sustained service to the Medical Library Association and the health sciences library profession. The 2021 recipient is Nancy Schaefer, AHIP. Her volunteer spirit and drive to help others and uplift the profession are apparent in all her MLA work, particularly within the Public Health/Health Administration Caucus. Nancy is the behind-the-scenes leader across MLA, contributing her expertise, her passion, and her knowledge to further MLA's goals.

The Estelle Brodman Award for the Academic Medical Librarian of the Year is given to a member who has made outstanding contributions to academic medical librarianship. I am pleased to share this video from Karen Gutzman, the 2021 recipient.

The Lois Ann Colaianni Award for Excellence and Achievement in Hospital Librarianship is given to a member who has made significant contributions to the profession through overall distinction in hospital librarianship. Let's hear from this year's recipient Nancy A. Clark.

The Consumer Health Librarian of the Year Award recognizes a consumer health librarian who exemplifies the best in consumer health librarianship. We are pleased to present the 2021 award to Shari Clifton. A tenured professor with more than twenty years of expert experience in health sciences, Clifton has provided significant consumer health information training in a variety of focus areas and to diverse consumer populations. This training has extended to public librarians and fellow colleagues, as well as students. Clifton has also developed a training program for public librarians and others seeking to obtain the MLA Consumer Health Information Specialization.

The Louise Darling Medal for Distinguished Achievement in Collection Development in the Health Sciences recognizes accomplishment in collection development for the health sciences. This year's winner is the Collection Development Caucus of MLA. The Collection Development Best Practices project is a noteworthy endeavor in both its aim and design that assists fellow librarians in acquiring or enhancing knowledge about the responsibilities and tasks involved in health sciences collection development. The Carla J. Funk Governmental Relations Award recognizes a medical librarian who has demonstrated outstanding leadership in the area of governmental relations at the federal, state, or local level and who has furthered the goal of providing quality information for improved health. Barbara A. Epstein, AHIP, FMLA, is the 2021 recipient.

The Lucretia W. McClure Excellence in Education Award honors outstanding practicing librarians or library educators in the field of health sciences librarianship and informatics. Let's hear from the 2021 recipient Jodi L. Philbrick, AHIP.

Kris Alpi: It's inspiring to hear the impact that MLA members are making in the field of health information sciences. The Rising Star Program began as a biennial initiative in 2010–2011 sponsored by the board of directors. It has now grown into an annual leadership development program that matches each Rising Star with a mentor in a comprehensive curriculum that includes monthly classes, training to develop self-awareness of skills, and a group project that relates to current MLA initiatives. Let's hear about this year's outgoing Rising Star class and the incoming one.

Darell Schmick: The MLA Rising Star program gives these members the opportunity to develop skills and knowledge needed to become a leader in MLA. The 2020–2021 cohort of Rising Stars [Alyssa Kathryn Migdalski, AHIP, Kathleen Elizabeth Phillips, JJ Pionke, and Erin M. Smith, AHIP] has been quite busy this past year. You are all invited to join them for their presentation on June 23, 2021, where they will share their research “Advocacy is all of us: recommendations to enhance MLA's advocacy initiatives.” I am pleased to introduce you to the ninth Rising Stars cohort. We look forward to seeing and hearing more from our new stars. You will have an opportunity to meet them in person and learn about their experience next year when they present their work at MLA '22 in New Orleans.

Lisa Traditi: Thank you to the many members that volunteer their time and efforts cultivating, mentoring, and directing the Rising Star program. Each year MLA recognizes both published and unpublished works that highlight health sciences librarianship and the many innovations taking place in the field. These works often have a significant impact on librarians in different settings such as academic, hospital, and special libraries. And the winners are …

Darell Schmick: The Ida and George Eliot Prize is awarded for a published work that has been judged most effective in furthering medical librarianship. The winning article is “Evaluating nursing faculty's approach to information literacy instruction: a multi-institutional study.” Let's hear more from team member Bethany McGowan.

The Erich Meyerhoff Prize was established to recognize and stimulate health sciences librarians' interest in the history of medicine. The 2021 prize is awarded to Aidy Weeks, AHIP. Her manuscript offers a fresh perspective on an important figure in the history of the MLA through an investigation of racial bias in several of Mary Louise Marshall's published works. The methods for identifying the sources selected for review are sound and transparent. The arguments set forth are astute and persuasive. The subject matter is relevant and timely, given the current state of American society, the current state of the library profession, and MLA's efforts to emphasize diversity, equity, and inclusion over the past several years.

The Rittenhouse Award recognizes the best unpublished paper on medical librarianship. Robert Browder is this year's recipient for his paper on the potential of blockchain technology to change the way health information is shared among health care providers and organizations. This has the potential to be very impactful in the field of medical informatics, as well as patient health, and invites discourse on the implications of blockchain technology on transparent communication, privacy, and security.

Kris Alpi: Congratulations to all of you. One of MLA's professional competency areas is the ability for health sciences librarians to understand scientific research methods and to critically examine and filter research literature from many related disciplines. MLA supports members in their research endeavors and recognizes those that advance evidence-based practice. Today, we acknowledge members who have made significant contributions and challenge us all to use the best available evidence. Here are the recipients of MLA research awards.

Darell Schmick: The Donald A.B. Lindberg Research Fellowship funds research linking the information services provided by librarians to improved health care and is awarded to a qualified health professional, researcher, educator, administrator, or librarian. The 2021 Lindberg Fellowship is awarded to Brandon Patterson and Kerri Shaffer. Their research “Implicit bias in health care: exploring the upstream and downstream effects with virtual reality” will explore the cultural roots of bias and how that bias impacts treatment provided by health care providers. Specifically, the project aims to better understand implicit bias in hopes of addressing and affecting health care providers behaviors. The project uses virtual reality to observe and track data points in simulated health care providers-patient interactions. Branson Patterson is a key staff member of the sponsoring institution's library and its virtual reality lab.

The Research Advancement in Health Sciences Librarianship Award recognizes organizations whose exemplary actions have served to advance health information research and evidence-based practice in health sciences libraries. We are so pleased to have two recipients this year [the Harvey Cushing/John Hay Whitney Medical Library and the University of Texas MD Anderson Cancer Center Research Medical Library].

The MLA Research, Development, and Demonstration Project Grant supports projects that will promote excellence in the field of health sciences librarianship and information sciences. This team proposes to promote excellence in health sciences librarianship and more effective interdisciplinary collaboration between librarians and nurses in both clinical and academic settings with a qualitative research study. The overall goal is to raise awareness for nursing faculty, nursing students, and practicing nurses regarding the need to evaluate resources based on a variety of criteria.

Lisa Traditi: It is now my honor to share with you the recipients of some of MLA's most prestigious awards. Fellows of the Medical Library Association are chosen for their outstanding contributions to health sciences librarianship and to the advancement of MLA's purposes. This year the Board of Directors has named three association members as MLA Fellows.

Linda Walton, AHIP, FMLA, is the associate university librarian at the University of Iowa. Her outstanding and groundbreaking career in health sciences librarianship has spanned over forty years. Her contributions to health sciences librarianship, both nationally and internationally, are numerous and significant. Linda's extensive record of service to MLA includes serving on MLA's Board of Directors as treasurer (2007–2009) and MLA president (2014–2015). Known as a down-to-earth, compassionate, and supportive person, she has mentored dozens of librarians over the years, encouraging them to step outside their comfort zone to do interesting and meaningful work. Linda is much admired by her MLA colleagues.

Kristine M. Alpi, AHIP, FMLA, has had a wide-ranging and positive influence on the Medical Library Association. Her impact in health sciences librarianship is broadly felt through her research and scholarship. One of Kris's most distinct attributes is her ability to turn questions that come up in normal library operations into excellent research questions that end up contributing to health sciences libraries. She is known as a compassionate leader who always strives to keep her staff and patrons at the forefront of her vision.

Janna C. Lawrence, AHIP, FMLA, has made significant, sustained contributions to MLA through her leadership and the investment of her talents to advance the association. Her great enthusiasm is evident, as she welcomes new members to MLA or through the mentoring of new/young members, which she has done quietly throughout her career. In her role as 2020 NPC cochair, Janna displayed great patience and tact while managing member expectations during the shift to the 2020 all-virtual conference.

Kris, Janna, and Linda now have FMLA as an added credential right next to their AHIP. Congratulations to you all and thank you for all you do for MLA and the profession.

Lisa Traditi: Each year, the MLA president has the honor of recognizing librarians who have enhanced the profession of health sciences librarianship and furthered the objectives of the association. Recipients of the President's Award will all receive a unique MLA lapel pin. When we are together in person or on video in the future, I hope they will wear their pins proudly.

Now, it is my privilege to present the MLA President's Award for 2021. Since 2009, Carolyn E. Lipscomb, AHIP, FMLA, served as program director of MLA's Oral History Project. Under her leadership, the project saw an organized workflow that resulted in smooth and consistent interviewing, editing, and publishing. Carolyn oversaw twenty-seven interviews, published forty-three oral histories, and introduced the advent of several digital publications that are available on MLANET. On behalf of the board and MLA members, we thank Carolyn for her work to preserve the interviews that help illuminate the history of health sciences librarianship and the association.

The authors of MLA's COVID-19 Resources pages and those of the Spanish-language COVID-19 Resources responded in record time to create an invaluable resource for librarians, our communities, and health care consumers. Thank you to all of you for such important work. Your work highlights the value of hospital and clinical librarians to the association and the communities they serve. Thank you for your work to provide access to reliable COVID-19 information in Spanish.

Diversity and inclusion became a strategic goal of MLA in 2017. President Barbara Epstein established the Diversity and Inclusion Task Force in August of 2017. This team presented their final report to MLA in 2020, with a call to create a permanent Diversity, Equity, and Inclusion Committee. Please take a moment to read the names of all your colleagues who were part of this initial task force. Task force chair, Sandra Franklin, now joins us to accept the award.

Kris Alpi: This next distinction is very special. The Marcia C. Noyes Award is the highest honor that MLA confers on any individual. The individual also gets the traditional silver bowl from MLA and the beautiful flowers from the Foundation of MedChi, the Maryland State Medical Society, where Marcia C. Noyes worked and lived for fifty years. Here to introduce this year's recipient, as is our tradition, let's hear from the 2020 Marcia C. Noyes Award recipient, Gerald J. Perry, AHIP, FMLA.

Lisa Traditi: And now, live from Durham, North Carolina, please welcome Beverly Murphy, AHIP, FMLA, this year's Marcia C. Noyes Award recipient.

Lisa Traditi: Hi, Beverly. So great you could join us today!

Lisa Traditi: Congratulations, Beverly. You are an inspiration to us all. What a wonderful way to finish the awards session.

Kris Alpi: The awards presentations remind us of the outstanding accomplishments our peers make to the profession of health sciences librarianship. It also encourages us to continue to grow toward new levels of achievement. Congratulations, everyone!

Lisa Traditi: Thank you friends and colleagues for joining us today to kick-off the 2021 vConference. We look forward to spending time with you over the next three weeks for a variety of fun events and engaging sessions. We'll see you soon! The Opening Session is now closed!

*[End of opening session.]*

## MLA '21 ANNUAL BUSINESS MEETING

### Tuesday, May 18, 2021

*The MLA Annual Business Meeting was held prior to the vConference and virtually due to the COVID-19 pandemic*.

Maria Lopez: Hello, everyone, and welcome today to MLA 2021. Thank you for joining us. We are using Zoom webinar today. So, this means that your mics have been muted, and your cameras are automatically turned off. We will not be using the Q&A function today and really not monitoring chat, beyond maybe if you have a small technical issue. Additionally, today, at a specific time, you may be asked to raise your hand, but that will be covered in the slides as we move forward. I would like to go ahead and then pass this off to your MLA president, Lisa Traditi.

Lisa Traditi: Thank you Maria, and good afternoon, everyone. I am Lisa Traditi, your MLA president, for the next hour or so anyway. We are now in our second week of the MLA '21 annual conference, and I hope many of you are participating and enjoying the contributed content with your colleagues. I'm also looking forward to the vendor presentations starting tomorrow, visiting the virtual exhibit, and chatting with our vendors. I also can't wait for next week's live program.

I am pleased to officially welcome you to the 121st Annual Business Meeting of our Medical Library Association, over which I will be presiding. Last year, we broke association records, with more than 500 members attending our first virtual business meeting. Many of you attended for the first time because being virtual is so much more inclusive. It's wonderful to know that so many of you are participating with us today.

We have a simple agenda with lots of information we hope you find compelling. The MLA Annual Business Meeting includes the presentation of your current MLA Board of Directors, reports by the MLA treasurer and executive director, election results, and the presentation of your new MLA board. Then, we will hear from incoming MLA president Kristine Alpi, who will present her inaugural address.

Before we get started, here are a few guidelines. All MLA gatherings and interactions need to respect MLA's Code of Appropriate conduct. Please consult it. If you need to report a violation, there's a link to do so on the web. We're using Zoom webinar today, with which, by now, you are all likely extremely familiar. We will be using Zoom polls for voting, as well as the “Raise Hand” function for additional business. We'll walk you through procedures in a few minutes. We will *not* be using the Q&A feature today. Parliamentarian Chris Shaffer will share the formal business meeting process for raising an issue soon. During the year, we offer topical open forums on many areas of MLA and invite conversations and questions and offer a better experience for dialogue. So, save any Q&A you have for that. But we'll also tell you how to get in touch. Feel free to use the chat, but please note we won't be monitoring the chat for questions. And you should also feel free to tweet about the meeting, using the hashtag #mlanet21.

To get started with the annual business meeting, I would like to recognize Chris Shaffer, MLA's parliamentarian. Chris will assist us with the business portion of the meeting.

Chris Shaffer: Hello, fellow MLA members. It's nice to see you again at our second electronic business meeting, and we're getting to be pros at this. Before I get started in my official role as MLA parliamentarian, let's practice together some of the Zoom webinar features, to make sure you're familiar with them before the real deal. And, of course, this practice is for everybody that hasn't been using Zoom for the whole of last year. First, let's practice hand raising. During the meeting, there will be times when you will have the opportunity to raise your hand to speak to an issue on the floor, in the context of the official business of this meeting. Here's the fine print. Number one, a motion is introduced. Then, the presiding officer restates the motion and asks if there's discussion. Members wishing to address the motion can raise their hands. Staff will let the presiding officer know that you wish to address the motion. The presiding officer will give you permission to speak for up to two minutes. Please announce yourself and identify your home institution before you ask your questions or before you raise an objection. This process is repeated until all those who have raised their hands have had two minutes to speak to the motion on the floor. Anyone wishing to speak a second time may raise their hands and have one additional minute to speak and respond to the previous speakers. The presiding officer will then repeat the motion and call for the vote. As to the specifics of how to use the “Raise Hand” feature and speaking in a Zoom webinar, we will be monitoring who has raised their hand and share this information with the president, who will then call on you by inviting you to speak. You will see a button appear on your screen prompting you to turn your microphone on. Once you do, please announce your name and institution, and speak. Once you have spoken, please lower your hand, so that we know that you don't want to speak again. Please locate the raise hand button on your Zoom control bar. For many of you, this will be at the bottom of your screen. If you pressed it, please also unpress it so that hands are lowered when we get started.

Now, let's go through the specifics of voting. When the presiding officer calls for a vote on a motion, you will see a poll appear on a screen. Vote yes, no, or abstain. And don't forget to press that important submit button. You will have two minutes to vote. After the vote, the results will appear on the screen and will also be announced by the sergeant-at-arms. We're going to practice. A poll will appear on your screen. And it just appeared in front of my script, so I will vote. I can't even vote because I'm a panelist. Go ahead and vote. Please keep this to a short thirty seconds for this practice session. Since I don't have a stopwatch in hand, I'm going to hope that thirty seconds have passed, and we get the results of the vote. Can MLA staff display the results of the vote? There they are. Well done. Almost all of you voted no, that you are not wearing pajamas now. I think that number probably would have been higher if we'd asked if you were wearing sweatpants.

So now you know how to raise your hand and you know how to vote. So let me share with you that we may not actually have to do any of that today. In fact, we kind of hope that we don't. Robert's Rules of Order allow business to be conducted by unanimous consent, which will remove the need for discussion and a full vote.

Any member present may object to unanimous consent and require the president to open the floor for formal discussion and put the question to members for formal vote. If you do that today, we will plan to use unanimous consent, and we will ask people to raise their hand to allow members to register an objection. If that happens, then we'll go through that entire process that we just walked through. And with that, I will turn it back over to Lisa.

Lisa Traditi: Thank you, Chris. Now I'm pleased to introduce Linne Girouard, AHIP, MLA sergeant-at-arms, who will assist us with the counting of the quorum.

Linne Girouard: Thank you, Madam President. And the count is 350 participants. We have a quorum.

Lisa Traditi: Excellent. There being more than 200 voting members present, we have a quorum. So, I now officially call our meeting to order.

Chris will now walk us through our next order of business: following MLA special rules of order that will support how we introduce, discuss, and vote on new business.

Chris Shaffer: So, as you may remember, at the May 2020 MLA Annual Meeting, members adopted rules for electronic meetings going forward. Fortunately, those rules still stand, so we don't have to do all of that again. We sent you a link to those rules on May 10, which you can also consult on MLANET. We are applying the same procedure this year, such as the ones that applied to the interactions or actions that you see listed on the slide. These rules are in effect in perpetuity, unless we decide to revise, suspend, or eliminate them by a vote of at least two-thirds of those present at the annual meeting.

Lisa Traditi: Thank you, Chris. We will now proceed with a vote on a motion on whether to object, using the special rules of order or electronic meetings, as adopted in 2020. If you object, please raise your hand and I will recognize you. If you don't object, you don't need to do anything. So only if you object, raise your hand. You have twenty seconds to do so.

Chris Shaffer: Actually, Lisa was more prepared than I was.

Lisa Traditi: Okay. Are there any objections? Oh, I see one person has their hand up. Jennifer McKinnell, please unmute yourself and speak.

Jennifer McKinnell: Oh, I don't have any questions. It was an error. But nice to see you all.

Lisa Traditi: We know though that the process works if someone raises their hand. So, we've tested that. Alright. So, thank you, Chris and Linne and Jennifer for testing the system. I would now like to welcome Gurpreet Rana, MLA secretary. Hi, Preet.

Gurpreet Kaur Rana: Hi. How are you?

Lisa Traditi: I'm well. Nice to see you.

Gurpreet Kaur Rana: Thank you. Great to be joining you for what is, very unfortunately, my last official act as MLA secretary. It seems so final.

It's been a really enjoyable, challenging, and exceptional three years. As MLA secretary, I get to review board minutes. I also get to present the agenda for the 2021 business meeting. Please take a look at the agenda on your screen. We've done the first three items already. By direction of the Board of Directors, in my final act on MLA Board of Directors, I—very emotionally—move that the agenda for the 2021 Business Meeting of the Medical Library Association be adopted.

Lisa Traditi: I propose to approve the motion by unanimous consent. Any member may object, in which case we will call for discussion and then vote. Please raise your hand if you object. You have twenty seconds to do so. Okay. So, not seeing any objections, we will not proceed to a vote. The motion is passed, and the agenda is adopted. Thank you very much, Gurpreet. Thank you.

Gurpreet Kaur Rana: Thank you.

Lisa Traditi: Thank you, all. Chris, Linne, and Gurpreet. Please stick around; we might need to call on you later. Now, please welcome Kevin Baliozian, MLA executive director. Hi, Kevin.

Kevin Baliozian: Hi, Lisa. Great to be here. And I have the honor to introduce the 2020–2021 MLA Board of Directors. We have been so fortunate to have such a group of strong leaders.

This Board of Directors has expertly steered us through what has been a tumultuous year. So, thank you so much. President Lisa K. Traditi, AHIP; Kristine Alpi, AHIP, FMLA; Julia Esparza, AHIP; Shannon Jones, AHIP; Donna Berryman, AHIP; Sally Gore; Heather Holmes, AHIP; Adela Justice, AHIP; Brenda Linares, AHIP; Dale Prince, AHIP; Gurpreet Rana; Meredith Solomon; and myself. And here are the pictures. And you know you have to match the pictures if you don't know yet. They are in the same order, so that should be easy.

Lisa Traditi: Thank you, Kevin, and congratulations to all the 2020–2021 Board of Directors. Now please welcome Shannon Jones, your MLA treasurer, for the next few minutes anyway. Hi, Shannon, and welcome to the business meeting. What financial news do you want to share?

Shannon Jones: Absolutely. Good afternoon, everybody. This is my final act as the treasurer of MLA. I'm excited and sad, at the same time. But here it goes. We've had a pretty interesting year, and I look forward to sharing with you how we did. And so, as the treasurer, I share the financial stewardship of our association with Kevin, our executive director. We rely on the insights and the review of the Finance Committee to ensure that the Board of Directors can exercise its duty of care. The Finance Committee was busy this year. And a little stressed, as you might expect. Extremely stressed. We managed to still have a pretty good year, so, in addition to reviewing the budgets and the financials prepared by the MLA staff, we also work with MLA's independent auditor to ensure that we are in compliance and that we're using best practices. The Finance Committee is also responsible for setting MLA's investment strategy with our independent financial advisor. We also examine key pricing models, and another thing that we do is we analyze our contract terms with our management company MCI USA.

The most important thing we did last year was ensure the financial sustainability of MLA during COVID. Even though COVID is not over, and we are not out of the woods yet, we do have some good information to share with you. You can go to the next slide. So, here is the Finance Committee. Serving in the world as treasurer, I could not do this alone. I want to thank this group of people that you see on the screen. Dale Prince, whom I will be handing the checkbook over to at the end of this meeting. Julia Esparza, our immediate past-president. Donald Berryman, who's a member of the board. Theresa Knott, who is our member-at-large. Kevin Baliozian, our executive director and a financial wizard. Also, Miss Kelly Weaver, who is the director of finance for MLA. This whole group deserves a round of applause for their dedication and their commitment this year. We are making sure that we still have an MLA and that we are going to have an MLA for the years to come. So, you can go to the next slide.

So, yes, we survived 2020. And I know probably in March of 2020 we didn't know where we were going to be. But I'm happy to report that we are actually stronger today than we were a year ago. Both as an association, but more importantly, financially. We are strong. We have outperformed our operating forecast, though we didn't meet our pre-COVID budget, but that's not a surprise to anyone. Our net assets went up, and thanks to the stock market for that. That means that our reserves and our endowment that supports the grants are both strong. We also achieved this because we were fast to adopt new models and because of all the transformational work we all did starting five years ago. So, we were prepared when COVID came in March of 2020. And so, our position, our cash position, is stronger. We are $500,000 higher today than it was last year. And that was for a lot of reasons. We benefited from CARES Act funding. We got CARES Act funding for $383,000. Our 2020 membership is above what we budgeted, and so this is a very good sign that you all find value in MLA, and you continue to maintain your memberships. We say thank you for that. If you are new to MLA, we say welcome, and we also thank you for joining us too. The conference, this conference, is a success. And the fact that it is in May, rather than in August, really helped out our cash flow as well. And then we continue to invest. We haven't cut any programs. We've increased our offerings, on the contrary, and our offerings include a lot of free content, free programming for our members and for the public. And we continue to invest in building our education and our technology.

In preparing for this meeting, we looked at what we shared with you last May. This side last year, it was really glomming. We had $1.5 million in revenue, and they were in jeopardy. We faced up to a $900,000 possible operating loss. And all of the gains—our investment gains had been wiped out. And so, here's how we did, how we ended: our operating losses were limited to $267,000, which was the realistic range between what we thought was between a $250,000 to $500,000 loss. So, we were good with that because it wasn't a $1.5 million loss. It was more reasonable. We also, our investment gains, we gained $279,000. At this time last year it was about a $280,000 loss. That's a $560,000 positive swing in the last seven months of 2020. So, as you can see, our net assets are above $3.8 million, which is very strong. About 60% of that is our MLA endowment. And so, we appreciate our donors. We thank them for their continued investment in MLA and for helping us, allowing us to continue to grow their endowment.

Now we're going to transition into 2021 and take a look at our 2021 budget. This is the budget that the Board of Directors approved in November of 2020. See on the left column what our 2020 numbers were. And so, to 2021 budget we had a reduced operating deficit of $85,000. Anything under the $250,000 loss would be—we were okay with that. MLA also budgeted and non-operating margin of zero. So far, our investments look good, but, as we know from 2020, things can change pretty quickly. But we do believe that we will be prepared, if that were to happen.

One of the things I'm really happy about is our socially responsible investing. Probably about two years ago, a couple of our members had emailed the board and have been pushing us to move and to act on this. And so, I'm really pleased to share with you all that last week, the board amended MLA's investment policy to include a new criteria for socially responsible investment, or SRI. On the finance committee work with our investment firm to identify some best practices: best practice approach that is evidence based, using an aggregate of indices established by multiple independent reviewers that assess the quality and ranking of every company listed on the stock market. That criteria, their criteria, are grouped into four categories: community, employees, environment, and government. And so, what you will see on the right column, it provides a detail of the subcategories for each of the categories. So, our new process will allow MLA to assess our overall SRI of our investment portfolios and to make sure it is improved from year to year with minimum requirement. We also will report on those improvements at every annual meeting or via an open forum, like we did in January. So, setting SRI objectives and defining a methodology to measure success are important objectives for MLA's overall DEI strategy.

We are in a good place financially. I'm really grateful to the Finance Committee. And congratulations to Dale for taking over as a treasurer. I have every confidence that he is going to do an amazing job. And huge shout-out to Kevin Baliozian, for all of the late nights that he stayed up making sure that we were able to take advantage of CARES Act funding and making sure that we had a plan. So, I just want to give a shout-out to him, because he is a really strong piece of this puzzle. Thank you all for the opportunity to serve as treasurer. I'm going to hand it back over to Lisa.

Lisa Traditi: Thank you, Shannon. And I just want to reiterate what Shannon said: first of all, thanks to Kevin. But also, the board, which is thrilled to include this new socially responsible investment policy. And we've been so grateful, Shannon and Kevin, for your expert stewardship of the entire financial team. So, the next order of business is the Executive Director report. Welcome back, Kevin. Nice to see you.

Kevin Baliozian: Thank you so much, Lisa. Thank you, Shannon, for the kind words and this amazing lift that the entire team did. So, Shannon shared with you the good news about MLA's finances, considering the doom we were facing a year ago. This success is not a coincidence. It's the result of years of strategic leadership and a major transformation in everything we do at MLA and in how we do it. It is the success story that is yours. The MLA success story is yours and the transformation continues. So, let's take this moment to get away from the weeds and to look at our fundamentals and how intentional we all have been in driving this success. There's this acronym V-U-C-A. Many of you may have heard it. It did come from the US military, a number of years ago. And it comes every time there's a major crisis. Leading in a VUCA world. V stands for volatile: change is rapid; it's unpredictable in its nature and extent. The U stands for uncertain: the present is unclear, and the future is uncertain. C is for complex: there are many different interconnected factors that come into play with the potential to cause chaos and confusion. And then, A is for ambiguous: there's a lack of clarity or awareness about situations. Individually, these challenges are significant, and formidable and daunting when combined. That certainly has applied to all of our businesses and institutions. And MLA is no exception. So, if you look at the right side of the slide, it's the strategies to deal with these four challenging elements. You counter volatility with vision: you accept and embrace change; you develop a clear, shared vision. You meet uncertainty with understanding: you pause to listen, understand, and develop new ways of thinking and acting in response, you review, you evaluate your performance, you experiment, and then you adapt. You react to complexity with clarity: you communicate clearly; you build teams that can work effectively in a fast-paced and unpredictable environment. And then lastly, you fight ambiguity with agility: you plan ahead, you expect to alter plans, you build contingencies, and you empower individuals and small teams to make changes.

So, let's go to MLA. And what are our fundamentals? The first one is a major cultural shift toward a diverse and inclusive organizational voice representing all our many MLA communities. I am MLA, you are MLA, we are MLA. This is repeated always, often, because it's so fundamental to who we are and who we want to be. The inclusion of all our diverse communities enriches the quality and the relevance of the resulting opportunities for engagement and learning.

Experts and contributors who feel included will self-identify and will collaborate to create high quality content that is relevant to a more diverse community of practice. Increasingly, it is our caucuses and domain hubs where ideas take shape and best align with the diversity of our association's community interest.

Now, we experimented and learned a great deal about virtual meetings in the last year. We wouldn't want to repeat the uncertainty of whether we were in person or virtual last year and the back and forth. But we certainly continue to learn from the next few weeks. We learned that virtual meetings can improve and increase diversity and equity. We also look forward; as we plan forward, we plan to retain what works well online. And we plan to build on what works best in person. We won't be having an in-person meeting that is exactly how we did in-person meetings in the past. We will always have a virtual component to our meetings. And so our challenge is to come up with an experience that works best in a combination of both virtual and in person. So we look forward, starting with MLA'22 in New Orleans, to do more innovation on how you will experience the annual conference in person, or virtually, or both. Flexibility is at the center of what we do. We were quick to react on a number of things. Pricing models and when we shifted from the canceled in-person conference to the virtual conference this time last year. There was a lot going on and moving from one to the other. We design flexibility, rather than one size fits all. We introduced institutional pricing for educational offerings and the annual conference. And today about 60% or more of the conference attendees are registered through group institutional purchases that didn't exist two years ago. We introduced the CE Passport (Continuing Education Passport), which provides an individual or an institution with access to an extensive offering of online learning. Very successful last year and successful as well this year, running through December 31. We introduced financial hardship support to offer no-charge registration to those who would not otherwise have been able to afford to participate. And this year we're extending this hardship support to CE Passports as well.

So, if we go back in the past, the revenues from the *Journal of the Medical Library Association* subscriptions, continuing education, and membership were the major contributors to MLA's finances. Over the last few decades, those revenue streams all but disappeared. *JMLA* became open access without charging publication fees. And revenues for education dwindled. So as a result, MLA's financial health has been highly dependent on membership revenue and the annual conference, which includes a high level of support from vendors. Hence our stress this time last year around, frankly, both of those. We are shifting to new services, education and grants, annual conference, and membership. So a much broader and diversified offering and set of revenues from the financial perspective. That includes IMLS grants, two of them for the Research Training Institute. It also includes new lines of service such as EFTS.

Another big change is, could you remember a week last year when nothing was going on? Probably not. Last year, we had something going on just about every week. And the MLA activities used to be centered around the annual conference, right? That included sections, whose main focus often was around annual meeting programming. Now, just last year was a very different picture. The success of February's Experience MLA month was resounding, with all but a handful of caucuses participating with outstanding programming. There is a reason why membership numbers remain strong, and you are the reason. You are participating. So, thank you so much for making your organization, MLA, so strong. Back to you, Lisa.

Lisa Traditi: Thank you, Kevin. And next order of business is the 2020–2021 annual report. The MLA annual reports are a valuable read for all of us. You might discover parts of MLA you didn't know about or incredible things your colleagues have enabled. Please take time to read them, even if it's just the executive summary. Those reports show the immense diversity of our communities and programs. They're also part of MLA's archives. So, if your name is in there, you are officially recorded for posterity. This slide shows all of MLA's components that contribute to this comprehensive document. The reports are available on MLANET, as are the reports from previous years. In the interest of time, we will receive the annual reports in a block. Are there any corrections, amendments, or questions from members regarding the content or meeting of any of these reports? If so, please use the raise your hand feature, so you may be recognized. I am timing, naturally. Being no objection, these reports will be filed as presented. Thank you.

Those who are nominated each year as potential members of the MLA Board of Directors are selected by virtue of their experience and reputation to serve the association. But few can really imagine beforehand the level of commitment that election to the board really requires. And that was even more true this year. The directors who have completed their term on the MLA Board have served our association with enthusiasm, dedication, grace, good humor, and perseverance. The association and the Board of Directors express our appreciation and recognize you today for the extraordinary work and thoughtful leadership you've provided during your terms of service. Thank you for a job well done, Gurpreet Kaur Rana and Shannon D. Jones, AHIP. Pausing for thunderous applause.

I would also like to express my sincere gratitude to Julia Esparza, AHIP, MLA's 2019–2020 president. Hi, Julie. Change was the major thing for your inaugural address. You've been recognized as being “fearless and tireless” in times of change.

Throughout your presidency, you went above and beyond to see that everyone was prepared to embrace that change. And we can certainly vouch for that. You were at the helm leading, encouraging, challenging, and cheering us all to our first ever annual virtual conference, which broke attendance records. At least recent attendance records for in-person conferences. Your vision for MLA is anchored in your expert knowledge and your experience of the health information profession, your broad and deep connection with MLA members, and your curiosity and ability to always think outside the box. Your active listening, your decisiveness, and your focus in all situations, including the most challenging ones, led MLA through a complex governance transition that's resulted in a successful restructuring of our communities and the continuing evolution of our commitment to creating a culture of diversity, equity, and inclusion within the association and the profession. We can never thank you enough for your dedication to the association, but I hope the crystal gavel that we sent to you will remind you that your hard work is deeply appreciated by every member of MLA. As MLA president, you've been an inspiration, a great advisor, and a confidant. And I will miss you next year, and I regret that I can't share my gratitude in person. Thank you for everything you've done for MLA and the profession.

Julia: Thank you, Lisa. I appreciate your kind words. It was an honor and privilege to serve the members of MLA. Thank you.

Lisa Traditi: Thanks, Julie. The MLA 2021 election was conducted from January 15 to February 19, 2021. Voting statistics can be seen on your screen. Election results were announced on March 4, 2021, in *MLAConnect.* And following are the election results. Nine individuals were elected for a one-year term to the Nominating Committee. And their names appear on your screen. I'm looking forward to working with all of you on the Nominating Committee. And Shannon D. Jones, AHIP, was elected to serve as president-elect. So, Shannon, I hope you've enjoyed your thirty-second respite from being on the board. You thought you might be done, but you're not. Welcome back and congratulations! Congratulations also to Janna C. Lawrence, AHIP, FMLA, and Tara Douglas-Williams, AHIP, who were each elected by the membership to a three-year term to the MLA Board of Directors. I'm so excited to get to work with both of you. And now, it's time for my year as MLA president to come to close. It's my honor and pleasure to introduce your 2021–2022 president, Kristine M. Alpi, AHIP, FMLA. Kris is the university librarian at Oregon Health & Science University, where she leads the OHSU Library team in research and education, in clinical practice engagement, and in building collections and environments that inspire and support users. She previously directed the William R. Kenan, Jr. Library of Veterinary Medicine at North Carolina State University. Earlier in her career, she served as associate library director at Weill Cornell's Medical Library and as director of the Public Health Library of the New York City Department of Health and Mental Hygiene. Kris received her bachelor of arts and master's in library science at Indiana University, a master's in public health from Hunter College/City University of New York, and a doctorate in educational research and policy analysis from North Carolina State University. Kris considers her MLA colleagues and staff as her professional family and has been a member of MLA her entire career, starting in library school. And I am thrilled to pass the gavel to my colleague and friend, Kristine M. Alpi, AHIP, FMLA.

Kristine Alpi: Lisa, we did. It's my pleasure, both personally and professionally, to thank you on behalf of the MLA membership for your wonderful leadership during this most challenging presidential year. At the opening session last week, I enjoyed hearing your perspective on how we in MLA have grown stronger and more beautiful, together with the struggles of this year. I hope that all of our members have been able to find the time to watch that session and to appreciate your efforts. I also want to congratulate you on your upcoming retirement from your day job at the Strauss Health Sciences Library and say how grateful I am that you're not retiring from MLA and will remain with us an additional year as past-president.

I also want to say to our audience that Lisa has control of the MLAPresident@mail.mlahq.org email address for the rest of the day. So, if you all want to send thank-you messages via email, as well as all these lovely chats we're seeing, that's an address you can go for. Lisa, we were happy to send you a silver cup or token of appreciation for all you have led us in accomplishing this year. While it's currently intact without kintsugi at this time, I hope that you will still display it proudly because it symbolizes a year when we in MLA broadened our opportunities to build our future under your leadership.

Lisa Traditi: Thank you, Kris. Yes, it's still very much intact. I appreciate it. Thank you all very much. It's been an honor.

Kris Alpi: MLA members, I'm pleased to present your 2021–2022 Board of Directors. Congratulations to all of the directors on being elected. If our names are not yet familiar to you, especially those of you who may have joined MLA after the election, I do hope you'll look us up and connect with us via MLANET. And here are our photos to help put faces with our names, as you see us either at future virtual forums, committee, caucus, chapter, or domain hub meetings, or in person next year at MLA. I look forward to our working together and thank the board members for their commitment to MLA and your dedication to the profession. I now have the honor to finish the remaining items of business before we adjourn.

And next our resolutions. We have no resolutions at this time, and we'll move onto new business. That still the case that we don't have any resolutions, Kevin? Okay. Does anyone have new business to bring before the assembly? Please raise your hand if you have new business. You'll have twenty seconds to raise your hand. Okay. Not seeing any raised hands, we have no new business at this time. So, let's welcome Heather Holmes, our newly appointed secretary of the MLA Board of Directors, for our final item of business. Hi, Heather.

Heather Holmes: Kris, I'm very excited to start my new role as secretary of the Board of Directors, with a very important piece of business to wrap us up today. And I know I have big shoes to fill following in Preet's footsteps, so I'll do my best to follow her. With that being said, as we end this year's meeting, I'm sending you all out with finger hearts. Put my slide up, Kevin. And hope you all have a great MLA experience this year. I now move to adjourn.

Kris Alpi: Okay, thank you, Heather. It has been moved to adjourn, but please stick around afterward for my inaugural presidential address. Let me propose to approve the motion by unanimous consent. Any member may object, in which case we will call for discussion and then vote. Please raise your hand if you object. You have twenty seconds to do so. Alright, not seeing any objections, we will not proceed to a vote. The motion is passed, and the meeting is adjourned. I want to thank you in advance if you're going to stay with me for the next piece of our program. And feel free to stand up and stretch because it's going to be a minute or two until the transition over to my slide deck. Thank you everyone.

## PROCEDURES FOR HANDLING NEW BUSINESS

### Obtaining and assigning the floor

- A member raises their hand when no one else has the floor.
- The moderator announces the name of the member to the presiding officer.
- The presiding officer recognizes the member.
- The moderator unmutes the member.
- The member gives their name, institution, city, and state.

### How the motion is brought before the assembly

- The member makes the motion: “I move that [or “to”]” and puts the motion in the chat box [or resumes their seat and gives a copy of the written motion to the chair in face-to-face meetings].
- Another member seconds the motion in the chat box: “I second the motion” or “I second it” or “second.”
- The chair states the motion: “It is moved and seconded that … Are you ready for the question?”

### Consideration of the motion

- Members can debate the motion.
- Before speaking in debate, members obtain the floor by raising their hands in the chat box.
- The maker of the motion has first right to the floor.
- The presiding officer recognizes the maker of the motion.
- Debate must be confined to the merits of the motion.
- Debate can be closed only by order of the assembly (two-thirds vote) or by the chair if no one seeks the floor for further debate.
- If other members seek the floor for further debate, the moderator announces them to the presiding officer.
- The presiding officer recognizes them.
- The moderator unmutes the member.
- Debate is limited to two minutes.
- Repeat the process until all who seek the floor have spoken.

### The chair puts the motion to a vote

- The chair asks: “Are you ready for the question?” If no one rises to claim the floor using the raise hands feature in Zoom, the chair proceeds to take the vote.
- The chair says: “The question is on the adoption of the motion that … It is time to put the question to a vote. A poll will appear on your screen. You have two minutes to vote.”
- [Poll options: Yes | No | Abstain]
- Sergeant-at-arms: “Madame President, the vote is # in favor, # opposed, # abstain.”
- President: “There being two thirds of members present voting in the affirmative, the motion passes;” or “there being two thirds of members present voting in the negative, the motion fails.”

### Amending a motion

You want to change some of the wording that is being discussed.

- After recognition, “Madame Chairman, I move that the motion be amended by adding the following words,________.”
- After recognition, “Madame Chairman, I move that the motion be amended by striking out the following words,________.”
- After recognition, “Madame Chairman, I move that the motion be amended by striking out the following words,________, and adding in their
- place the following words,________.”

### Additional options for handling new business

#### Refer to a committee

You feel that an idea or proposal being discussed needs more study and investigation.

- After recognition, “Madame Chairman, I move that the question be referred to the xxx committee/board for further study.”

#### Postpone indefinitely

You want the membership to have more time to consider the question under discussion, and you want to postpone it to a definite time or day and have it come up for further consideration.

- After recognition, “Madame Chairman, I move to postpone the question until________.”

#### Previous question

You think discussion has gone on for too long, and you want to stop discussion and vote.

- After recognition, “Madam President, I move the previous question.”

### Limit debate

You think discussion is getting long, but you want to give a reasonable length of time for consideration of the question.

- After recognition, “Madam President, I move to limit discussion to two minutes per speaker.”

### Permission to withdraw a motion

You have made a motion and, after discussion, are sorry you made it.

- After recognition, “Madam President, I ask permission to withdraw my motion.”

## MLA 2021/2022 INCOMING PRESIDENTIAL ADDRESS (PLENARY SESSION)

### Tuesday, May 18, 2021

*The MLA 2021/2022 Incoming Presidential Address was held prior to the vConference and virtually due to the COVID-19 pandemic*.

Kris Alpi: Okay. I hope everybody's seeing this title slide. Be great to get some reassurance on that one. But thank you all for being here. And I want to start with a physical description, kind of my own alt text. So, I'm a middle-aged white woman with glasses and shoulder-length curly brown hair with white streaks. You might not be able to tell in today's light, but I'm wearing the same green blazer as in my professional headshot that was shown earlier, so you can see the real me. I had promised in the preliminary program to spotlight how I have been inspired by the unique and powerful roles that we as library and information professionals play in changing our institutions, our community, our profession, and society. But honestly, as this talk evolved, it became more just a story of us. So, I'm in my library today, and first I want to acknowledge the original inhabitants and traditional village sides of the land that Oregon Health & Science University is occupying and built upon: the Multnomah, Kathlamet, Clackamas, Tumwater, Watlala bands of the Chinook, the Tualatin Kalapuya, Molalla, Wasco and many Indigenous nations of the Willamette Valley and Columbia River Plateau. I thank the original caretakers of this land—past, present, and future. But land acknowledgments are just a beginning, and we need to invest further. I recently learned of this site Living Nations, Living Words from our first Native American United States Poet Laureate, Joy Harjo. Please visit this site, recognize First Peoples, and read, love, invest in, and share their poetry.

So, I want to start with all the thank-yous in case people can't stay till the end of this talk.

But this is also an opportunity for us to get to know each other and build community, something that Lisa already said is easier in this virtual environment than in a big meeting hall. If I identify communities in the slides or verbally and you've been part of that community, if you're comfortable doing so, please identify yourself in the chat so that others in the session can meet you and see what we all have in common. And although I can't track on that, during this session, I will look forward to reading the chat afterward.

First of all, thank you to my family and all my coworkers past and present, especially those from Oregon Health & Science University are shown here at the picture at the top. Coworkers are what makes being able to serve as positions like MLA president possible. Also, I want to thank my boss David Robinson who's on vacation today. I would love to see any coworkers from any of my previous institutions say hi and where we worked together in the chat. I want to thank Sheila Hofstetter for my first hospital library internship experience at Community Hospitals in Indianapolis. Sheila also recommended me for the National Library of Medicine (NLM) fellowship. Any NLM fellows or NLM staff here, please say hello in the chat. Next, I want to shout out to my chapter homes. The Midwest chapter, where I started, the Mid-Atlantic chapter, as a member twice, the New York-New Jersey chapter, now the Liberty chapter, and the Pacific Northwest chapter, my current home. If you're here from one of those chapters say hi. And then, finally, to all the juries and all the donors of the awards and scholarships shown here. I want to especially thank the two that are from some of my many caucus homes, from medical informatics and from public health/health administration.

I need to pause here and say that while most of this talk is focused on inclusion, care, and joy, I will be speaking about topics that might be activating or triggering to some of you, and so I wanted to give you a warning and also say that I understand if you can't stay for this.

I'm here as your MLA president due to many amazing mentors and friends. And I didn't plan to talk about structural racism today, but here we are. So, I was going to tell you about how I became a medical librarian, because I was planning to be an art librarian, and art historian Joyce Taylor, shown here on the left, was my library school advisor. She also taught the reference course. And I was a working student working in many libraries during my training. And you couldn't do her course observation in a library, where you had worked. So, her class sent me to the Ruth Lilly Medical Library of Indiana University to do an observation, and the rest is kind of history. But when I came back and wanted to switch, she helped me make it work, even though medical libraries weren't something that she had a lot of experience with. And I wanted to share her with you as part of my story. This is how she looked in 1995–1996 when I was a library student, when we had pictures of all the faculty up on the wall. And so, when I went to look for this picture, all I could find was a blurry group photo and a newsletter about some other event. And I found in *Indiana Libraries Magazine* in the archives her 2003 essay on her retirement, but it didn't have a photo. And I'll be honest; she was my advisor, but I didn't really ask her that much about her journey and her story. So, when I read this retirement essay I learned that she had written, “Since 1973 there have only been seven African Americans who have earned a PhD from Indiana University School of Library and Information Science. I was the last of the seven in 1993.” So, in 2003 there hadn't been others and her rank was senior lecturer. Now, I don't know if that was by choice or if she felt a lack of support to join the professorial ranks. My alma mater, probably like many others, doesn't make it easy to find out about Dr. Taylor or other Black librarians who earned doctorates or taught there. I had to contact the archives to find and digitize this photo. It was the only photo they had, and the newly minted metadata, assigned very recently, simply identifies her by name, the school, and the topics “women” and “minorities”—nothing about her faculty role, alumni role, or specific identity. So, I wanted to just say to all of us that as alumni we have a powerful voice. We can push our schools past the structural racism that keeps role models like Dr. Taylor hidden. And that's something that I'm going to be committed to doing so. Going to pause for a second.

Another powerful early mentor I want to share with you today is Dr. Zoe Stavri. Zoe was the National Library of Medicine Associate Fellows Coordinator when I became part of the program. In the middle here is the photo from when she was herself an NLM Fellow. She inspired me and Tammy Mays in the photo on the right to pursue our doctoral education. Zoe and I always had the best of intentions to collaborate at some point on a consumer health informatics project, something like that, but I was always too busy. In 2014, we learned that Zoe, who'd had a lifelong battle with depression, had died by suicide. And so, if you've been wondering when you're going to make time for psychological or mental health first aid training or your organization offers QPR (Question, Persuade, and Refer) training, please consider making the time. It may help you help yourself or help other colleagues.

I want to say I learned so much from my fellow North Carolina librarians who trusted me to serve as president of a group called ANCHASL—the Association of North Carolina Health & Science Libraries. Say hi if you're an ANCHASL friend. And I got to have one because Beverly Murphy's on this session. When I was elected to the MLA Board, I asked this brain trust of North Carolina MLA past-presidents and board members to dinner. When we took this photo, I had no idea that Beverly and I would eventually become MLA presidents. I am truly honored to follow in Beverly's footsteps.

Now that you know me a bit, I want to get into our theme for today. I knew I wanted to talk about feeling alone in our organizations and about MLA as a way to find communities. But fate decided to help me out with the theme. So, first, I received a Christmas gift this year of a unicorn hat that's being shown here modeled by Chex. And then, after visiting Birmingham in the UK and becoming a cricket fan, a friend's brother launched the first LGBTQ-inclusive cricket team in Birmingham, the second in just all of England. And you can see here that their mascot is the unicorns. They play their first match next week, and I'm really looking forward to hearing how that goes. But then I saw the logo for the February 2021 Mountain Pacific Health Science Libraries Conference, and who could resist a cat-a-corn, so this had to happen.

How are we like unicorns? This is how I see you, my fellow health sciences librarians; we are beautiful, we are brave, and we are valuable. And last year Lisa asked about our superpowers, and I'll say that I think mine is in asking hard questions and speaking up. Definitely on the brave side of this like-unicorn trilogy. But I think that, although I see all of our value and likeness to unicorns, I think our uniqueness makes us susceptible to extinction by those who really don't understand us.

So, I want first to say thank you to Virginia Bowden because I was funded by the Kronick Traveling Fellowship, and Virginia and David Kronick are shown here, to visit five public health libraries. But before I could finish writing up their stories to publish them, I learned that several of those libraries were going to be closed. And I was a relatively new researcher at the time, and I could not figure out how to write about them as exemplars when their excellence couldn't actually ensure their own survival. So, I put that data and those interviews and that work aside. I still have all those notes. And now I think I have the skills and perspective for how I can share them. And so, I'm going to commit to writing those stories this year in order to have them available for MLA's 125th Anniversary.

Unicorns don't have to be alone. I think the cricket club that I mentioned is a lot like MLA, bringing together teams of individuals who might feel unique or alone in other organizations. I want to specifically shout out to the solos. If you're a solo, please say hi in the chat. I've never been one fulltime, just temporarily. But honestly, I wouldn't have succeeded without colleagues in MLA, especially those in the Animal and Veterinary Information Specialist Caucus, when I became a veterinary librarian at the William Rand Kenan, Jr. Library of Veterinary Medicine. And if you know veterinary librarians, you know that we love our collective nouns, so share in the chat if you know what a group of unicorns is called, or if you've ever answered an animal health question in your career. I'll give you a second to do that before I get to the next slide, which tells you what that collective noun is.

So, a group of unicorns is a blessing. And my first MLA presentation and first caucus engagement was with the Medical Library Education Caucus, which really encouraged my love affair with research. And on the left, you can see the program from my first MLA meeting presenting as a graduate student in the new perspectives, kind of fresh voices programming sponsored by that group at the time. And I want to thank all of the Research Caucus members. My accountability buddy Dr. Nina Exner and the many collaborators and colleagues who have participated in my studies. And I want to especially thank the more than 400 of you who made time this year to reply to the COVID ILL Research team survey that we put out. We're finishing that analysis now. And to be honest, it's been very emotional reading what's happened with everyone during the pandemic, and I would not, or could not have done, this work alone. I'm also very excited to collaborate with the Communities Council, Adela Justice, and the MLA staff on helping plan and assessment of how this new community structure is working for you.

Being a unicorn apparently isn't enough these days, so here comes the cat-a-corn bringing all these great things together and making it look so easy. But we know it's not. At Wishard Health Services, where I started my career as a hospital librarian, my library director also ran media services, the patient TV system, and lots of other hospital administrative activities. I don't know when it disappeared, but there's no longer a library there. And changes and loss don't just happen to small libraries. We see the National Library of Medicine making difficult decisions about how to allocate time and resources. We answer requests for information; we give strategic feedback. Our voices matter. And yet, we have seen this year that the one great library cannot satisfy all the needs we have for it. So, sometimes we say no, and sometimes we say yes. And you'll probably have that experience with me this year as your MLA president.

Saying yes to developing the epidemiology continuing education course for MLA brought me, in a roundabout way, this position in Oregon. You can see here the beautiful silver falls, where I taught the workshop, and many of the attendees hanging out during the evening. Really lovely place to visit. I was so excited to join the Oregon Health Sciences Library Association that had previously sponsored me to be a course instructor, and I wanted to give back. But it turned out that saying no to a leadership role in that organization when I arrived in Oregon was the right decision. And because of my MLA president-elect role and attending the Chapter Council meeting, I learned about NAHSL, the North Atlantic Health Sciences Library chapter's state SIG model and was able to offer that as a potential pathway to dissolve OHSLA and find a new way for us to remain connected. What I learned from this is that when our capacity and energy is limited, sometimes it's better to use it to restructure things to a sustainable scale than to try to maintain the status quo until we burn ourselves out.

There's just two more slides. Let's rest here for a minute—appreciate those of you who've stayed for this. I inserted this image from our Portland Art Museum, because we were not able to welcome you to Portland. And then I realized the title “Please Participate” and this was actually perfect for talking with fellow members of the volunteer-led organization.

Here we are. This is my last slide. My goals this year are to help you achieve your goals and to help MLA achieve goals together, to reflect critically on our past and future, and to make those commitments I promised earlier throughout this talk. Let's create and share memories this year together.

I just have two more things that I want to kind of share very quickly. The first is that I want to congratulate my friend, mentor, and collaborator Patricia Gallagher on her retirement. And you can see a picture of us here twenty years ago in Orlando with our colleague Gail Hendler presenting on New York online access to health. And then also to say, you can see here I'm being brave; I'm riding a horse on the streets of the Upper West Side, in New York City, wearing my MLA 2001 odyssey t-shirt. And so, to wish you all happy trailblazing and to hopefully see you all in New Orleans in 2022. Thank you for being here. I really hope that you've enjoyed seeing each other in the chat. So, I'm going to stop sharing and turn it over to see if there's any last-minute things from Kevin or Maria. Thank you, everyone.

Kevin Baliozian: We're good. Kris, thank you so much. You have the last word.

Kris Alpi: Excellent! Well, thank you, everyone. Have a great week and I hope to see you at the exhibit hall ribbon cutting bright and early tomorrow morning. Thank you. Maria, I'm just staying on for a minute so I can grab the chat or can you grab the chat for me I'm not sure.

Maria Lopez: The chat is recording.

## OTHER PLENARY SESSIONS

The following plenaries were live-cast events due to the COVID-19 pandemic.

### Monday, May 24, 2021, John P. McGovern Award Lecture

*Keynote Speaker: Damon Tweedy, MD*

Dr. Damon Tweedy is the author of the *New York Times* bestseller *Black Man in a White Coat,* selected by *Time* magazine as one of the top ten nonfiction books of 2015. He has published articles about race and medicine in the *Journal of the American Medical Association* and other medical journals. His columns and op-eds have appeared in the *New York Times,* the *Washington Post,* and various other news publications.

Dr. Tweedy travels frequently to speak to physicians and clinicians, health care companies, medical schools and teaching hospitals, and other organizations involved in health and wellness about the impact of race on the medical profession at all levels.

Dr. Tweedy is a graduate of Duke University School of Medicine and Yale Law School and completed both his medical internship and psychiatry residency at Duke Hospital. He is currently an associate professor of psychiatry at Duke University School of Medicine and a staff psychiatrist at the Durham Veteran Affairs Health Care System.

### Tuesday, May 25, 2021, NLM/MLA Joseph Leiter: The Culture of Public Health and Food Safety

*Keynote Speaker: Mitzi D. Baum, MS*

Mitzi Baum joined the team at Stop Foodborne Illness (STOP) as the chief executive officer in May 2019. She is motivated by the mission to drive change through advocacy, collaboration, and innovation. During her tenure, STOP has implemented the Alliance to Stop Foodborne Illness program, which is a collaboration with food industry companies and STOP's constituent advocates to improve internal food safety culture; STOP has conducted a literature review to support research focused on the early detection of foodborne illness, and STOP convenes two multifaceted working groups to support the FDA's New Era of Smarter Food Safety Blueprint.

Prior to beginning her tenure at STOP, Mitzi cultivated a twenty-three-year career at Feeding America, beginning as a network services representative and rising to the senior-level position of managing director of food safety. Prior to Feeding America, Mitzi managed restaurants for the Peasant Restaurant Company in Atlanta, Funky's Restaurants in Cincinnati, and Lettuce Entertain You Enterprises in Chicago.

Mitzi holds a master of science in food safety and certificate in food law from Michigan State University. She earned her bachelor of science degree from Bowling Green State University. She has earned certificates in nonprofit management from the University of Chicago, food safety management from Cornell University, and quality management from DePaul University.

### Wednesday, May 26, 2021, Janet Doe Lecture: Diversity That Defines Us: The View Through A Crystal Lens

*Keynote Speaker: Sandra G. Franklin, AHIP, FMLA-Woodruff Health Sciences Center Library*

Sandra G. Franklin, AHIP, FMLA, is director of Emory University's Woodruff Health Sciences Center Library, Atlanta, GA. The library includes four clinical branches. Since joining the Emory Libraries, Franklin has served as reference librarian, head of public services, assistant director, associate director, and director since 2002.

Franklin's service to MLA began as a member and then chair of the Credentialing Committee. She served MLA as a member of the 2008 Nominating Committee, secretary/treasurer of the Leadership and Management Section, member of the Board of Directors, member of the 2015 National Program Committee, chair of the Local Assistance Committee for MLA '18 in Atlanta, and chair of MLA's Diversity and Inclusion Task Force. The task force surveyed MLA members to obtain demographic information and inserted inclusive language in association documents.

Franklin was the 2015 Southern Chapter/MLA Academic Librarian of the Year and in 2017 became an MLA Fellow. Her other professional association service includes president of the Georgia Health Sciences Library Association; treasurer, credentialing liaison, program chair, and president of the Southern Chapter/MLA; president and member of the Board of Directors and committees of the Association of Academic Health Sciences Libraries (AAHSL); and chair of the National Library of Medicine (NLM)/AAHSL Future Leadership Committee. Franklin recently became a member of the Friends of the National Library of Medicine Board of Directors.

## PROGRAM SESSIONS

Program sessions were available in an on-demand viewing format and a live format.

On-demand viewing for 100 posters, 100+ papers, and 53 lightning talks began May 10, 2021; some of these sessions are ongoing.

Sponsors: Springer Nature, Clinical Key, and the National Library of Medicine presented four on-demand sessions.

Pre-Conference Live sessions began with the Opening Session on May 10, 2021, and concluded Monday, May 17, 2021.

vConference Live sessions were presented in the following time slots: May 10, 2021, 10:30 a.m.-noon; May 12, 2021, 2:00 p.m.-3:15 p.m.; May 13, 2021, noon-3:00 p.m.; May 17, 2021, 11:00 a.m.-4:25 p.m.; May 18, 2021, 1:00 p.m.-2:30 p.m.; May 19, 2021, 9:45 a.m.-10:00 a.m.; May 24, 2021, 10:00 a.m.-5:00 p.m.; May 25, 2021, 10:00 a.m.-5:00 p.m.; May 26, 2021, 10:00 a.m.-7:30 p.m.; May 27, 2021, 10:15 a.m.-4:00 p.m. (All events were on central time.) Featured during these time slots were 18 immersion sessions, 100+ papers, and 53 lightning talks. The live immersion sessions included interactive breakout sessions, Q&A, and virtual chat with presenters.

Paper abstracts that were scheduled to be presented are available on the MLA '21 website. The final version of the abstracts reflecting only those presented at the meeting is included as a supplemental appendix to these proceedings.

## POSTER SESSIONS

The poster gallery featured 100 posters in an on-demand viewing format beginning May 10, 2021. These included audio presentations and virtual and/or chat Q&A sessions with the authors.

Poster abstracts that were scheduled to be presented are available on the MLA '21 meeting website. The final version of the abstracts reflecting only those posters presented at the meeting is included as a supplemental appendix to these proceedings. The actual posters are available online in the MLA '21 meeting website.

## OTHER MEETINGS AND EVENTS

Due to the COVID-19 pandemic, the following meetings were held virtually prior to, during, and after MLA '21: African American Medical Library Alliance Caucus Business Meeting, February 25, 2021, Business Meeting and Meet/Greet, May 25, 2021; Animal and Veterinary Information Specialist Caucus Information Meeting, May 6, 2021, and informal meeting May 24, 2021; Basic Science Caucus Business Meeting, May 17, 2021; Cancer Librarians Caucus Business Meeting, May 3, 2021; Clinical Librarians and Evidence-Based Healthcare Caucus Business Meeting, May 4, 2021; Collection Development Caucus Business Meeting, March 15, 2021; Community Council Business Meeting, May 13, 2021; Consumer and Patient Health Information Services Caucus Business Meeting, October 7, 2021; Data Caucus Business Meeting, March 22, 2021; Dental Caucus Business Meeting, May 19, 2021; Federal Libraries Caucus Business Meeting, May 3, 2021; Health Association and Corporate Librarians Caucus Business Meeting, May 14, 2021; History of the Health Sciences Caucus Business Meeting, May 13, 2021; Hospital Library Caucus Business Meeting, May 11, 2021; International Cooperation Caucus Business Meeting, May 28, 2021; Interprofessional Education and Practice Caucus Business Meeting, May 7, 2021; Latinx Caucus Business Meeting, May 25, 2021; Leadership and Management Caucus Business Meeting, May 5, 2021; LGBTQIA+ Health Sciences Librarians Caucus Business Meeting, May 17, 2021; Libraries in Health Sciences Curriculum Caucus Business Meeting, May 11, 2021; Medical Informatics Caucus Business Meeting, May 5, 2021; NNLM Regional Meetings, Monday, May 17, 2021; New Member Caucus Business Meeting, July 21, 2021; New Member/First Time Attendee Program and Networking, May 12, 2021; Nursing and Allied Health Resources Services Caucus Business Meeting, May 6, 2021; Osteopathic Libraries Caucus Business Meeting, May 26, 2021; Pediatric Librarians Caucus Business Meeting, May 12, 2021; Pharmacy and Drug Information Caucus Business Meeting, April 28, 2021; Public Health/Health Administration Caucus Business Meeting, May 20, 2021, October 6, 2021; Public Services Caucus Business Meeting, April 27, 2021, informal social, May 25, 2021; Research Caucus Business Meeting, January 27, 2021, July 21, 2021; Scholarly Communications Caucus Business Meeting, January 12, 2021, August 23, 2021; Systematic Reviews Caucus Business Meeting, April 26, 2021; Technical Services Caucus Business Meeting, May 7, 2021; Technology in Education Caucus Business Meeting, May 3, 2021; User Experience Caucus Business Meeting, May 26, 2021; Vision Science Caucus Business Meeting, April 13, 2021.

## OPEN FORUMS

There were nine open forums held virtually prior to and following the vConference: Finance, January 26, 2021; Publications, April 1, 2021; AHIP Q&A, April

14, 2021; DEI Committee, April 21, 2021; Education, July 20, 2021; Invitation to MLA'22, August 5, 2021; Research Training Institute, October 21, 2021; *JMLA,* Striving for Equality, November 2, 2021; MLA Vision 2048, November 17, 2021.

## NATIONAL LIBRARY OF MEDICINE UPDATE

The National Library of Medicine (NLM) Update and Q&A took place May 27, 2021, from 10:15 a.m.–12:15 p.m.

## LEGISLATIVE UPDATE

“Advocating for Medical Libraries with MLA: Success, Challenges, and Future Efforts” was held Thursday, May 25, 2021, 1:30 p.m.–2:45 p.m.

## OTHER SPECIAL EVENTS AND RECEPTIONS

### Wednesday, May 12, 2021, 2:00 p.m.–3:15 p.m.

- New Members/First Time Attendee Program & Networking

### Wednesday, May 19, 2021, 9:45 a.m.–10:00 a.m.

- Ribbon Cutting and Opening of the Virtual Exhibit Hall

### Wednesday, May 26, 2021, 4:45 p.m.–5:45 p.m.

- Gather, Meet, and Greet Janet Doe Lecturer Sandra G. Franklin, AHIP, FMLA

### Wednesday, May 26, 2021, 6:00 p.m.–7:30 p.m.

- Take a Break with Dr. Patricia Flatley Brennan

### Wednesday, May 26, 2021, 3:30 p.m.–4:00 p.m.

- Closing Session
- Introduction of NPC Chairs for MLA'22

## VIRTUAL EXHIBIT HALL AND EXHIBITOR SOLUTION SHOWCASES

The Virtual Exhibit Hall was home to forty-one vendors who presented videos, downloaded product and education materials, and participated in public chat and private video sessions from May 19 to May 26, 2021. Material was available for viewing for thirty days.

Exhibitors held Solution Showcases to provide information and to introduce new products and services. The following sessions were held.

### Wednesday, May 19, 2021

- Cochrane “Inside/Outside”: An Update from Carol Lefebvre, sponsored by Wiley
- The R2 Digital Library: from Rittenhouse Evidence-Based Collection Development for Health Sciences, sponsored by Rittenhouse
- Ovid—What is new and upcoming in 2021, sponsored by Walters Kluwer
- Asserting Research Expertise Using Our New InCites Article-level Microtopic Classification Scheme, sponsored by Clarivate
- NEJM Catalyst, The Source for Practical Innovations in Care Delivery, sponsored by NEJM Group
- State of GRacE—The Lancet's Group for Racial Equality, sponsored by Elsevier
- 5 Ways COVID-19 Has Changed Medical Libraries, sponsored by EBSCO

### Thursday, May 20, 2021

- NEJM Evidence—Meet the Editors, sponsored by NEJM Group
- The R2 Digital Library a Health Sciences eBook Platform, sponsored by Rittenhouse
- MedOne-Education: The Best Content Solution for Basic Sciences and Anatomy, sponsored by Thieme
- Identifying Duplicate References in EndNote 20, sponsored by Clarivate
- Pandemic at the Disco: Increasing the Pace of Translational Research, sponsored by Clarivate
- When All of Your Users Suddenly Become Remote: How COVID-19 Accelerated UCSF's Shift to Electronic Resources, sponsored by Wolters Kluwer
- Getting the Most from Your Systematic Reviews: A Co-Presentation from EndNote and DistillerSR, sponsored by Clarivate
- Improving Knowledge, Diagnosis, and Care for Patients of Color, sponsored by visualDX
- What's New in the Cochrane Library, sponsored by Wiley
- Chemical Science Resources for the Medical Library: Get to Know ACS Publications, ECRI. How ECRI Guidelines Trust (EGT) Empowers Medical Librarians to Succeed

## CONTINUING EDUCATION COURSES

There were no continuing education courses offered during the vConference.

## RESOURCES AND SERVICES

The online itinerary planner sponsored by Wolters Kluwer allowed attendees to peruse programs and events online. Live streaming was available on Twitter using the hashtag #mlanet21. The annual meeting blog posts are available on the MLA website. The MLA Professional Recruitment and Retention Committee (PRRC) is pleased to sponsor the MLA '21 Virtual Resume Clinic.

## SUPPLEMENTAL FILES

**Appendix 1:** MLA '21 Program Session Abstracts

**Appendix 2:** MLA '21 Poster Session Abstracts

