# Supplementary material for: 121st Annual Meeting Medical Library Association, Inc. May 10–27, 2021
Source: J Med Libr Assoc. 2022 Jan 1;110(1):E1–E29. doi: 10.5195/jmla.2022.1438 (PMC8830401; doi:10.5195/jmla.2022.1438)
Supplement: Supplementary file 1 — Appendix 1: MLA '21 Program Session Abstracts [file jmla-110-1-E1-s01.pdf]

Medical Library Association
MLA ’21 Immersion Session, Paper, and Lightning Talk Abstracts

MLA ’21
MAY 21–26
#MLANET21
Transforming Our Diversifying Communities

vConference & Exhibits

Abstracts for the contributed content sessions are reviewed by members of the Medical Library Association National Program Committee (NPC), and designated NPC members make the final selection of content to be presented at the annual conference.

IMMERSION SESSIONS

Content in this section is sorted by title in alphabetical order.

Accessibility during the Pandemic

Practice area: Professionalism & Leadership

JJ Pionke - Applied Health Sciences Librarian, University of Illinois at Urbana-Champaign, Champaign, IL

Katherine Greene, AHIP - Assistant Director for Resources and Copyright Support, Dahlgren Memorial Library/Georgetown University Medical Center, Washington, DC

Jess L. Callaway, AHIP - Clinical Research Librarian, Noble Learning Research Center, Atlanta, GA

Andy Hickner - Education and Outreach Librarian, Weill Cornell Medicine, Astoria, NY

Objective: This session will cover various aspects of accessibility during the pandemic and how to think about expanding and modifying digital services to be more inclusive. Topics covered will include the use of Universal Design while developing digital materials, accessibility while making said materials, for example the use of alt-text, and accessible online presentation etiquette.

Methods: We will use a variety of methods for instruction, including but not limited to lecture, discussion, mini-assessments, and short videos. We will also provide participants with a resource sheet, PowerPoints, and any other materials that we land up using.

Participant Engagement: Participants will engage in a variety of activities that are meant to spur discussion from Think-Pair-Share to group discussion.

Sponsor(s): Accessibility and Disability Caucus, UX Caucus

Health and Libraries during COVID-19: An MLA International and Intercultural Session

Practice area: Health Equity & Global Health

Ana Corral - Medical and Health Sciences Librarian, University of Houston Libraries, Houston, TX

Virginia (Ginny) Pannabecker, AHIP - Director, Research Collaboration and Engagement, University Libraries, Virginia Tech, Blacksburg, VA

Avril E. Reid - Head, Medical Sciences Library, The University of the West Indies, St Augustine Campus, Trinidad and Tobago

Jonathan Pringle - holarly Communications and Digital Librarian, University of New Mexico Health Sciences Library, Albuquerque, NM

Grace A. Ajuwon - Reference and Information Services Librarian, University of Ibadan, Ibadan, Nigeria

Ana Isabel Delgado Valentín - Legal Research Librarian, Suffolk University Law School, Boston, MA

Ellie Svoboda - Education Informationist, University of Colorado Anschutz Medical Campus

Karen Centeno-Casillas - Education Librarian, Old Dominion University, Norfolk, VA

Objective: Join this collaborative panel discussion to hear from and engage with a panel of LIS professionals and students from around the world on how they promote equity in access to health information and library services and programs, especially during a pandemic. During this session, participants will hear a range of examples related to: -- Providing services in the tumultuous time of COVID-19 from speakers from different countries -- How programs have been modified and adapted to remote work -- How presenters have successfully utilized technology and/or network collaborations to provide virtual or remote services -- Additional perspectives, including challenges and opportunities, on how libraries overall continue to work and provide services, and resources Participants will have opportunities to discuss with presenters how they implemented the tools at their disposal in innovative and creative ways.

Methods: Session organizers will work with the panelists and share the learning outcomes to guide their development of their presentation for the immersion session. With the panelists, organizers will prepare facilitation methods to encourage open discussion via chat, audience response polls, or other methods. Organizers will share with all participants: related resources and opportunities for further learning on the topics included in the session.

Participant Engagement: We will foster and encourage audience participation by: -- See the Session web page for detailed panelist bios and more session info, including ways to engage with panelists and others or share resources, questions, and ideas before the session: https://guides.lib.vt.edu/MLANET21_HealthAndLibrariesInCOVID -- Join in the pre- and post-session Twitter discussion May 19-28, 2021 at the hashtags: #LibrariesAndCovid #mlanet21

Sponsor(s): International Cooperation Caucus

Health Informatics: A Beginner’s Guide to Meeting Diverse Community Needs

Practice area: Innovation & Research Practice

Alison P. Gehred - Reference Librarian, Nationwide Children's Hospital, Columbus, OH

Nancy Shin - Research and Data Coordinator, NNLM PNR, Seattle, WA

HelenAnn B. Epstein, MLS MS AHIP FMLA – Informationist, Virtua Health Sciences Library, Monroe, NJ

Emily M. Johnson-Barlow, AHIP - Associate Professor & Regional Health Sciences Librarian, UIC Library of the Health Sciences – Peoria, Peoria, IL

Objective: With technology and large amounts of health data becoming more accessible, more and more health sciences librarians are expanding their roles in health informatics. This interactive session will allow librarians with a background in health informatics to discuss the basics of this field and teach participants how to apply this information in their practice and to the communities they serve. Panelists will relate their areas of expertise and illustrate how their work meets the health informatics field characteristics. Participants will engage in small-group breakout discussions to identify informatics in their own work and brainstorm opportunities for further involvement in their own environment. Overall, the goals of this session will lead to a better understanding of the informatics field.

Methods: Panelists will break down the session into three parts based on the American Medical Informatics Association definition of biomedical informatics. The panelists will lead sessions focusing on their area of expertise in the field. The end of each part will include a poll question to help participants interact with the content. Participants will answer the question and be randomly separated into breakout rooms where a panelist will guide them through the scenarios in the polls. Participants will discuss how these topics apply to their work. The group will reconvene with panelists summing up what happened in the breakout rooms and sharing their expertise. This will continue until the last session, where participants will be asked to apply what they know to the communities they serve and how informatics can be used for better health outcomes. The last part of the presentation will be a debriefing and then a panelist question and answer period. Participants will engage with each other in a cooperative learning environment. ○ Draft schedule: 5 min Whole group Welcome to presenters, overview in the context of the definition focusing on scope and application in transforming diverse groups. 15 min Whole group The presenters talk about the scope and breadth of biomedical informatics. Participants will engage in polling about the scope and breadth from their environment. We will sort into random breakout rooms to discuss how broad the applications are. 10 min Small groups Discuss the results of polls and give examples of scope and breadth in their professional life. 15 min Whole group Moderators choose one question from the breakout rooms and lead into the next session about talk about theory, methodology, and the technological approach of health informatics. We will sort into random breakout rooms to discuss the above. 10 min Small group Discussion with small groups. 15 min Whole groups

Panelists will discuss the results of polls and give examples of case studies in their own experience. Presenters will then discuss the human and social context of informatics and ask participants how they can apply their basic understanding of this field to better serve their community. Participants will randomly be sorted into breakout rooms. 10 min Small group Discussion with small groups. 10 min Whole group Summarize generated knowledge; Q&A with presenters

Participant Engagement: Participants will actively participate in guided small group discussions throughout the immersion session in breakout rooms. The chats will be moderated, and a panelist will respond to individuals with questions. There will be a panelist monitoring the chat to keep track of participant questions. The use of interactive polls will be part of the session so that participants can interact with the panelists and the group. There will also be an open Google Document for participants to write down their thoughts and questions. The link to this document will be sent out to all participants via email after the session. A hashtag will also be created so participants can follow up on Twitter and engage with each other there. A panelist will also moderate the chat feature for people who want to chat and people who may be blocked from Google Drive by their employer. Participants will be encouraged to participate however they feel is appropriate with panelists encouraging creativity and openness as they learn more about health informatics. The use of cloud technologies will allow participants to participate in various programs and allow the panelists to translate participants’ questions into applicable practice. At the end of the session, there will be a question and answer session so participants can discuss what was learned. The panelists will also supply some supplemental reading so participants can continue learning at home.

Sponsor(s): Medical Informatics Caucus, Clinical Librarians and Evidence-Based Healthcare Caucus, Innovation and Research Practice Hub, Hospital Library Caucus

Leadership and Management Best Practices for Leveraging Community Connections to Improve Access to Health Information

Practice area: Professionalism & Leadership

Martha E. Meacham – NNLM Project Director, National Library of Medicine, Litchfield, NH

Tony Nguyen, MLIS, AHIP – Executive Director, Network of the National Library of Medicine, Southeastern/Atlantic Region, Baltimore, MD

Tess Wilson, MLIS, MFA - All of Us Community Engagement Coordinator, Network of the National Library of Medicine, Middle Atlantic Region, Pittsburgh, PA

Objective: This session will use hands-on exercises to engage attendees in the development of their own definitions of and strategies for community engagement through a leadership lens.

Additionally, attendees will gain practical skills they will be able to implement which demonstrate and exemplify leadership skills and practices.

Methods: This session will present some material in a traditional presentation format, but the primary means of instruction will be with active learning techniques. The beginning of the session will involve some pre-instruction polling that will be used to gauge attendee’s current knowledge. The end of the session will include activities that gauge knowledge and understanding after the session material is covered.

Participant Engagement: While there will be some material presented to the attendees, this session will primarily use several different active learning techniques. This includes, polling and other means of gathering input from attendees; small group exercises; think-pair-share; individual reflection.

Learning to Teach All Over Again: Learning With, From, and About Each Other to Engage Students in Hybrid and Online Environments

Practice area: Education

Kayce Gill - Lipscomb University, Nashville, TN

Emily Gorman, AHIP - Research and Education Librarian, University of Maryland, Baltimore

Hilary M. Jasmin - Assistant Professor/Research and Learning Services Librarian, University of Tennessee Health Science Center, Health Sciences Library, Research and Learning Services, Memphis, TN

Christina M. Seeger, AHIP - Pharmacy & Health Science Center Librarian, Medical Sciences Library, College Station, TX

Melissa L. Hunter – Drug Information Director, University of Wyoming School of Pharmacy, Laramie, WY

Objective: The advent of COVID-19 forced many instructors and librarians to quickly transition their classes from an in-person to a hybrid or virtual format in the spring of 2020. A year later, and many instructors and librarians are still teaching in a hybrid or virtual environment. One of the greatest challenges of that environment is how to ensure that active learning remains an important component of teaching and student engagement. In this immersion session, a panel of librarians and a drug information pharmacist will discuss how they adapted their teaching methods, used active learning and technology tools to engage students, and relied on a cross-institutional peer mentoring group to share ideas, successes, and failures to thrive in an unprecedented situation.

Methods: In this moderated panel discussion, panelists will share specific examples from their experiences of shifting classes and active learning activities from an in-person to a hybrid or online format for teaching drug information and literature evaluation. Panelists will also briefly demo some of the tools they used to engage students in asynchronous and synchronous hybrid and online classes. Tools will include Google Docs, LibGuides, Mentimeter, Padlet, PollEverywhere, and Zoom. The panelists will discuss their experiences and perceptions of using a peer mentoring group to learn from others with similar backgrounds. After the panel discussion and demos, attendees will have the opportunity to participate in one of several facilitated group discussions. Topics may include active learning ideas, gamification, peer mentoring groups, teaching drug information online, technology tools, and more.

Participant Engagement: Attendees will be able to participate in Mentimeter questions at the beginning of the session to identify audience demographics such as type of librarian or instructor and their experiences of teaching during the COVID-19 pandemic. Attendees will have the opportunity to share their ideas and tools in smaller, facilitated group discussions. Each group leader will create a Padlet to capture ideas and key takeaways. Each group leader will share at least one idea with the larger group. The session will wrap up with a Q&A time.

Sponsor(s): Pharmacy and Drug Information, Technology in Education, Clinical Librarians and Evidence-Based Healthcare, Libraries in Health Sciences Curriculums

Legislative Update: Advocating for Medical Libraries with MLA: Successes, Challenges, and Future Efforts

Practice area: Professionalism & Leadership

Mary M. Langman - Director, Information Issues & Policy, Medical Library Association, Chicago, IL

Margaret Ansell, AHIP - Associate Chair, Gainesville, Health Science Center Libraries, Gainesville, FL

Andy Hickner - Education and Outreach Librarian, Weill Cornell Medicine, Astoria, NY

Chris Shaffer, AHIP - University Librarian and Assistant Vice Chancello,r University of California, San Francisco

Teresa Zayas Caban, PhD - Assistant Director for Policy Development, National Library of Medicine, National Institutes of Health

Objective: The session moderator will review the accomplishments of the association’s 2021 Capitol Hill meetings. A representative from the National Library of Medicine will give a Legislative Update. Following the presentation, participants will have the opportunity to share their recent experiences meeting with their Congressional representatives, which took place

during MLA ‘21 Exploration weeks (May 10-21). At least 30-45 minutes will be dedicated to small group discussions among members from the same state or region.

Methods: The session moderator will review the accomplishments of the association’s 2021 Hill meetings, including Zoom meetings to identify the association’s key legislative priorities; coaching participants through the process of scheduling their meetings with Congressional staff and other logistics; and preparing participants for the meetings. An NLM representative will give an update on the latest legislative issues relevant to NLM. Small group discussions using breakout rooms will be used in order to reinforce the learning outcomes and allow participants to identify and share lessons learned from their recent experiences with advocacy on the Hill.

Participant Engagement: We will engage participants using breakout rooms, which were used effectively in the 2020 immersion session. We will allocate 30-45 minutes for small group discussions in breakout rooms, organized geographically to allow for conversation between participants from the same state or region.

Sponsor(s): MLA Governmental Relations Committee MLA/AAHSL Joint Legislative Task Force

Let's Talk: LGBTQIA+ Inclusion and Equity at Your Workplace

Practice area: Professionalism & Leadership

Hannah Schilperoort, MLIS, MA - Head, Wilson Dental Library, USC Libraries, Los Angeles, CA

April Wright, MLS - Environmental Health Program Specialist, NNLM/University of Maryland, Baltimore

Brandi Tuttle, AHIP – Research and Education Librarian, Duke University Medical Center Library & Archives, Durham, NC

Brenda M. Linares, AHIP – Health Sciences Librarian, A.R. Dykes Library, University of Kansas Medical Center, Kansas Coty, KS

Emily Vardell, AHIP – Assistant Professor, Emporia State University School of Library and Information Management, Olathe, KS

Jacqueline Leskovec – Network Li brarian, NNLM GMR, Chicago, IL

Jane Morgan-Daniel, AHIP - Community Engagement and Health Literacy Liaison Librarian, Health Science Center Libraries, University of Florida, Gainesville, FL

Mary Catherine Lockmiller, AHIP – Health Sciences Librarian, Cline Library / Phoenix Biomedical Campus, Tempe. AZ

Meredith I. Solomon, MLA, AHIP – Senior Outreach Officer, Countway Library of Medicine/Outreach Office, Quincy, MA

David G. Keddle, BA – Medical Library Services Director, Health Sciences Library, Woodland Hills, CA

Session Format: This flipped immersion session will include a pre-recorded video and handout for participants to view before the live session. The live session will be interactive and include a panelist Q & A and facilitated breakouts. Participants are invited to come with questions, ideas, and challenges that relate to work you are doing or want to do at your library or institution.

Please watch this video before the live session: https://youtu.be/dqS0vL9aqFI

Objective: Are you looking for practical and concrete ways to support LGBTQIA+ participants at your library or institution? Join your colleagues to discuss real-life examples of inclusive LGBTQIA+ policies, programs, services, collections, and spaces. Learn from your colleagues and share your own experiences and expertise, with the main objective of developing potential initiatives to deploy at your own institution.

Methods: The session is based on a flipped instructional model, in which participants review the provided video and handout before the session, so that the live session can focus on hands-on engagement and project planning.

Participant Engagement: The session is designed for individuals with some knowledge of LGBTQIA+ inclusive language and practices who are interested in planning practical strategies for implementing LGBTQIA+ initiatives at their own libraries. Before the session, participants are encouraged to review the provided video and handout. During the breakouts, facilitators will guide participants through a vision and goal setting worksheet to start the planning process.

Sponsor(s): LGBTQIA+ Caucus, African American Medical Library Alliance Caucus, Latinx Caucus, Leadership and Management Caucus, Social Justice Caucus

Managing Change When You’re Not in Charge: How to Keep Your Sanity During Organizational Change

Practice area: Professionalism & Leadership

Catherine B. Soehner, AHIP - Associate Dean for Research and Director, Spencer S. Eccles Health Sciences Library, Salt Lake City, UT

Objective: In this session, you will learn tools to survive and keep your sanity in an ever-changing environment. You will discover your power in an organization, possible ways to deal with the problems you see, and how to give recommendations to your boss. You will get clear about where you have and do not have control in your library.

Methods: Instructional methods will include presentation of concepts with intermittent opportunities for discussion with the instructor and one another. Participants will be asked to consider hypothetical conditions and apply new knowledge and skills to these situations with discussion in pairs or small groups. There will be opportunity for large group interactions and discussions. A bibliography listing all sources will be provided to participants for follow up reading.

Participant Engagement: Participants will be actively involved in the session through large group interactions over chat along with breakout rooms that may include paired or small group discussions. Hypothetical situations will be presented to allow participants to apply new knowledge immediately and challenge long-held beliefs and perspectives.

Managing Change: How to Lead in an Ever-Changing World

Practice area: Professionalism & Leadership

Catherine B. Soehner, AHIP - Associate Dean for Research and Director, Spencer S. Eccles Health Sciences Library, Salt Lake City, UT

Objective: Learn how to invoke change with the least amount of upset and the most movement toward stated goals. Discover systems thinking and consider how people in organizations typically respond to change, how you respond to change, and how this affects the overall library. Understand how to respond to feedback to better hear concerns from your employees and to better evaluate that feedback for inclusion in a change process. Apply this new knowledge to help change interpersonal dynamics within your organization.

Methods: Instructional methods will include presentation of concepts with intermittent opportunities for discussion with the instructor and one another. Participants will be asked to consider hypothetical conditions and apply new knowledge and skills to these situations with discussion in pairs or small groups. There will be opportunity for large group interactions and discussions. A bibliography listing all sources will be provided to participants for follow up reading.

Participant Engagement: Participants will be actively involved in the session through large group interactions along with breakout sessions that may include paired or small group

discussions. Hypothetical situations will be presented to allow participants to apply new knowledge immediately and challenge long-held beliefs and perspectives.

My Favorite Tool

Practice area: Education

Margaret A. Hoogland AHIP - Clinical Medical Librarian, Mulford Health Science Library,Temperance, MI

Kerry Dhakal - Assistant Professor, Research and Education Librarian, The Ohio State University

Paul C. Gahn - Assistant Director/Associate Professor, Electronic and Collection Services, University of Tennessee Health Science Center, Health Sciences Library, Memphis, TN

Hannah J. Craven MLIS - Research & Scholarly Communications Librarian, Ruth Lilly Medical Library, Indianapolis, IN

Katherine V. Chew - Research/Outreach Services & Mortuary Science, Center for Allied Health Programs and Center for Bioethics Librarian, University of Minnesota Health Sciences Libraries, Minneapolis, MN

Kathryn Vela AHIP - Medical Librarian, St. Luke's Health System, Boise, ID

Rebecca Carlson MLS, AHIP - Liaison to the Eshelman School of Pharmacy, University of North Carolina at Chapel Hill, Chapel Hill, NC

Mirian Ramirez - Research Metrics Librarian, Ruth Lilly Medical Library Indianapolis, IN

Ivan A. Portillo AHIP - Coordinator of Rinker Campus Library Services, Chapman University, Irvine, CA

Alysha Sapp - Nursing & Nurse Anesthesia Librarian, Texas Christian University, Fort Worth, TX

C. Erik Wilkinson - Regional Library Director, Texas Tech University Health Sciences Center, Odessa, TX

Tara Brigham AHIP - Librarian, Mayo Clinic, Jacksonville, FL

Objective: This pecha kucha format gives each contestant up to 3 minutes (9 slides with 20 seconds per slide) to describe a new tool to audience members, small group moderators, and the session moderator. The presentation styles of this session are simple and spontaneous, which keeps contestants and audience members engaged. It is innovative, because at least

one aspect is refined or adjusted each year. For 2021, it will be ready for either an in-person or virtual meeting. It’s popular. In 2018 and 2019, the “room” was packed for this session. Whatever space (virtual or physical) is allotted, anticipate standing room only and arrive early to “claim” a seat.

Methods: Participants receive instructions at the beginning of the session on how to vote and what to expect during the discussion sessions. Powerpoints, handouts, videos, and pdfs of tools are prepared by the contestants for the audience participants.

Participant Engagement: Participants are encouraged to vote after each contestant. The votes inform the contestant on how to improve and the top 3 contestants win prizes.

On the Edge of Innovation: How Libraries Can Develop Health Datathons and Hackathons for Data Literacy

Practice area: Information Management

Bethany S. McGowan, MLIS, MS, AHIP - Associate Professor, Libraries and School of Information Studies, Library of Engineering and Science, West Lafayette, IN

Peace Ossom Williamson, AHIP - Director of Research Data Services, The University of Texas at Arlington

Nancy Shin – Research and Data Coordinator, NNLM PNR, Seattle, WA

Objective: This panel presentation will provide participants with information about hosting and participating in data competitions, including hackathons, datathons, and mapathons. These are project “marathons,” in which a team comes together to devise a plan and see an entire project through from start to finish under a shared theme. Projects might include programming skills, 3D printing, design, research skills, building resources, or anything that is within the bounds of the competition, if any bounds are set. The panelists will describe the role of data competitions for building awareness and knowledge; will discuss the management and use of open data sources--many of which can come in a variety of formats, and will detail the different aims of mapathons, hackathons, and other similar events. Attendees will be provided the tools and processes needed to plan their own events and establish campus buy-in.

Methods: We will begin with a welcome and a brief introduction of the presenters, then the presenters will give a brief introduction, overview, and history of data competitions and will summarize research supporting their effectiveness as instructional tools (10 mins). Participants will then be provided with a data competition simulation activity where they build a resource around a particular theme (15 mins). Following the activity, the panelists will each describe the competitions they have planned, along with relevant research assessing the success of these programs (15 mins each). The session will then open up to three break out sessions where

people can actively discuss their concerns and successes with a type of data competition - i.e., hackathon, datathon, and mapathon (20 mins). We will end by regrouping with an open question and answer period (15 mins).

Participant Engagement: Throughout the session, the presenters will encourage feedback, brainstorming, and sharing of techniques and sources for facilitating data competitions as well as interactive activities for building enthusiasm.

Sponsor(s): Data Caucus

Research 101

Practice area: Innovation & Research Practice

Abby L. Adamczyk AHIP - Graduate Medical Education Librarian, Scott Memorial Library, Philadelphia, PA

Nina Exner - Research data librarian, Virginia Commonwealth University Libraries, Richmond, VA

Rebecca Carlson MLS, AHIP - Liaison to the Eshelman School of Pharmacy, University of North Carolina at Chapel Hill, Chapel Hill, NC

Emily Vardell AHIP - Assistant Professor, Emporia State University School of Library and Information Management, Olathe, KS

Dede Rios AHIP - Director of Optometric & Clinical Library Services, University of the Incarnate Word Libraries/Optometry, San Antonio, TX

Objective: This session is designed to introduce librarians and information professionals to the research process. It will consist of panel presentations and case studies from research experts that will introduce important concepts and methods and give examples of how to apply them, and group discussions and interactive polls to engage attendees with the content. Attendees will gain practical information and resources to be able to conduct their own research in the future. A partner “Research 102” session is also proposed to allow for ongoing engagement.

Methods: Static content will be shared in advance, to shift the live session towards discussion and engagement. We will use polling to determine audience interests and starting points, in order to ensure responsiveness to reach the objectives. Panelist interaction will help to give multiple perspectives.We will also share multiple resources for individuals to continue to develop their own skills.

Participant Engagement: We plan to use case study discussions with practical points to encourage Q&A and discussion. Furthermore, the IRP-DH survey identified this as a topic of high need for many members.

Sponsor(s): Research Caucus, Medical Library Education Caucus, New Members Caucus

Resilience Engineering: A Guide Toward Persistent Knowledge Services Even in Disruptive Times

Practice area: Professionalism & Leadership

Nicole Capdarest-Arest AHIP - Head, Blaisdell Medical Library, University of California, Davis, Sacramento, CA

Sara R. Tompson - Independent Engineering & Science Researcher, Research Solutions, Lawrence, KS

Lorri Zipperer - Digital Project and Systematic Review Librarian, Blaisdell Medical Library, Albuquerque, NM

Objective: Resilience is found in adaptive, complex systems, like health care and aviation, which have integral features that encourage and require resilience to protect safety. Given the current challenges of our society (e.g., pandemics, cyber-attacks, natural disasters, civil unrest, radical disparities, harmful adverse events, downsizing, mergers), the resilience engineering model offers a way to design systems and organizations to better accommodate such disturbances. This interactive session will offer participants opportunities to learn about resilience engineering and share ideas of how health sciences librarians might use resilience engineering strategies to make our organizations more agile in dealing with and learning from such disruptions.

Methods: The facilitators, who each have interest in the topic of resilience engineering and its applicability to health sciences librarians, will introduce resilience engineering strategies, how they impact organizational resilience, and provide a case study of resilience engineering in practice in a health sciences library. Participants will then engage in interactive small-group discussions using structured questions and frameworks in a variety of library types. The small groups will explore themed topics relating to the session's learning outcomes and discussions will be synthesized for the entire group at the end of the session. In this way, participants will have opportunities to broaden their individual knowledge and skills around the learning outcomes in a cooperative, learning-focused environment.

Participant Engagement: Participants will actively participate in guided small-group discussions using structured prompts and frameworks (e.g., resilience engineering components such as Monitor, Respond, Anticipate, Learn). There will also be opportunities to

share across groups so that knowledge generated in the small groups can be assimilated by everyone, as well as to ask questions of the facilitators.

Sponsor(s): Medical Informatics Caucus; Clinical Librarians and Evidence-based Healthcare Caucus; members of the Special Libraries Association

Striving for Deep Engagement: Cultivating a Library Environment Where Everyone Feels Included

Practice area: Professionalism & Leadership

Shannon D. Jones AHIP - Director of Libraries, Medical University of South Carolina Library, Charleston, SC

Daina Dickman AHIP - Scholarly Communication Librarian, California State University, Sacramento

JJ Pionke - Applied Health Sciences Librarian, University of Illinois at Urbana-Champaign, IL

Tamara M. Nelson AHIP - Associate Professor/Senior Research & Learning Services Librarian, University of Tennessee Health Science Center, Health Sciences Library, Research & Learning Services, Memphis, TN

Mary Catherine Lockmiller AHIP - Health Science Librarian, Cline Library / Phoenix Biomedical Campus, Tempe, AZ

Brenda M. Linares AHIP - Health Sciences Librarian, A.R. Dykes Library, University of Kansas Medical Center, Kansas City, KS

Kelly Thormodson - Associate Dean and Director, Penn State University Harrell Health Sciences Library, Hershey, PA

Susan Swogger - Librarian, , Kirksville, MO

Kelsa Bartley - Education and Outreach Librarian, Louis Calder Memorial Library, University of Miami Miller School of Medicine, Miami, FL

Hannah Rutledge PhD, MLIS, AHIP - Director, Biomedical Library, University of Pennsylvania Libraries, Philadelphia, PA

Session Format: Moderated roundtable discussions focused on inclusive practices for shaping meaningful library experiences for individuals from marginalized groups.

Objective: The news and scholarly literature are replete with stories describing the challenges that individuals from marginalized groups face in their local communities and workplaces. Fulfilling the Library’s mission to its parent organization requires that those of us who work in libraries to create environments that resist oppression and are welcoming, inclusive, and safe for people who experience marginalization because of their: race, age, ethnicity, economic class, body size, religion, sexual orientation, gender identity, gender expression, relationship status, faith status, ability status, or health status. Participants will be invited to share their experiences, frustrations, challenges, and solutions to providing inclusive library environments.

Methods: Participants will be encouraged to read 1 to 2 articles or book chapters prior to attending the session. To set the stage for dialogue, the facilitators will introduce the guidelines for respectful discussion at the beginning of the session. Then, the facilitators will lead participants in an interactive dialogue aimed at achieving each learning outcome. Each group will be provided a case study related to the discussion topic and discussion questions to guide the conversation. Each session will conclude with a wrap up.

Participant Engagement: Participants will participate in interactive small group discussions where the focus will be to share practical strategies, barriers, and lessons learned on how we can actively recruit, retain, and meet the information needs of individuals from marginalized groups in our libraries. One or more live polls will be embedded using an audience response system. There will be time built into our schedule for audience Q&A. Participants will be invited to co-create a Google Doc of strategies and best practices that will be shared at the session’s conclusion.

Sponsor(s): Accessibility and Disability African American Medical Librarians Alliance Latinx Leadership and Management LGBTQIA+ New Members

The Great Disconnect: Challenges of Getting Users to Full Text

Practice area: Information Services

Andy Hickner - Education and Outreach Librarian, Weill Cornell Medicine, Astoria, NY

Eric Phetteplace - Systems Librarian, California College of the Arts Libraries, Oakland, CA

Lindsay Barnett - Collection Development and Scholarly Communication Librarian, Cushing/Whitney Medical Library, Yale School of Medicine, New Haven, CT

Angela Spencer, AHIP - Health Sciences Reference Librarian, Medical Center Library, St. Louis, MO

Basia Delawska-Elliott, AHIP - Health Sciences Education, Research and Outreach Librarian, Oregon Health & Science University, Portland, OR

Objective: In this immersion session cosponsored by the Hospital Library, Solo Librarians, Technical Services, and User Experience caucuses, we will explore one of the challenges identified in recent summits of the MLA InSight Initiative - specifically, getting full text of articles to library end users. It’s one of the biggest frustrations for both librarians and users, and can be particularly challenging for smaller, less-resourced libraries that don’t have access to in-house developers and UX departments. Topics discussed will include NLM’s Library LinkOut using Outside Tool, subscription-based tools such as LibKey and Kopernio, designing more intuitive link resolver interfaces, and the inherent flaws of discovery layer linking.

Methods: Our immersion session will be led by a moderator and a panel of librarians representing both the back-end and end-user-facing perspectives. Panelists will share case studies from their own institutions. We will include time for moderated Q&A with our panel during the session. We will share electronic versions of supporting materials, including recommendations for further reading on the topic.

Participant Engagement: Participant engagement strategies for this session will include live polling; think/pair/share and small group activities using Zoom breakout rooms; and possibly a learning game tool such as Kahoot!.

Sponsor(s): Hospital Library, Solo Librarians, Technical Services, and User Experience caucuses

We Got You! Empowering BIPOC Information Professionals through Virtual Development Opportunities

Practice area: Professionalism & Leadership

Tyler Moses - Assessment and Life Sciences Informationist, Woodruff Health Sciences Center Library, Atlanta, GA

Aidy Weeks MSLIS, AHIP - Interim Director/GME Liaison Librarian & Collections Manager, UNLV Libraries, Las Vegas, NV

Xan Goodman AHIP - Health Sciences Librarian, Lied Library/ RED Division, Henderson, NV

Beverly Murphy, MLS AHIP, FMLA - Assistant Director, Web Content & Development; DUHS Hospital Nursing Liaison: Watts CON Liaison, Duke Medical Center Library & Archives, Durham, NC

Shannon D. Jones AHIP - Director of Libraries, Medical University of South Carolina Library, Charleston, SC

Kelsa Bartley - Education and Outreach Librarian, Louis Calder Memorial Library, University of Miami Miller School of Medicine, Miami, FL

Jamia J. Williams - Health Sciences Librarian, SUNY Brockport, Rochester, NY

Tamara M. Nelson AHIP - Associate Professor/Senior Research & Learning Services Librarian, University of Tennessee Health Science Center, Health Sciences Library, Research & Learning Services, Memphis, TN

Objective: The purpose of this session is to introduce and describe activities of AAMLA’s Virtual Engagement Committee in building community and support for Black, Indigenous, People of Color (BIPOC) information professionals throughout the year. This immersion session will cover strategies and processes used to strengthen programmatic engagement for use as a model for other Caucuses. Presenters will discuss the steps needed to implement successful programming, identify roles involved, impart best practice strategies, and offer tools for tracking progress.

Methods: Information and resources will be provided to participants in a mutually engaging, hands-on format. Presenters will provide an overview of how the Virtual Engagement Committee was established, define virtual engagement, and discuss its benefits. In addition, participants will be instructed on appropriate data collection strategies, how to generate programming ideas, and create a strategy/plan for their own Caucus using polling and breakout rooms.

Participant Engagement: Participants will have the opportunity to brainstorm programming ideas, create a strategic plan for implementing them, and be invited to co-create a Google Doc that will be shared at the session’s conclusion. One or more live polls will be embedded throughout the session using an audience response system and there will be time built into our schedule for audience Q&A.

Sponsor(s): AAMLA Caucus MLA Latinx Caucus

Research 102

Practice area: Innovation & Research Practice

Abby L. Adamczyk AHIP - Graduate Medical Education Librarian, Scott Memorial Library, Philadelphia, PA

Tony Nguyen MLIS, AHIP - Executive Director, Network of the National Library of Medicine, Southeastern/Atlantic Region, Baltimore, MD

Nina Exner - Research data librarian, Virginia Commonwealth University Libraries, Richmond, VA

Helena VonVille - Research and Instruction Librarian, University of Pittsburgh Health Sciences Library System, Pittsburgh, PA

Kristine M. Alpi MLS, MPH, PhD, AHIP, FMLA - University Librarian, OHSU Library, Portland, OR

Objective: This session is designed to extend the knowledge and skills of librarians who feel comfortable with the introductory concepts of the research process. The session will consist of panel presentations and case studies from research experts, focusing on the relationship of theoretical frameworks and study design, protocol development, project funding, and managing research data. The knowledge and skills gained in this session will provide a solid foundation for the development of a well-crafted research project. This session follows the basic concepts introduced in the partner “Research 101” session.

Methods: Content will be shared in advance allowing for the live to session to involve more participant engagement in questions and discussion. Panelists will give a short presentation on their specific topic. Then attendees will choose breakout rooms for deeper discussion with that topic with the panelist. Questions will be collected in advance to help make the discussions more relevant to attendees.

Participant Engagement: Topical group breakout discussions will give attendees the opportunity to share their experiences and discuss the challenges that are most relevant to them. Moderators will bring experience and discussion prompts on the specific topic, to help ensure engaged discussion.

Sponsor(s): Research Caucus, Medical Library Education Caucus, New Members Caucus

PAPERS: RESEARCH ABSTRACTS

Papers in this section are sorted by title in alphabetical order.

Aligning a Medical Science Library’s Instructional Vision for Local Professional Health Programs Curricula Using the Steps of Evidence-Based Practice

Practice Area: Education

Micah J. Waltz, Lecturer, Texas A&M University, College Station, TX

Heather K. Moberly, AHIP, Professor/Coordinator of Veterinary Information and Research Services, Texas A&M University Libraries / Medical Sciences Library, College Station, Texas

Catherine Pepper, Associate Professor/MSL Regional Services Coordinator, Medical Sciences Library, Texas A&M University, Austin, Texas

Rachel Blume, Public Health Librarian, Texas A&M University Medical Sciences Library, ,

Sheila W. Green, AHIP, HSC Bryan Campus Librarian, Texas A&M University Medical Sciences Library, Texas

Christina M. Seeger, AHIP, Pharmacy & Health Science Center Librarian, Medical Sciences Library, College Station, Texas

Stephanie Fulton, AHIP, Associate Dean and Director, Texas A&M University Library / Medical Sciences Library, Bryan, Texas

Objectives: This research continues to examine how information skills and behaviors are present in the program-level curricular documents from five professional health programs at one university, including a veterinary curriculum. Each program’s accrediting bodies were also examined to compare how they presented information skills and behaviors with the programs’ curricula. These data will be framed within the steps of evidence-based practice (EBP) to develop local sustainable, data-driven instruction to support populations with diverse instructional needs and student bodies.

Methods: Using a previous generated seven-category rubric for qualitative coding, each curricular outcome from all five local professional health programs was qualitatively coded using an inductive approach. This coding was done by analyzing individual curricular outcomes’ verbs. The intent of each curricular outcome was identified by using the primary action verb.

The mission statements and any outcomes for the accrediting bodies for each of the five local professional health programs were also coded with the same rubric to identify how information seeking behaviors and skills are represented.

The information from the local programs’ curricula was mapped to the steps of EBP. This provided a framework for moving towards local, sustainable instructional support.

Results: All five programs had curricular outcomes which implied information skills and behaviors—such as competencies about lifelong learning. An noticeable emphasis of the curricular outcomes was evaluating information.

This emphasis on evaluating information across the local curricula highlights an emphasis on EBP. Mapping the local programs’ curricular competencies to EBP highlights the multiple needs for health librarians in developing and providing contextualized instruction about how to access, identify, and evaluate information necessary to generate the evidence for a decision.

Conclusions: The program-level curricular documents contain valuable data about what information skills and behaviors are being emphasized in the local health professional curricula. A common emphasis across the local health professional curricula is demonstrating the ability to “evaluate” information. Mapping the local curricula to the steps of EBP provides a framework for contextualizing how to instruct information skills and behaviors. This provides a direction to meet the instructional needs of the local programs.

Are There Retractions in Your Systematic Review? Findings from the Pharmaceutical Literature

Practice Area: Innovation & Research Practice

Caitlin Bakker, AHIP, Research Services Librarian, University of Minnesota, Minneapolis, Minnesota

Sarah Jane Brown, Assistant Librarian, Health Sciences Library, University of Minnesota, Minneapolis, Minnesota

Nicole Theis-Mahon, AHIP, Librarian Liaison & Collections Coordinator, University of Minnesota, Minneapolis, Minnesota

Objectives: Systematic reviews are considered the apex of the evidence pyramid, as they embody comprehensive literature searches and rigorous assessments of methodological quality. Despite this, flawed research is still being incorporated into these publications. In this follow-up to our presentation of preliminary findings on the citation and use of retracted articles in higher levels of evidence in the field of pharmacy, we describe the characteristics of retracted publications cited in systematic and other evidence-based reviews and discuss the factors that may be associated with the likelihood of citation.

Methods: Based on data provided by the Retraction Watch database, we identified retracted articles in pharmacy, toxicology, and drug design. We identified all articles citing these publications in Scopus and Web of Science before deduplicating these results and screening to identify systematic and other evidence-based reviews. We then identified whether the citation occurred prior to or following retraction of the original article, and analyzed which factors may be associated with the likelihood of a systematic review citing a retracted publication, both before and after its retraction.

Results: Of 1,396 retracted publications, 283 were cited in 859 systematic reviews. Approximately 66% of the citations occurred before the retraction, and 34% after. Items were most frequently retracted due to data falsification or manipulation (38%), ethical misconduct including plagiarism (30%), or concerns about or errors in data or methods (28%). Compared to retracted articles not cited in systematic reviews, cited items were significantly more likely to be retracted due to data falsification and manipulation ($p < .0001$), to be published in high impact factor journals ($p < .0001$), and to have longer delays between publication and retraction ($p < .0001$).

Conclusions: Retractions present challenges for researchers and librarians conducting and reading systematic reviews. Using retracted literature appropriately requires being able to identify and assess that literature. Librarians have a role in teaching critical thinking skills and can incorporate methodological assessment into instruction to provide students with the skills needed to assess systematic reviews. Additionally, librarians are in a unique position to contextualize problems associated with continued citation of retracted articles and advocate for transparency around the retraction process. This perspective can be used to build awareness of the challenges that retractions pose in systematic reviews among health sciences researchers, faculty, and students.

Compliance of Pediatric Preoperative Fasting Guidelines: A Look at the Literature

Practice Area: Information Services

Linda Delwood, Graduate Student, University of North Texas College of Library and Information Science, Irving, Texas

Ana D. Cleveland, AHIP, FMLA, Director of Health Informatics Program, University of North Texas, Denton, Texas

Objectives: Parents/caregivers frequently do not comply with preoperative fasting guidelines for healthy pediatric elective surgery patients which increases their risk for a number of complications. The purpose of the literature review was to identify the reasons for the lack of compliance and identify informatics solutions.

Methods: The databases, PubMed, CINAHL Complete, and Scopus were searched from September 2015 to September 2020. Search terms used were “preoperative fasting”, “fasting

guidelines”, “child”, “child 0-3 years”, “child 3-6 years”, “surgery”, “anesthesia”, and “smartphone apps”. The result of each search was recorded. Duplicate articles were eliminated. For each article, the abstract and summary were read for relevance. Articles of high relevance were read in its entirety. Compliance issues and informatics applications were recorded and analyzed.

Results: Searches “pediatric fasting guidelines” and “anesthesia” as well as pediatrics AND “fasting guidelines” AND anesthesia yielded 86 results with high relevance. There was an agreement regarding pediatric preoperative noncompliance with fasting guidelines by parents or caregivers. It appears to result from not understanding instructions, what “fasting” means, or the reason for fasting; there are occasional lapses of supervision during which the child accesses food and situations in which the parents intentionally mislead anesthesia providers. A number of studies reported positive results on mobile health systems using smartphone apps to treat a variety of conditions. Other informatics solutions were presented.

Conclusions: The literature indicates that key stakeholders in health care organizations fail to effectively communicate pediatric preoperative fasting guidelines to parents/caregivers of healthy pediatric elective surgery patients in an effective way. Also, reasons for the failure of compliance is the health literacy level of parents/caregivers. Effective methods to improve communication needs to be explored such as m-health, patient portals, SMS, and apps. From the literature review, there is potential for informatics to improve compliance of preoperative fasting guidelines. There is a need for collaboration between health care providers, and medical librarians to develop effective information tools for parents and caregivers of pediatric patients regarding preoperative fasting guidelines.

Considerations for Conducting Evidence Syntheses: Evaluating the Performance of Various Electronic Methods for De-Duplicating References

Practice Area: Information Services

Sandra McKeown, Health Sciences Librarian, Queen's University, Kingston, Ontario 12:00:00 AM

Zuhaib M. Mir, Resident physician, Department of Surgery, Queen's University, , Ontario Canada

Objectives: The objective of this study was to determine the accuracy and performance of commonly-used electronic methods for de-deduplicating references retrieved from searching multiple bibliographic databases.

Methods: A heterogeneous sample of references was obtained by conducting a similar topical search in MEDLINE, Embase, PsycINFO and Cochrane CENTRAL. Manual abstraction was used to identify duplicate references among the search results and develop a gold standard for

comparison. De-duplication methods included Ovid multifile search, EndNote desktop (versions X9 and 20), Mendeley, Zotero, Covidence and Rayyan. False negative and false positive duplicate references for each method were identified and then the accuracy, sensitivity, and specificity of each was calculated.

Results: Default de-duplication settings in Ovid multifile search and dedicated review software (Covidence and Rayyan) significantly outperformed reference management software. Ovid, Covidence and EndNote 20 possessed the highest specificity (1.00, 1.00 and 0.998, respectively) for identifying duplicate references, while Rayyan demonstrated the highest sensitivity (0.96). The accuracy of EndNote desktop improved from the X9 version (0.76) to the newer 20 version (0.92).

Conclusions: This study highlights the strengths and weaknesses of commonly-used electronic methods for de-duplicating references and suggests strategies to avoid unintentionally removing eligible studies and introducing bias into syntheses. When selecting database platforms and utilizing software programs for the review process, de-duplication functionality and performance is an important consideration for improving the efficiency and quality of evidence syntheses.

Consolidating and Delivering Comprehensive Library Services Following a Health Care System Merger or Acquisition: Protocol for a Delphi Study

Practice Area: Professionalism & Leadership

Stacy F. Posillico, Senior Librarian, Northwell Health Eastern Region Hospitals' Libraries, Hempstead, New York

Jaclyn Vialet, AHIP, Senior Librarian, Daniel Carroll Payson Medical Library/North Shore University Hospital, Manhassett, New York

Saori Wendy Herman, AHIP, Head of Education and Access Services, Donald and Barbara Zucker School of Medicine at Hofstra/Northwell, Hempstead, New York

Background: When healthcare system mergers occur, full financial and cultural integration provides the most benefit. While there are well-researched standards for healthcare executives to follow during a healthcare system merger, little empirical research is available for librarians to use in these situations. As a result, librarians are often left to develop their own methods of providing access and services in the newly merged system.

Objective: To describe the design of a Delphi study intended to achieve expert consensus from library administrators in the development of research-based models for delivering library services through one comprehensive, geographically distributed system following a merger or acquisition. This protocol may serve as a research framework for future Delphi studies in library science research.

Methods: The researchers will select, using precise criteria, an expert panel of library leaders from hospitals and healthcare systems within the United States who have recently steered library management following a merger or acquisition. The topics, principles, and standards for the Delphi panel will be preliminarily selected from a targeted literature search. A Delphi consensus survey will be developed, and a timeline to conduct at least 3 rounds to achieve consensus (75% agreement) will be planned. The Delphi survey is subject to Institutional Review Board approval.

Results: This paper will present a fully developed protocol for the Delphi study. The implementation of this Delphi study is anticipated to begin in the Fall or Winter 2021.

Conclusion: Healthcare system mergers are the present and the future of hospital patient care. The proposed research will fill an existing gap in the knowledge as to how to best provide library services as healthcare systems transformatively expand. Hospital librarians, who are often unrepresented at merger negotiations, will be able to use this guidance to address library assimilation after a merger occurs. This paper will provide transparency into the research process, allowing for greater confidence in the results. Librarians will also be encouraged to develop future Delphi studies in other areas.

Diversity and Inclusion in the Network of The National Library of Medicine MidContinental Region: An Environmental Scan

Practice Area: Professionalism & Leadership

David Brown, Wyoming Coordinator, Network of the National Library of Medicine, Laramie, Wyoming

A James Bothmer, AHIP, Consultant, University of Utah Eccles Health Science Library, Omaha, Nebraska

Kiara Comfort, Education and Outreach Coordinator, Creighton University, Omaha, Nebraska

John C. Bramble, Associate Director Network of the National Library of Medicine, MidContinental Region, Eccles Health Sciences Library, University of Utah, Salt Lake City, Utah

Crystal L. Hastings, MLIS student, NNLM MCR Student Intern, Utah

Ellen Thieme, Research Specialist I, Missouri Outreach Support Specialist, J. Otto Lottes Health Science Library, National Network of Libraries of Medicine, MidContinental Region (NNLM MCR), Columbia, Missouri

Objectives: A study was undertaken to investigate diversity and inclusion activities by member libraires in the MidContinental Region (MCR) of the Network of The National Library of

Medicine (NNLM). Through the use of an environmental scan, best practices are identified to increase diversity and inclusion priorities at libraries in the MCR. Lessons learned could easily be adapted by other regions and states.

Methods: The NNLM-MCR conducted an environmental scan of member libraires in the region. The region consists of the following states: Colorado, Kansas, Missouri, Nebraska, Utah and Wyoming. The initial steps included investigating the websites and published materials of member libraries. Based on this review, themes were identified, and recommendations were developed to ensure library programing addresses issues of diversity and inclusion.

Results: Preliminary results indicate a number of member libraries are concerned about issues of diversity and inclusion. However, a majority of the programing being offered at these libraries do not encompass issues of diversity and inclusion. The final project report and results will be completed before the meeting in May.

Conclusions: Diversity and inclusion are major issues facing the profession of health science librarianship. This environmental scan indicates that diversity and inclusion issues are paramount in the region and are major issues facing the profession of health librarianship. This study indicates efforts need to be undertaken to investigate and address issues of diversity and inclusion. Issues of diversity and inclusion should be addressed with a commitment from library administration and staff to understand and address issues of diversity and inclusion. Specific approaches include developing a diversity and inclusion plan for the library. This plan should ensure all members of the library are committed to the implementation of the plan. Additional scans should be conducted to address issues of diversity and inclusion.

Do as I Say, Not as I Do: Reference Rot in Articles Published in the Journal of the Medical Library Association, 2010-2019

Practice Area: Information Management

Sally A. Gore, Manager, Research & Scholarly Communication Services, University of Massachusetts Medical School, Worcester, Massachusetts

Objectives: Citing Internet-based resources is common in scholarship today. The fundamental nature of the Web, however, leaves many originally cited sources unstable, affecting the integrity and reproducibility of the published work. The Hiberlink Project coined the term “reference rot” to describe this problem. The library profession is charged with preserving the scholarly record of research, but how well is our own scholarly record preserved? The purpose of this study is to assess the frequency of reference rot in articles published in the Journal of the Medical Library Association (JMLA) over the past 10 years.

Methods: Standard practice for citing web-based resources in bibliographies is the use of Universal Resource Identifiers (URIs), most commonly in the form of Uniform Resource Locators (URLs). References from all articles published in JMLA between January 2010 and December 2019 possessing a PubMed ID were extracted from PubMed Central. From this set of all references, a subset containing URIs (with the exception of those with Digital Object Identifiers, or DOIs) were identified and then tested on the web to see if they remain active and/or accurate. Frequency of both link rot (broken links) and content drift (working links that now point to different content) within the URIs was determined.

Results: A total of 626 items published in JMLA between the years 2010-2019 are indexed in PubMed. The total number of citations from this corpus is 10,832. Of these, 2,305 (21.28%) are URIs pointing to web-based sites and/or products other than those with minted DOIs. Only 64.21% of these URIs (1,480) continue to accurately lead to the referenced content.

Conclusions: Preservation and access are two key pillars of the library and information profession. Previous research shows the prevalence of reference rot in Science, Technology, and Medicine publications is approximately 20%. The comparably high occurrence (~36%) of this same issue within the professional journal of the Medical Library Association is a threat to both the reproducibility of the Association's scholarly record and to the integrity of the medical and health sciences library profession. Given that the citing of web resources continues to rise in publications, it prompts the need to examine current citation practices and establish new guidelines that will ensure (1) the content referenced in JMLA's publications can continue to be located and validated over time, and (2) that a profession charged with preserving scholarship is doing so.

EBVM Learning II: Updating an Open-Access Evidence-Based Veterinary Medicine (EBVM) Online Tutorial

Practice Area: Education

Heather K. Moberly, AHIP, Professor/Coordinator of Veterinary Information and Research Services, Texas A&M University Libraries / Medical Sciences Library, College Station, Texas

Sarah Baillie, Emeritus Professor of Veterinary Education, University of Bristol, UK

Ellie R. Sellers, Veterinary Clinical Demonstrator, University of Bristol, UK

Fiona J. L. Brown, Liaison Librarian, University of Edinburgh, Easter Bush, Midlothian, Not applicable United Kingdom

Emma Place, Medical Subject Librarian, University of Bristol, United Kingdom

Objectives: This project aims to update an open-access evidence-based veterinary medicine online tutorial, EBVM Learning, http://ebvmlearning.org. The original EBVM Learning, released

in 2015, receives more than 1000 visits per month, and is used both in universities and by veterinarians in practice.

Methods: Funded by a RCVS Knowledge Target Grant, EBVM Learning is based on a five-step description of evidence-based practice: Ask, Acquire, Appraise, Apply, and Assess. Additionally, an ABCs section provides overall context.

The revision was designed as a team exercise. Teams included project management, programming, and individual steps. Four librarians were responsible for Acquire.

Project management led the year of work. EVBM step members worked remotely in the spring, met in July 2019, and continued work remotely until the fall deadlines. EBVM Learning II is scheduled for release in late 2020.

Revision and updates were based on expertise, website feedback, surveys, and updated software capabilities. Feedback and surveys asked respondents to identity what they liked most, liked least, and solicited improvement and enhancement ideas. An article about the user feedback was published in Veterinary Evidence in February 2021.

Results: Release of the revised version was scheduled for late 2020, however, the global pandemic caused delays and it was released in spring 2021. Both the original and revised versions of EBVM Learning are available through July 2021 through the single website http://ebvmlearning.org. The two versions are simultaneously available to accommodate in-progress use and university teaching of the original version which will be archived in July. The revised version, currently available directly at http://learn.rcvsknowledge.org/ebvm-learning, takes advantage of the new Moodle-based RCVS Knowledge Learn platform. Additionally, each version is available as a pdf download.

User feedback about the original version indicated that the current format was unsuitable for busy veterinarians in practice. Accordingly, the next phase of the project will create a slimline version especially for veterinary practitioners. Currently in development, it is scheduled for release in late 2021.

Conclusions: The of EBVM Learning tutorial has increased awareness of evidence-based practice in the profession and at universities. It is helping the next generation of veterinary students and veterinarians engage in EBVM in clinical practice.

Effects of the COVID-19 Pandemic on Academic Medical Library Services

Practice Area: Information Services

Deborah H. Charbonneau, Associate Professor, Wayne State University, School of Information Sciences, Detroit, Michigan

Emily Vardell, AHIP, Assistant Professor, Emporia State University School of Library and Information Management, Emporia, Kansas

Objectives: The aim of this study was to gain a better understanding of the scope of reference services provided by academic health sciences libraries amidst the COVID-19 pandemic. The research study examines how reference services changed during the COVID-19 pandemic (March 2020-March 2021), the types of COVID-related reference questions that librarians working in academic health sciences libraries received, and challenging reference inquiries.

Methods: In March 2021, librarians working in academic health sciences libraries in the U.S. were invited to participate in an anonymous online survey about their experiences providing reference services during the COVID pandemic. The online survey was developed, pre-tested, and distributed to the MEDLIB-L and MLA Chapter listservs. One email reminder was sent, and data collection concluded after one month.

Results: A total of 205 individuals (N=205) responded to the online survey. Respondents indicated the scope of reference services provided during the COVID pandemic included email-based reference services (97.04%), virtual reference (89.35%), telephone (79.88%), text-based (32.54%), and in-person (30.77%). Librarians received COVID-19 reference questions from a variety of patrons, including faculty (75.71%), students (59.29%), researchers (50.71%), staff (47.14%), health care providers (42.14%,), university administrators (22.14%), and the general public (17.14%). The most common types of reference question topics included COVID-19 treatments (52.90%), safety precautions (45.65%), vaccines (40.58%), and prevalence (38.41%). Additionally, the identification of challenging reference questions and examples of “misinformation” were provided by respondents.

Conclusions: The results of the national survey characterize the scope of COVID-related reference services and range of questions. Given the evolving nature of the COVID pandemic, this research study sought to learn more about reference services and types of inquiries during this time of uncertainty. Librarians reported an increase in reference questions during the pandemic and are answering them in creative ways despite barriers (e.g., limited time and reduction in resources). It is also clear there is an opportunity for librarians to address COVID-related misinformation amongst a variety of patron types. Overall, these preliminary results shed light on the adaptive nature of reference services and provide useful insight for library practitioners and administrators planning reference services during public health crises. The study further demonstrates the role that academic health sciences librarians and information professionals are playing in connecting people with the latest information on this emergent topic.

Employee Wellness Concerns before and after Pandemic-Driven Changes to the Work Environment

Practice Area: Professionalism & Leadership

Terry Kit Selfe, AHIP, Translational Research and Impact Librarian, University of Florida, Gainesville, Florida

Objectives: In February 2020 our wellness committee completed a survey assessing the wellness needs of library employees. The data were meant to inform creation of employee wellness programming and serve as baseline data for evaluating the program's effectiveness. Within a month of the completion of data collection the work environment drastically changed. Most of us were no longer working onsite. In November 2020 we once again distributed the survey. The objective of the follow-up survey was to discover how wellness concerns and priorities may have shifted in light of COVID-19 and the resultant changes to the work environment.

Methods: An anonymous online survey was distributed via our library employees email list in November 2020 as a follow-up to our February 2020 survey. Topics covered in the 7-minute Qualtrics survey included the dimensions of wellness respondents would like a wellness program to address, health topics of interest, and concerns regarding wellness within the work environment. For the follow-up survey, respondents were instructed to answer the questions based on their current work environment, whether that be on-campus, working from home, or some hybrid arrangement. Aggregate responses to the two identical surveys were compared to identify any changes.

Results: Ninety-seven employees completed the February survey and 80 the follow-up in November 2020 (38% and 30% response rates, respectively). The top wellness concerns identified by respondents included morale (n=55) and mental exhaustion (n=51) in February, and mental exhaustion (n=48) and work/life balance (n=42) in November. Wellness dimensions most respondents would like a wellness program to address included physical (n=75), emotional (n=67), and occupational wellness (n=65) in February, and emotional (n=56), physical (n=54), and occupational (n=43) at follow-up. Stress management was the topic of most interest to respondents both in February (26.8%) and November (32%).

Conclusions: Our preliminary findings suggest overall library employee wellness concerns remained fairly stable, despite a major change from an onsite work environment to a predominantly work-from-home model. The top three wellness domains remained the same, although the order changed, and stress management remained the health topic of most interest. The main difference we noted was that work/life balance rose to one of the top concerns in the follow-up survey. This may reflect an increased difficulty in maintaining boundaries between work and home life when both are occurring in the same location. Going forward, our library wellness committee will prioritize content on stress management given the ongoing interest in the topic.

EndNote or DistillerSR for De-Duping References, Which to Use?

Practice Area: Innovation & Research Practice

Linda M. Hartman, MLS, AHIP, Researcn Librarian, RQM+, Glenshaw, Pennsylvania

Objectives: Because databases differ in subject matter, indexing, and holdings, more than one is used when conducting literature searches. As a result, the same reference can be captured multiple times, creating duplicate records in the retrieval. The duplicates must be removed before reference screening and data extraction. This study reviews the deduping features in the reference management software EndNote and the systematic review software tool DistillerSR. The hypothesis is that EndNote allows for more robust duplicate detection. However, the advantage to deduping in DistillerSR is that the duplicates are automatically accounted for in the PRISMA diagram that it can generate.

Methods: EndNote and DistillerSR each have default settings for duplicate detection which include the author and title fields. In each product it is possible to adjust what fields are used when detecting duplicates. Bramer et al’s 2016 JMLA article provides a method using various field combinations. Three separate searches were conducted in PubMed and Embase. The resulting references were exported to EndNote and then uploaded into DistillerSR. Deduping was done using the default settings and Bramer et al’s method in EndNote as well as in DistillerSR. While Bramer et al’s method was designed for EndNote, it is possible to use the same concept of manipulating the fields in DistillerSR.

Results: When EndNote was used the number of duplicates found using Bramer et al’s method was greater than the default. When DistillerSR was used the number of duplicates found was the same with either method. Both tools allow the user to compare the references identified as duplicates side by side. The user can also accept wholesale the duplicates identified by both programs. One difference is that DistillerSR displays a per cent confidence for each duplicate pair. The user can select the confidence threshold above which the duplicates are removed without review by the user.

Conclusions: If the researcher has access to DistillerSR it is recommended to use it for the deduplication process. There is no difference in which duplicates are found and doing it here allows the researcher to use the PRISMA diagram function. If the deduping is done in EndNote before uploading the references to DistillerSR the PRISMA diagram would not account for the duplicates. If the researcher does not have access to DistillerSR it is recommended to use Bramer et al’s method since more duplicates are found using it than with the default settings.

Examining COVID-19 Resources on Association of Academic Health Sciences Libraries (AAHSL) Websites

Practice Area: Information Management

E. Bailey Sterling, Digital Communications Specialist, Network of the National Library of Medicine, Region 3, PLANO, Texas

Ana D. Cleveland, AHIP, FMLA, Director of Health Informatics Program, University of North Texas, Denton, Texas

Jodi L. Philbrick, AHIP, Senior Lecturer, University of North Texas, Denton, Texas

Objective: 1) Examine AAHSL member library websites and their coverage of COVID-19. 2) Analyze AAHSL COVID-19 LibGuides to determine the quantity and origin of links included.

Method: Through stratified sampling based on total enrollment size of medical or health science academic institutions, 51 of 147 (34.7%) eligible AASHL library websites were selected for inclusion in the study. Data from the AAHSL library websites were captured on a spreadsheet, including COVID-19 LibGuide resources and embedded content, from April 3 - April 21, 2021. The LibGuide content was analyzed, and the origin of each link was categorized.

Results: The majority (43/51, 84.3%) of sampled AAHSL libraries have at least one COVID-19 LibGuide, with 8 out of 43 (18.6%) libraries having two or more. Also, 8 out of 51 (15.7%) libraries have no published COVID-19 LibGuide. A total of 8848 links within the examined LibGuides were visited. Links to academic institutions (2439/8848, 27.6%) including universities' own internal links were most common, while news outlets (220/8848, 2.5%), social media (169/8848, 1.9%), and international government (49/8848, 0.6%) were linked least. Regarding individual organizations, the Centers for Disease Control and Prevention (575/8848, 6.5%) and the National Institutes of Health (529/8848, 6%) were most frequently linked.

Conclusions: Over three quarters of sampled AAHSL libraries have at least one COVID-19 LibGuide. Links to university resources comprise over one quarter of COVID-19 LibGuide links. Domestic and international news sources; social media including Facebook, Twitter, and YouTube; and non-U.S. government sources were linked least.

Exploring Health Literacy Interventions and Understanding the Experience of Shared Decision-Making from the Perspective of Breast Cancer Patients

Practice Area: Clinical Support

Antonio P. DeRosa, AHIP, Program Lead, Decision Navigation & Patient Support / Oncology Consumer Health Librarian, Weill Cornell Medicine, Astoria, New York

Objectives: The goal of the proposed research is to understand the lived experiences of breast cancer patients surrounding the shared decision-making (SDM) process and to investigate areas for health literacy intervention improvements to support the decision-making practices of this patient population. Evidence suggests that cancer patients actively taking part in decision-making might have a positive impact on their quality of life and overall outcomes. Models of SDM have been tested in various healthcare settings and in different diagnoses, with favorable results reported when patients’ personal values and preferences are taken into consideration during the decision-making process.

Methods: A qualitative phenomenological research design consisting of semi-structured interviews with breast cancer patients will take place. A purposive sample of participants will be recruited through flyers and posters placed throughout the Weill Cornell Medicine Meyer Cancer Center (WCM-MCC) Breast Center. After consented, participants will be scheduled for their in-depth interviews with the researchers, which will be recorded. Recordings will be transcribed by a consultant after the interviews and significant statements will then be extracted by the researcher. The meaning of these statements will be determined based on re-reviewing of the transcripts and re-listening to the recordings. Following this re-evaluation to determine meaning of significant statements, clustering of the meanings will take place and be organized into common themes which will make up the essence of the lived experience described by participants.

Results: Anticipated results include a definition of SDM based on the qualitative analysis of interviews with breast cancer patients. Along with a definition of SDM among this patient population, results will include thematic presentation of the main concerns and experiences with SDM from the perspective of these patients. Results will also provide a deeper understanding of the health literacy knowledge and needs of breast cancer patients as they embark on making critical treatment decisions.

Conclusions: The results of this research will inform potential health literacy interventions for breast cancer patients who need to make a decision about their treatment or overall care plan. It is also the intention of the researcher to promote employing a phenomenological approach to understanding the information and decision-making needs of diverse patient populations and in various healthcare settings. Results of this research will add to the growing body of literature on supporting healthcare consumer decision-making through information and health literacy education services.

Facilitating Data Literacy: Evaluation of the Data Analytics Research Training (DART) Course

Practice Area: Education

Peace Ossom Williamson, AHIP, Director of Research Data Services, The University of Texas at Arlington, Arlington, Texas

Hammad Khan, Data Management Librarian, The University of Texas at Arlington, ,

Laura N. Haygood, AHIP, Research and Education Librarian, University of North Texas Health Science Center, Gibson D. Lewis Library, Fort Worth, Texas

Objectives: There is an urgent need to train health information professionals (HIPs) who are increasingly moving into data-related positions that support and collaborate in research; however, few informatics and data literacy trainings are available for HIPs. To address this need, we developed and assessed the Data Analytics Research Training (DART) Course on its effectiveness for HIPs working in areas with limited resources to acquire data literacy skills. The DART Course is a free, self-paced data science program delivered in online modules.

Methods: We developed a curriculum based in situated learning and cognitive apprenticeship to align with data literacy competencies and online learning principles from Quality Matters, a certification focused on quality online and hybrid education development and experiential learning. We recruited HIPs from diverse ethnic and work backgrounds who were working with underserved communities. Evaluation was based upon a logic model created from the Kirkpatrick Model. The presenters will also describe the process of facilitating the program.

Results: Currently, most DART Course participants were female (88%) with the largest proportions from the National Network of Libraries of Medicine South Central Region (37.5%) and Middle Atlantic Region (25%). Of the 24 participants, 18 worked in medical and academic libraries, three worked in health departments, and two worked in public libraries. We will discuss lessons learned from developing the program, changes made due to the COVID-19 pandemic, evaluation results, and future plans. Key lessons include the importance of building a culture of support and learning, using project management tools, and applying strategies to effectively streamline information.

Conclusions: We hypothesize that intentionally designed, competency-based data science trainings will have a significant impact by building the data literacy capacity of HIPs in underserved communities.

Health Sciences Librarians’ Engagement in Open Science: A Scoping Review

Practice Area: Information Management

Kevin Read, Associate Librarian, University of Saskatchewan, Canada

Dean Giustini, UBC Biomedical Branch Librarian, University of British Columbia, Vancouver, British Columbia, Canada

Ariel Deardorff, Data Services Librarian, UCSF Library, San Francisco, California

Lisa Federer, AHIP, NLM Data Science and Open Science Librarian, National Library of Medicine, North Bethesda, Maryland

Melissa L. Rethlefsen, AHIP, Executive Director, Health Sciences Library & Informatics Center / University of New Mexico, Albuquerque, New Mexico

Objectives: To identify health sciences librarians’ (HSLs) engagement in open science (OS) through the delivery of library services, support, and programs for researchers.

Methods: We performed a scoping review guided by Arksey and O’Malley’s framework and Joanna Briggs’ Manual for Scoping Reviews. Our search methods consisted of searching five databases (MEDLINE, Embase, CINAHL, LISTA, and Web of Science Core Collection), tracking citations, contacting experts, and targeted web searching. We used Zotero to manage citations, and Covidence for screening. To determine study eligibility, we applied predetermined inclusion and exclusion criteria, achieving consensus among reviewers when there was disagreement. Finally, we extracted data in duplicate and performed qualitative analysis to map key themes.

Results: We identified 54 included studies after reviewing 8173 citations and 319 full text studies. Research methods included descriptive or narrative approaches (76%), surveys, questionnaires and interviews (15%) or mixed methods (9%). Publication types included case studies (46%), journal articles (39%), conference posters and abstracts (9%), book chapters (4%), and reports (2%). Using FOSTER’s Open Science Taxonomy, we labeled studies with one or more of six themes: open access (54%), open data (43%), open science (24%), and open education, open source and citizen science (17%). Key drivers in OS were scientific integrity and transparency, openness as a guiding principle in research, and funder mandates making research openly-accessible. HSLs engaged in advocacy for OS in many ways, with most examples coming from academic institutions. HSLs assumed key roles by advocating for and promoting OS, and by collaborating on policy development, especially in support of OA and open data.

Conclusions: HSLs play key roles in advancing OS worldwide. However, more formal studies are needed to assess the impact of HSLs’ engagement in OS to determine best practices. Future studies should identify OS researchers’ needs, and evaluate the library service models best designed to meet them. HSLs should promote broader adoption of OS within their

research communities, and develop strategic plans aligned with institutional partners. Further, HSLs can promote OS by adopting more rigorous and transparent research practices of their own. Future research should consider examining HSLs’ engagement in OS through social justice and equity perspectives.

Health Sciences Librarians’ Engagement in Work-Related Reflection: Results of a Qualitative Exploration of Why They Engage in Reflective Practice

Practice Area: Professionalism & Leadership

Jolene M. Miller, AHIP, Director, Mulford Health Science Library, University of Toledo, Toledo, Ohio

Objectives: Published research on health science librarians use of reflection at work (reflective practice) tends to be results of action research and surveys. The current study fills a gap in the literature, exploring the following questions: As librarians engage in intentional reflection to improve work performance, what are they actually doing? Why do they invest time and energy in reflection in the first place? The purpose of this qualitative study is to better understand how health science librarians experience and understand reflective practice.

Methods: In 2020, 18 health science librarians who use reflection at work were recruited using email invitations sent to medical/health science librarianship email distribution lists based in the US and Canada. There were no limitations on how, to what extent, or how long participants had been using reflection. They were interviewed using a video conferencing platform about their use of reflection at work and what advice they would give to someone new to reflective practice. The interview transcripts were analyzed within a phenomenological framework for themes using MAXQDA 2020 software. (Prior to recruitment, this study was reviewed and identified as exempt research by the University of Toledo Social, Behavioral, and Educational Institutional Review Board.)

Results: Several themes emerged from the analyses: (1) participants reported a variety of benefits from engaging in reflection at work; (2) the importance of intentionality in reflection; (3) the role of emotion in reflective practices; and (4) the wide range of reflective strategies used by participants. The primary themes regarding advice to people new to reflection: (1) start small; and (2) find what works best for you.

Conclusions: This project provides rich detail about the ways that health science librarians engage in using reflection at work. Perhaps the most important finding is that librarians used reflection in ways that work best for them in terms of their workload, work environment, personality, and extent of experience with reflection. Hopefully, this encourages health science librarians to consider using reflection to improve their work performance without fear that they are “doing it wrong.”

Implications of COVID-19 Systematic Reviews Published as Non-Research Publications

Practice Area: Innovation & Research Practice

Kathryn Vela, AHIP, Medical Librarian, St. Luke's Health System, Boise, Idaho

Electra Enslow, Director, Clinical Research and Data Services, University of Washington Health Sciences Library, ,

Aidy Weeks, AHIP, Interim Director, UNLV Health Sciences Library, Las Vegas, Nevada

Objectives: To examine the practice of publishing systematic reviews on COVID-19-related clinical topics as non-research publications (i.e. editorials, commentaries, etc.). We will explore the prevalence of this publishing practice and its implications for clinical research and decision-making.

Methods: We searched LitCovid, Embase, and WHO databases for published studies using terms for “COVID-19” and “systematic review”, and limited our search to studies published between December 2019 and May 2020 (Search conducted on June 1st). We excluded studies that: described protocols or guidelines or consensus statements; were non-systematic reviews; addressed a non-clinical topic; or were systematic reviews published as a research paper or a preprint. Our search retrieved 305 unique results and we were left with 30 systematic reviews for analysis after title/abstract review. We also randomly selected a sample of 30 systematic reviews published as a traditional research paper from our selection of 305 results to use as a comparison set. Each review will be assessed using the PRISMA 2020 reporting guidelines and AMSTAR 2 quality criteria; each systematic review group will be compared with descriptive statistics.

Results: Using the results of the PRISMA and AMSTAR 2 assessments, we will be able to discuss the quality and utility of publications labeled as systematic reviews that may not have been subject to peer-review or the traditional systematic review framework.

Conclusions: This study will help to educate healthcare workers and clinical researchers on how to critically appraise high-level evidence; conclusions will be available for the MLA21 conference.

Machine Learning in Systematic Reviews: a Systematic Review

Practice Area: Innovation & Research Practice

Stephanie Clare Roth, AHIP, Biomedical & Research Services Librarian, Temple University, Ginsburg Health Sciences Library, Philadelphia, Pennsylvania

Alex Wermer-Colan, Digital Scholarship Coordinator, Temple Libraries, Loretta C. Duckworth Scholars Studio

Jenny Pierce, Head Research Education and Outreach, Temple University Health Sciences Libraries

Objectives: Machine learning for natural language processing (also known as text mining) has the potential to significantly reduce the labor and time involved in conducting systematic review research. Our systematic review was designed to answer the question, “What impact does the use of machine learning have on the methods used to carry out the systematic review process, such as the precision and development of search strategies, unbiased article selection, or data abstraction and analysis for systematic reviews and other comprehensive review types of similar methodology (i.e. scoping review, rapid review)?”

Methods: A comprehensive literature search was performed from 2003 to December 4, 2020 to identify all potentially relevant studies about machine learning in systematic reviews with a focus on text mining. A protocol was registered at the Open Science Framework https://osf.io/4zwmc/ and search strategies were deposited in TU ScholarShare http://hdl.handle.net/20.500.12613/4637. Studies that were not related to the systematic review or similar review types and their methods were excluded, including systematic reviews that may have used text mining or machine learning, but did not assess the value of their methods for improving their systematic review. Studies focused solely on updating an existing systematic review were also excluded. The search was developed for PubMed (NLM) and was translated to Embase (Elsevier), Cochrane Central (Wiley), Scopus (Elsevier), LISTA (EbscoHost) and the Social Science Premium Collection (Proquest). An attempt to locate grey literature was carried out using a Google search and SuRe Info. A handsearch was conducted by scanning reference lists of key journals and conference proceedings.

Results: Machine learning can be used for various stages of the systematic review and other comprehensive review types. The literature has shown evidence that machine learning can decrease the amount of time and labor it takes to complete a full systematic review. However, there are still several barriers to the successful implementation and utilization of machine learning to improve the overall systematic review process.

Conclusions: Machine learning can improve the systematic review process from start to finish by reducing both time and labor. Further studies into best practices and software is needed. Substantial work is necessary to integrate machine learning into the systematic review process

in a streamlined and user-friendly way leading to widespread adoption of machine learning for systematic reviews and other comprehensive review types.

Maximizing long-term compensation: Retirement plan options for medical librarians at public universities

Practice Area: Professionalism & Leadership

David Petersen, AHIP, Assistant Professor, Research & Learning Services Librarian, Preston Medical Library, University of Tennessee Graduate School of Medicine, Knoxville, Tennessee

Objectives: Full-time academic medical librarians at public institutions are typically provided with an employer-sponsored retirement plan, a vital benefit to ensure long-term financial stability, particularly as COVID-19 restricts salary raises. This study examines three retirement plan categories offered to librarians at state university systems: defined benefit (pension), defined contribution (ORP), and hybrid (pension/ORP components) plans. This study analyzes all currently available plans to assess whether a medical librarian can expect consistent benefits across state systems and plan categories or whether major differences exist.

Methods: States plus the District of Columbia were split into seven geographic groupings based on established NNLM Regional Medical Library regions. Researchers gathered pertinent data for every public state university system; information was only obtained for plans that a new employee could enroll in. Due to limitations, community colleges and private institutions were excluded. Because some institutions use a formula based on age and/or salary, this study uses a 35 yr. old individual earning $60,000 annually. Overall, 77 ORP plans, 32 defined benefit plans, and 9 hybrid/alternate plans were examined for employer contributions, required employee contributions, vesting periods, and other requirements. Retirement calculators were then used to project benefits.

Results: All states offer a retirement plan with 46 offering an ORP style plan, 29 providing a defined benefit or hybrid plan option, 2 states providing a defined benefit option at some universities, and 1 state providing an ORP style plan at some universities. ORP plans vary greatly from 5% - 15.25% employer contributions. Region 5 had the highest average employer contribution (10.95%), while Region 2 had the lowest (8.02%). Defined benefit uncapped multipliers ranged from 1% - 2.5%. Required employee contributions to defined benefit and hybrid plans ranged from 0% - 14%.

Conclusions: Retirement plan benefits are highly variable as to the state and/or university providing them. Some states offer generous plans, while others are far more modest. Vesting requirements for employer contributions remain an issue in several states with some plans requiring as many as 10 years of service. Six states university systems in whole, and one state in part, do not participate in FICA (Social Security), thus placing a greater weight on selected retirement plans. Prospective employees, particularly those seeking long-term positions, must be aware of available retirement plans to make an informed choice.

The Open Access Citation Advantage in the Health Sciences: Results of a Systematic Review and Meta-Analysis

Practice Area: Innovation & Research Practice

Caitlin Bakker, AHIP, Research Services Librarian, University of Minnesota, Minneapolis, Minnesota

Allison Langham-Putrow, Scholarly Communications Librarian, University of Minnesota Libraries, Minneapolis,

Amy L. Riegelman, Social Sciences Librarian, University of Minnesota Libraries, Minneapolis, Minnesota

Objective/Research Question: Open access publishing is a well-established, yet often debated, channel in the health sciences. Researchers selecting a journal may consider the quality and prestige of the journal, its readership and the intended audience of the paper, and the potential to maximize research impact. It’s been argued that choosing to publish open access could lead to higher impact, but is that the case? And how would that compare to other disciplines? In this follow-up presentation to our preliminary findings at last year’s MLA, we explore the impact of open access on citations in the health sciences.

Methods: We conducted a systematic review focusing on items reporting the impact of open access status on citation of articles. We executed this search across seventeen databases representing a broad range of disciplines. Title and abstract screening, full-text screening, data extraction and risk of bias assessment were independently completed by two reviewers. We extracted data on the disciplines and journals included, and mapped these data points to subject areas to create disciplinary groupings. We also extracted data on sources of information, sample size, and study design.

Results: After screening 4,019 items, we included 134 studies. The majority of these were focused on gold OA (53/134, 39.6%), measured citations in means (88/134, 65.7%), and used Web of Science as a data source (82/134, 61.2%). There were no statistically significant relationships between the findings and OA modes included ($p = 1.61$), metrics used ($p = .861$), or data source ($p = .663$). Of the 134 studies, 36 were focused on the health sciences. 19 of those found an OACA, while 14 did not and 3 found an OACA only in subsets of articles.

Discussion: While the majority of studies found a positive relationship between citation counts and OA status, appraisal and synthesis of this literature is challenged by incomplete reporting, potential methodological biases, and a wide variety of existing and emerging metrics and definitions of OA. The use of reporting guidelines to increase the transparency and clarity of bibliometrics studies would improve the state of the field while allowing for more conclusive answers.

Organizational Culture and the Implementation of Health Information Technology: A Scoping Review

Practice Area: Innovation & Research Practice

Caitlin Bakker, AHIP, Research Services Librarian, University of Minnesota, Minneapolis, Minnesota

Gretchen Hultman

Sripriya Rajamani

Genevieve Melton-Meaux

Objectives: Despite growing use of health information technology (HIT) across healthcare settings, and the increasing amount of time and resources spent, many HIT implementations are not successful. There can be a great value-add in facilitating the assessment of contextual factors related to implementation of HIT. Health information professionals are well-positioned to contribute to this assessment, as they are engaged in managing access to and promoting use of knowledge resources and systems. This scoping review examined the role of organizational culture in HIT implementations. This presentation will describe the results of this review and will provide practical strategies for information professionals.

Methods: We conducted a comprehensive search of 13 databases using controlled vocabulary and natural language searching. We focused on the HIT implementation studies in the last 10 years in the US. The review was guided by the Consolidated Framework for Implementation Research (CFIR), a state-of-the-science framework in implementation research. Culture related definitions and constructs mentioned in CFIR were used to develop inclusion and exclusion criteria. Guided by Arskey and O’Malley’s five-step framework, two independent researchers screened each title and abstract, and this process was followed with full-text screening using previously established criteria. Data extraction forms were developed by one researcher prior to piloting by the group for further refinement. We extracted data points on the study setting, study design, type of HIT implemented, and references to culture.

Results: We screened 2,178 records, ultimately reviewing 333 articles in full text and including 55 articles representing 52 studies. Data in these studies were most frequently gathered through interviews and focused on the implementation or evaluation phases of HIT projects. Themes of leadership engagement and resource availability were more prominent, while employee workload, stress and motivation were less frequently discussed.

Conclusions: Librarians and information professionals are involved in the implementation and maintenance of HIT. Understanding of the role of organizational culture in these implementations can assist librarians as both expert users and team members.

Paths to Systematic Review Librarianship: An Exploratory Study

Practice Area: Information Services

Jeremy J. Tietgen, Librarian, Lennox School District, Sioux Falls, South Dakota

Emily Vardell, AHIP, Assistant Professor, Emporia State University School of Library and Information Management, Emporia, Kansas

Objectives: This study seeks to fill the gap in the literature regarding how academic librarians are preparing for their roles as systematic review librarians (SRLs). Data was gathered to explore common themes amongst current systematic review librarians. This is a crucial time for academic librarians to demonstrate the capacity to be a part of and/or lead systematic reviews (SR).

Methods: Fourteen Zoom interviews were conducted with academic health librarians who perform systematic reviews. The semi-structured interview questions were designed to gather information about the participants' paths from undergraduate degree to their current positions. The questions centered around education and employment history, professional development, professional affiliations, number of systematic reviews authored, and recommendations for those who are thinking of becoming SRLs. The authors performed a thematic analysis of the data to identify how SRLs prepared to serve in their roles.

Results: This presentation will focus on a few of the most relevant and salient codes of the total 41 thematic codes. Of the 14 participants, it was clear there is no one straight path to the role of SRL. All educational backgrounds, employment history, and “paths” to performing SRs at their institution were vastly different among the interviewees. Two common themes that were discovered were the specific SRL training attended by the participants and all the workshops attended were paid for by their institutions. Thirteen of the 14 participants had taken at least one or more of the following courses: University of Michigan Systematic Review Workshop, University of Pittsburgh Systematic Review Workshop, and the University of Colorado Denver Anschutz’s Evidence Based Medicine course.

Conclusions: The data collected will provide valuable information for current librarians, future librarians, library administrators, academic and professional institutions, and library science graduate programs. The results of the study should provide answers to what is currently working for today’s SRLs. By understanding the paths taken by those SRLs, individual librarians, administrators, institutions, and future librarians can make informed decisions. This study confirms there is currently no one standard path to becoming an SRL. This study only involved medical SRL and does not speak to librarians performing SRs outside of the health sciences. The data implies a need for future research to better clarify if there is a need for a path which may include specific educational and employment training.

Patient Value Learning: Using Market Research and Qualitative Research Techniques to Understand Patient Needs

Practice Area: Innovation & Research Practice

James Michael Lindsay, AHIP, Head of Collections & Access Services, Preston Medical Library / University of Tennessee Graduate School of Medicine, Knoxville, Tennessee

David Petersen, AHIP, Assistant Professor, Research & Learning Services Librarian, Preston Medical Library, University of Tennessee Graduate School of Medicine, Knoxville, Tennessee

Kelsey L. Grabeel, AHIP, Assistant Director, Preston Medical Library / Health Information Center, University of Tennessee Graduate School of Medicine / University of Tennessee Medical Center, Knoxville, Tennessee

Alexandria Q. Wilson, Assistant Professor/Research & Learning Services Librarian, Preston Medical Library, University of Tennessee Graduate School of Medicine, Knoxville, Tennessee

Martha Earl, AHIP, Director/Associate Professor, Preston Medical Library, Tennessee

Objectives: To transform our diversifying communities, librarians must understand the goals and values of our patrons. Librarians can do this by drawing upon and using knowledge from a variety of disciplines. In the discipline of Marketing, market researchers strive to learn what is important to their customers. Librarians at Preston Medical Library engaged in a qualitative research study using market research techniques to learn the goals of patrons in using our service.

Methods: This qualitative research study draws upon Customer Value Learning, a branch of marketing research designed to elicit the desired end states and goals that customers or patrons have in using our service. We initially interviewed two patrons of the library’s consumer health information service, conducting 45-minute laddering interviews. With permission, these interviews are recorded, and the recordings are used to develop customer value hierarchy diagrams.

Following these interviews, the library began a qualitative research project using these methods. Consulting with the UT Graduate School of Medicine’s qualitative research expert, the library developed an interview guide for further research, drew up a list of the most consistent users of the service, drafted an invitation letter to these potential participants, applied for IRB approval of the project, and trained interviewers.

Results: In our initial results, the library interviewed two patrons using the laddering technique, beginning with attributes of the service, progressing to the patron’s interactions with the service, and finally reaching their desired end states or goals for using the service. Following these interviews, customer value hierarchies were constructed, illustrating graphically how the

patron interacted with the service. The results of a full study, involving at least 10 additional participants, will be reported at the time of the conference.

Conclusions: Insights from the initial reviews have convinced the library of the value of this technique, and findings from the full study will be reported on at the conference, then integrated into the library’s marketing and promotion efforts as well as satisfaction measurement efforts.

Predatory Journals in PubMed Central: A Comprehensive Analysis

Practice Area: Innovation & Research Practice

Jason Burton, Science Collections Librarian, University of California, Los Angeles, OXFORD, Mississippi

Objectives: Predatory publishing has been a long-standing problem across scholarly publishing for more than a decade. More recently concerned has been raised about the presence of predatory titles in PubMed Central and by extension PubMed. Research has been done to, in part, identify the extent of predatory publishing in PubMed in a disciplinary context, specifically rehabilitation, neuroscience, and neurology. This study looked to quantify the problem of predatory publishing in PubMed Central by asking a simple question, how many articles from predatory journals appear in PubMed Central?

Methods: Individual journals and publishers were identified using Beall's List of predatory journals and publishers. Journal titles were pulled out for each publisher listed. Publisher websites that were no longer online were retrieved using the Internet Archive’s Wayback Machine and journal titles were pulled from the highest journal output for the publisher. Titles appearing on the individual journal list were deduplicated from journals pulled from the publisher’s list. Journal titles were then searched in PubMed Central with the following information collected: availability in PubMed Central, method for entry into PubMed Central, number of articles, publication date, and author manuscript funding agency.

Results: A total of 27,147 total journals were identified from Beall’s List of journals and publishers. 1,147 had articles submitted to PubMed Central by individual authors and 150 were PubMed Central titles for a total of 1,298 archived in PubMed Central. These journals made 270,642 articles available in PubMed Central; 4,280 articles were the result of direct submissions and 266,362 appeared in PubMed Central titles. Calculating from the number of articles archived in PubMed Central (6,200,000) at the time of analysis, June 2020, 4.3% were from predatory journals.

Conclusions: This analysis points to a small, but still significant presence of content from predatory journals in PubMed Central. The scale of the problem, like all discussions of predatory publishing, hinges on defining what is and is not predatory. Lists of predatory journals and publishers, like Beall’s, and PubMed Central will not always agree on an

individual title. The current disagreement makes up more than 98% of the predatory article content in PubMed Central. Further work is needed to provide authors and readers the tools to assess publisher output.

The Publication Fate of Abstracts Presented at the Medical Library Association's Annual Meetings

Practice Area: Innovation & Research Practice

Part of the Research Training Institute Ignite Session

Rachel Hinrichs, AHIP, Health Sciences Librarian, IUPUI, Indianapolis, Indiana

Mirian Ramirez, Research Metrics Librarian, Ruth Lilly Medical Library, Indianapolis, Indiana

Mahasin S. Ameen, Teaching and Learning Librarian, IUPUI University Library, Indianapolis, Indiana

A Self-Paced Online Library Orientation: Development, Implementation, and Assessment

Practice Area: Education

Hannah Schilperoort, Information Services Librarian, Norris Medical Library, University of Southern California, Los Angeles, California

Amy J. Chatfield, Information Services Librarian, Norris Medical Library, Los Angeles, California

Jennifer E. Dinalo, Clinical Services Librarian, USC Norris Medical Library, Los Angeles, California

Holly Thompson, Acting Head, Wilson Dental Library, Los Angeles, California

Karin Saric, Information Services Librarian, Norris Library, California

Objectives: Health sciences librarians may find it difficult to meet demands for in-person or online synchronous library orientations for various reasons, including lack of staff, expansion of online programs, and temporary campus closures. A review of the literature revealed that online asynchronous instruction is as effective as in-person or online synchronous instruction, so we aimed to determine if a self-paced, interactive, asynchronous, online orientation would be as effective. We created an orientation tutorial for use across various health sciences programs, used it with multiple programs, and assessed the effectiveness of the tutorial.

Methods: We created a master tutorial to be copied and altered by each librarian for the needs of their programs. We chose LibWizard to create the tutorial because it is readily available through Springshare and has many features, including side-by-side layout, variety of question types, instant feedback, live website and database interaction, certificate of completion, and built-in reporting. Each tutorial includes activities and program-specific questions throughout, a pre- and post-evaluation asking students to rate confidence using the library catalog, searching PubMed, and asking a librarian for help, as well as an open-ended question for comments and suggestions. To assess tutorial effectiveness, we used LibWizard reports to analyze data related to pre- and post-confidence ratings and user comments. We hypothesized that most users would express increased confidence in using the library catalog, searching PubMed, and asking for help after completing the tutorial.

Results: Participants’ pre and post confidence ratings and comments indicated that the tutorial was effective for student learning. Overall, participants' comfort level using the library catalog to locate resources, searching PubMed, and asking a librarian for help increased after completing the tutorial. Participant comments were overwhelmingly positive.

Conclusions: Self-paced, interactive tutorials can be effective for replacing or supplementing librarian led in-person or synchronous online library orientations. Overall, LibWizard is a great tool for creating interactive tutorials for library and information literacy instruction. It does have some limitations, but if you want students to be able to interact with live sites, databases, and library catalogs, LibWizard is an excellent choice.

Structural Models and Emerging Priorities in Academic Health Sciences Libraries

Practice Area: Professionalism & Leadership

Oya Y. Rieger, Senior Strategist, Ithaka S+R, New York,

Objectives: 1) Understanding the potential impact of mergers and other organizational changes on the academic health sciences library services, staff, and user communities.

2) Discussing the priority service areas of academic health science libraries, including research support, data management, bioinformatics, systematic reviews, assessment of research impact, and community outreach.

3) Considering community building and engagement requirements as libraries try to strike a balance between maintaining cohesiveness as a library system and being visible attending to the needs of specific user communities.

Methods: We initiated the study with a literature review and an analysis of organizational trends in order to understand how academic health sciences libraries are structured from the leadership, program, financial, IT, and HR perspectives in relation to the university libraries. Based on a semi-structured interview guide, we conducted interviews with 36 individuals

(mainly academic health sciences library leaders) from 28 libraries. Most of the interviewees have been in the field for several years and many have held other positions in libraries with different organizational configurations so their remarks were informed by their overall experiences, and were not limited to their current role.

Results: Regardless of organizational configuration, there is tremendous pressure on academic health sciences libraries to do more with less–save money, be efficient, and keep up with emerging or expanding service areas such as research data and bioinformatics support. Whether the preference is for administrative affiliation with the main library or the academic side, the interviewees stressed the situated nature of the arrangement and factors such as the styles and attitudes of library leaders.

Conclusions: Academic health sciences libraries (AHSLs) are mission-driven organizations that thrive by aligning their programs and services with the new and emerging needs of the programs they serve. Their roles are expanding, particularly in the areas of research support, data management, bioinformatics, systematic reviews, assessment of research impact, and community outreach. Because AHSLs serve a myriad of campus constituents including health care service providers, the directors interviewed made a case for having sufficient autonomy to harmonize services and procedures based on their user communities. It is important to establish and maintain close ties with diverse user communities in order to be effective, responsive, and visible.

Systematic Review Improvement through Librarian-Led Innovation

Practice Area: Innovation & Research Practice

Rebecca Carlson, MLS, AHIP, Liaison to the School of Pharmacy, University of North Carolina-Chapel Hill, Chapel Hill, North Carolina

Emily P. Jones, AHIP, Health Sciences Librarian, University of North Carolina at Chapel Hill, Health Sciences Library, Chapel Hill, North Carolina

Michelle Cawley, Head of Clinical, Academic, and Research Engagement, UNC Chapel Hill Health Sciences Library, Chapel Hill, North Carolina

Nandita S. Mani, AHIP, Associate University Librarian for Health Sciences & Director, Health Sciences Library, University Libraries, Chapel hill, North Carolina

Objectives: Systematic reviews (SRs) are an increasingly popular research methodology. New methods are emerging to improve the efficiency of SRs and thus new roles are surfacing for librarians to lead innovation in review-based research. This paper will explore new scholarship on SR innovations, including improving education on review methods, advising review teams, creating and testing emerging tools and approaches, and the crowdsourcing of review expertise.

Methods: The authors will synthesize, compare, and contrast existing literature on five aspects of systematic reviews (SRs) following searches of biomedical literature databases.

Results: This paper will include a descriptive analysis of the cost-effectiveness and time investment of SRs, the range in quality of many published reviews, emerging automation tools for reviews, and the importance of expert searcher involvement for improved quality and efficiency. Additionally, a description of future research opportunities and potential roles for librarians as systematic review innovators will be discussed.

Conclusions: Insights on how librarians can lead innovations to improve systematic reviews will be useful to all information professionals as they collaborate and educate on SR research methods.

Transforming Interim Leadership in Health Sciences Libraries: Barriers and Facilitators

Practice Area: Professionalism & Leadership

Roy E. Brown, AHIP, Research and Education Librarian, Health Sciences Library, VCU Libraries / Research and Education Department, Richmond, Virginia

John W. Cyrus, Research and Education Librarian, Health Sciences Library / Research and Education, Richmond, Virginia

Emily J. Hurst, Interim Director and Associate Dean, VCU Libraries, Health Sciences Library, Richmond, Virginia

Objectives: Interim leadership poses a number of challenges both to the individual placed in the role as well as the organization. While common practice at many organizations, interim leadership in libraries is not well documented and structured research is lacking. This research aims to inform health sciences librarians entering interim roles and administrators laying the groundwork for interim leadership at their library by identifying the barriers and facilitators faced with these roles. Surfacing these aspects of interim leadership positions may help individuals and organizations make better-informed decisions about how to prepare for interim leadership.

Methods: In order to understand the barriers and facilitators experienced by those in interim leadership roles, the researchers surveyed health sciences librarians who have served in such a role between 2015 - 2020. The survey consists of demographic questions as well as structured and open-ended questions across six domains: conditions of the interim role, position responsibilities, organizational and individual support systems, position expectations, personal motivation, and group and personal dynamics. The instrument was distributed across health sciences listservs including Medlib-L, AAHSL, and advertised via social media. Data was collected using the QuestionPro online survey tool. We will share early descriptive

analyses of the results, including a cross-tabulation of scale responses by demographic groups.

Results: Analysis of survey results will be prepared and provided prior to the presentation.

Conclusions: Conclusions will be drawn from data analysis.

Understanding the Faculty Experience: Findings From Focus Groups about Librarian Research Consultations

Practice Area: Innovation & Research Practice

John W. Cyrus, Research and Education Librarian, Health Sciences Library / Research and Education, Richmond, Virginia

Rachel Amelia Santose Koenig, Research and Education Librarian, Virginia Commonwealth University, Richmond, Virginia

Objectives: Research consultations are a standard service in academic libraries consuming significant library resources. However, there is a lack of scholarship evaluating this essential service. This presentation supplements survey data shared at MLA 2020 and provides additional insight into faculty perspectives and experiences with librarian research consultations, especially concerning the impact of research consultations on faculty productivity and the role of consultations in the research process.

Methods: Two researchers hosted three focus groups with faculty from the schools of Dentistry, Medicine, Nursing, Pharmacy, and the College of Health Professions as follow up to a survey about the usefulness of research consultations with librarians. Focus group participants were asked to comment on ways to improve the consultation service, discuss any “successful” or “positive” experiences, and describe the ways in which the consultation influenced either the faculty research process or inclination to refer others to librarians. Sessions were hosted via Zoom and were recorded. The audio recordings from each focus group were transcribed using the Zoom captioning service. The researchers used a grounded theory approach to develop themes and subthemes. All analysis was completed in HyperResearch qualitative data analysis software.

Results: A total of 12 faculty participated in three focus groups. Faculty identified six consultation-related themes, including (1) the role of the librarian, (2) the value of consulting with a librarian, (3) the librarian as expert, (4) the barriers and facilitators of consulting with a librarian, (5) the awareness of consultation services, and (6) special considerations for faculty populations.

Conclusions: Focus group findings document the ways in which librarians participate in complex research interactions with faculty. Faculty described multifaceted roles of librarians as ranging from instructor to project manager or from facilitator to collaborator. Findings from the

focus groups will inform the liaison program in a couple of ways. First, through the development of a New Faculty Toolkit, which will help outreach to new career faculty as well as those new to the institution. These findings will also help liaisons understand that clinical faculty and research faculty have different needs when it comes to research consultations with librarians. Finally, faculty descriptions of the value of research consultations and the expertise of librarians will help librarians communicate more effectively with our users.

PAPERS: PROGRAM DESCRIPTION ABSTRACTS

Papers in this section are sorted by title in alphabetical order.

Accessibility for Online Conferences: Developing Best Practices in Socially Distanced Times and Beyond

Practice Area: Professionalism & Leadership

Daina Dickman, AHIP, Scholarly Communication Librarian, California State University, Sacramento, Sacramento, California

Hannah Pollard, MLIS, AHIP, Health Sciences Librarian, Pacific Northwest University of Health Sciences, Washington

Background: During the COVID-19 pandemic many conferences moved online. Virtual conferences can open participation to those not able to attend in-person due to financial and logistics constraints. However, there is the potential to unintentionally exclude participants who rely on adaptive technologies and accessibility modifications. When moving conferences online, it is important to integrate best practices for accessibility into conference planning to promote medical librarian values of inclusion. This session is intended for conferences organized by volunteers doing planning in addition to typical job duties, rather than conferences using professional conference services, but key takeaways will be applicable to various online programs.

Description: Following experiences of planning the Mountain Pacific Health Science Libraries conference, the authors aim to present an easily digestible and replicable set of best practices that organizers can use while planning online conferences. Creating an inclusive conference was a priority from the beginning, and planning and budgetary considerations included audio, visual materials, and anticipating the needs of participants.

Audio accessibility options consisted of captioning and transcripts, with focus given to the benefits of a trained live captioner rather than computer generated captions. Materials such as handouts and slides were remediated using Section 508 guidelines, converting documents into

accessible resources. These materials were made available outside of screen share for participants who use screen readers. Care was also taken to make sure that inclusive language was used when asking participants about accessibility needs, and that participants were informed of available accessibility options.

Conclusion: After the COVID-19 pandemic ends, the benefits of virtual conferences may encourage organizations to stay virtual or add a virtual component. It is necessary to establish best practices for online conference accessibility. More than that, it is important to create a wider culture of accessibility where all organizers feel responsible for health sciences librarianship values of diversity and inclusion enacted in making conferences as accessible as possible.

The authors plan to create a follow-up survey that will measure the success of the accessibility options for the Mountain Pacific Health Science Libraries conference. After analyzing the survey data and gathering feedback from a broader population of users of adaptive technology, an OA toolkit may be created to guide future conference organizers.

An interprofessional approach to scholarly communication

Practice Area: Professionalism & Leadership

Sinead M. O'Rourke, Content Development Specialist, Weill Cornell Medicine - Qatar, Doha, Ad Dawhah 12:00:00 AM

Jamie M. Gray, Director, Distributed eLibrary, Weill Cornell Medicine - Qatar, ,

Hidenori Miyagawa, Visual Design Specialist, Weill Cornell Medicine - Qatar, Doha,   Qatar

Background: Interprofessionalism is now regarded as a necessity in clinical practice. It is increasingly demonstrated in academic settings through interprofessional education and interdepartmental collaboration, as well as academic/industry partnerships. To date, however, literature related to interprofessionalism within the medical library context has often had a focus on enhancing the contributions of librarians beyond the library setting versus exploring the potential of bringing novel skillsets into the library team. As a step towards a more intentional interprofessional approach, our medical library sought to expand into areas such as scientific and visual communication in response to a growing need within our community.

Description: During 2019, the library recruited two new roles—the Content Development Specialist and Visual Design Specialist. The goal of these roles was to establish a formal publication service that combined the traditional, librarian-led scholarly communication function with expanded offerings in editing and visual design. A scholarly communication position was already established, so individuals with specific qualifications and experience in science communication and design were recruited. To date, this service has offered manuscript editing, consultations on design-focused scholarly communication (e.g. visual abstracts), as well as

specialty training (e.g. plain language writing) both through library teaching and through the continuing professional development department. From an internal library perspective, the new roles have been involved in the redesign of our library guides, and have enhanced event planning, elective curricular content, our social media presence, and the library’s story and editorial guidelines development.

Conclusion: Since we educated faculty and staff about the new roles and service offerings, there has been increased demand for editing services, design consultations, and teaching on related topics. These roles have brought non-library-expert perspectives to the team, providing a level of real-time feedback that reflects the experiences of our constituents. In the future, medical libraries would benefit from diversifying their teams to meet the evolving needs of our users and the information landscape at large by strengthening and enhancing the scope and value of our services. One proven approach for such diversification is to support enhanced scholarly communication activities within an institution by recruiting staff with specific competencies in science communication, both written or visual, or similar backgrounds.

Are You on Board with Virtual Onboarding?: Transforming New Employee Training

Practice Area: Education

Stacy Torian, Health Sciences Librarian, University of North Carolina at Chapel Hill, Health Sciences Library, Chapel Hill, North Carolina

Rebecca Carlson, MLS, AHIP, Liaison to the School of Pharmacy, University of North Carolina-Chapel Hill, Chapel Hill, North Carolina

Emily P. Jones, AHIP, Health Sciences Librarian, University of North Carolina at Chapel Hill, Health Sciences Library, Chapel Hill, North Carolina

Michelle Cawley, Head of Clinical, Academic, and Research Engagement, UNC Chapel Hill Health Sciences Library, Chapel Hill, North Carolina

Background: Virtual onboarding can be more difficult than in-person onboarding. It can impact how new employees connect with their institution and understand their roles, as well as how the institution supports these employees. The Health Sciences Library at the University of North Carolina at Chapel Hill added two new librarians in 2020, while all librarians were working remotely due to the COVID-19 pandemic. This paper describes the challenges and opportunities of virtual onboarding generally, and for librarians specifically, while offering our perspective on how to improve virtual onboarding.

Description: The library adapted the onboarding checklist for new librarians in 2020 to ensure that all relevant training areas, including search skills training, were covered while staff were working remotely. In their first few months on the team, the librarians learned about library functional areas (through one-on-one meetings with colleagues), practiced literature searching,

and shadowed research consultations and instruction. Each librarian had weekly meetings with the department head and separate weekly meetings with an “onboarding buddy,” an experienced librarian with whom they discussed orientation activities, training experiences, library culture, and work expectations. New librarians were encouraged to provide feedback on the content and pace of the onboarding activities during each step of the process.

Results: After completing the virtual onboarding program, new librarians should be able to conduct expert searches in multiple health sciences databases, participate in research consultations with students and faculty, and lead instructional sessions for library patrons. They should also have a strong understanding of library policies and priorities, as well as the ability to communicate those policies and priorities to library and university stakeholders. Ideally, they will experience a sense of connectedness to the library and camaraderie with their colleagues within the virtual environment.

Conclusions: Two librarians were hired in 2020. One was new to librarianship; the other had worked as a health sciences librarian for three years. The experienced librarian concluded that having a library background made the virtual onboarding process go more smoothly for her. The newcomer to the field acknowledged this, while noting that virtual onboarding can offer unique advantages, such as fewer distractions. While both librarians completed their onboarding successfully, pandemic-related restrictions placed substantial limits on in-person communication and training in clinical settings. In-person communication is critical during the onboarding process, as is regular interaction with other campus libraries and departments. Efforts to enhance internal and cross-departmental communication will continue once all staff members are back on site.

Book Clubs: Vehicles for Starting Health Conversations

Practice Area: Clinical Support

Darlene Kaskie, Community Engagement Coordinator, Network of the National Library of Medicine, Iowa City, Iowa

Michele Spatz, NNLM Community Engagement Coordinator, NNLM, Seattle, Washington

George G. Strawley, Engagement Coordinator, National Network of Libraries of Medicine, Salt Lake City, Utah

Background: This session will feature a presentation by the team behind a national campaign to encourage reading and discussion of books on health-related topics. Rather than focusing on imparting predesignated facts or engaging in the kinds of interventions of other public health campaigns, the program aimed to get public library patrons, especially those in underserved communities, thinking and talking about health issues with the aid of reliable information. Organizers created ready-to-use health literacy toolkits to help libraries support health information needs in their communities through book discussion groups.

Description: The team selected three book titles each month across a variety of genres: fiction, nonfiction, memoir or graphic medicine. The project emphasized subjects of interest to communities underserved in healthcare and health information. Topics included healthy aging, heart health, racism and health, LQBTQ health, substance abuse and several others.

The team shipped free book kits to eligible libraries upon request, accompanying them with discussion guides, promotional materials and evidence-based health information. The collateral materials also became available for download from the project’s website. The project shipped more than 730 free, ready-to-use book kits over 17 months.

After shipping operations shut down because of COVID-19 concerns, organizers pivoted from concentrating on print book kits to facilitating eBook club discussions and virtual events. The team is now offering featured online author talks to keep selected health topics in front of library patrons.

Conclusion: The campaign represents an innovation to one of librarianship’s most popular and time-honored programs, the book club. Through the fun and intimacy of book discussions, readers can widen their perspectives on health and wellness topics while also receiving quality health information to make informed decisions about their health or that of a loved one. The approach is well-suited to partnerships between health sciences and public libraries that focus on community engagement. The presenters will share evaluation metrics from the program, including topics and titles most frequently selected by libraries.

Breaking It All Down: Teaching American Medical Association (AMA) Style Citations to Pre-Pharmacy Students

Practice Area: Education

Brittany R. Heer, Health Sciences Librarian, Butler University Libraries, Indianapolis, Indiana

Background: In their required fall semester course, “Exploring Pharmacy," first year pre-pharmacy students were assigned a group project to explore different aspects of the opioid crisis through the creation of an infographic and PowerPoint presentation. The instructors of this course were frustrated with the quality of the AMA style citations in both the final infographics and the PowerPoint presentations from previous years and sought an innovative pedagogical approach to teaching AMA style with the support of the health sciences librarian.

Description: In the summer of 2020, the librarian was invited by the instructors of “Exploring Pharmacy” to introduce pre-pharmacy students to basic information literacy concepts, plagiarism, and how to create AMA style citations in order to support their group projects. The librarian successfully lobbied to spread out the content into two 75-minute class periods: the first would cover information literacy concepts, and the second would cover plagiarism and AMA style citations. The design of the citations class focused on the importance of citing

sources, breaking down the essential components of an AMA citation using pharmacy examples from the library’s catalog, and having the students collaboratively work on a short in-class activity that scaffolded the content with each successive question. Formative feedback was gathered using Poll Everywhere throughout the class.

Conclusion: Initial verbal feedback from the instructors was positive regarding the new pedagogical approach to AMA style citation instruction. As the group projects were being presented and graded later in the semester, one instructor highlighted that the citations have improved significantly from previous years. Based on the student evaluations of the session, the pre-pharmacy students also appreciated the opportunity and the attention given to citations in a low-stakes environment. However, even after a 75-minute class period, students indicated that they would like more time and opportunities to practice creating and editing citations to feel confident in their abilities.

Bring Your Own Story: A Library Program to Celebrate Diverse Voices

Practice Area: Professionalism & Leadership

Shalu Gillum, AHIP, Head of Public Services, University of Central Florida College of Medicine, Orlando, Florida

Natasha Williams, AHIP, User Services Librarian, University of Central Florida College of Medicine, Orlando, Florida

Background: As part of its existing monthly lunch and learn series “Bring Your Own Lunch”, the library created a session called “Bring Your Own Story” to showcase the diverse stories and unique experiences of faculty and staff, and to promote a greater sense of community within the College of Medicine.

Description: Faculty and staff volunteers were solicited via email and word of mouth. Speakers were asked to create a 12–15-minute presentation and were encouraged to incorporate slides, photographs, or other objects to enhance the session.

The first hour-long session featured three speakers who shared their wide-ranging narratives, from converting to minimalism, to life as a White American female in South Africa, and a Bengali physician who combined a medical career with one in the arts. Subsequently, the Office of Diversity and Inclusion asked to collaborate with the library to combine this initiative with the college’s new “Human Library Project.” This new event was scheduled for the college’s Diversity Week activities. Combining these two programs resulted in a wider audience, and the inclusion of medical students. The final product was a Library Guide where these and future narratives could be archived.

Conclusion: In the future, the Health Sciences Library would like to hold Bring Your Own Story events more frequently. Following the Diversity Week activity, it was decided that

decoupling these sessions from the library’s regular lunch and learn schedule would keep the momentum for the Human Library Project and its Library Guide going and give Bring Your Own Story its own identity. The library will continue to partner with the Office of Diversity and Inclusion to host these events on a quarterly basis.

Challenge Accepted: Teaching a Four-Week Research Rotation for Third-Year Medical Students

Practice Area: Education

Molly Montgomery, MLS, MS, Director of Library Services, Idaho College of Osteopathic Medicine, Meridian, Idaho

Todd Coffey, Chair and Associate Professor, Research and Biostatistics

Background: When the new Idaho College of Osteopathic Medicine developed its curriculum in 2017, it included a mandatory one-month scholarly activity rotation during students’ 3rd-year clinical clerkships. The intended purpose of the rotation is to familiarize students with the research process and to give them a chance to start a project of their own. The 3rd year students are spread around the country, so this course is taught online and is mostly asynchronous.

Description: Responsibility for developing and teaching the research rotation fell to the Chair of Research and Biostatistics and the Director of Library Services. The primary challenge was deciding how to distill the research process into just four weeks, especially since the students have had limited exposure to literature searching, research methodology, biostatistics, and scholarly communication skills up to this point. We decided to build the rotation around the goal of preparing the students to have a small-scale research project ready once they enter their residency programs. Assignments build upon each other starting with topic choice and a literature review and moving on to filling out an IRB application form and submitting a detailed research protocol. The final projects include a research manuscript or a case report, a research poster, and a 3-minute lightning talk.

Conclusion: The first session of the research rotation started in July 2020 and 149 students will go through one of 11 sessions by June 2021. We hope to find that by going through this research rotation, students are less intimidated by the idea of engaging in research and are more likely to pursue scholarly activity projects in the future.

Changing Faculty Opinions about Information Literacy Instruction: Successful Communication Strategies

Practice Area: Education

Amy J. Chatfield, Information Services Librarian, Norris Medical Library, Los Angeles, California

Background: Librarians are frequently asked to provide a single lecture at the beginning of a course to explain all information resources students will be expected to use throughout the semester. This approach is incongruent with educational theories about adult learning, and results in bored attendees, multiple individual questions appearing at reference services, and lack of use of appropriate resources in course assignments. After facing this scenario for several years, I decided to try to change faculty’s minds to accept different instruction patterns and methods. It was then necessary to obtain faculty buy-in to integrate this new instructional style into the curriculum.

Description: In early 2017, I designed optimal library instruction sequencing that could be adapted to many courses, meet faculty preferences, and support student learning: micro lectures of no more than 20 minutes would be presented near the beginning of a course and 1 to 5 other instructional activities would be integrated at the time of need. I used rhetorical strategies, communications theories, illustrations of student work and faculty work and their relationships to educational theories, and persistence to communicate the benefits of this optimal sequencing. In fall 2020, faculty leading two long-standing courses previously requesting a single lecture requested a micro lecture and additional instructional activities, and a course scheduled for spring 2021 has also indicated interest. All of these efforts can be adapted by other librarians to their situations when needing to change faculty minds about education and instruction.

Conclusion: Changing beliefs about instructional sequencing, depth, patterns, and methods is difficult but not impossible. With practice, anyone can adapt these communications and rhetorical strategies to change attitudes about instruction.

Co-Instructing in a Course on Rigor, Reproducibility, and Transparency

Practice Area: Education

Nina Exner, Ph.D., Research Data Librarian, Virginia Commonwealth University, Richmond, Virginia

Background: The presenter will discuss their librarian role in a team-developed credit course on rigor, reproducibility, and transparency. Rigor and reproducibility is emphasized by the NIH. Recently, that emphasis has included a scorable requirement to address formal instruction in

rigorous experimental design and transparency to enhance reproducibility. This requirement applies to institutional training and career development grants, among other NIH grant types. The requirement affects campus’ success in getting these institution-level T and K grants. Therefore, a team led by our School of Medicine formed to pilot a course for doctoral students on transparency, rigor, and reproducibility.

Description: The course was designed for medical and health sciences doctoral students, particularly MD/PhD students. The design team included faculty in medicine, pharmacy, and biostatistics, the research integrity officer, and the research data librarian. As a team, we developed learning outcomes. Then we divided up the content into modules. We used a hybrid synchronous-asynchronous design, with activities to do before classes followed by synchronous discussions. The librarian focused on modules on data recording and analysis, and data reporting and transparency. Evaluations averaged above-neutral on the rating: “I am better able to list basic strategies to increase the openness and reproducibility for the products of research.” Student answers in activities showed a modest increased understanding of reproducibility requirements in their field’s journals, and an appreciation of ways to improve results reporting to ensure reproducible science.

Conclusion: The pilot course was a positive experience for students, and a valuable partnership across departments. The course met goals of increasing awareness, but will be revised next year to balance robustness against time management. PIs of institutional training grants appreciated having a formalized way to address reproducibility requirements. It was particularly useful in addressing reproducibility and transparent reporting issues that are not always addressed in methods classes. Finally, this gave the librarian an opportunity to work as an equal co-instructor and build connections with a variety of faculty and professionals. The course demonstrated the role of library expertise in supporting the research lifecycle and essential skills for reproducibility and transparency in research.

Consumer Health Information Professionals as Leaders in Promoting Informed Decision-Making: Development of a Decision Navigation and Patient Support Program at a Major Urban Cancer Center

Practice Area: Global Health & Health Equity

Antonio P. DeRosa, AHIP, Program Lead, Decision Navigation & Patient Support / Oncology Consumer Health Librarian, Weill Cornell Medicine, Astoria, New York

Terrie R. Wheeler, AMLS, Director and Chair, Samuel J. Wood Library, New York, New York

Erica Phillips, Associate Professor of Clinical Medicine, Weill Cornell Medicine, ,

Mary Frances Emlen, Director, NewYork-Presbyterian, ,

Background: In 1989, the American Cancer Society (ACS) held “National Hearings on Cancer in the Poor” where they heard testimony from economically disadvantaged Americans of all racial/ethnic minority groups having been diagnosed with cancer. As a result, the ACS issued its “Report to the Nation on Cancer in the Poor.” There have been numerous studies and reports from government, non-profit, and commercial sectors on the benefits of supporting informed decision-making by empowering patients to take active roles in their care. Decision Navigation has emerged as an evidence-based practice, leading patients to feel confident and experience less regret in making treatment decisions.

Description: The Decision Navigation and Patient Support Program at the Weill Cornell Medicine Meyer Cancer Center (WCM-MCC) functions as a leader in providing decision-making support and promoting health literacy education among the oncology community through the coordination of a Patient Support Corps. Knowledge of cultural competence and an aptitude for serving underrepresented, socioeconomically diverse, and racial and ethnic minority populations, the program ensures patient comprehension of care and facilitates strategies to empower patients to advocate for their health. The program mentors a team of student interns to provide evidence-based decision navigation interventions, information counseling services, and health literacy awareness campaigns. The program liaises with clinical and administrative leaders in the WCM-MCC to improve the patient experience and overall outcomes through patient-centered care and decision navigation advocacy efforts. Community-based outreach and research is also a function of the program through collaborations with local and regional organizations and key external stakeholders.

Conclusion: The main goal of the program is to provide decision-support services to the oncology community to support a culture of patient-centered care and shared decision-making in an effort to improve the overall experience and health outcomes of patients at WCM-MCC. Other goals include deploying a framework for charting decision navigation interventions and information counseling patient encounters in the electronic health record (EHR); engage the WCM-MCC Patient & Family Advisory Council (PFAC) to garner input on Patient Support Corps initiatives and proposed practice changes; promote patient-centric and value-based care among WCM-MCC faculty, staff, and students by highlighting the importance of patient preferences, goals, and personal values when it comes to decision-making and advocating for one’s health.

Creating Videos to Support the University Community during a Rapid Shift to Online Course Delivery: A Democratic Team-Based Approach

Practice Area: Professionalism & Leadership

Gary M. Childs, Engineering (STEM-H) Librarian, West Chester University, West Chester, Pennsylvania

Background: Due to the shift to remote learning at a public university during the spring semester of 2020, the Libraries needed to immediately respond to dynamic changes in delivery of services and policies. The university has continued to operate remotely during the fall semester of 2020 and the Libraries have recently implemented a new discovery search tool. To meet the needs of the university learning community, the Libraries created an array of video tutorials to address commonly asked questions. As the perceived need for video materials grew, a team of librarians self-organized to create this content.

Description: This session will focus on the team-based processes of a group of librarians formed to hasten the creation, scope, and assessment of video content within an institution that highly values face-to-face instructional methods. The use of a flat hierarchical team structure and a consensus-based approach to address potential topics, themes, directions, statistics, and organization of content will be explored. This presentation will describe an ad hoc cohort founded to address an immediate need while honoring personal choice, autonomy, academic freedom, and responsibility. Democratic voice and inclusivity informs the views, processes, and actions described in this case study.

Results: The Libraries Tutorial Team created 13 videos during the summer of 2020, followed by 18 more videos in fall. There were 152 tutorial viewings that occurred over the summer and this increased to 761 views in the fall. The most viewed tutorials dealt with common library-related questions such as, performing basic searches using a discovery tool, accessing full text, obtaining print books, and using interlibrary loan. One discipline-specific tutorial demonstrating the use of the database, CINAHL was also viewed heavily during the fall semester. The team also reviewed survey results from library faculty and staff that pertained to tutorials. Library colleagues indicated specific content they wanted to see the team focus on and indicated that they would like training events centered around video creation.

Conclusion: As this effort is ongoing, limited evaluation data is available currently. Further statistics related to video content use and reference assistance will be analyzed following the conclusion of the spring semester.

Critical Appraisal Institute for Librarians: A Year Three Evaluation

Practice Area: Education

Marie T. Ascher, Lillian Hetrick Huber Endowed Director, New York Medical College, Valhalla, New York

Deborah A. Crooke, Associate Director, New York Medical College, Valhalla, New York

Amy Blevins, MALS, Associate Director for Public Services, Ruth Lilly Medical Library, Indianapolis, Indiana

Joey Nicholson, Interim Chair/Interim Director, NYU Health Sciences Library, New York, New York

Rachel Pinotti, AHIP, Director, Library Education & Research Services, Levy Library, Icahn School of Medicine at Mount Sinai, New York

Abraham Wheeler, AHIP, Health Sciences Librarian, Michigan State University, East Lansing, Michigan

Background: Critical Appraisal Institute for Librarians (CAIFL) is a professional development program for librarians aimed at developing critical appraisal skills. Many health sciences librarians provide evidence-based medicine (EBM) training at their home institutions. For many, curricular involvement is limited to teaching: asking questions (PICO); searching resources; and only a rudimentary coverage of critical appraisal. The goal of CAIFL is to enhance the abilities of health sciences librarians to take EBM training to the next level – to develop competencies in biomedical statistics and scientific methodology; and, to enhance the confidence and comfort of librarians in teaching concepts of critical appraisal.

Description: CAIFL was developed with funding from the National Network of Libraries of Medicine, Middle Atlantic Region. A multi-institutional planning team was created by identifying librarians who were engaging in critical appraisal instruction at their institutions, and a physician partner to develop the curriculum with us and provide a framework and content. The course that emerged is a fully online 8-week modular course employing a flipped approach, plenary sessions and weekly small group facilitated sessions, peer presentations, and a final journal club presentation. The course has run through three years. Evaluation has evolved from using known evidence-based evaluation tools to developing our own validated instrument.

Conclusion: This presentation will provide three years of evaluation data, the evolution of the institute, and the burgeoning community that is being formed as a result of the program. If accepted, outcomes will be expanded in this space. The institute has been well-received by attendees and has developed a good reputation as well as collaborative relationship with other EBM training opportunities for librarians. Additionally, program alumni are invited to attend and present at monthly journal clubs. This group will be surveyed for ongoing impact of their experience with the institute and ongoing participation in the community of practice.

Data Core 2.0: How Our Library Expanded the Secure Data Enclave across Institutions and into the Cloud

Practice Area: Innovation & Research Practice

Peter R. Oxley, Associate Director of Research Services, Weill Cornell Medicine, New York, New York

Terrie R. Wheeler, AMLS, Director and Chair, Samuel J. Wood Library, New York, New York

Background: Medical research frequently requires access to sensitive datasets, which stipulate access conditions, research purposes, and data retention or disposition requirements. These governance constraints, potentially combined with multi-institutional collaboration, lead to a tension between security and accessibility, and a need for an 'honest broker' to stand between researchers and the data. Medical libraries, with their expertise and understanding of data management, remote access issues, researcher needs, and licensing and contract management, are ideally suited to take on the role of managing institutional secure enclaves as the honest broker, and liaison between researchers, ITS, and data providers.

Description: Under the leadership of the library, our secure enclave "Data Core" has recently expanded to allow capacity for multi-institutional data sharing projects. This includes the construction of a learning healthcare system environment, which provides regular import and controlled export of patient data from a dozen hospitals. We have also hosted a series of projects that are part of the largest urban clinical network in the nation, providing data import from multiple sites, as well as the ability for researchers from multiple institutions to securely and efficiently access the data. Finally, recognizing limitations of existing on premise infrastructure for managing an economically scalable enclave, we have collaborated with our ITS cloud team to build a number of cloud solutions for our patrons, that still meet the requirements of security and efficient access.

Conclusion: Our library's ability to understand the research needs of faculty, and the security needs of ITS, has allowed us to stand in the middle and ensure that data are appropriately handled, without imposing prohibitive burdens on the faculty and research staff. Our experience has now allowed us to expand to more complicated research projects, leveraging the technical and governance knowledge we have gained, and building from the trust researchers have in our ability to manage their projects.

Delivering a Flexible and On-Demand Instruction Program (From Home)

Practice Area: Education

John A. Borghi, Manager, Research and Instruction, Lane Medical Library / Stanford Medicine, Stanford, California

Amanda Woodward, Web Services Librarian, Lane Medical Library, Stanford School of Medicine

Lily Ren, Research Communications Librarian, Lane Medical Library, Toronto, Ontario Canada

Hong-nei Wong, DVM, MLIS, MPVM, Medical Education Librarian, Lane Medical Library, ,

Background: Prior to the onset of the COVID-19 pandemic, our instruction program included a mix of "regular" classes, one-off workshops with standardized content and learning objectives, and on-demand sessions requested by an individual program, research group, or other units. In response to shelter-in-place orders, we immediately shifted our instruction online and subsequently saw a substantial increase in demand. To meet this and the changing needs of our researchers, we began to offer short (30-45 minute) "pop up" classes on topics, such as finding COVID-19-related literature, dealing with online health misinformation, reference management, and finding and evaluating preprints.

Description: Liaison librarians have proposed, developed, and led new “pop up” classes based on their interests and the needs of their liaison groups. As materials for each class are developed, they are shared with members of the research and instruction team for feedback. To evaluate “pop up” classes, we have developed a lightweight assessment form that was sent to all class participants during and following each class. Compared to our assessment process for our regular roster of classes, which asks class participants to rate how well a class met a standardized set of learning objectives, the assessment process for “pop up” classes simply asks them what they found to be useful and what from the class they want to know more about. Classes are continuously refined based on peer feedback, information from the assessment forms, and the experience of each instructor.

Conclusion: Due to the success of our “pop up” classes, they will remain part of our instruction program even after we return to in-person instruction. We have also worked with our Continuing Medical Education office so that class participants can earn CME credit for their attendance. A select number of classes initially taught as “pop ups” will be added to our regular roster of classes, meaning they will be taught on a regular basis and accompanied by standardized materials and online supplements maintained by the research and instruction team. In parallel, we will continue to stand up new “pop up” classes to meet the changing needs of our users.

Delivering a Pharmacy Orientation Online Using a Flipped Classroom Model

Practice Area: Education

Glyneva Bradley-Ridout, Liaison & Education Librarian, University of Toronto, Toronto, Ontario , Canada

Background: In fall 2020, all orientation activities at the Faculty of Pharmacy, University of Toronto were moved online, including the library orientation for new pharmacy students in a bridging program. Additionally, live time for the librarian to teach the students was reduced, requiring a solution to maintain the same learning objectives in the new timeframe. A flipped classroom model is a method of delivering instructional content using asynchronous pre-work combined with a live synchronous session. The purpose of this program was to move a traditional in-person orientation session online using a flipped classroom model.

Description: Students were required to complete 30 minutes of pre-work before attending a 1-hour live session. The pre-work materials were designed using Articulate Rise 360. Students were required to move through self-paced lessons on preparing for a search, including completing an activity which was submitted. Students were encouraged to complete the pre-work and activity, but no grades were assigned as incentive. The pre-work was followed by the live synchronous workshop which focused on hands on demonstrations and activities. Effectiveness of the orientation was evaluated using a qualitative student feedback form and by reviewing the quality of the pre-work submissions.

Conclusion: Students expressed positive views on the flipped classroom format in the feedback forms. The pre-work module specifically was shown to be effective, based on the number of students who submitted the optional pre-work assignment and the high quality of the submissions. A flipped classroom model may be a viable option for other librarians as they develop and deliver their orientation sessions in an online environment

Development of a Health Literacy Workshop

Practice Area: Education

Dana L. Ladd, AHIP, Health and Wellness Librarian, Health and Wellness Library, Lanexa, Virginia

Background: Health literacy is defined as, “the degree to which individuals have the capacity to obtain, process, and understand basic health information and services needed to make appropriate health decisions.” A consumer health librarian and two nurse educators

collaborated to develop and conduct health literacy workshops at multiple locations throughout the 1125 bed health system. The goals of the health literacy workshops were to teach health care providers skills they can use in the clinical setting to reduce the health literacy burden of their patients.

Description: The librarian and nurse educators planned and developed a health literacy workshop and offered CE and CME credit to attendees. Each member developed the workshop content for their health literacy specialty areas: overview of health literacy (definition, statistics, impact, and who it affects), assessment of patient literacy levels, demonstration of teach back, evaluation of written patient educational materials, and an overview of finding and evaluating consumer health information. The librarian scheduled two workshops to be held at the library located at the hospital’s main location and the nurses scheduled three additional workshops to be held at remote satellite locations. They scheduled the workshops over the lunch hour and provided lunches to employees. They advertised the workshops on the library’s website and through mass mail. Attendees completed an evaluation form following the workshop and again six months later.

Results: Forty-eight healthcare providers registered and thirty-nine attended a session. Following the program, most attendees (88.89%) reported having a very adequate understanding of why health literacy is important for their patient population. All (100%) reported they agreed that using the Teach Back Method improves patient understanding and helps reduce hospital admissions.

Conclusion: This collaboration allowed the librarian and nurse educators to use skills from a variety of backgrounds to create a health literacy workshop aimed at health care providers. There was interest and attendance from multiple health disciplines. These sessions were effective in increasing health providers’ understanding of health literacy and its importance in changing their patient education practice to incorporate health literacy practices. The collaborators plan to conduct the sessions again in subsequent years during Health Literacy Month and to conduct health literacy tabling events for health care providers to visit and ask questions. The content is also being developed as an online educational option to be offered for CE and CME credits to allow for asynchronous learning of the material.

Diversified: Supporting Diversity, Equity, and Inclusion (DEI) in the Clinical Setting

Practice Area: Clinical Support

Michelle R. Lieggi, AHIP, Clinical Research Librarian, N/A, San Francisco, California

Helen-Ann Brown Epstein, AHIP, FMLA, Informationist, Virtua Health Sciences Library, Mt Laurel, New Jersey

Kristin Chapman Willard, AHIP, Medical Librarian, Horblit Health Sciences Library, Danbury Hospital, DANBURY, Connecticut

Marilyn G. Teolis, AHIP, Clinical Medical Librarian, Library Service, Tampa, Florida

Background: Clinical librarians support research and patient care for clinicians in both inpatient and outpatient settings, and often work directly with patients and families. These populations are composed of diverse individuals and require strategies that acknowledge this diversity, as well as those that ensure support is equitable and inclusive. As organizations such as the American Medical Association and the American Nurses Association promote diversification in the workforce, and hospitals continue to treat COVID-19 and other conditions that disproportionately affect Blacks and Latinos, it is inevitable that clinical librarians will continue to serve diverse populations.

Description: Clinical librarian strategies for addressing diverse populations are reported in the literature and communicated at conferences and other professional venues. The authors, all clinical librarians, searched PubMed, CINAHL and the MLA website for recent reports (last 5 years) on clinical librarian activities around diversity, equity and inclusion (DEI). They also surveyed librarians via the MEDLIB-L listserv for further information on strategies for supporting DEI in clinical settings. The authors supplemented this information with their own experiences supporting DEI as clinical librarians. The strategies and programs related to clinical librarian support for DEI will be summarized, and feedback from participants encouraged to promote further discourse and clinical librarian work related to DEI.

Results: There appears to be little in the literature specifically about clinical librarians supporting DEI, although much about medical librarians serving diverse populations (e.g., LGBTQ). Of 52 respondents to the survey, 80% reported supporting DEI via reference, 46% via outreach, 44% via instruction, 40% via library management, and 42% via "other" (e.g., diversity committees, CME). Activities reported include creating library/resource guides and participating in presentations related to DEI. Many reported supporting DEI via literature searches and targeted collections.

Conclusion: In reporting on clinical librarian activities related to DEI, especially information gained from the experiences of the presenters and conference participants, the authors hope to present a more complete picture of clinical librarian activities supporting DEI in hospital and other clinical settings. Although there is still much work to do in our profession to promote DEI in our libraries and in our institutions, we hope presenting these strategies will help other clinical librarians in their quest to support DEI. Providing support that recognizes diversity, is equitable to all, and includes values and viewpoints of more diverse audiences can only contribute to better outcomes for patients and families seeking care in our institutions.

Diversify Your Health Sciences Course Syllabus: A Pilot Program

Practice Area: Education

Ellie Svoboda, Education Informationist, University of Colorado Anschutz Medical Campus, Aurora, Colorado

Lilian Hoffecker, Research Librarian, Strauss Health Sciences Library

Christi Piper, AHIP, Informationist, Strauss Health Sciences Library, Aurora, Colorado

Samantha Kennefick Wilairat, Graduate Assistant, Strauss Health Sciences Library, San Jose, California

Nina L. McHale, AHIP, Head, Education & Research, Strauss Health Sciences Library, University of Colorado, Colorado

Background: The Diversify Your Syllabi pilot program is a new initiative at the Strauss Health Sciences Library on the University of Colorado Anschutz Medical Campus. Inspired by a program on the neighboring University of Colorado at Denver Auraria campus, the dean of the College of Nursing approached the nursing liaison librarian in August of 2020 about launching a diversify your syllabi pilot program on the Anschutz Medical campus. The goal of the program is to help faculty build syllabi and reading lists that are inclusive of many viewpoints and perspectives as well as authors that come from diverse backgrounds.

Description: This pilot program is focused on the College of Nursing and launched in the spring of 2021, as faculty members prepare their summer and fall 2021 syllabi. At this early stage, the pilot program consists of a LibGuide with resources for faculty to use to diversify their own syllabus and librarian consultations, which allow the course instructors to share their goals for their syllabus and provide examples of the types of readings they are seeking. Librarians follow up after this consultation with aggregated resource lists. It is the faculty member’s responsibility to consider the resources and integrate the readings into the syllabus. Librarians will follow up with faculty members later in the semester to see how the implementation of the diversified syllabus was received by students.

Conclusion: Thus far the Diversify Your Syllabi program has been enthusiastically received by the Nursing faculty. Librarians have received requests to consult on eight nursing courses and have met with four faculty members. Once the pilot with the College of Nursing concludes, we will modify as needed, and then offer the service to other campus units. Our presentation will include examples of courses and suggested materials as well as lessons learned along the way.

Diversifying Library Skills of Undergraduate Student Workers in a Health Sciences Library: The INSERT Curriculum

Practice Area: Education

Kathryn M. Houk, AHIP, Health Literacy & Community Engagement Librarian, University of Nevada Las Vegas, Las Vegas, Nevada

Aidy Weeks, AHIP, Interim Director, UNLV Health Sciences Library, Las Vegas, Florida

Background: The University of Nevada Las Vegas’s Health Sciences Library includes a team of library faculty, staff, and undergraduate student workers, the latter of which were generally underutilized and not provided meaningful opportunities for skill development. Recently, the library developed a partnership program providing training for student workers that pairs them with a librarian to assist in completing projects. The presenters were both interested in having student workers participate in literature reviews for publications but recognized that skills development would be required. This led us to develop a curriculum of self-paced learning modules with scaffolded assignments.

Description: The INtroduction to Synthesis, Evaluation, and Review Technique (INSERT) curriculum was developed to provide self-directed learning for those with little to no experience in researching and writing synthesized literature reviews. The six self-directed modules guide learners through the search and synthesis process while providing scaffolded assignments. A final reflective project and feedback meetings with the librarian partner integrated throughout the curriculum provide the opportunity for reflective self-evaluation. In this presentation, we will discuss our main goals and theoretical influences while developing the curriculum, the content and structure of the modules, and the results and feedback from piloting the curriculum with three instructors and three undergraduate student workers. Our discussion will also include our plans for creating version two of the curriculum.

Conclusions: Three student-instructor pairs worked through the six modules mainly in the Spring of 2021. Time to completion varied and depended on both the student’s and the instructor’s schedules and capacity. Student feedback regarding the structure and content of the modules, as well as the time needed to complete each, were generally positive. Two example suggestions for improvement provided by the students centered on providing skills checks within the modules and providing alternative scenarios for learning concepts. Feedback from the instructors included providing more structure for individual assignments from the students, a better platform for access to the curriculum, and a briefer, more direct overview of the purpose and goals of the curriculum for potential instructors.

Do not Google your Symptoms!: Teaching Health Literacy to Undergrad Students

Practice Area: Education

Cecelia Vetter, AHIP, Instruction and Outreach Librarian, Concordia University St. Paul, St. Paul, Minnesota

Rachel I. Wightman, Associate Director for Instruction and Outreach, Concordia University, St. Paul, ,

Background: When assigning an in-depth research paper did not fit well into the undergraduate general education course Health Sciences, the library worked with faculty to adopt an assignment focused on health literacy. Librarians helped faculty create an assignment that matched the course’s focus on personal health and wellness and then developed an accompanying library instruction session to teach students how to find and evaluate health information on the internet. Discussions of bias within resources and how search engines can reinforce racism were included in the library information sessions to stress the need for critical appraisal online information.

Description: Process: During Spring 2020, a faculty member expressed to a librarian that the Health Sciences research paper received criticism in student feedback. The librarian suggested an assignment focused on health literacy concepts and worked with the faculty to create an assignment where students evaluate online health resources. Summer 2020 a one-shot information literacy session and LibGuide were created to teach students how to identify bias in online health information and understand racial bias within search engines.

Implementation: During Fall 2020, the four sections of Health Sciences attended the health literacy library one-shot and completed the new course assignment.

Evaluation: Librarians will meet with the Health Sciences faculty in early 2021 to gather feedback on the new assignment and suggest improvements. Librarians will also review the in-class activity documents from the library one-shot to improve health literacy and critical appraisal instruction.

Conclusion: Expected outcomes: The Health Science faculty will find that this new health literacy assignment fits well within the course material. Additionally, undergraduate students will now have the skills to critically evaluate online health information when researching their own health conditions. With this information, students will hopefully be able to identify commercial bias in online health information and be aware of racial bias in search engine results.

Emergency Distance R Programming Instruction: Lessons Learned and Bridges Built

Practice Area: Education

Fred Willie Zametkin LaPolla, Research and Data Librarian, Lead Data Education, NYU Langone Medical Center, BROOKLYN, New York

Background: At the outset of COVID-19, many bench researchers who rely on in-person work were unable to conduct their research. To help upskill during this time, a librarian at our academic medical center was recruited by faculty in our graduate biomedical sciences program to create R programming instruction for wet lab researchers working from home. The R training was in preparation for subsequent sessions on RNA-seq analysis with subject matter experts.

Description: In consultation with bioinformatics experts, a librarian developed and tailored a synchronous online curriculum, utilizing an outcomes-focused framework centered around skills students would need in subsequent RNA-Seq instructional sessions. Seven one-hour sessions were taught by a health sciences librarian to wet lab researchers, ranging from introductory sessions for complete beginner programmers through topics including data structures, data cleaning, visualization and basic analysis. These sessions were later offered by Zoom to general library patrons as well with less overt focus on bioinformatics skills. In turn lessons learned, particularly around common areas of confusion and difficulty, informed librarian-led instruction to biomedical/life sciences graduate students later in the summer.

Conclusion: Across classes, enrollments were robust: in terms of attendees in Zoom sessions 543 attended the 7 bench science R sessions, 602 logged into library-held R sessions, though these numbers count “seats” filled, not unique individuals. Of those in the wet lab researcher group, students were deemed prepared for subsequent materials on RNA-seq by informatics instructors and evaluations were positive. The library sessions were also positively received based on evaluations, but the differing levels and background in coding represented a challenge to teach effectively to all. Finally the lessons learned helped improve learning activities for the more in-depth masters and PhD classes, and students evinced a high level of mastery on their final projects and evaluations were positive

Employing Community Wellness Liaisons to Create More Inclusive Public Libraries

Practice Area: Global Health & Health Equity

Rochelle C. Cassells, Research Associate, University of Utah/Genetic Science Learning Center, Salt Lake City,

Louisa A. Stark, Professor, Human Genetics, Salt Lake City, Utah

Background: Lack of access to reliable health information contributes to pervasive racial and ethnic health disparities. Libraries, through their resources and programs, are sources of health information for the public. Our previous research found that individuals from diverse communities are unaware of library services and therefore underutilize this resource. Such outcomes are partially explained by the fact that these individuals do not feel welcome in their local libraries.

Description: We developed a partnership with a research university, two library systems, and leaders from five communities underrepresented in biomedical research to address this gap. Our goal was to implement an intervention that increases the capacity of libraries to meet the health information needs of diverse communities. This intervention rests upon the hiring of five Community Wellness Liaisons (CWLs), one from each community, who were expected to improve the connection between diverse communities and libraries. The CWLs received library-specific training from library supervisors and health information training from a university health sciences librarian. CWLs also completed level one of the Consumer Health Information Specialization. Monthly meetings gave partners an opportunity to share information and to report on successes and challenges. The CWLs met with their community leaders to identify high-priority health concerns and with their library supervisors to plan events. The evaluation employed a mixed-methodology approach and explored partnership functioning, goal achievement, and project value.

Conclusion: Prior to the COVID-19 pandemic, the CWLs reported interactions with >1700 individuals (55% with diverse community members); 25% of interactions were health related. They assigned 90 library cards (33% to diverse community members) and completed 36 programs and outreach across five health domains. One CWL shared health resources with 643 Spanish-speaking businesses over a six-month period. All stakeholders believe that the project is valuable and has the potential for lasting impact, especially regarding the perception of what libraries offer to diverse community members. One CWL said, “when you [have] worked in the community then you kind of know why a CWL is necessary because not everybody has access to [health] information,” and a leader noted that individuals from their community are becoming more “engaged in their own health.” Additionally, librarians shared that the project emphasized the need for stronger health reference and cultural awareness trainings for library staff. The lessons learned, including how to navigate multistakeholder partnerships and how to stimulate interest in library careers among underrepresented minority individuals, provide insights to others interested in using this model.

Empowering Community Health Organizations with Community Health Maps

Practice Area: Education

Tyler Nix, Informationist, University of Michigan, Ann Arbor, Michigan

Marisa L. Conte, AHIP, Associate Director, Research and Informatics, University of Michigan, Ann Arbor, Michigan

Alexandra Rivera

Justin Schell, Director, Shapiro Design Lab, University of Michigan Library, Ann Arbor, Michigan

Background: In 2019 and 2020, the University of Michigan Libraries partnered with the National Library of Medicine (NLM) Specialized Information Service's Community Health Maps (CHM) program to offer a series of virtual and in-person mapping and geospatial training workshops.

The workshops introduce participants to no-cost / low-cost geospatial tools with the goal of empowering community health organizations - especially those that focus on serving vulnerable populations - with the knowledge and resources to collect, analyze, and share spatial data to address health challenges within their communities (e.g., visualizing local health risks, disease prevalence, hazards, clinic locations).

Description: This education initiative consisted of hosting four workshops at four University of Michigan campuses in the spring of 2019. Volunteer coordinators - an interdisciplinary group of librarians and faculty/staff champions from across the campuses - promoted the workshops and secured food, facilities, and loaner laptops. The workshops were developed and led by Kurt Menke of Bird's Eye View GIS, whose time, travel, and lodging were funded in full by the NLM CHM program.

Participants gained hands-on experience in creating a spatial data collection form, collecting field data, and using their data to generate a web-based map and a desktop map in the open-source mapping software, QGIS. Each in-person workshop concluded with moderated discussion time to encourage connections among local participants.

In 2020, two virtual follow-up workshops introduced participants to collecting field data, and to mapping COVID-19 in QGIS using publicly available data.

Conclusion: The in-person and virtual sessions drew nearly 300 attendees representing a diverse range of community members, from local health department staff to researchers and students, both domestic and international.

Anecdotal feedback following the workshops was very positive and the results of a formal assessment will be provided in the session. We look forward to this program's potential to

empower community health champions to meet their geospatial and mapping needs, and will report on future plans.

(Note- The workshop curriculum does not represent an endorsement of any software. The authors have no conflicts of interest to report.)

Enhancing Biomedical Research Using Common Data Elements: Roles for Librarians

Practice Area: Information Management

Lisa Federer, AHIP, NLM Data Science and Open Science Librarian, National Library of Medicine, North Bethesda, Maryland

Background: Common Data Elements (CDEs) are standardized, precisely defined questions paired with a set of specific allowable responses, used in common across different sites, studies, or clinical trials to ensure consistent data collection and enable data interoperability across sites, studies, or trials. While CDEs have been used in National Institutes of Health (NIH)-funded research for years, their value to increasing the efficiency of research and pace of progress has been highlighted by the urgency of addressing the COVID-19 pandemic. Efforts to develop, adopt, and use CDEs have accelerated at the NIH with leadership from the National Library of Medicine (NLM).

Description: This talk will provide an overview of CDEs and how they can be used to facilitate data sharing and interoperability, enabling integration and meta-analysis of data across multiple studies. This talk will also outline how CDEs have been developed and used to help speed COVID-19 research, and how this work also provided a catalyst to increase adoption of CDE use across biomedical research. In addition, this talk will describe NLM tools and resources for working with CDEs and discuss how librarians can provide support for researchers interested in using CDEs and provide outreach at their institution to increase awareness of the usefulness of CDEs.

Conclusion: As NIH continues to support the use and development of CDEs in patient registries, clinical studies, and other human subjects research through incentives made via funding announcements, awards, contracts, and other resources, CDEs will become increasingly relevant for researchers and the librarians who work with them. Librarians who can provide guidance, outreach, and support for CDEs will be a valuable asset to help improve data quality and enhance research reproducibility at their institution.

Enhancing Research Reproducibility through Integrated Data Retention: Using the Library Data Catalog as the Hub for Data Management

Practice Area: Innovation & Research Practice

Peter R. Oxley, Associate Director of Research Services, Weill Cornell Medicine, New York, New York

Terrie R. Wheeler, AMLS, Director and Chair, Samuel J. Wood Library, New York, New York

Background: Better access to the data, workflows and analyses behind published research improves reproducibility, increasingly meets funder mandates, and protects individuals and institutions against allegations of research misconduct. Key elements of this capacity include the ability to identify, locate, and then access the data used, and produce immutable records of the analytical process. Unique difficulties arise when research involves confidential data, whose access cannot be made available readily. Nevertheless, it is important to be able to publish the rules by which access can be granted.

Description: To address these challenges, we created a unique data catalog that integrates data discoverability, data access governance, electronic lab notebooks (ELNs) and a filesystem management tool. ELNs capture research workflows and store small datasets. The file management system allows all non-ELN files associated with a project to be specifically tagged and tracked within the institutional storage infrastructure, capturing file movement and changes, and assisting with data archiving at the point of publication or project closure. All these features are rolled into our data catalog, allowing centralized searching and rapid retrieval of data. By capturing data governance, we ensure that access and data reuse conditions are immediately visible to the searcher and the library data curation team, who broker access to these data.

Conclusion: Our library has been able to provide a key role in architecting the data management solutions for our institution, leveraging our knowledge of stakeholder requirements, from funders, through to researchers and administration. Trust and competence were built by early involvement in the data retention policy discussions, and by demonstrating the capacity to handle data cataloging and data governance concerns. As implementation continues, we will be measuring user uptake, surveying stakeholder satisfaction, and auditing our ability to produce data in line with institutional and third party retention policies.

Facilitating Updates to Faculty Governing Documents: A Better Process for Encouraging Participation

Practice Area: Professionalism & Leadership

Christi Piper, AHIP, Informationist, Strauss Health Sciences Library, Aurora, Colorado

Dana Abbey, AHIP, Community Engagement Coordinator, Strauss Health Sciences Library, Aurora, Colorado

Tina M. Moser, Access Services Librarian, Access Services Librarian, Aurora, Colorado

Background: University librarians have Faculty status and privileges, and therefore, have official Faculty Guidelines including Appointment and Promotion (A&P) documentation. A recent review and update of all Regents Laws and Policies and University-wide Administration Policy Statements (APS) required the Library Faculty Senate to update their A&P documents to align with sweeping changes at the system-wide level in less than a year. The objectives of the update were to: 1) align Library procedures with campus-wide faculty procedures, 2) enable Library Faculty to better understand and apply the library appointment and promotion processes, and 3) ensure that all Library Faculty voices were heard.

Description: A task force was appointed to determine updates and provide recommendations. A three-step process was used to garner Faculty feedback, updates, and recommendations. Step 1 sought to identify items for updating and gathering information about changes required to satisfy the new system policies. Step 2 included analyzing and clarifying the initial responses. Step 3 involved presenting the recommended changes via a series of three surveys. These surveys provided 2-3 recommendations for each item and an opportunity for Faculty comments. A fourth survey was designed to address topics of significant disagreement and to present alternatives for those items. At the conclusion of the fourth survey, all updates were made to the official document and presented as a final draft in its entirety to Faculty. One open discussion session was held for final comments before the official vote to approve the document.

Conclusion: The processes employed greatly streamlined and improved participation ensuring that all Faculty had input into the documents. The task force found that by providing concrete recommendations for how to change the document, discussion was improved by focusing on specific concerns. Using surveys allowed feedback from all Faculty, even those who typically did not speak up as much during in-person meetings. Additionally, the use of surveys forced discussions to focus on topics that were already identified as the most important and kept faculty on track with their feedback. This paper discusses the overarching framework used for this process, as well as examples of how surveys and individual questions were structured to receive feedback in an efficient manner.

Hard Skills for Software: How We Revolutionized Scientific Software Provisioning at an Academic Medical Institution

Practice Area: Innovation & Research Practice

Peter R. Oxley, Associate Director of Research Services, Weill Cornell Medicine, New York, New York

Terrie R. Wheeler, AMLS, Director and Chair, Samuel J. Wood Library, New York, New York

Background: Academic institutions employ an increasing diversity of proprietary and open-source scientific software products for research and teaching. This creates a complex set of interactions between vendor contract management, license requesting, software provisioning, billing, and user education. The library is optimally placed to be the central administrator of all these moving parts. At our institution, the library has increasingly taken over the role of managing scientific software, dramatically improving patrons' experience. This presentation will describe the key elements of our software management approach, and notable pitfalls that can be avoided.

Description: Following an approximately chronological narrative of our experience, we will walk through the following steps for setting up an exceptional software service:

1) Surveying user needs
2) Identifying existing licenses
3) Cataloging software and receiving requests
4) LibGuides, and user education
5) Centralizing and renegotiating contracts
6) Financial sustainability and customer billing
7) Partnering with ITS for streamlined software deployment
8) Stakeholder satisfaction, and how to measure success

Conclusion: Over the course of three years, we have processed 3,045 software requests for 19 different scientific software products. We have saved researchers thousands of dollars through bulk purchasing and institutional discounts, and provided a central point of help for researchers, teachers and students who needed to transition to remote work and learning during COVID, while still maintaining access to their software. We have also continued to identify new product needs, and advocate for our researcher's needs when communicating with software vendors.

Health and Wellness in Uncertain Times: Supporting StudentsThrough The Creation of a Physical and Digital Consumer Health Information Center

Practice Area: Information Services

Andrea C. Kepsel, AHIP, Health Sciences Educational Technology Librarian, Michigan State University, East Lansing, Michigan

Iris Kovar-Gough, AHIP, Health Sciences Librarian, Michigan State University Libraries, East Lansing, Michigan

Background: Recognizing the importance of student health and wellness, and the role of the library as a connector to on and off-campus resources, two health sciences librarians created low-barrier and stigma-free access to curated, high quality health information for students. The resulting collection features information from campus and local community organizations paired with consumer health books, on topics including housing, food and nutrition, legal services, counseling, and mental health support. Use of the collection encourages students to become informed health information consumers, assisting navigation of the various social, health, and emotional challenges that happen during one’s time at a university.

Description: An internal microgrant provided funds for purchasing a movable shelving unit, display materials, and printing of informational handouts from local organizations. Existing books from main stacks were relocated to the new location and additional titles were purchased to address gaps. Use of the books is measured by circulation statistics, and the handouts are monitored for how frequently they need to be reprinted. The SARS-CoV-2 pandemic has only amplified the importance of supporting student health and wellness. In response, the librarians created a virtual version of the collection as a libguide with links to campus and community organizations and purchased more ebooks. The guide is assessed based on the number of views and clicks on links and is included in the library’s online promotion efforts.

Conclusion: Despite the difficult circulation circumstances that limited promotion and in-person circulation and browsing of the collection during the COVID19 pandemic, the collection has been a welcome addition to our library. It emphasizes our commitment to student health and wellness, and by connecting students with local resources we are exemplifying our role as a hub of information. Our goal is to continue expanding the collection and re-assess, affirm, and promote local organizations more as students return to campus in Fall 2021.

Highlighting Contributions of BIPOC Students, Faculty, and Staff through Library Social Media

Practice Area: Education

Nina Stoyan-Rosenzweig, Senior Associate in Libraries, University of Florida, Gainesville, Florida

Chloe Hough, Evening Circulation Supervisor, University of Florida Health Science Center Library, Gainesville, Florida

Background: Black, Indigenous, people of color (BIPOC) are underrepresented in the STEM fields- and those black, indigenous people of color who do work in these fields are underrepresented in how their work and their lives are presented in the University setting. At the same time, highlighting the work of such individuals is vital to encourage more participation, diversity, and inclusion. This presentation describes a new library project to highlight people of color on library social media, with a permanent presence on the web.

Description: Individuals are identified through research- especially to learn more about those involved early on in the institution’s history as it grappled with integration, and through recommendations from library liaisons. Those selected for this program can be alumni of undergraduate or professional programs, current or past faculty and staff. A brief biography is prepared from records and sources and the individual is contacted and asked for their permission and support (and a photograph). If the individual wants to have their biography highlighted, their agreement is documented, the material is then scheduled for publication and run on Twitter and Facebook, as well as on the Library’s website.

Conclusion: The project thus far has been very successful in attracting interest and engagement through the posts- the engagement with posts thus published has been 21 times higher than for other library social media posts. We will analyze social media engagement metrics and will develop a report on the qualitative as well as quantitative information generated by the project. At the time of the conference, there will be significantly more data on the response to this project and how it engages students at the university.

Hosting a COVID-19-Centered Data Sciences Training Series

Practice Area: Education

Fred Willie Zametkin LaPolla, Research and Data Librarian, Lead Data Education, NYU Langone Medical Center, BROOKLYN, New York

Nicole Contaxis, Data Librarian, Lead of Data Discovery, NYU Health Sciences Library, New York, New York

Alisa Surkis, Assistant Director, Research Data and Metrics/Vice Chair for Research, NYU Health Sciences Library, New York, New York

Background: As COVID-19 emerged, researchers quickly initiated studies to better understand all aspects of the pandemic, and research support staff worked to develop resources to facilitate these studies. Our institutional clinical informatics group created a de-identified COVID-19 EHR dataset and reached out to the library to provide training and support to researchers seeking to use this dataset. Recognizing the broader data-related challenges of researchers seeking to work in this area, our library leveraged its position as an institutional hub for data-related education to provide a series of classes to support COVID-19 research.

Description: We planned a series of synchronous online workshops, making use of existing content on the COVID-19 de-identified dataset, adapting other materials to be COVID-19 focused, and leveraging institutional relationships to bring in classes with non-library instructors. We planned seven educational workshops, four classes were taught by library faculty -- accessing our institution’s COVID-19 de-identified data, an introduction to R for pulling data from the data lake, R for data cleaning and analysis, and finding COVID-19 data -- and three by partners from across the medical center -- the use of medical center tools to build COVID-19 cohorts, a local COVID-19 risk index, logistic regression and survival analysis, and finding COVID-19 data.

Conclusion: Enrollments were lower than we anticipated for our sessions ranging from a high of 42 to a low of 2. These results were much lower than other online trainings done earlier in the pandemic on REDCap and de-identified EHR data. We believe the low turn out was due to Zoom fatigue as the pandemic wore on and decreased interest in professional development. We surveyed participants for attitudinal evaluation, but return rates for the survey were very low, with only 8 individuals sending them back. The challenges in this series highlight a degree of fatigue and lack of enthusiasm as the year wore on.

Improving User Experiences with Electronic Resources: A Database Description Project

Practice Area: Information Management

Lauren Martiere Fletcher, Instruction & Research Librarian, University of Mississippi Medical Center/Rowland Medical Library, Jackson, Mississippi

Sarah C. Adcock, Instruction & Research Librarian, University of Mississippi Medical Center/Rowland Medical Library, Jackson, Mississippi

Background: This session examines how a Research and Instruction Librarian team updated and re-envisioned a library’s A to Z Database list to include relevant information that would suit the needs and requests of users across an academic health science center campus.

Description: The team determined that each database description should include alternative names, subjects, content categories, date range, icons, and vendor information. The reimagined descriptions removed extraneous vendor content that confused users and focused on information necessary to make quick determinations on which databases were best for their information needs. They worked systematically to correlate subjects and content with the educational, clinical, and research missions of the university. The project team selected 20 subjects, 21 content categories, and four resource icons for possible assignment to each database. All categories went through review by the library director and all faculty librarians. The team created a Microsoft Form to collate all data for each database in one location and used the collated information to update descriptions. Decisions on database best bests and content experts, features of the Springshare platform, were made by consensus.

Conclusion: The project team anticipates that updating the A to Z Database page will improve user access and experiences when using the library website and electronic resources. The team will compare vendor-reported database usage statistics, Springshare A to Z Database Tracking statistics, and Google Analytics data to determine if description updates correlate to an increased usage of available databases. Additionally, the project team will collect user opinions on updates through instruction and research/reference interactions.

Incorporating Self-Directed Learning Skills into Peer-to-Peer Search Seminars to Enhance Searching Competencies

Practice Area: Information Services

Phill Jo, AHIP, Head of Access Services/Associate Professor, University of Oklahoma Health Sciences Center, Oklahoma City, Oklahoma

Tara R. Malone, MLIS, Assistant Professor/Head of History of Medicine and Serials, Robert M. Bird Library, Oklahoma City, Oklahoma

Sheryl Lynn Hamilton, Assistant Professor of Research / Reference & Instructional Librarian, Robert M. Bird Library, University of Oklahoma Health Sciences Center, Oklahoma City, Oklahoma

Shari Clifton, MLIS, AHIP, Professor / Associate Director & Head, Reference, Robert M Bird Health Sciences LIbrary, Oklahoma City, Oklahoma

Background: Since 2016, librarians at the [redacted] library have engaged in peer-to-peer (P2P) seminars to expand the skills of searchers, share searching experiences, and address performance indicators from MLA Professional Competency 1: Information Services. To maximize the effectiveness of these seminars and better improve searching knowledge for team members, librarians recently implemented a pilot project applying self-directed learning (SDL) principles to the P2P seminar process with particular focus on self-identifying one’s own knowledge gaps, establishing goals for the seminar, and reflecting on the seminar discussion to integrate new knowledge.

Description: At times seminar participants have been challenged by an inability to diagnose their own learning needs, set up individual objectives, reflect and assess their learning strategies, or measure learning outcomes. To address this, a form incorporating an SDL framework was developed. Prior to each P2P seminar, the searcher used the form to identify knowledge gaps, challenges, and goals/objectives associated with the search. The search topic and strategy were also required for the submission. After the seminar, searchers completed information about the seminar discussion, as well as how/if knowledge gaps were addressed, whether objectives were met, and what lessons were learned. The team reviewed data from completed forms during subsequent seminars to assess the effectiveness of the SDL framework in addressing challenges.

Conclusion: While the integration of SDL principles into the P2P search seminar is still in its early stages, the goal is to provide individuals with tools to enhance their ability to assess, reflect, and apply SDL skills to searching. Data collection is underway, and conclusions will be updated prior to MLA 2021.

Informationists Collaborate with Nurse Educators to Integrate a Virtual Dissection Table into a Staff Development Curriculum

Practice Area: Education

Don P. Jason, Health Informationist, University of Cincinnati Libraries, Cincinnati, Ohio

Alison Trammell, Critical Care Nurse Residency Program Facilitator, University of Cincinnati Medical Center, , Ohio

Tiffany Grant, Research Informationist, University of Cincinnati, Health Sciences Library, Cincinnati, Ohio

Background: Informationists and nurse educators launched a pilot program that integrates a Sectra Virtual Dissection Table into the Critical Care Nurse Residency Program (CCNRP) curriculum at the University of Cincinnati Medical Center (UCMC). CCNRP is a mandatory education program for novice, critical care registered nurses who are hired into UCMC's intensive care, emergency care or progressive care areas. Cohorts range in size from 12 to 32 members. CCNRP meets two days a week for seven weeks. In the past, the course featured traditional lectures and PowerPoints. Now the CCNRP cohorts spend a portion of their time in the Donald C. Harrison Health Sciences Library engaging in interactive learning with the virtual dissection table.

Description: The CCNRP course material is divided into several modules. Each module begins with an overview of anatomy and the normal function of the body system. Nurses learn this portion of the course material on the Sectra Virtual Dissection Table. The body systems covered in the course include cardiac, neurological, pulmonary, gastrointestinal, renal, hematological and endocrine. The course also covers trauma and multi-system organ failure. The CCNRP course instructor develops lesson plans and storyboards for the Sectra table. After nurses complete the seven-week course, they take a post-course survey. This survey measures satisfaction with the virtual dissection table and residents’ perception of knowledge gained of anatomy. The mixed-methods survey instrument contains both quantitative and open-response questions.

Conclusion: The first cohort to experience the Sectra table completed the CCNRP in mid-April 2019. At the conclusion of the course, the nurses completed an evaluation. Their reaction to the Sectra table was overwhelmingly positive. One student shared, “I really liked the Sectra table because I learn better visually and hands-on, and this table definitely provides both aspects.” Another student shared, “I would recommend using this technology because it helped me study anatomy and pathophysiology together. It would be great for other students to have a chance to experience this.” Six cohorts have experienced the updated CCNRP curriculum. In total, 105 nurses have utilized and evaluated the Sectra Table. This paper will present an analysis of 105 post-course surveys.

Leveraging Colleague Expertise for a Library-Wide Staff Training Day

Practice Area: Professionalism & Leadership

Hannah F. Norton, AHIP, Chair, Health Science Center Library - Gainesville, University of Florida, Gainesville, Florida

Background: While many individual professional development opportunities are available to staff at our library, the idea of an All Staff Training Day arose as a means for employees across our two sites to build rapport, share their expertise, and learn from one another. Staff across the library contributed by brainstorming topics and activities to include, presenting to their peers, and providing feedback on successes and potential improvements for subsequent iterations of the training.

Description: A sub-team of three individuals (one paraprofessional from each location and a librarian administrator) developed an initial slate of topics, followed by an email solicitation to the rest of the staff for additional ideas; contributors were encouraged to consider both topics they wanted to learn about and topics they felt they could teach to their peers. After several reschedules based on staff availability, the first All Staff Training Day took place in January 2019, with staff from our smaller Jacksonville branch traveling to Gainesville so that everyone could participate together in person. Interactive sessions, such as a team-building exercise, diversity training, and hands-on technology training, were interspersed with short didactic sessions including refreshers on using specific literature databases and brief presentations by project teams (e.g. assessment team, marketing team).

Conclusion: Results from a follow-up evaluation survey, distributed electronically shortly after the event, indicated that attendees valued the All Staff Training Day as a whole. Overwhelmingly, participants found sessions both helpful to their work (e.g. technology and diversity trainings) and enjoyable (e.g. an initial team-building exercise and a personality test conducted over the lunch hour). Staff identified a number of topics they would like covered in subsequent trainings as well as logistical suggestions for an even more inclusive future iteration (e.g. involving more collaborative scheduling of the event and inviting student workers to attend). A second All Staff Training Day, incorporating these suggestions, is being planned for 2021.

Leveraging Librarian Involvement in Systematic Reviews to Amplify (Lack of) Diversity

Practice Area: Information Services

Rachel Keiko Stark, AHIP, Health Sciences Librarian, California State University, Sacramento, Sacramento, California

Background: The purpose of this program is to provide an example of how librarians can leverage their involvement in Systematic Reviews to bring attention to issues of diversity in health sciences research. This program will highlight the issues of representation of diverse communities in health sciences research and literature and why representation is not only important but necessary. This program will explore why systematic reviews need to and should highlight issues of representation for diverse communities and provide examples of ways to accomplish this important work that center the role of the librarian.

Description: Librarians who are involved with Systematic Reviews have the ability to shape what will be analyzed in a Systematic Review. Depending on the role a librarian plays, there are many ways that a librarian can suggest, request, or require that the Systematic Review team consider, highlight, and address issues of representation and diversity in their work. This case study presents what one librarian is doing to bring issues of diversity and representation to Systematic Review support offered at a large public university. It will provide examples of different ways that diversity and representation have been incorporated into systematic reviews conducted at this university and will provide examples of the various roles of the librarian how those different roles can take different actions to highlight the importance of diversity and representation in the work of a systematic review.

Conclusion: This case study presents multiple examples where the role of the librarian has successfully engaged with the systematic review on issues of representation and diversity during the review process. There is currently no data available on published systematic reviews, but there are helpful insights into barriers faced when this work is written into in the systematic review and the systematic review undergoes resistance during the peer review process. Is the hope of the author that by the time of this presentation, concrete examples of published reviews will be available.

Librarians Promoting an Institutional Culture of Research Integrity

Practice Area: Education

Terry Kit Selfe, AHIP, Translational Research and Impact Librarian, University of Florida, Gainesville, Florida

Michelle Leonard, Associate University Librarian, Marston Science Library, University of Florida, Gainesville, Florida

Background: One of the co-authors was approached by our university’s research and research integrity administrators to quickly develop an online responsible conduct of research (RCR) training program for staff working in research labs closed due to COVID-19. A pre-series survey was administered to identify the potential audience, discover their level of RCR knowledge, and understand their goals for enrolling in such a program. The responses informed the development of a synchronous, active learning RCR seminar series taught primarily by library faculty and designed to enhance our institution’s culture of research integrity by providing training to previously underserved research personnel, among others.

Description: Of 404 potential participants, 307 completed the pre-series survey (76% response rate). Based on survey responses, federal RCR program content requirements, and available expertise, the resulting program comprised a total of 22 online sessions delivered synchronously throughout the summer of 2020. A certificate option requiring completion of 3 pre-requisites, 13 core courses, and 2 electives was available for those wanting to satisfy NSF and USDA RCR training requirements. Core topics included research misconduct, ethics of authorship, collaborative research, conflicts of interest, data management, research replicability, and peer review. Each session included active learning elements, such as polls, case studies and small-group discussion. Librarians taught all but four sessions; others with specific expertise were recruited for institutional compliance sessions. A program evaluation was administered at the end of each session. The active learning elements were identified as strengths of the sessions.

Conclusion: This pilot program was an unqualified success. Demand was high. Program registration, capped at 300 registrants per session, had to be closed early as we had reached maximum capacity. Even though the labs reopened the week the series began, average per session attendance was >200 and 123 people completed all requirements for the RCR certificate. Session evaluations were extremely positive. Attendees appreciated the opportunity to actively engage via polls, chat, and discussion rooms. Given the strong response and commitment from our research integrity office, the RCR seminar series will be offered every summer. Furthermore, the US Health and Human Service’s Office of Research Integrity approved a grant (as yet unfunded) to refine and expand this librarian-led RCR program.

Librarians Transforming Medical and Dental School Curricula to Meet Accreditation Standards

Practice Area: Education

Jessica A. Koos, AHIP, Health Sciences Librarian, Stony Brook University, Stony Brook, New York

Jamie Saragossi, Head of the Health Sciences Library, Stony Brook University Libraries, Stony Brook, New York

Background: Due to the rapidly changing nature of medical literature, accrediting bodies for health professions schools want to ensure that graduates are equipped to continuously update their knowledge after graduation. The School of Medicine was cited by the Liaison Committee on Medical Education (LCME) for lacking self-directed learning in their curriculum, and the School of Dental Medicine also had similar concerns regarding their Commission on Dental Education (CODA) accreditation. By incorporating principles of evidence-based practice (EBP), liaison librarians for the SOM and SODM created activities to integrate self-directed learning objectives into the curriculum to satisfy LCME and CODA standards.

Description: The proposal included the creation of an asynchronous EBP tutorial, a one-shot class on searching skills, and a class in which students work together in small groups to present critical appraisals of individual research studies. Each of these components are being implemented at various stages in the curriculum. This new approach will provide the liaison librarians with the opportunity to introduce, reinforce and ultimately evaluate the students’ comprehension of EBP. The asynchronous tutorials include an interactive component, requiring students to correctly answer questions about core principles of evidence-based practice before advancing to the next section. Therefore the successful completion of the tutorial indicates a baseline of student comprehension. The librarians plan to survey the students about their experience and comfort level incorporating EBP into their clinical practice after having completed these trainings throughout the curriculum.

Conclusion: Many health sciences librarians are involved with the accreditation or reaccreditation processes of the academic programs they serve. Developing content that can help the academic programs meet necessary accreditation standards, while enhancing the research and critical thinking skills of the students, is an opportunity for librarians to demonstrate the importance of their contributions to the curriculum.

Making Hard Choices a Little Easier: Gathering Actionable Data from the Campus Community to Inform Collections Decisions

Practice Area: Information Management

Nina L. McHale, AHIP, Head, Education & Research, Strauss Health Sciences Library, University of Colorado, Aurora, Colorado

Yumin Jiang, Head, Collection Management, Strauss Health Sciences Library, University of Colorado Anschutz Medical Campus, Aurora, Colorado

Background: In late 2019, faced with a fiscal shortfall due to a flat budget and the rising cost of electronic resources, the Head of Collection Management and the Head of Education & Research at the University of Colorado Strauss Health Sciences Library sought feedback from the campus community about hard renewal choices. Previously, subject liaison librarians in Education & Research were asked to solicit feedback from their liaison audiences; however, this feedback was anecdotal and limited to the groups of faculty and clinicians with whom the liaisons had had the most interaction. In the fall of 2020, the library’s materials budget faced additional threats due to COVID-19, so the survey was updated and repeated.

Description: The two department heads created a campus-wide survey for the clinical, research, and academic library users at the Anschutz Medical Campus. Based on a journal cancellation survey created by librarians at the University of New Mexico (Nash and McElfresh, 2016), the survey asked library users to rate resources based on their use of them: “Never Use,” “Rarely Use,” “Useful,” “Important,” or “Essential.” Survey respondents were provided the opportunity to comment on how they used the resources as well. The survey was distributed via a campus-wide email list and promoted by the liaison librarians, and in 2019, 630 results were received, and in 2020, 512 results were received, each in a span of about four weeks. From the results, the Head of Collection Management focused on items’ data in “Important” and “Essential” categories to guide decisions on electronic resources based on the survey respondents' estimations of their importance.

Conclusion: In both years, the survey provided actionable data to guide renewal decisions with greater confidence; greatly increased and improved communication and user engagement in the collection decisions process; educated users campus-wide about the nature and challenges of the library’s materials budget; and finally, minimized the number of complaints about electronic resources that were not selected for renewal.

Making the Most of the Month: Design and Implementation of an Advanced Searching Skills Crash Course for Pharmacy Rotation Students

Practice Area: Education

Hilary M. Jasmin, MSIS, Research and Learning Services Librarian, University of Tennessee Health Science Center, Memphis, Tennessee

Background: Pharmacy students enter Advanced Pharmacy Practice Experiences (APPEs) midway through their third year of the PharmD curriculum, and one variety of an APPE is an academia rotation. In these rotations students are expected to contribute in the research process with their faculty preceptors, often in developing the literature review for the faculty’s manuscripts. The librarian was called upon to develop an advanced searching crash course that could be added at the beginning of rotations to enable the student to fully contribute to the month’s work.

Description: A three-session instruction series was developed to meet the needs of preceptors as their rotation students arrived with minimal experience in searching for and synthesizing information. The intent of the series was to guide students directly in application of skills including concept development, use of appropriate controlled vocabulary and entry terms, translating searches into multiple databases, and finally running searches for export into citation software. A pre/post self-assessment with five outcomes across a Likert scale was disseminated to each student to measure their confidence in their searching skills.

Conclusion: By the end of April 2021, 37 students have completed the crash course. In their pre-assessment, 77% of responses answered “absolute beginner” or “novice”. In their post-assessment, 100% answered “developing” or “competent”. In addition to these marked improvements in their confidence, the students have unanimously expressed their enthusiasm for the skills they have been able to apply, and many expressed that they would have liked this cohort-based format much sooner than rotations. The crash course will continue to be enhanced and modified as needed, and future opportunities for new collaborations are being sought

Managing Library Staff in Times of COVID: What We Can Learn from the Remote Work Literature

Practice Area: Professionalism & Leadership

Ardis Hanson, AHIP, Assistant Director, Research and Education, University of South Florida USF Health Libraries, Tampa, Florida

Diane Fulkerson, Library Director, Drake Memorial Library SUNY Brockport, Brockport, New York

Background: Before the global COVID pandemic, the literature shows that, overall, remote work increased job satisfaction, performance, and feelings of commitment to an organization among its employees, who tended to experience less work stress or exhaustion. Since the COVID quarantine, however, there seems to be an increased sense of professional and social isolation, regardless of the virtual presence and engagement available via online platforms. Anecdotal reports from administrators and employees also suggest there is a greater blurring of boundaries between work and personal life, greater cognitive strain in the performance of their duties, and an increase in low-level anxiety and depression.

Description: This paper will examine what is known in the literature and provides insights from the field by two senior librarians, both in administrative and leadership roles at their institutions. In addition, the paper will suggest recommendations to address specific issues in managing libraries during the pandemic and offer some post-pandemic and long-term frames to effectively address remote now and hybrid work possibly in the future.

Conclusion: In the face of certain challenges and on-going risks of the pandemic, library directors and senior administrators are rightly concerned about how their institutions will continue to be affected and what the next steps may be. It is estimated that 42 percent of the U.S. labor force now works from home full-time. With second and possibly third waves of the COVID pandemic forecasted, it is inevitable that remote work will remain an important part of our libraries’ services, support, and management. Understanding best practices in managing remote workers and the caveats are critical to ensure the resilience of our libraries now and in the future.

Meeting Needs and Building Institutional Knowledge through Diversifying Research Workshops

Practice Area: Education

Stephanie M. Henderson, Nursing Liaison Librarian, University of Kentucky Medical Center Library, Lexington, Kentucky

Lauren Elizabeth Robinson, Medicine Liaison Librarian, University of Kentucky Medical Center Library, Nicholasville, Kentucky

Background: The needs of the students, faculty, and researchers that our library serves, has evolved over the past four years. It has become increasingly common for our patrons to ask more complex questions that. often call for repeated consultations. In the same time period, our library lost four librarian positions. While we have been understaffed, the colleges our library serves continue to grow, and user requests are more time and labor-intensive. To meet their needs, a virtual Lunch & Learn Research Workshop Series was developed. The program objectives were to teach frequently requested topics and to create recordings to share.

Description: To determine which workshops to offer, we looked at and discussed the questions we received over the past few years. Systematic Reviews, EndNote, and PubMed were the most requested topics. We also included workshops that introduced new library users to our resources and specific databases. Initially, the workshop series was going to be held in our Zoom equipped conference room so that we could record the workshops. Due to the COVID-19 pandemic, we had to adjust our plan and offer the series completely via Zoom. We created a website for the workshop series which included the registration link, recording links, and handouts from the workshops. Participants who registered for the sessions were sent calendar invites as well as the link to the recording of the session.

Conclusion: Our virtual workshop series is in progress. An evaluation form was created to assess the program. The outcomes we are expecting to measure are: did it meet their expectations, did participating learn something new, and would they recommend the workshops to others. We have received anecdotal positive feedback and plan to continue the workshop series for Spring 2021.

NNLM Course Development: Building Reference Skills for Nursing and Allied Health Research

Practice Area: Education

Margot G. Malachowski, AHIP, Education & Outreach Coordinator, Network of the National Library of Medicine, New England Region, Worcester, Massachusetts

Katherine B. Majewski, Librarian, Office of Engagement and Training, U.S. National Library of Medicine, Bethesda, Maryland

Emily Hamstra, Outreach Coordinator, Network of the National Library of Medicine, Pacific Northwest Region, Seattle, Washington

Elizabeth Waltman, Outreach, Education and Communications Coordiantor, Network of the National Library of Medicine, Southeastern Atlantic Region

Molly Knapp, AHIP, Training Development Manager, NNLM, University of Utah Eccles Health Sciences Library, Texas

Margie M. Sheppard, Community Engagement Coordinator, Network of the National Library of Medicine - Region 3, Olathe, Kansas

Background: In 2019, the Network of the National Library of Medicine (NNLM) embarked on a process to update a course that aimed to 1) teach participants how to evaluate quality health websites for nurses; 2) enable participants to describe and evaluate quality nursing resources available on the Internet; and 3) demonstrate searches for nursing literature in PubMed. The curriculum development team further refined these objectives. The new course would enable participants to connect National Library of Medicine resources to the needs of nursing and

allied health professionals. After taking the course, participants would be able to list the ways librarians can support the information needs of their nursing and allied health audiences.

Description: The NNLM curriculum development team met from November 2019 through December 2020 to develop a course that would be launched in early 2021. In the kick-off meeting, the team agreed to investigate needs and best practices for librarians teaching nursing and allied health professionals. Members of the team divided up course development tasks. They reconvened bimonthly to share relevant information resources, sharpen the learning objectives, and share ideas for course activities such as readings, quizzes, and written assignments. The result is a three-week asynchronous course that is designed for library staff who support, or want to support, nursing and allied health professionals. In week one, participants learn about the information needs of nursing and allied health professionals. Week Two introduces participants to relevant information resources. Week Three asks participants to apply learning through creating and sharing a professional development plan.

Conclusion: We gathered evaluation data after the pilot launch in December 2020 and three sessions taught in early 2021. We will address participant needs for better navigation and more time to complete the professional development plan. We hope to offer this course in Summer 2021.

Navigating Institutional Research Information Management Priorities: Rolling out of an Institutional Data Management Plan (DMP) Requirement

Practice Area: Information Management

Elizabeth C. Whipple, AHIP, Assistant Director of Research & Translational Sciences, Ruth Lilly Medical Library, Indianapolis, Indiana

Gabriel R. Rios, Library Director, Ruth Lilly Medical Library, Indianapolis, Indiana

Background: Our School of Medicine’s Research Administration identified several priorities related to Information Management, with one of them being the implementation of required Data Management Plans (DMPs). The impetus for the requirement was risk mitigation and preparation for a forthcoming NIH requirement. Our role has been to lead the process for the entire medical school including the communication, progress, and guidance. We completed the first round of DMP collection prior to the release of the NIH Policy on Data Sharing and Management. We will share our outcomes and how the NIH policy informed the development of our new DMP collection tool.

Description: Working with an Advisory Board, we designed a PI-based DMP collection tool vs. an award/project-based DMP. The library took the lead developing the DMP questions, tracking for compliance, creating FAQs, designing the communications to PIs and directing outreach to PIs in departments to move the initiative forward. The rollout of the DMP initiative

started in mid-2019, with the deadline for DMP completion initially slated for March 2020. In mid-2020, we started planning for the second iteration of the Qualtrics DMP form. Using a university-developed tool, we worked with the university technology development team to identify possible APIs to improve the form and reached out to faculty and staff stakeholders. This second iteration will be rolled out late 2020.

Conclusion: The new iteration of the DMP will speed up the time it takes to complete a DMP, better integrate into departmental annual reviews as a metric, and prepare our PIs to meet the new NIH Policy on Data Sharing and Management. The library’s leadership continues to showcase our value to our institution and move strategic priorities forward for our School of Medicine.

One Health COVID-19 Education for Pet Owners in Marginalized Communities during the Pandemic

Practice Area: Global Health & Health Equity

Michael Moore, Grants & Special Projects Librarian, University of Washington Health Sciences Library, Seattle, Washington

Electra Enslow, Director, Clinical Research and Data Services, University of Washington Health Sciences Library

Background: This project identified and assembled educational materials about COVID-19 and animal health subjects, adapted currently existing English-only health information resources focused on pet care during COVID-19 into other languages, and used a peer education model to distribute these materials in underserved communities in the Pacific Northwest.

Description: The Health Sciences Library partnered with the Center for One Health Research to provide COVID-19 health information outreach to marginalized communities focused on caring for pets and companion animals during the global pandemic. Outreach initiatives focused on two underserved populations: homeless and non-English speaking communities. Marginalized communities including individuals experiencing homelessness and ethnic minorities are at increased risk of contracting COVID-19 and have less access to education surrounding their own COVID risks and the risk to their pets, due to language and cultural barriers and the lack of appropriate educational materials. Additionally, this community is less likely to access veterinary care due to prohibitive costs, language barriers, and transportation issues. To fill that health information gap, the project disseminated these needs to communities in the Pacific Northwest. This project was funded with a COVID-19 Health Information Outreach Award.

Conclusion:

- Objective: Create educational materials accessible to marginalized English-speaking communities
  - Process Measures: Materials created; number of meeting to pilot drafts
  - Output/Data Tool: Flyers, brochures, web page information, pdfs for download
- Objective: Translate materials for non-English Speaking Communities
  - Process Measures: Number of translations of materials
  - Output/Data Tool: Flyers, brochures, pdfs for download in 7 languages
- Objective: Train peer educators
  - Process Measures: Number of peer educators trained; pre/post training surveys
  - Output/Data Tool: Peer-educators; number of training session; results of pre/post survey
- Objective: Peer educator outreach to the homeless and non-English Speaking Communities
  - Process Measures: Number of interactions; number of flyers disseminated
  - Output/Data Tool: Outreach records
- Objective: Free materials available for download
  - Process Measures: Number of sites hosting materials
  - Output/Data Tool: Website lists

Online Program-Based Library Orientations for Multi-Institutional Health Sciences Educational Partnerships

Practice Area: Education

Sandra J. Weingart, Animal Health and Agricultural Sciences Librarian, Utah State University, Logan, Utah

Suzanne Fricke, AHIP, Animal Health Sciences Librarian, Animal Health Library/Washington State University Libraries, Pullman, Washington

Background: Academic health science partnerships between institutions promote career opportunities, train a workforce in underserved regions, and allow institutional partners to build expertise without commitment to a full clinical program. Such programs are growing in number. Students receive pre-clinical education at their home institutions before joining the larger cohort for clinical instruction.

Students in such programs move between institutional library and information systems throughout their education and careers. The shift to online learning because of Covid-19 enabled librarians at two partner institutions to pivot from campus-based to collaborative, online, program-based library orientations for incoming veterinary medicine students.

Description: Six synchronous one-hour Zoom video conference sessions were offered to 139 incoming students in August 2020. All students have library access at the primary partnering

institution, while smaller cohorts also have access to their home institution library. Librarians provided pre-session written introductions via library guides and tutorials a month in advance of the orientation. Sessions were limited to 25 students, with one break out room interactive exercise mid-session. Librarians covered on- and off-campus access options, types of resources including ebooks and databases, new tools for browsing current journals, browser extensions for accessing pdfs, and reference management software. Students compared and contrasted resources across institutions and increased their awareness of the broader information landscape required for professional practice. Librarians provided written feedback on in-class submissions and students completed post-session evaluations.

Conclusion: All incoming Doctor of Veterinary Medicine students registered, 128 attended via Zoom, 11 had scheduling conflicts and were provided a recording of the session. Attendees submitted a reference management assignment for evaluation. Student feedback confirms enthusiasm for learning about information resources prior to the start of formal classes. Librarians providing online instruction in multi-institutional health science programs should contrast and compare resources and systems across institutions and focus on skills that transfer into new information environments and clinical practice.

Pain Points and Barriers to Clinical Information Access: Results from MLA InSight Initiative Summits 4 and 5

Practice Area: Information Services

Elizabeth Laera, AHIP, Medical Librarian, Brookwood Baptist Health, Birmingham, Alabama

Angela Spencer, AHIP, Health Sciences Reference Librarian, Health Sciences Library - Saint Louis University, Saint louis, Missouri

Charlotte Beyer, AHIP, Library Director, Rosalind Franklin University of Medicine and Science, North Chicago, Illinois

Jean Song, AHIP, Associate Director, Taubman Health Sciences Library / University of MI, Ann Arbor, Michigan

Karen Gutzman, Head, Research Assessment and Communications, Galter Health Sciences Library & Learning Center, Illinois

Background: The Medical Library Association’s InSight Initiative Summits provide an open and collaborative environment for library and industry partners to discuss vexing problems and find solutions to better serve their users. At the end of Summit 3, participants determined that one of the most pressing problems currently faced is to better understand how users discover and access information. Summits 4 and 5 saw a group of participants establish a framework for understanding end user pain points, or the barriers users encounter while attempting to access information resources, focusing specifically on pain points encountered in and around the clinical environment.

Description: Pain points are defined as problems that limit a user's ability to access information. The working group, librarians employed in both hospital and academic settings as well as vendors from various industry partners, identified eight pain points specifically experienced by users in clinical environments: time, awareness, access, paywalls, resource platforms, resource scope, integration, and financial limitations. These points were developed over a series of conversations within the working group as well as an environmental scan. An End User Advisory Board (EUAB) composed of physicians, clinical researchers, and clinical faculty in biomedicine was established to share thoughts on accessing and using information resources, which helped the group refine the pain points. The EUAB later provided feedback on the final pain points and imagined how potential solutions could affect workflow.

Conclusion: Identifying potential solutions and exploring what solutions are currently in the works was a main goal of the working group. The EUAB clarified that time is the most pressing pain point and solutions in this area would be the most impactful. According to the working group’s research, most of the ongoing solutions lie in the area of access. However, the EUAB elucidated that these solutions would not be as impactful on their workflow as improvements in other areas such as paywalls and integration. The working group calls on librarians and industry partners to further explore end user pain points and focus on solving the issues that will make the most impact on the end user experience.

Petting Zoo of Learning Objects: A Virtual, Self-Paced Professional Development Program for Employees at an Academic Library

Practice Area: Education

Erica R. Brody, Research and Education Librarian for School of Dentistry, Health Sciences Library, Richmond, Virginia

Hope Y. Kelly, Online Learning Librarian, Virginia Commonwealth University Libraries, Mechanicsville, Virginia

Background: Library staff developed asynchronous instructional content for the largely online environment of higher education in 2020 due to the COVID-19 pandemic. At Virginia Commonwealth University, a variety of techniques and technologies were used to deliver information ranging from orientations to information literacy instruction. The resulting collection of learning objects presented a unique opportunity to create a virtual professional development experience for all library employees. By reviewing this body of instructional material from various library units, participants could learn different ways to present asynchronous instruction and potentially improve upon their teaching techniques and products.

Description: We employed the Canvas learning management system to develop a digital “petting zoo.” Fifteen learning objects and descriptions were collected from eleven librarians using Google Drive between October 20 and December 2, 2020. The authors organized

content into a series of webpages, each represented by a zoo animal to make the module navigable and fun. The program was promoted via blog posts and departmental announcements.

Starting on December 7, twenty-seven employees enrolled in the petting zoo Canvas course and viewed an average of 33 pages. Nine learning object authors each hosted a live one-hour Zoom Q&A session at different times during a weeklong period. Attendance at each session varied with a maximum of seven participants and two sessions with no attendees. Participants submitted a total of 15 comments on discussion threads dedicated to each object. Our evaluation revealed that participation was greatest among employees for whom instruction is a significant part of their work life. Overall, participant feedback was positive, providing inspiration for participants’ future instruction efforts.

Conclusion: Modeled after the MLA 2020 vConference, the “petting zoo” provided an opportunity for library staff to review asynchronous instructional content created by peers to increase their knowledge of developing engaging learning objects, potentially improving the quality of future programming. The largely asynchronous nature of this professional development program was designed to maximize convenience, and thus participation. The findings will inform the continued development of a community of practice focused on instructional design and educational technology applications within VCU Libraries. Further, other academic libraries may adapt this format to create asynchronous professional development programs for their employees.

Planning, creating, delivering, and evaluating an informatics course for health sciences students

Practice Area: Education

Mê-Linh Lê, AHIP, Health Sciences Librarian, University of Manitoba, Winnipeg, Canada

Background: Due to changes in the scope and nature of care provided by pharmacists, all accredited Canadian Pharmacy programs were required to transition from a 4-year BSc to a 5-year PharmD by 2020. At the University of Manitoba the PharmD program launched in Fall 2019 and included the creation of a new 1-CR Pharmacy Informatics course. In late 2018, the Pharmacy liaison librarian was asked to be part of the planning team and soon was tasked to take the lead role. This presentation will present the challenges and opportunities associated with taking on an expanded role of this nature.

Description: During the nine-month planning process, the liaison librarian took a leading role in outlining the entire structure of the course, writing the syllabus, mapping out content and objectives to competencies and standards, creating all assessments, delivering the majority of the content, and marking and providing feedback. This session will also delve into the practical aspects of being an instructor that are not often discussed by librarians, such as figuring out honorifics, convincing students that not everything can be evaluated via a multiple-choice

question, how presentation styles and tone change when moving from a librarian to an instructor role, dealing with academic integrity issues in a professional program, and how to avoid teaching without being distracted by the thought of student evaluations.

Conclusion: Assessment of the initial course was done through two means: student evaluations and faculty feedback. Student evaluations were used to better understand their perspective on the quality of the course and modify future offerings. Faculty feedback has been incredibly positive; the librarian has developed a much stronger relationship with the college with hours of instruction to the college increasing from 2 hours to 10, has been invited to participate in future course design, and taught the course again in 2021. Ultimately, this presentation will show another way in which librarians can demonstrate their value to administrators, which can be accomplished by broadening the instructional roles that liaisons play.

Practice Gives Confidence: Preparing Medical Students to Present Evidence in Clerkships

Practice Area: Education

Amy Christine Studer, AHIP, Health Sciences Librarian, Blaisdell Medical Library, University of California, Davis, Sacramento, California

Nicole Capdarest-Arest, AHIP, Head, Blaisdell Medical Library, University of California, Davis, Sacramento, California

Background: Our Transition to Clerkship (T2C) course occurs immediately prior to clerkship rotations and includes an evidence-based medicine (EBM) module. T2C had been synchronous and in-person for all Year 3 medical students, but due to COVID-19, content needed to be transformed to be delivered virtually. Librarian instructors of the EBM module therefore re-designed content to increase emphasis on the primary deliverable: a practice oral evidence presentation. Content was revised to be asynchronous and to further develop learner skills in finding evidence, synthesizing that evidence, and then supportively practicing the new skill of presenting an oral evidence synthesis for a clinical setting.

Description: We re-designed content driven by learning objectives mapped to our school of medicine competencies and AAMC Core Entrustable Professional Activities. With primarily asynchronous delivery, we provided learners with frameworks and instructions to support their success with the activities, which were designed to be accomplished in 2 - 3 hours. The module consisted of pre-work and a paired activity. The pre-work focused on context for clinical oral evidence syntheses and on finding quality evidence. The paired activity consisted of selecting from a menu of topics, finding a relevant guideline and a primary study, synthesizing evidence found, recording and presenting a brief oral update via video to a peer, and providing peer feedback. We reviewed each student’s submitted video based on the peer

feedback rubric, as well as their feedback to their partner, and then provided aggregate feedback to the students.

Conclusion: In this revised format, learners deliberately practice an oral evidence presentation in a safe environment assuring supported practice before moving into the clinical environment. Initial feedback shows the instruction was effective (most rating it “very effective” or higher) and that students appreciated the module structure, learning how to find guidelines, and the opportunity to practice presenting. Feedback included comments such as, “I loved the EBM project and found it especially useful to 3rd year.” We were impressed by the high level of engagement that students demonstrated in this module, as compared with prior years’ in-person sessions. In the future, we hope to gather impact measures from learners as they progress through clerkship rotations and integrate further goal-directed practice.

Providing Evidence about the Pandemic: Librarian Roles on a Rapid Response Team for COVID-19

Practice Area: Information Services

Hannah J. Craven, Research & Scholarly Communications Librarian, Indiana University School of Medicine, Indianapolis, Indiana

Rachel Hinrichs, AHIP, Health Sciences Librarian, IUPUI, Indianapolis, Indiana

Caitlin Ann Pike, AHIP, Research Engagement Coordinator, IUPUI University Library, Indianapolis, Indiana

Amy Blevins, MALS, Associate Director for Public Services, Ruth Lilly Medical Library, Indianapolis, Indiana

Background: SARS-CoV-2 has led to a deluge of information. Health leaders/personnel need curated and synthesized information to aid their decision-making regarding diagnosis, treatment, mitigation, reopening plans, etc. A collaboration involving the state department of health has been developed to respond to those needs. Several programs were created simultaneously, two of which depend on active librarian involvement. One focuses on rapid expert responses to questions from state leadership supported by annotated bibliographies. The other is a daily digest of emerging literature including reviews on patient care, law and ethics, communication, forecasting and surveillance, schools, and mitigation.

Description: Librarians from several libraries at a university are responding to the need for synthesized high-quality information related to the pandemic. These librarians have expertise in the areas of evidence-based medicine, data management, public health, and law. From the beginning, librarians have been heavily involved in creating workflows related to project and data management. Project management included having a standard process for tracking questions, delivering information, and team development. Librarians developed the team through onboarding, defining roles, scheduling, consistent communication, and shared

document templates. Librarians created data management processes such as citation management, readme files, file management/naming conventions, data backup, etc. Librarians encouraged adoption of creative commons license notices on public-facing work to make the information more accessible. Project assessment was built into workflows and includes tracking: questions, turnaround time, updates, and return on investment.

Conclusion: Results will be included if the abstract is accepted.

Public Health Programming to Transform Library Visibility and Campus Community Interaction

Practice Area: Information Services

Kathryn M. Houk, AHIP, Health Literacy & Community Engagement Librarian, University of Nevada Las Vegas, Las Vegas, Nevada

Maggie Bukowski, Library Technician II, UNLV University Libraries, Health Sciences Library

Background: Employees at the University of Nevada, Las Vegas Health Sciences Library (UNLV HSL) found themselves struggling to reduce transactional interactions and increase community between library staff and patrons within their spaces. Reduced opportunities for face-to-face interactions during COVID-19 sparked the development of a passive programming plan centered around national health observances. The awareness campaigns focus on creating messaging that patrons interact with individually. The objectives are to market the UNLV HSL and its resources, increase awareness on public health topics across the health sciences and undergraduate campuses, and build opportunities for conversation and exploration between patrons and staff.

Description: This presentation will focus on one HSL’s approach to planning passive programming, why this style of programming best fits our community, and how the continuing pandemic affected plans. The program designers were inspired by monthly heritage month programming the HSL has already been producing in partnership with the School of Medicine. Heritage programming includes physical posters and slides on visual displays, and we built upon this idea to create programming around health observance months. Library staff creates an array of informational objects including table tents, visual slides, social media posts, and buttons. These objects highlight health and heritage observances as well as interesting health facts, and patrons are able to choose their level of engagement with the information provided. In our discussion, we will address challenges, limitations, and observations from this project as well as our next steps.

Conclusion: A survey of library patrons revealed table tents and buttons were the most widely-seen promotion efforts. Feedback included a desire for going beyond simple health facts and statistics to include more humanistic elements such as ways to build empathy for those living with disease or illness. New promotional channels will be explored, as well as increasing access to passive activities that promote wellness and fun.

Redesigning Library Orientation for First-Year Medical Students during the Pandemic

Practice Area: Education

Andy Hickner, Education and Outreach Librarian, Weill Cornell Medicine, Astoria, New York

Loretta Merlo, Manager Circulation Services, Weill Cornell Medicine, New York, New York

Drew Wright, Scholarly Communications Librarian, Weill Cornell Medical Library, New York City, New York

Background: Before 2020, the Wood Library at Weill Cornell Medicine conducted first-year medical student (M1) orientation as a "Treasure Hunt.” a The goal of this team competition activity was to inform students about library services, policies, and space, and introduce them to staff, in a way that was fun for them. Each year, students typically reported the Treasure Hunt was the most enjoyable orientation session. Due to the COVID-19 pandemic, the orientation for the MD class of 2024 was shifted to an all-virtual format, which mandated a full redesign of the Library orientation.

Description: The Samuel J. Wood Library sought to preserve the excitement and fun of the treasure hunt in the new virtual format. The competition was redesigned as a Zoom meeting using breakout rooms, with library faculty and staff serving as team facilitators. Tasks were rewritten, shifting the focus from the library’s physical spaces to its virtual services and online resources. The redesigned orientation was evaluated using two data sources: a post-session survey of student participants and a debriefing of the library employees who participated. Student evaluations were positive, while the faculty and staff provided numerous suggestions for improving future virtual orientations. We evaluated the redesigned orientation through by debriefing the Library employees who served as small group facilitators and by administering an online survey of the students about their experience.

Conclusion: A successful virtual library orientation requires careful preparation, including testing the competition tasks, full rehearsal with library facilitators, and a thoughtful approach to technology and logistics. We have chosen to share the materials we developed for other academic health sciences libraries that may wish to take a similar approach to their own virtual orientations.

Revamping Publicity and Promotion Strategies to Engage and Connect in Time of a Pandemic

Practice Area: Professionalism & Leadership

Lin Wu, AHIP, Assistant Director for Research & Learning Services, University of Tennessee Health Science Center Health Sciences Library, Memphis, Tennessee

Sarah Thompson, Communications Coordinator, UTHSC Health Sciences Library

Jennifer M. Langford, Archivist and Special Collections Librarian, UTHSC Health Sciences Library/Health Sciences Historical Collections, Memphis, Tennessee

Kay Strahan, Research and Learning Services Librarian, University of Tennessee Health Science Center

Background: Publicity is about getting the message out via different outlets, while promotion is about encouraging people to use the services and resources they hear about. Both are part of marketing strategies. Marketing library services and resources has been an on-going effort for an academic health science center library. When the entire campus was quickly turned into a virtual working and learning environment due to the coronavirus pandemic, marketing strategies became even more vital and challenging. The study aims to describe the library’s efforts in restructuring marketing strategies to reach out and engage its users in the virtual environment.

Description: In response to the abrupt shift to remote learning and working, the library was faced with the dilemma of how to effectively get the messages out and maintain a meaningful connection with the campus community. There were no formal policies or procedures on marketing the library. In March 2020, the Communications Coordinator was hired to lead the marketing efforts. The library marketing strategies were assessed and implemented. Some of the strategies included re-planning digital advertising, categorizing marketing content, featuring librarians and resources, and repurposing or creating resource guides. Digital outlets such as the campus Daily Digest, library website, and social media platforms are used to implement the change. Did our efforts make a difference? Ten-month usage statistics on library resources and services were collected and examined to see the impact.

Results: Statistics showed some overall increases in library resource guide visits, library workshop attendance, and information services. We had some increases in followers on our social media platforms (e.g., 44% for Instagram, 25% on Twitter, and 56% increase on Facebook). With the decrease in reach we gained a substantial increase in engagements, which indicated that people stopped scrolling to see our posts and that possibly brought more awareness to our content.

Conclusion: The virtual environment opens up the opportunity to connect with our patrons and learn about their needs beyond the physical building. Our efforts in publicity and promotion have helped enhance library's visibility and improve awareness of the library resources and services. The experience taught us it was effective to use short teasers with links to point patrons to vital resources and services. More posts didn't necessarily mean more engagements. The process of thinking, planning, publicizing, and assessing is the key in marketing the library successfully. We're in the process of creating more videos and Instagram stories to attract more users to our resources and services.

Shape Shifters Wanted: Transforming Health Sciences Librarian Careers between Hospitals and Academia

Practice Area: Professionalism & Leadership

Jenessa Marie McElfresh, AHIP, Health Sciences Librarian, Clemson University, Clemson, South Carolina

Rachel Keiko Stark, AHIP, Health Sciences Librarian, California State University, Sacramento, Sacramento, California

Background: The purpose of this paper presentation is to explore health sciences librarian competencies, training, and job expectations for individuals whose career journeys have included shifts to and from hospital and academic settings. Though united in the field, different health sciences librarian roles demand varying areas of expertise. Managing these transformations requires flexibility and an adaptable skill set that all health sciences librarians can benefit from developing. This session is geared to new and experienced professionals from all organization types.

Description: This program utilizes a multi-case study approach highlighting the professional competencies necessary in both academic and hospital settings, the expertise needed that is unique to these librarian roles, and examples of the skill sets implemented by librarians who have been successful in transforming their careers to become hospital and academic health sciences librarians. Discussion about the importance of a strong CV, targeted cover letter, and various other documentation required during the application process will be presented. The differences between interview styles for hospitals and academia will also be explored, as well as expectations for service. Variations in workplace expectations and institutional cultures will be emphasized and evaluated throughout the presentation. Power dynamics, with an emphasis on socio-cultural structures and experiences of individuals from historically marginalized groups, will be examined particularly as they apply to health sciences and hospital librarians.

Conclusion: This multi-case study will present attendees with examples of career journeys that incorporate transformations to and from hospital and academic library roles. The multi-institutional success of the individuals framed in these case studies stands as evidence of the efficacy of the strategies presented throughout the paper presentation. Case study experiences that involve transition challenges and unexpected barriers will also be incorporated as conclusive knowledge. Attendees can expect to walk away with an understanding of the ways that different health sciences roles and organization types intersect and allow the nimble health sciences librarian to craft a successful career in all worlds. To supplement the material, references, recorded webinars, and other training documentation will be incorporated for attendee use

Stories of Illness during Epidemics: Library Role in Preserving Illness Narratives

Practice Area: Global Health & Health Equity

Nina Stoyan-Rosenzweig, Senior Associate in Libraries, University of Florida, Gainesville, Florida

Background: This presentation describes library involvement in collecting, recording, preserving, and publishing historical records of illness experiences during epidemics- in this case, experiences of illness during polio epidemics in the United States during the 1940s and 1950s. By reading and studying these narratives, society learns and relearns how people respond to medical sides of illness as well as the uncertainty of fear and social change. In particular it examines how telling these stories of illness helps the ill redefine who they are in relation to the illness- and we'll see how it helps families rebuild their lives in face of tragedy.

Description: This presentation describes a program that collects and preserves narratives of illness to document the experience of illness, as well as societal responses to epidemics. The program involves collecting oral histories- working with post-polio groups to identify people interested in participating in the oral history process and finding ways to highlight and publish written stories of illness from polio survivors. Recorded stories will be recorded- videotaped in some cases, and all transcribed and made available through library-maintained sites and working with a university press to publish a particularly unusual, one of a kind narrative.

Conclusion: This presentation will describe the program collecting polio illness narratives and preparing them for use in teaching, while making them available, through library-maintained sites, for research and outreach. The presentation also will spotlight efforts to publicize and encourage use of this resource.

Supporting Consensus Statements: Not Your Average Systematic Review

Practice Area: Information Services

Michelle Demetres, Scholarly Communications Librarian, Weill Cornell Medical College, New York, New York

Drew Wright, Scholarly Communications Librarian, Weill Cornell Medical Library, New York City, New York

Diana Delgado, AHIP, Associate Director, Information, Education & Clinical Services, Samuel J. Wood Library, New York, New York

Background: Literature has shown the importance of involving a librarian in evidence-based literature searches such as systematic reviews (SR). Increasingly, a critical eye has been placed on the methodological quality of consensus statements (CS). As evidenced-based searchers, librarians that support SRs are often called on to support CS work. However, despite similar methodology, there are important distinctions and key considerations that should be taken into account before supporting CS work.

Description: Using Weill Cornell Medicine's Systematic Review Service experience as a guide, the aim of this paper is to answer three questions: What is a consensus statement? How does this compare to other evidence-based methodologies? What are practical points to consider when supporting CS?

A CS is similar to a practice guideline, but its recommendations are developed based on collective opinion or consensus of the convened expert panel. Often a CS is done when a rigorous practice guideline cannot be performed due to lack of high-quality evidence. There are many important considerations of which a librarian team should be aware before taking on this work, including: (1) librarian as project manager; (2) multiple searches/reviews for one CS; (3) multi-institutional/international teams; (4) scalability with staff time/library assistant help.; (5) authorship; (6) access/troubleshooting for a SR support tool; (7) full text, copyright, Interlibrary Loan.

Conclusion: Despite differences from a practice guideline, a CS should still be evidence-based and center around a SR. Supporting CS work is an important avenue for librarians to help improve methodological quality and ultimately impact clinical care. However, before embarking on this kind of support, it's important for librarians to be familiar with the study type, its differences from other evidence-based recommendations, and the nuances involved in working with CS teams. Our experiences will help other libraries adequately prepare for or promote this novel service.

Thinking outside of the E-Book Box: Crafting Custom License Addenda for Maximal Use of Images in Health Sciences Curricula

Practice Area: Information Management

Susan K. Kendall, AHIP, Health Sciences Coordinator, Michigan State University Libraries, East Lansing, Michigan

Iris Kovar-Gough, AHIP, Health Sciences Librarian, Michigan State University Libraries, East Lansing, Michigan

Background: Our university libraries support several health colleges with image-rich online textbook resources. These images are in demand by instructors in the health professions programs. When developing course materials, instructors were asking questions about image use and copyright. In some cases, they were paying permission fees and passing the cost on to students. We knew that use was governed by licenses instead and found that the standard

license agreements and terms of use were confusing, varied by publisher, and did not reflect today’s educational needs. We negotiated license addenda to several products that make transformational use possible for our educators.

Description: We needed terms of use that clearly allowed instructors to embed images from library-subscribed sources into course materials and websites. Starting in 2012, we negotiated license addenda with each major vendor’s legal department one by one. We pointed out problematic language in the existing licenses and crafted plain language to ensure library staff at all levels of experience could understand. We created and promoted a guide to direct course content creators to approved sources for images. We assessed the success of the program in several ways. We tracked the vendors willing to negotiate, the usage of relevant e-books packages from 2014-2019, the costs of course materials, and faculty feedback.

Conclusion: Our efforts were successful in meeting these goals. Instructors in the health professions and related fields have praised our guide and have chosen to primarily embed library-subscribed content into course materials. Usage of relevant e-book packages increased by 50-500% between 2014 and 2019. Costs of course materials decreased. Our efforts demonstrate the willingness of major publishers to negotiate and to update their understanding of online education pedagogy and allow use often prohibited by standard agreements and terms of use. Because contracts supersede copyright and fair use, advocating for and empowering librarians to craft custom usage addenda that meet the unique needs of the institution's faculty and mission is essential to supporting 21st century educational needs.

Transformation through Collaboration: A Partnership in Diversity Initiatives and Programming

Practice Area: Professionalism & Leadership

Natasha Williams, AHIP, User Services Librarian, University of Central Florida College of Medicine, Orlando, Florida

Shalu Gillum, AHIP, Head of Public Services, University of Central Florida College of Medicine, Orlando, Florida

Heather R. McClellan, Assistant Director, Office of Diversity Equity and Inclusion, saint cloud, Florida

Ethel M. Smith, Diversity Programs Coordinator, Office of Diversity Equity and Inclusion, Orlando, Florida

Saleh M. Rahman, Assistant Dean for Diversity and Inclusion (Interim), University of Central Florida College of Medicine, Florida

Background: The College of Medicine Office of Diversity, Equity, and Inclusion (ODEI) reached out to the library to pursue a collaborative effort to build a “diversity corner” for the

college that could highlight diversity in healthcare. It was determined that a library guide would best serve this purpose. Two projects emerged from this collaboration: the creation of a Diversity, Equity, and Inclusion Library Guide, and the launch of a virtual Human Library Project collection of stories.

Description: The library guide showcases literature, research, and other content available through the Health Sciences Library’s licensed resources related to diversity in healthcare. ODEI provided guidance on the content, and a member of the library team constructed the guide.

The Human Library Project initiative was conceived by ODEI and needed a programming platform to kick-off its creation as well as a permanent virtual home. The Health Sciences Library, ODEI, and members of the college’s Council for Diversity and Inclusion worked together to arrange speakers, create personal narratives, and host a lunch and learn to introduce the concept to the college during Diversity Week. The narratives were then added to the library guide.

The success of the library guide and Human Library Project will be evaluated using website analytics and process, impact, and outcomes measures.

Conclusion: The project team will continue to build upon the library guide’s content to provide a comprehensive tool that the College of Medicine community can leverage in their pursuit to understand and embrace diversity in medicine and healthcare. College faculty, staff, and students may use these resources to assist with research and course curriculum, and in challenging racism, societal stigmas, stereotypes, and bigotry.

Transforming Practice through an Innovative Training Model: MLA Research Training Institute

Practice Area: Innovation & Research Practice

Jodi L. Philbrick, AHIP, Senior Lecturer, University of North Texas, Denton, Texas

Lorie A. Kloda, Associate University Librarian, Library, Montreal, Quebec Canada

Susan Lessick, AHIP, FMLA, RTI Project Director/Librarian Emerita, University of California, Irvine, Anaheim, California

Emily Vardell, AHIP, Assistant Professor, Emporia State University School of Library and Information Management, Emporia, Kansas

Background: The MLA Research Training Institute (RTI) is a unique training program funded by the Institute of Museum and Library Services (IMLS) to equip practicing health sciences librarians with advanced research competencies related to scholarly research, inquiry, and publishing. It includes an innovative combination of a one-week residential workshop in

advanced research methods, a real-world opportunity to develop and conduct a research project, and a full year of post-workshop mentoring. This paper will focus on program outcomes related to participating librarians’ research knowledge and engagement from the first two institutes held July 2018-June 2019 and July 2019-June 2020.

Description: This paper will compare RTI participants’ perceived confidence with research skills before, after, and one year later after the residential workshop, comparing survey results for two subsequent years. The instrument used for the pre- and post-workshop assessment is based on methods of Brancolini and Kennedy (2017) and adapted for use for the RTI training model. The Wilcoxon Signed Ranks Test was performed to determine if there were statistically significant differences in the self-reported research confidence of the participants before and after each workshop. The non-parametric Friedman’s test of differences was also used to evaluate the differences in median research confidence levels of RTI Fellows across three different time points. Findings from workshop and program evaluations, as well as Fellows’ research progress, outputs, and impacts will be summarized. Additionally, the authors will share informal feedback on participants’ research competencies and engagement.

Conclusion: Assessment findings demonstrate that the RTI learning model is sound and effective and increases research competencies, confidence, and productivity of participants. Data shows there were statistically significant differences in self-reported research confidence of both the 2018 and 2019 cohorts across all 26 research skills assessed (except one). In another data collection activity, 95% of 2018 participants and 100% of 2019 participants reported that their understanding of research had increased as a result of the RTI program, and 95% of both groups were confident in applying what they learned in the RTI program.

Transforming REDCap Trainings for COVID-19 Emergency Distance Learning

Practice Area: Education

Fred Willie Zametkin LaPolla, Research and Data Librarian, Lead Data Education, NYU Langone Medical Center, BROOKLYN, New York

Background: The survey capabilities and electronic consent (eConsent) functionality of REDCap became critical when COVID-19 forced a shift to distance-based research. As researchers shifted to remote research, there was a dramatic increase in the training needs and questions about REDCap functionality. Our library sought to meet this need by providing both synchronous distance education and asynchronous resources for eConsent.

Description: To provide synchronous training, a librarian at our academic medical center updated formerly in-person training materials to provide an increased number of training workshops that each spanned a shorter time period. These were offered by Zoom to classes capped at 500 participants, rather than the in-person cap of 24. The live, Zoom-based training series was held every other week at the outset of pandemic. For asynchronous training, the

librarian consulted with the institutional IRB and IT to create online MP4 videos detailing the process of eConsent. These were updated iteratively to address issues the IRB was seeing in applications involving eConsent. The recordings are hosted on the library’s YouTube page.

Conclusion: Over 1100 individuals attended nineteen distance-based workshops, including an introductory session and classes on surveys, longitudinal studies, efficient use of REDCap and data import/export. This represents a vastly increased attendance compared to those possible in a physical classroom. Additionally, the eConsent videos have been viewed over 1000 times, reflecting a high need for such resources. The process of creating videos also strengthened connections between the library and IRB staff, which led to mutual referrals, an additional benefit to the research community.

Transforming the Teaching Landscape: Librarians Leading Online Course Design and Review

Practice Area: Education

Jessica Sender, AHIP, Liaison to the College of Nursing, Michigan State University, East Lansing, Michigan

Andrea C. Kepsel, AHIP, Health Sciences Educational Technology Librarian, Michigan State University, East Lansing, Michigan

Background: In response to the ongoing pandemic and the shift to online courses, librarians acted as facilitators for a campus-wide Quality Matters peer-review process to provide feedback and assistance to instructors as they developed online courses. After receiving Quality Matters training, librarians led instructors through a rigorous two-week course-review process, designed to improve the quality of online courses and instruction for the Fall 2020 semester. By serving as facilitators, librarians contributed their instructional design expertise and built partnerships with instructors across campus to improve course design, reduce barriers for students, and lead to better academic outcomes.

Description: With the move to exclusively online learning for the Fall 2020 semester, faculty were encouraged to participate in one of two professional development opportunities to develop their online course content and curriculum. Upon completion of the workshop and creation of their course, faculty participated in a hands on, in-depth course review process based on Quality Matters. Feedback from this program was largely positive, even with serious time constraints, heavy workloads, and ongoing pandemic fatigue. Faculty and educators indicated that it was helpful for their own teaching to review courses and contributed to bettering their own online courses. With over 600 participants, the campus-wide impact of this program is undeniable. The involvement of librarians in this program showed how integral librarians and the library resources are when transitioning courses from in-person or hybrid to entirely online.

Conclusion: There has been a deliberate shift by the university to elevate and assess online courses, especially as the pandemic and emergency orders wane, and the university looks to reinstate in person learning. Many faculty found success in their online course environments, and there is a renewed focus on online and hybrid courses as faculty move from a reactionary approach to deliberate instructional course design. The environment of assessment and evaluation for these online and hybrid courses, and the implementation of course quality reviews campus-wide, requires leadership from librarians within the peer-review online course review process. This program has also proven to instructional and educational technology partners on campus-including IT- that librarians have a strong and diversified background in instructional design and development, and are integral partners when developing robust online courses.

Unlocking Zoom’s Potential in Graduate Medical Education: Teaching and Adapting during COVID-19

Practice Area: Education

David Petersen, AHIP, Assistant Professor, Research & Learning Services Librarian, Preston Medical Library, University of Tennessee Graduate School of Medicine, Knoxville, Tennessee

Daphne M. Norwood, Associate Professor of Medicine, University of Tennessee Graduate School of Medicine - Knoxville, Knoxville, Tennessee

Anthony Wilson MD, Chair of Family Medicine, University of Tennessee Graduate School of Medicine

Alexandria Q. Wilson, Assistant Professor/Research & Learning Services Librarian, Preston Medical Library, University of Tennessee Graduate School of Medicine, Knoxville, Tennessee

James Michael Lindsay, AHIP, Head of Collections & Access Services, Preston Medical Library / University of Tennessee Graduate School of Medicine, Tennessee

Kelsey L. Grabeel, AHIP, Assistant Director, Preston Medical Library / Health Information Center, University of Tennessee Graduate School of Medicine / University of Tennessee Medical Center, Knoxville, Tennessee

Martha Earl, AHIP, Director/Associate Professor, Preston Medical Library, Knoville, Tennessee

Background: The Preston Medical Library partnered with the University of Tennessee Graduate School of Medicine (UTGSM) faculty and continuing education department to provide an online workshop on Zoom instructional techniques. Although the university had an institutional license, Zoom was seldom used before the COVID-19 pandemic. After the pandemic’s onset, UTGSM academic instruction was moved to a virtual or hybrid format. Faculty requested assistance explaining the variety of Zoom instructional tools along with personalized content focusing on access and use of Zoom. Researchers believe the Zoom workshop will increase faculty confidence and abilities to integrate Zoom techniques for interactive learning.

Description: A one question survey was sent out to UTGSM faculty asking what they would like to learn about Zoom. After analysis, researchers compiled a list of the most requested instructional components of Zoom. These components included topics like breakout rooms, how to co-host, and incorporating interactive learning techniques while also dealing with institution specific questions. An online synchronous workshop was developed to deliver the content while an adapted tutorial is available from the library’s website. A pre-test and post-test accompanied the synchronous workshop and the asynchronous tutorial to measure faculty confidence with utilizing Zoom for instruction.

Conclusion: Pre- and post-workshop surveys measured faculty comfort level with teaching on Zoom, familiarity with interactive learning, using integrative instructional techniques, ability to use select Zoom tools, and satisfaction with the workshop. Results indicated that faculty increased comfort levels with Zoom after participating in the workshop.

Using Comics to Talk about Librarian Experiences

Practice Area: Professionalism & Leadership

Ariel F. Pomputius, AHIP, Health Science Liaison Librarian, University of Florida, Gainesville, Florida

Background: Academic librarians face joys and stresses in their work, some of which are the usual joys and stresses that face anyone in a career and some unique to the field of librarianship. Speaking openly with colleagues about the highs and lows of librarianship can be difficult, particularly if librarians are new to the field. Creating comics, even for professionals unfamiliar with the medium, serves as a self-reflective exercise that can encourage open communication about difficult topics. Using a comic drawing exercise, this research project explored the stresses and joys of the work experiences of librarians.

Description: The comic-creating workshop was created for newer librarians in the early stages of their careers as academic librarians. Librarians participated in two comic-drawing exercises, one exploring what brings them joy and satisfaction in their work and the other outlining what causes them stress or pain at their job. After each comic exercise, the comics were used by the participants to discuss their work experiences and find differences and similarities in their joys and stresses. At the end of the workshop, participants talked about how they handle the highs and lows of their work experiences. The goal was to encourage participants to alleviate stress, celebrate their achievements, bond over shared experiences, and share advice over how to be more resilient.

Conclusion: The pilot version of this workshop was evaluated through a participant survey. Results of this pilot show that participants felt the comic-drawing format of the exercises was more conducive to discussing work experiences. Future versions of this workshop will work with a larger audience and involve an analysis of the comics created by participants to explore what causes librarians the most stress and joy.

Using Gamification to Teach Critical Appraisal Skills

Practice Area: Education

Charlotte Beyer, AHIP, Library Director, Rosalind Franklin University of Medicine and Science, North Chicago, Illinois

KatieRose McEneely, Electronic Resources Librarian, Rosalind Franklin University of Medicine and Science, North Chicago, Illinois

Background: In early spring 2020, faculty members of a medical school curriculum committee sought to improve students’ Evidence Based Medicine (EBM) skills and reached out to the library team to design a workshop which would teach students aspects of critical appraisal. Instead of teaching the concepts in the form of a lecture, the librarians use gamification to engage students while learning about how to effectively evaluate information. This mandatory workshop was embedded into an interprofessional clinical reasoning course with second year students from both medical and podiatry programs. The workshop focused on appraising randomized controlled trials (RCTs).

Description: The workshop was built around a card game and an additional trivia game with mini lectures to introduce the concepts. Students were divided into small groups of about eight students with one fourth year medical student (M4) as facilitator. The card game involved each group being assigned a RCT to review. Students selected a card from a deck with a question about blinding and randomization, and then answered the question on the card with a green card for yes or a red card for no. After the questions were answered, the M4 would lead a group discussion on the overall quality of the article. The cards provided a visual to guide the students in the discussion. The second part of the workshop was a trivia game around interpreting results. The group with the highest score, won gift cards for coffee.

Conclusion: To assess the workshop, students were given a paper survey with six questions. Out of 280 students, 207 filled out the post- workshop survey, and when asked if they preferred the content being presented in an interactive workshop format over a traditional lecture, 175 respondents selected strongly agree or agree. Librarians also had a conversation with the M4 group facilitators. Overall the M4s appreciated that the concepts were being covered as the content often comes up in rotations and boards. Students and facilitators also indicated that they preferred the card game to the trivia game due to the conversations it generated. Using gamification like this workshop can be one strategy to engage students in practicing their critical appraisal skills.

Workshop the Workshop: Continuous Quality Improvement Strategies for an Instruction Series

Practice Area: Education

Heather S. Healy, AHIP, Clinical Education Librarian, University of Iowa Libraries/Hardin Library for the Health Sciences, Iowa City, Iowa

Jennifer Deberg, User Services Librarian, Hardin Library for the Health Sciences, University of Iowa Libraries, Iowa CIty, Iowa

Riley J. Samuelson, Education & Outreach Librarian, Hardin Library for the Health Sciences, University of Iowa, Iowa City, Iowa

Matt R. Regan, Clinical Education Librarian, Hardin Library for the Health Sciences, Iowa City, Iowa

Damien Ihrig, Curator, John Martin Rare Book Room, University of Iowa Hardin Library for the Health Sciences, Iowa

Chris Childs, Clinical Education Librarian, , Iowa City, Iowa

Jessica L L Elliott, Administrative Services Coordinator, Hardin Library for the Health Sciences, University of Iowa, Iowa City, Iowa

Janna C. Lawrence, AHIP, Director, Hardin Library for the Health Sciences, University of Iowa, Iowa City, Iowa

Background: Like many health sciences libraries, Hardin Library since 2010 has offered a workshop series for database searching, citation management software, mobile apps, data management, and scholarly communication topics. Recently, instruction staff investigated and began to implement process improvement strategies to refine the overall delivery of the service. This paper outlines a team-based approach to enhancing the workshop program. Libraries with similar workshop programs can benefit from the examples presented of successes and challenges with marketing, branding, technology, and accessibility. Establishing an infrastructure with a team-oriented approach can produce a successful library instruction program with a focus on ongoing quality improvement.

Description: Staff have redesigned the program’s structure and logistics to effect a variety of improvements. Efforts to increase marketing include promoting the series in key campus communication outlets. Strategies to improve convenience include calendar appointments, automated reminder emails, and on-demand sessions. Branding improvements encompass standardized templates and design for promotional materials, slide decks, handouts, and videos. Accessibility was improved through changes to pointer size and color while screen sharing, online handouts that adhere to WCAG 2.0 standards, and exploring closed captioning for online sessions. Technology enhancements include mobile device screen sharing during online sessions—allowing teaching with mobile apps—as well as the addition of workshop

snippet videos on YouTube. The program’s structural improvements include workflows around scheduling, tracking, and evaluation. Attendance statistics (affiliation status, department, and view time) are collected, and attendees receive a follow up survey.

Conclusion: Staff have solidified processes to increase the professionalization of the program and to provide the infrastructure necessary to limit demands on staff time and ensure sustainability. The Hardin Library instruction team is using attendance statistics, engagement data (e.g., unique viewers, viewing time, and clickthroughs) from YouTube, and qualitative and quantitative survey data to inform future program decisions for the selection of appropriate workshops and content creation as well as scheduling. Because the end-of-session survey completion rate is currently low, strategies to increase response rates are being explored and tested.

What Can We Do about Dr. Google? Utilizing the Electronic Medical Record (EMR) to Prescribe Reliable Online Patient Education

Practice Area: Clinical Support

Ruti Volk, AHIP, Lead, Patient Education and Health Literacy Program, University of Michigan Health System, Ann Arbor, Michigan

Karelyn Munro, Patient Education Resources Coordinator, Michigan Medicine, Ann Arbor, Michigan

Amy Hyde, Patient Education Librarian, University of Michigan - Michigan Medicine, Ann Arbor, Michigan

Background: Research has shown that over 60% of American adults search for health information on the Internet and the majority of health information seekers do not check the source or currency of information they find online. Patients are also not able to correctly assess if the information they find online applies to them. This leads to unnecessary anxiety and stress, medical mistakes and bad outcomes. Physicians and other clinicians are concerned about the wide use of “Dr. Google” and the difficulties of responding to patients demanding unproven or unnecessary clinical tests and therapeutic approaches they found online.

Description: Participants were providers at a large health system utilizing the Epic EMR. The institution maintains a web-based database that links to print and electronic patient education materials that have been vetted by content experts. Clinicians worked with librarians to create sub-pages within the database that link to the resources they recommend for patients seen at a specific clinic or area in the health system. Librarians collaborated with the information technology (IT) department to implement a solution that enables clinicians to quickly and easily

send the sub-page's Uniform Resource Locator (URL) to the After Visit Summary (AVS) or as a message via the patient portal. The process has been named an "Education Rx".

Conclusion: Providing patients with a URL for a webpage that links to recommended patient education online resources has proven to be an effective strategy in directing patients to reliable resources. Analytics data from one surgery clinic demonstrate that the majority of patients who receive the Education Rx in their AVS visit the webpage. The Education Rx has been implemented in over 60 units across the institution and more are in the works. This process can be easily adapted by other institutions using an EMR system such as EPIC or Cerner.

LIGHTNING TALKS: RESEARCH ABSTRACTS

Lightning Talks in this section are sorted by title in alphabetical order.

Characterizing Observational Database Research Using Python

Practice Area: Innovation & Research Practice

Amanda Sawyer, Associate Fellow, National Library of Medicine

Vojtech Huser, n/a, staff scientist, Lister Hill National Center for Biomedical Communications

Objectives: The importance of observational data in healthcare research continues to grow due to its ability to provide valuable contextual information about real-world treatments and cost-effective opportunities for large-scale studies. Currently, no standards exist to guide the collection and presentation of article citations of observational data research literature. Using an existing standard such as the Medical Subject Headings controlled vocabulary can introduce an improved method for revealing themes in this dynamic subset of healthcare research. This project proposes a methodology that informatics librarians and researchers can use to analyze the existing corpus of research in the bibliographies of selected observational databases.

Methods: Clinical Practice Research Datalink (CPRD) was chosen to serve as the gold standard observational database for the development of a methodology to characterize research outputs. Using Python and the NCBI E-Utilities, metadata was collected on articles listed in CPRD’s bibliography and analyzed to develop frequency tables for MeSH keywords. The methodology was applied to two more observational databases – PEDSnet and FDA Sentinel – with the goal of reproducing the methodology with different datasets and identifying potential challenges.

Results: This project developed a broad methodology for characterizing research by identifying top keywords and creating visualizations for CPRD, PEDSnet, and FDA Sentinel observational databases. The top twenty keywords for each database were identified and visualizations demonstrating the breakdown of research were created. Additionally, the project identified key barriers to collecting the observational database bibliographies and proposed areas for future research in this area.

Conclusions: The methodology developed in this project demonstrates one way of characterizing observational data research through the MeSH controlled vocabulary specifically for observational databases tracking research outputs through a bibliography which can be replicated by librarians and researchers for other similar databases. Future research in this area should consider how to automate identification of observational data research and construct bibliographies for databases that are not regularly tracking research outputs.

Developing Librarian Data Services Skills: An Assessment of Current Levels of Competency and a Plan for the Future

Practice Area: Information Management

Justin Fuhr, Liaison Librarian, University of Manitoba, Winnipeg, Manitoba, Canada

Objectives: Research data services are increasingly being offered by health sciences and academic libraries. Librarians may need to upskill in order to provide sufficient services or to build capacity within their institution. This research study measures the current level of data services skills of health sciences and academic librarians. Taking librarian’s current level of skill, this paper then explores the preferred method of professional education in support of librarians providing data services.

Methods: A twenty-two question online survey of Likert-scale questions was circulated. The survey was circulated on listservs (MEDLIB-L, CANMEDLIB, DataLibs, CANLIB-Data) and social media (Twitter, LinkedIn) over a period of two months at the beginning of 2020. Respondents self-assessed their data skills along four overarching categories: general data services, programming languages and software, library instruction, and soft skills. Within each of the four overarching categories are associated skill sets, such as data mining and data curation in the general data services category, and management and leadership within soft skills. Likert-scale questions were also used to measure how important various professional development initiatives were to respondents. Short answer questions were used to gather additional information about the respondent’s self-assessment of data skills and professional development initiatives.

Results: There were 120 responses to the survey. Participants were from Canada, the United States, the United Kingdom, and Australia. There is correlation between percentage of time spent providing data services and higher levels of skill in the two technical categories: general data services, and programming languages/software. Canada and the United States have

similar levels of skill in each of the four overarching categories. There is no correlation between number of years spent in the field and higher levels of skill. There is no correlation between any of the four geographic regions and higher levels of skill in any category.

Conclusions: The study results show that the more time librarians spend performing data services, the higher technical data skills they have. Libraries that plan to or currently offer technical data services should consider hiring a dedicated data librarian. Libraries that choose to do this will increase their capacity to provide a higher level of data services to their users. Based on the study results, librarians need training opportunities to improve skills in specific areas. Training should be provided for librarians who provide any level of data services, including workshops or bootcamps, communities of practice, and mentorship with peers.

Effect of Streaming versus In-Person Virtual Reality Experiences on Empathy in Physician Assistant Students

Practice Area: Education

Elizabeth J. Dyer, AHIP, Interim Dean of Library Services/Research & Teaching Librarian, University of New England Library Services, Biddeford, Maine

Dana Villmore, Assistant Clinical Professor, UNE Physician Assistant Program, Portland, Maine

Objectives: The purpose of this study was to determine if streamed virtual reality (VR) increased knowledge and empathy in physician assistant (PA) students compared to an in-person experience. Since 2017, librarians with PA faculty have incorporated the Alfred Lab into the curriculum, a VR application enabling students to experience vision and hearing loss from a first-person perspective. COVID-19 necessitated streaming the VR to students. This study tested the hypothesis that the more interactive in-person experience resulted in increased understanding and empathy of older adults living with macular degeneration and hearing loss compared to the less interactive streamed experience.

Methods: This mixed-methods study used surveys with Likert-type questions to obtain quantitative data, and open-ended survey and interview questions to obtain qualitative data. The intervention was the Alfred Lab, enabling learners to “embody” a 74-year-old man with advanced macular degeneration and high-frequency hearing loss. Fifty first-year PA students experienced the streamed Alfred Lab and completed pre and post surveys. Follow-up interviews were done with selected faculty and students. Data from 47 PA students who experienced the lab in-person in 2019 were compared to data from the 2020 cohort of 50 students who experienced streaming VR. Data analysis included frequency analysis and paired samples t-test for Likert-type data, and content analysis for qualitative data.

Results: In both cohorts, eight of 11 Likert-type questions showed significance ($p=0.05$) in mean differences between the pre and post-surveys per the paired samples t-test. Frequency analysis of the percentage of students who chose “Agree” or “Strongly Agree” for Likert-type

survey questions showed no major differences between cohorts. Content analysis of open-ended questions also showed similarity between cohorts. Results showed that the intervention is effective in helping students become more empathic health care providers, with similar effects for both the in-person and streaming experience, though the perspective-taking effect may be larger for the in-person cohort.

Conclusions: VR in education and health care is a burgeoning field that presents many opportunities for learning, and for library partnerships with faculty. Designed to provide an experiential learning opportunity as an interactive experience, it was unknown if the less-interactive streaming experience necessitated by COVID-19 would still be effective. This study provided evidence to support that VR delivered in-person or by streaming video is effective in providing future PAs understanding and empathy regarding macular degeneration and hearing loss as presented in the Alfred Lab. Future research could explore whether or not VR learning experiences translate to better patient care.

Librarian involvement in competency-based medical education: a scoping review

Practice Area: Education

John W. Cyrus, Research and Education Librarian, Health Sciences Library / Research and Education, Richmond, Virginia

Molly Knapp, AHIP, Training Development Manager, NNLM, University of Utah Eccles Health Sciences Library, Houston, Texas

Brandon Patterson, Technology Engagement Librarian, Eccles Health Sciences Library, Salt Lake City, Utah

Laura Zeigen, MA, MLIS, MPH, AHIP, Health Sciences Education and Research Librarian, OHSU Library, Portland, Oregon

Amy Blevins, MALS, Associate Director for Public Services, Ruth Lilly Medical Library, Indiana

Objectives: The Association of Academic Health Science Libraries Competency Based Medical Education (CBME) task force was assigned several charges. The primary charge is to “create a collection of case studies, vignettes, best practice stories, or other representations demonstrating the beneficial roles and positive impacts of librarian engagement in [CBME].” In discussing ways to accomplish this, members of the task force identified a gap in literature addressing the ways in which librarians are directly involved with CBME. In order to address this gap, the task force will conduct a scoping review to identify demonstrable evidence of librarian involvement in competency-based medical education.

Methods: We have created a protocol and are conducting a scoping review following PRISMA-ScR and Joanna Briggs Institute methodology. The review addresses the question: Is there demonstrable evidence of librarian involvement in competency-based medical education? Specific outcomes of interest include roles that librarians play in supporting CBME, outcomes used to measure the impact of librarian work in CBME, and evidence that outcomes measured are related to clinical competence of learners. This scoping review will be run in the following databases: Ovid/Medline or PubMed, Embase, ERIC, CINAHL, Scopus, LITA/LISA/LISTA. Our inclusion criteria include: studies with outcomes for undergraduate medical education in the United States for programs with LCME accreditation that include librarian involvement. Our exclusion criteria are non-English language publications.

Results: This section will be updated as results are available if this lightning talk is accepted. Our initial approach to search strategies and a preliminary data dictionary can be shared.

Conclusions: Now, more than ever, it is important for librarians to show demonstrable evidence of the ways their involvement directly benefits institutions engaged in competency-based medical education. The purpose of this project is to highlight this evidence for medical educators.

A Knowledge Management System framework to crack open science

Practice Area: Global Health & Health Equity

Lisa M. Kruesi, PhD Candidate, Faculty of Information Technology, Monash University, Victoria, Victoria 12:00:00 AM

Objectives: A conceptual Knowledge Management System (KMS) framework has been developed for analyzing and evaluating repositories and platforms that support the advancement of open science. Library and communications higher degree students tested the KMS framework, at an online workshop, by analyzing Epistemonikos, Europe PMC, and ResearchGate during an online workshop. Did the students find the framework a useful tool? What improvements to the framework were proposed? How could health library and information services benefit from the adoption of the framework?

Methods: The KMS framework was derived from action research undertaken during a Ph.D. program. The KMS framework comprises nine processes that are the foundation for biomedical knowledge; these include discovery, creation, representation, classification, storage, retrieval, dissemination, transfer, and translation. The design and evaluation criteria are achieved from auditing and aligning the nine processes with people, process, technology, and content elements of information platforms. This presentation focuses on the final cycle of the action research, which involved twenty higher degree students in applying, reporting, and summing up their experience of using the framework. Students were provided with links to reading materials to prepare for the session and they were allocated to one of three groups to

evaluate either Epistemonikos, Europe PMC, or ResearchGate. The findings were tabulated using Google forms and Google sheets.

Results: Students responded positively to all of the feedback questions. For example, all of them agreed that the KMS framework can help with designing, analyzing, and evaluating open scholarship repositories. Even so, the verbal student summaries given at the workshop did raise some contrasting findings. It was evident during the verbal feedback during the workshop that the students evaluated the typical information service processes rather than taking an expansive knowledge management approach. Students spoke of traditional areas such as retrieval and representation and did not refer to processes such as transfer and translation.

Conclusions: Even though the student worksheet feedback was positive, it was evident during the verbal feedback session that the students focused on traditional, information services processes, rather than appraising aspects such as content discoverability and interoperability. It would be beneficial to test the KMS framework further and explore the opportunity to help library students and librarian practitioners adopt a wider paradigm for the analysis of information platforms and repositories. The KMS conceptual framework presents a new tool to critique open science platforms and repositories.

Librarian Collaboration with Nurse COVID and Historical Epidemics Lit Repository

Practice Area: Information Management

Marie St. Pierre, AHIP, Medical Librarian, Childrens Hospital Colorado, Aurora, Colorado

Figaro Loresto Jr, Nurse Scientist, Childrens Hospital Colorado

Objectives: To provide the most up to date information on nursing and the novel coronavirus and comparisons to historical pandemics, nurse researchers and a librarian at Children’s Hospital Colorado abstracted literature around COVID-19 specific to nursing from two established literature databases, the LitCOVID and CORD-19. Abstracting the key information from these database as well as abstracts, they created a repository that utilized text mining to summarize the literature providing improved accessibility to the literature for nurses.

Methods: To accomplish the stated objective, two databases, the CORD-19 and LitCOVID were searched using keywords ‘nurse’ or ‘nursing’ to search for publications around COVID-19 applicable to nursing. The two databases were searched daily to see what new publications have appeared. Information such as the title, authors, journal, and abstract are gathered and added to the regularly updated data set for the repository. This in turn is examined by a nurse scientist who incorporated data mining and natural language techniques to analyze the abstracts. A searchable application has been created to allow nurses to access this information.

Results: The repository was created and deployed on May 2020. To date there are 2179 articles around COVID-19 and historical epidemics applicable to nursing within the repository. As of December 2020, it has been used 1441 times at about 2 hours per week. An analysis of 770 articles in July revealed that the nursing literature focused much on the psychological status and care of health care workers as well as the need for rapid education. Further, other themes included nursing delivery changes in the light of the challenges of the large numbers of patients and new precautions needed.

Conclusions: The repository highlighted an important and useful tool to support for nurses in accessing relevant literature. Nurses primary responsibility is on the bedside and having a literature resource allows them to quickly obtain needed information to support evidence-based practice., Other repositories could be created in the future for other topics of interest to nursing, or other health professions. Librarians can help in planning which databases and information will be needed for individualizing such repositories, and in gathering the data. The partnership between nurse scientist and librarian is highlighted in this work.

Leading in Health Sciences Librarianship: Perspectives from Black Library Deans and Directors

Practice Area: Professionalism & Leadership

Bethany S. McGowan, MLIS, MS, AHIP, Assistant Professor, Purdue University Libraries and School of Information Studies, West Lafayette, Indiana

Jahala Simuel, Medical Librarian and Head, Access Services, Louis Stokes Health Sciences Library, Clayton, North Carolina

Objectives: Having people of color in leadership positions in health sciences libraries is crucial because it is one of the domains where librarians can affect the most change while setting examples for those seeking similar positions. This talk will illustrate how Black health sciences library leaders are championing minority recruitment and retention and advocating for opportunities for inclusion, cultural competency, and humility; while proactively dealing with the challenges of bias, microaggressions, racism, and more. Their efforts are vital in creating an open, welcoming, and inclusive environment.

Methods: The authors will connect with AAMLA representatives to help identify Black library directors and deans from institutions belonging to the AAHSL. They will conduct virtual video interviews, transcribe the interviews, and topically analyze the interview text data. A manual topical analysis will be conducted using Excel, and automated analyses will be conducted using Voyant Tools. Results from the manual and automated analyses will be compared and discussed. The authors will identify recurring themes across responses and will seek to understand how responses complement or contradict each other.

Results: We identified 13 potential candidates and included 10 interviews in this analysis. Perhaps the strongest recurring theme heard across the interviews is that library leaders must

understand and respond to their institution’s changing needs. Leaders should master engagement strategies and should seek out opportunities to advocate for the library.

Study participants noted shrinking library staff and budgets as key challenges, and leaders anticipate that the COVID-19 pandemic will exacerbate these issues. This trend, paired with concerns related to the consolidation of health sciences libraries into the broader university library system, could drastically reduce the number of leadership positions available in health sciences libraries.

Conclusion: HBCUs have played an important role in preparing Black librarians for leadership positions. 60% of this study’s participants are HBCU alumni. And, the Southeast Atlantic region has the most Black health sciences library leaders, with half of this study’s participants being from that region.

Black library leaders are optimistic as more people of color enter careers in libraries and enter leadership positions. In fact, half (5/10) of this study’s participants are new leaders with 1-5 years of experience. However, our findings show that Black health sciences librarians are often very experienced before entering leadership positions. Though the average time spent in librarianship was 28.7 years, the average time of leadership experience was only 5.6 years.

Navigating COVID-19 databases and resources to find public health evidence: Tips & tricks

Practice Area: Innovation & Research Practice

Leah Hagerman, Research Assistant, National Collaborating Centre for Methods and Tools, , Ontario 12:00:00 AM

Sarah Neil-Sztramko, Assistant Professor, Health Research Methods, Evidence, Impact, McMaster University, ,

Emily Clark, Knowledge Broker, National Collaborating Centre for Methods and Tools, Hamilton, Ontario, Canada

Maureen Dobbins, Professor, School of Nursing, Ontario, Canada

Objectives: The COVID-19 pandemic has created a need for rapidly available, high-quality, synthesized evidence. Our team has responded to the needs of public health decision makers in Canada by creating a rapid evidence service, through which we quickly produce rapid reviews and review updates in as little as 5-10 days. As part of this process, we developed a transferrable strategy to quickly and efficiently locate and identify relevant evidence. This strategy includes 20 databases and can be used across most public health topics.

Methods: Our team accepts COVID-19 rapid review questions from public health stakeholders, which we prioritize based on timeliness, team expertise, availability of evidence,

and potential for duplication of research efforts. Possible relevant key terms are first searched in high-yield databases and repositories to assess return volume and plan workflow. Initial search results are screened for recent, high-quality syntheses, which are assessed to validate and revise search terms. Terms are adjusted to maintain a manageable volume of search results while maintaining a rigorous search. Once terms have been finalized, search strings are developed and adapted for each of nine core databases that have proven to provide comprehensive search results, plus a selection of eleven topic-specific databases. Search engine sophistication of each database, such as availability of advanced search functions and evidence sorting functions, is taken into account when developing search strings.

Results: Our transferrable and adaptable search strategy has allowed our team to complete 50 rapid reviews and updates on 27 unique topics in nine months. Each search is completed by one or two team members often within one business day. As the COVID-19 pandemic evolves, databases and search terms are revisited to ensure comprehension, efficiency, and thoroughness of the search. This search strategy allows for rapid and thorough identification of syntheses, single studies, and preprint studies relevant to public health topics concerning the COVID-19 pandemic.

Conclusions: Our team’s optimized search strategy allows for rapid identification of newly published evidence on COVID-19. This strategy allows us to pivot to new research topics to rapidly meet the evidence needs of decision makers as well as conduct efficient review updates. Development of this strategy can be used to inform future efforts to rapidly identify evidence. Reviews completed using this search strategy have been used by decision makers across Canada and worldwide and have been accessed in over nine countries, including Canada, the United States, and the United Kingdom.

Programmatic generation of bibliometrics statistics for faculty at Weill Cornell Medicine

Practice Area: Innovation & Research Practice

Paul Albert, Identity & Access Management Architect / Associate Professor, Weill Cornell Medicine, New York, New York

Sarbajit Dutta, n/a, Senior Software Developer, Weill Cornell Medicine, Somerset, New Jersey

Terrie R. Wheeler, AMLS, Director and Chair, Samuel J. Wood Library, New York, New York

Objectives: Committees and senior leadership at Weill Cornell Medicine regularly need to decide whether to appoint, promote, or grant tenure to faculty. Towards that end, a key consideration is scholarly productivity. To facilitate that work, we will use our publication data to output and share with stakeholders a series of bibliometric statements which can be used in evaluations.

Methods: With its open source ReCiter publication disambiguation system, Weill Cornell Medicine Library tracks the publication output of over 15,000 individuals at Weill Cornell Medicine. This includes everyone from full-time faculty to postdocs to alumni. These data, updated weekly, can be used to inform and support the judgments of decision-makers. Such data can also be used by faculty for grant applications. Drawing upon data in our publications database, we wrote a series of SQL queries against our publication database, which output a set of 10 bibliometric statements.

Results: On a weekly basis, we generate a set of statistics which are used for faculty assessment. These include statements like the following.

- Dr. X has an h-index of 82.

- Dr. X has authored 24 academic articles with times cited counts in top 10% relative to NIH-funded studies.

- Dr. X has authored 8 academic articles as a senior author with times cited counts in top 5% relative to NIH-funded studies.

The code for this output will be shared.

Survey of knowledge graph usage in biomedical ontologies

Practice Area: Innovation & Research Practice

Sonia M. Pascua, Ontologist / Research Assistant / Instructor of Record, Metadata Research Center / College of Computing and Informatics, Philadelphia, Pennsylvania

Objectives: Monarch Initiative is one of the efforts to semantically integrate information across databases by employing ontologies. It is an integrative data and analytic platform connecting phenotypes to genotypes across species that imported data from 30 data sources using 24 ontologies. This study aims to understand the ontologies used in the Monarch Initiatives to account for the knowledge graph usage of the project. Knowledge graph, knowledge bases, ontologies, are interchangeably used due to lacking solid foundation on their definition, scope and most of all usage. This study also aims delineating the boundaries of these terms to better understand and use them.

Methods: Rapid evidence reviews which is a variation of systematic review is the method utilized in this study. All bio-ontologies in the Monarch Initiative are subjected for grey literature and descriptive summary of the findings is the synthesis output. Popular "grey literature" resources include clinicaltrials.gov, NIH RePORTER and the monarchinitiative.org. FINER (Feasible, Interesting, Novel, Ethical, Relevant) and PICOT (Population, Intervention, Comparison, Outcome of Interest, Time) are the 2 frameworks used for Refine Questions and Define Parameters stages. For biases identification, issues per possible rapid review process

will be introduced to the study protocol and are decided to be acceptable or not given time constraints. Quality appraisal to identify the quality of the study conducted includes evidence summary matrices. Evidence synthesis includes the results of the implications for the study design and methods together with the relevance of the study.

Results: Study is still being conducted and is expected to complete by Q1 of 2021.

Conclusions: Today’s information landscape pictures knowledge graph to be a marketing-oriented term associated with the structed data representation. When Google announced a product called the Knowledge Graph in 2012, it’s considered more than an evolving project and a vision rather than a precise notion or system. It is argued to be the materialization and implementation of the Semantic Web Project. In 2013, Facebook launched its graph searched which followed by other giants like Microsoft, Amazon, eBay etc. in using knowledge graphs. As time passed, KG has formalized specially in the field of knowledge management. It is the convergence of statistical and logical methods. Deep and machine learning are soaring to compliment the need for automation in training and testing KG data.

Testing Search Filters for Covid-19 Literature

Practice Area: Innovation & Research Practice

Edwin V. Sperr, AHIP, Clincial Information Librarian, AU/UGA Medical Partnership, Athens, Georgia

Objectives: The unprecedented flood of items relating to Covid-19 makes it crucial for users to find effective ways to filter their searches. To this end, several different groups have released search filters designed to focus a user’s search on topics such as diagnosis, critical care or “long covid” syndrome. The objective of this project is to examine how effective these filters are in terms of sensitivity and selectivity.

Methods: This project will proceed in stages, beginning with the selection of publicly available Covid-19 search filters. Once those are selected, team members will collaborate on a set of rubrics to be used for testing the concept behind each filter (i.e. what is meant by “transmission” in this context, etc.).

Once grouped sets of filters and rubrics are defined, a very broad search strategy will be constructed for each set. These searches will be performed against PubMed, and a random selection of resulting items will be retrieved for examination by team members. For each randomly selected set of results, members will examine each item and determine whether it matches the predetermined rubric. Those matching items will comprise “known good” validation sets that can later be used to test each search filter in a quantitative manner.

Tracking COVID Compliance in a Medical Library: An Observational Study

Practice Area: Professionalism & Leadership

Laura Menard, Assistant Director for Medical Education and Access Services, Indiana University, Indianapolis, Indiana

Objectives: Research question: Given a strict, campus-wide mask and social distancing mandate to slow the spread of COVID-19, at what rate is adherence observed in patrons of a large medical library?

Methods: At a large medical school in the Midwest, a mask ordinance was implemented to slow the spread of COVID-19 when campus was re-opened in the summer of 2020. All students, faculty, and staff were required to sign a commitment form affirming that they would wear a mask while on campus. By the fall of 2020, many in the community were beginning to experience "pandemic fatigue," which was reflected in the rising number of cases in the state and region. Curious about whether the patrons of the library continued to adhere to the mask mandate when out of sight of the main desk and any enforcement mechanisms, we instituted a compliance check to be performed by a staff member every other hour. Staff observed users of the library, tracking total number of patrons with and without masks.

Results: During the observation period of October 2020-March 2021, rates of mask mandate compliance in the library averaged between 87%-100%. While 87% is a relatively low rate of compliance, most of these cases were noted while patrons were sitting at individual, isolated desks. Staff in the library conjecture that this may have been due to misunderstanding on the part of the patrons about when masks were required to be worn. Staff did not a few instances of refusal when patrons were asked to don masks, which were addressed at a higher level of authority.

Conclusions: Even with a strong mask mandate and clear guidance from the school, COVID-19 precaution compliance was not perfect. Going forward, library faculty and staff will continue to work with student groups and medical school administration to ensure that our patrons adhere to best practices for protecting the health and safety of the campus community.

Trends in the Covid-19 Literature

Practice Area: Innovation & Research Practice

Edwin V. Sperr, AHIP, Clincial Information Librarian, AU/UGA Medical Partnership, Athens, Georgia

Objectives: With over 100,000 (and counting) citations for “covid-19” in PubMed, the impact of the current pandemic on the biomedical literature is apparent to even the most casual reader. Less clear however is how that impact has changed over the course of the past year, or how it has varied between disciplines. This study seeks to quantify how the production of items relating to Covid-19 has changed over time by examining those citations in PubMed on a weekly basis.

Methods: Counts of items relating to Covid-19 in PubMed were determined by programmatically searching the string “Covid-19” in PubMed using Python programs to interface with NCBI’s public API. Searches were scoped by week using the “Create Date” PubMed field. In addition to raw numbers of Covid-19 items, further counts were taken of those Covid-19 items found in Journal Subsets, Publication Types and selected by the LitCovid filters.

Results: Overall production of Covid-19 items quickly rose to a level of 8,000 per 100,000 PubMed items in May of 2020 and have primarily remained in the range of 6,500 to 8,000 up to the present. For Journal Subsets, the most striking pattern was that seen in the percentage of Covid-19 items found in "Abridged Index Medicus". For this large and important subset, just over 20% of all items were about Covid-19 in May of 2020, but that percentage has fallen steadily in subsequent months. In terms of Publication Types, letters was the predominant type last May, but has since been overtaken by reviews. Finally, for LitCovid items, there was a notable decline in "Mechanism" items from the beginning of the pandemic, when they comprised over half of all items.

Conclusions: Much has changed over the past year in terms of our understanding of SARS-CoV-2 and Covid-19. In the face of this, the overall pattern of Covid-19 item production in PubMed holding steady seems somewhat paradoxical. It remains to be seen if this trend will hold as 2021 continues.

Trends In Osteopathic Medical Education: Development of a Scoping Review

Practice Area: Information Services

Megan De Armond, Research and Instruction Librarian, Assistant Professor, Jay Sexter Library, Henderson, Nevada

Abbey Griffith, MLS, Medical Librarian, Kansas City University-Joplin, Joplin, Missouri

Katie Hoskins, Research and Instruction Librarian, California Health Sciences University, ,

Molly Montgomery, MLS, MS, Director of Library Services, Idaho College of Osteopathic Medicine, Meridian, Idaho

Debra Orr-Roderick, Medical Librarian / Manager, Heritage College of Osteopathic Medicine, Ohio University, Ohio

Hannah Pollard, MLIS, AHIP, Health Sciences Librarian, Pacific Northwest University of Health Sciences, , Washington

Melinda C. Robertson, Assistant Library Director for Health Sciences and Medical Librarian, University of Pikeville-Kentucky College of Osteopathic Medicine, Pikeville, Kentucky

Darell Schmick, AHIP, Director of Library Services, Noorda-COM Library, ,

Jeanne Strausman, AHIP, Senior Medical Librarian, New York Institute of Technology/College of Osteopathic Medicine, Old Westbury, New York

Sarah Wade, AHIP, Assistant Medical Librarian, Campbell University Jerry M. Wallace School of Osteopathic Medicine Medical Library, Lillington, North Carolina

Objectives: The American Association of Colleges of Osteopathic Medicine (AACOM) sent out a call to the Council of Osteopathic Librarians (COOL) to explore trends in osteopathic medical education literature. Attendees will gain an understanding of how ten librarians from ten different institutions successfully manage a virtual project across multiple time zones, collaborating in the development of sound search strategies within a specialized subset of medical librarianship.

Methods: Librarians joined forces to start identifying trends from literature published from 2010 to the present in the field of undergraduate and graduate osteopathic medical education, with the exclusion of articles related to COVID-19. We considered different types of reviews and agreed that a scoping review would be best at achieving this goal. Our workflow has been an iterative process involving multiple strategies to manage the project such as creating various subgroups to streamline workflow, creating a timeline, designating two members as representatives to AACOM, and using technologies to coordinate such as email, Google Suite, Sciwheel, Teams, When2Meet, and Zoom.

Results: The group is making progress on project goals and deliverables such as the scoping review protocol. Throughout this process the group has found several best practices for online collaboration, such as structuring tasks so not all members have to be present to complete the task, creating multiple subgroups for quick development, and developing a culture of flexibility to adjust as needed.

Conclusions: While it can be challenging to collaborate across multiple organizations and time zones, there are a variety of tools and techniques to facilitate project goals while simultaneously fostering connection and community.

When My Preferred Term Isn't in MeSH: Automating Analysis of Proposed MeSH Terms with Python to Support Researchers

Practice Area: Innovation & Research Practice

Levi Dolan, Associate Fellow, National Library of Medicine

Amanda Sawyer, Associate Fellow, National Library of Medicine

Dan-Sung Cho, n/a, MeSH Senior Analyst, NLM, MeSH

Objectives: Each year, National Library of Medicine analysts evaluate proposed terms for inclusion in the Medical Subject Headings (MeSH) controlled vocabulary. This project demonstrates how Python can automate stages of new MeSH term analysis workflow, and how librarians and researchers can use this same method for insight into whether their preferred term is likely to appear in MeSH in the near future, and obtain the scope of how their preferred term has been previously indexed.

Methods: Two features of new MeSH term analysis were implemented and refined through this project, and one feature was exploratorily designed utilizing original Python code supported by existing open-source Python libraries, potential new MeSH terms, and PubMed citation information. Dan Cho, Lead MeSH Analyst and sponsor for this project, created a Jupyter Notebook which two NLM Associate Fellows refined, identifying and testing areas where computational efficiency and user results could be improved. Finally, the associates conducted research on natural language processing techniques in Python and established preliminary parameters for a fully developed NLP feature.

Results: Three measurable efficiency improvements were identified through comparison of computational processing time, and the modifications were integrated into the process drafted in the original Jupyter Notebook. In total, these improvements resulted in a reduction of computational processing time of approximately 90%; the reduction was not uniform in testing times. However, this project provides the functionality to accept a user's term input, count its appearances in titles and abstracts in MEDLINE PubMed citations, and then return the percentages of existing MeSH terms under which this selected group of citations has been previously indexed.

Conclusions: The modifications to new MeSH term analysis this project presents improve the process of quickly getting scoping information about existing and potential MeSH terms, and the literature associated with them. Therefore, further testing and implementation of these automation processes in a format that does not require Python knowledge would be a useful functional progression. Future research could explore the viability of natural language processing solutions to further inform analysts and provide additional context.

Virtual and Augmented Reality in Health Sciences Libraries: A Scoping Review

Practice Area: Information Services

Alanna Campbell, Public Services Librarian, Health Sciences Library, Sudbury, Ontario, Canada

Objectives: This scoping review identifies why and how health sciences libraries are integrating virtual reality (VR) and augmented (AR) technologies into their collections, spaces and workloads. This review aims to inform health sciences libraries at any stage in the adoption of AR/VR within their service provisions and spaces.

Methods: A peer-reviewed systematic search was conducted in Ovid MEDLINE, CINAHL, ERIC, Education Source and LISTA for articles published between January 2016-February 2021. Article citation lists as well as the conference proceedings, paper and poster presentations for the Medical Library Association and Canadian Health Libraries Association, were also scanned. A total of 19 resources were included for data extraction from the 176 located through searching.

Results: Resulting resources varied in-depth as they included a number of formats such as program descriptions, columns and a conference poster, conference abstract and detailed primer. A number of themes emerged during review. This included reasons for AR/VR initiatives; types of initiatives; financing; hardware/software; collaboration; space; promotion; staffing; program assessment and future planning. Reasons for implementation were varied, however centered around collaboration - between the library, external departments, researchers and learners. How these initiatives launched showed some diversity, although shared commonalities including being highly dependent on financing, space, staffing and promotion.

Conclusion: The results showed that primarily academic health sciences libraries are currently integrating AR/VR into their workloads. Further research is needed on its implementation in hospital and other health sciences libraries. AR and VR are already being implemented, and in some cases required, at the research and educational level globally, this is only expected to grow as the technology becomes more affordable and diverse applications are made available.

LIGHTNING TALKS: PROGRAM DESCRIPTION ABSTRACTS

Lightning Talks in this section are sorted by title in alphabetical order.

2 for 1: promoting preprints and librarianship as an ASAPbio Fellow

Practice Area: Professionalism & Leadership

Carrie L. Iwema, AHIP, Coordinator of Basic Science Services, Health Sciences Library System, Pittsburgh, Pennsylvania

Background: ASAPbio (Accelerating Science and Publication in biology) is a global scientist-led organization that promotes transparency and innovation in life science communication, with a focus on preprints and open peer review. Beginning in 2020, ASAPbio introduced a Fellows program with the aim of providing participants with information, tools, and support so they in turn will be equipped to offer guidance on preprint resources within their own communities.

Description: I was the only librarian to be selected as a Fellow in the first cohort of the program, which included primarily biomedical researchers such as postdocs and grad students. There were 26 participants from Africa, Europe, North America, and South America. Over six months, we engaged in monthly sessions that explored different facets of preprints, shared ideas and feedback on how to encourage alternative modes of scientific communication, and worked on small group projects to support what was discussed. During this experience I interacted with scientists and preprint proponents from around the world, gained perspectives on emerging trends in research communication, and learned how to promote concepts still gaining traction. Simultaneously, I was able to enthusiastically advocate for librarians in all of these interactions and open the eyes of some to our role in supporting the research life cycle.

Conclusion: This lightning talk briefly presents my experience as an ASAPbio Fellow. Thanks to this opportunity I was able to achieve two accomplishments: (1) learn more about preprints in a welcoming community, which directly influenced the content of a preprints class I co-teach as well as sparked the idea for an open peer review “something” at my institution (…in progress…) and (2) promote biomedical librarians as valuable assets to the preprints community. For information on ASAPbio, preprints, peer review, and the Fellows program: https://asapbio.org/.

Accelerated learning plan for consumer health information specialization as response to pandemic

Practice Area: Clinical Support

Molly Knapp, AHIP, Training Development Manager, NNLM, University of Utah Eccles Health Sciences Library, Houston, Texas

Background: In February 2020 I created an accelerated learning plan for MLA's Consumer Health Information Specialization (CHIS) to respond to the pandemic.

Description: Based on my experience as an academic health sciences librarian during Hurricane Katrina, I personally knew that professional development was a way to fill the open hours made available due to a sudden move to remote work. Based on my experience as a training development specialist for a large, distributed organization, I knew the landscape of our CHIS classes, and suspected we had enough free, fully-built educational products to enable an abruptly remote library workforce to earn a CHIS specialization completely online in about a month. I also knew we had stakeholders who quickly needed to replace in-person training with "something else" before April 30th, 2020. And finally, I knew that we offered sponsorships for the CHIS application fee, making this specialization completely free to library workers. So I opened up a new spreadsheet and got to work.

Conclusion: A year later we see that the accelerated learning plan had a direct influence on our training statistics, including a 25% increase of consumer health related classes taught across the U.S. from 2019-20 (57 classes) to 2020-21 (71 classes); and an incredible 46% increase of consumer health class registrations from 2019-20 (5,775) to 2020-21 (8,422). CHIS sponsorship recipients increased 29% from 2019-20 (138) to 2020-21 (178). In retrospect, I realize now how this project was a comforting and consuming way to create order while the world was falling apart. Take the things that you have and build something new. Connections can increase health literacy.

Assessing library site value and client satisfaction during a global pandemic

Practice Area: Professionalism & Leadership

Morgan Truax, Director, Knowledge Resource Service, Alberta Health Services, Edmonton, Alberta, Canada

Jeanette Blanchard, Team Lead, Knowledge Resource Service, Alberta Health Services, Edmonton, Alberta, Canada

Kathryn Tippell-Smith, Team Lead, Knowledge Resource Service, Alberta Health Services, Calgary, Alberta, Canada

Background: A provincial health authority library system sought to understand the use and value of its 6 geographically dispersed libraries, to inform future strategic planning. This was undertaken during a time when libraries were temporarily closed due to the Covid-19 pandemic. Under normal circumstances, gate counts and exit interviews would be used to gather information from library users as to their satisfaction with the library space and offerings. Library leadership was challenged to evaluate libraries and gain client perspectives while working from home.

Description: A unique methodology was devised to reach out to library users and gain their perspective. This was accomplished through virtual, one-on-one interviews between library staff and known users of each library, using a set of pre-prepared questions to guide the conversation. The questions focused on usage, satisfaction, barriers, perceived value, and ideas for future development. A target of 5 interviews per library was set, which was deemed to be achievable for both busy library staff and clinicians, yet high enough to yield useful results. Each interview was manually recorded, entered into a spreadsheet, and then coded with descriptive tags and broader themes, to assess and compare the results. Two outputs were then produced – a system-wide overview summary, and a more granular, site-by-site summary. This allowed for broad conclusions as well as differentiation of issues between sites.

Conclusion: This method was generally successful to gauge client satisfaction and perspectives. Each library met or exceeded the 5-interview target, and interviewees included a wide range of job roles. The information gathered will inform library strategic direction and has provided a template for future outreach. However, there were known limitations which must be acknowledged, such as no interviews with non-users, no gate count, variability in interview techniques, and no validation of coding and theming. Regardless, this process allowed strategic planning initiatives to continue, even in the midst of a global pandemic and temporary library closures.

Books to Bedside: Developing a direct delivery system to bring library services to patients during the COVID-19 pandemic restrictions.

Practice Area: Information Services

Andrea Slonosky, Information Specialist, Sinai Health -Bridgepoint Active Healthcare, Toronto, Ontario, Canada

Background: Located in a 464-bed continuing care/rehabilitation hospital, we provide rehabilitation patients, caregivers and staff with consumer health information, leisure reading material, and access to the internet. Bed-bound patients are served by a book cart. The library is managed by a librarian supervising a team of volunteers. During the COVID-19 pandemic the hospital was closed to visitors and volunteers. The library was closed to patients, the book

cart service was banned and sharing and circulating materials was prohibited. The librarian had to adapt quickly and constantly to provide patients with access to library services and collections.

Description: We established a project to work with the Therapeutic Recreation department to develop and implement services providing technical support both to help patients communicate with family and friends and to access our collections of leisure and health materials. We created easy-to-read instructions for using tablets, setting up accounts, accessing FaceTime/Skype/etc. Library staff assisted in facilitating video calls between patients and families and directed patients towards online resources. By following the Reopening Archives, Libraries, and Museums project we developed a site -specific protocol to provide a safe direct-to-patient delivery model. We created an online catalog and circulation system for the library’s collection of leisure books so that patients could select books remotely.

Conclusion: Through flexibility, following real-time research, and collaboration with other hospital departments, the library has been able to develop a new model of service to support patient care and rehabilitation at a time when many other activities have been suspended indefinitely. To date the library has added 2592 titles to its catalogue and served 129 patients over 6 months. Staff have appreciated help with technology, with handouts being used by patients and staff. All parties have been pleased with the online catalogue. Partnering with the Therapeutic Recreation department allows the library to provide services to patients who would not have used the library pre-pandemic, either because they were not sufficiently mobile or because they were not aware it existed.

Building first-year medical students’ skills in finding, evaluating, and visualizing health information through a “Debunking Medical Myths” module

Practice Area: Education

Katherine Goold Akers, Biomedical Research and Data Specialist, Shiffman Medical Library, Detroit, Michigan

Ella Hu, Biomedical Sciences Reference & Research Informationist, Shiffman Medical Library, Wayne State University, Michigan

Background: To improve community health, future physicians must be able to locate, appraise, and assimilate evidence from biomedical studies and present health information in plain language to patients. Here, we describe our experience in developing a semester-long virtual “Debunking Medical Myths” module embedded in a required service learning course for first-year medical students.

Description: In collaboration with faculty in the School of Medicine, we developed and implemented an online “Debunking Medical Myths” module as part of a required service

learning course for first-year medical students. Across a series of multiple assignments, groups of students chose a COVID-19-related medical myth, searched the literature to find evidence speaking to that myth, evaluated the literature to select the strongest evidence, and created an infographic that debunked the myth for a layperson audience.

Conclusion: Students showed evidence of taking a thoughtful and critical approach to literature searching, evaluating articles considering the hierarchy of evidence, and designing effective infographics. Their final products received positive evaluations from peers, faculty, and community members.

The “Debunking Medical Myths” module can be adapted and used for health information literacy instruction for different types of undergraduate, graduate, and professional students.

Capturing Oral Histories of Healthcare Workers During the COVID19 Pandemic

Practice Area: Clinical Support

Brandon Patterson, Technology Engagement Librarian, Eccles Health Sciences Library, Salt Lake City, Utah

Anna Neatrour, Interim Head of Digital Library Services and Digital Initiatives Librarian, University of Utah Marriott Library, Utah

Gretchen Case, Associate Professor, University of Utah School of Medicine, Salt Lake City, Utah

Jeremy Myntti, Interim Associate Dean, University of Utah, J. Willard Marriott Library, Salt Lake City, Utah

Background: In early April 2020, a physician from the emergency department at the institution’s hospital approached the library asking if there was a way to document the collaboration efforts he was seeing around the hospital and beyond to confront the COVID-19 pandemic. After being connected to an oral history expert in the medical humanities program at the School of Medicine, a protocol was developed that helped speak to the process of capturing these important stories.

Description: Librarians at the main campus academic library created a collection that captured photos and stories from around the state about the COVID19 Pandemic crisis. This collection was expanded and adapted to involve a section dedicated to oral histories. An electronic release form was developed and entrees could be collected digitally. All oral histories could be taken through a videoconferencing application, making the full process easy for busy healthcare workers, while also complying with current health regulations.

Conclusion: The COVID-19 Oral History Collection currently holds 59 interviews and has grown beyond telling the stories of healthcare workers. In partnering with a history class, students have interviewed professors, city council members, ski and recreational leaders, and others in the community. The Oral History Collection fits within a larger COVID-19 collection that also highlights photography and personal stories. The oral histories are among the most visited in the collection.

Community Building with Conversations about COVID-19

Practice Area: Information Services

Nora Franco, Consumer Health Librarian, Network of the National Library of Medicine Pacific Southwest Region / UCLA Biomedical Library, Los Angeles, California

Yamila M. El-Khayat, Outreach Services Librarian, University of Arizona Health Sciences Library, Tucson, Arizona

Lara Miller, Analytics & Assessment Librarian, University of Arizona Libraries, ,

It is well known that group gatherings and collective events are an important socio-cultural characteristic of Latinx and Native communities, and the COVID-19 pandemic has drastically altered the setting in which these gatherings take place. To address this problem, a group of health sciences librarians produced an online series of platicas/ cafecitos , or conversations, held in collaboration with several promotores, or community health workers. Librarians also worked with 2nd year medical students, introducing them to reliable health information resources in order for them to use and share with classmates. The focus of this approach is to train both medical students and community health workers how to access COVID-19 health information resources and products of the National Library of Medicine, National Institutes of Health, Centers for Disease Control and other government agencies. Health topics include infection control and prevention, diagnosis, treatment, and mental health with an emphasis on cultural relevance.

The project team, made up of librarians and medical students, designed a program using multimodal forms of communication to recruit participants in the Latinx (Promotores) and Native American Community Health Workers. The team will conduct and promote sessions using social media, Zoom, local radio broadcasts and newspaper ads. The curriculum includes the promotion of health information resources that can be used to learn more about health topics and develop culturally relevant information which can be disseminated related to Covid-19. After the session, participants will share the information learned with community members to increase their knowledge of NLM and reliable health information resources. Evaluation of the program will ask participants how they plan to share the information learned with community members and whether they think it will be a useful set of tools for the community.

The evaluation methods used throughout the program will inform future developments or iterations. The assessment librarian used data collected from surveys, pre-tests, and

observational techniques to assess both medical students and Community Health Workers. Medical students will acquire the knowledge, skills, and abilities necessary to teach health literacy training to Community Health Workers. Community Health Workers will concurrently increase their knowledge, skills, and abilities necessary to implement health literacy activities in their communities. This will lead to Community Health Workers’ increased planning and implementation of health literacy outreach activities and community engagement. In the long term, Native American and LatinX communities will have increased access to the National Library of Medicine and reliable health information resources.

Creation and evolution of a Rapid Evidence Service to support evidence-informed public health decision making for COVID-19

Practice Area: Innovation & Research Practice

Sarah Neil-Sztramko, Assistant Professor, Health Research Methods, Evidence, Impact, McMaster University

Emily Belita, RN, PhD, Postdoctoral Fellow, School of Nursing, McMaster University

Robyn L. Traynor, Research Associate, National Collaborating Centre for Methods and Tools, Canada

Emily Clark, Knowledge Broker, National Collaborating Centre for Methods and Tools, Hamilton, Ontario, Canada

Leah Hagerman, Research Assistant, National Collaborating Centre for Methods and Tools, Ontario, Canada

Maureen Dobbins, Professor, School of Nursing, Ontario Canada

Background: Public health decision makers need to be able to easily access evidence in order to apply research to policy and programming decisions. This is of the utmost importance in times of crises when resources become additionally strained, but decisions still need to be made quickly. As COVID-19 pandemic evolved, decisions were also made based on rapidly evolving evidence, of varying quality. In response to this challenge, we developed a Rapid Evidence Service, building on internationally accepted rapid review methodologies, to synthesize the best available evidence on priority COVID-19 public health questions for local, regional and national public health decision makers.

Description: Our process involves: framing prioritized topics received from public health decision makers into searchable questions; developing and conducting a comprehensive search; rapid screening for relevance and minimizing duplication of efforts; critically appraising relevant evidence using validated tools; summarizing key findings from individual studies; GRADEing the evidence; and synthesizing the findings into a final report that includes an

overall summary and key messages along with the certainty of the findings, and an overview of remaining knowledge gaps. Our team includes members with specialized expertise in each of these areas and we collaborate with content-specific experts, as needed. We complete and disseminate most rapid reviews within one to two weeks. We have improved the efficiency of our process by using collaborative systematic review technologies and building upon already completed syntheses where available.

Conclusion: We have conducted more than 50 rapid review or review updates in 9 months. We continue to address new priority questions and update some of our completed reviews, as new evidence emerges. Our process has already evolved to ensure feasibility, accuracy, and efficiency as the pandemic and its evidence landscape changes. While we are planning a formal evaluation of our process and its impact, preliminary feedback has shown that this service is helpful, timely, and informative. Our process is one real-world example of how review-level evidence can be mobilized – rapidly, reliably, and needs-driven – during such unprecedented times.

Cultural Humility in a Multi-Institutional Network of Libraries: A Move Towards Equity

Practice Area: Professionalism & Leadership

Zoe Unno, Education and Outreach Librarian, NNLM PSR, Los Angeles, California

Nora Franco, Consumer Health Librarian, Network of the National Library of Medicine Pacific Southwest Region / UCLA Biomedical Library, Los Angeles, California

Carolyn Martin, MLS, AHIP, Consumer Health Coordinator, Network of the National Library of Medicine, Pacific Northwest Region, Seattle, Washington

Tess Wilson, MLIS, MFA, All of Us Community Engagement Coordinator, Network of the National Library of Medicine, Middle Atlantic Region, Pittsburgh, Pennsylvania

Tony Nguyen, MLIS, AHIP, Executive Director, Network of the National Library of Medicine, Southeastern/Atlantic Region, Baltimore, Maryland

April Wright, Community Engagement Coordinator, University of Maryland, Baltimore, NNLM/SEA Regional Medical Library, Baltimore, Maryland

Background: Addressing health disparities and inequities requires an understanding of the diverse communities that healthcare organizations and the institutions and community groups that collaborate with them. Health sciences libraries are included in this group and contribute to eliminating the disparities in their work with providers, community-based organizations, and consumers.

Description: A multi-institutional network of libraries formed a Cultural Humility Advisory Team (CHAT) to advise on matters of strategy and policy pertaining to the efforts to embody, build capacity, and respond to issues and challenges around cultural humility, equity, and inclusion within the institutions through their outreach, products, and services. The team members’ work included an exploration and understanding of existing and ongoing organizational efforts; identification of micro-aggressions and micro-assaults; solutions to efforts around cultural humility, equity and inclusion; and recommendation and implementation of training resources and programs. The impact was assessed by resource usage and training attendance.

Conclusion: This talk will present the inaugural year efforts and successes towards reducing heath disparities and inequities.

Designing and Curating a Database of Software Tools as a Case Study of Embedded Librarianship

Practice Area: Information Services

Wladimir Labeikovsky, Bioinformationist, Strauss Health Sciences Library, Aurora, Colorado

Background: Liaison and embedded librarianship are often defined vaguely largely due to the diverse nature, circumstances, and requirements of different patron and academic settings. Concrete examples of librarian/researcher collaborations can therefore be helpful for librarians looking to increase or adapt their outreach efforts in their own settings. This talk describes a collaboration between the Strauss Health Sciences Library and the Biostatistics Department of the Colorado School of Public Health in crafted a specialized information resource for the Metabolomics research community.

Description: The task (funded through a NIH consortium grant supplement) was to create a curated database of metabolomics software tools. The collaboration between domain experts and bioinformationists involved solving associated problems and iterating as well as embedding the librarian in the relevant research group. Many lessons were learned, as this project was a first for all involved.

Conclusion: The information resource is now in production and curation and documentation are ongoing. Future collaboration between the library and the research groups is now set up for both enhancement of the information resource as well as ongoing data management issues that the research group will face not directly related to the original project. Thus, this project-based collaboration resulted also in a continued liaison.

Developing Well Rounded Patrons through the Arts: Three Library Initiatives to Integrate the Humanities into our Health Community

Practice Area: Information Services

Christopher Hooper-Lane, AHIP, Director, Ebling Library, University of Wisconsin, Madison, Wisconsin

Lia Vellardita, Health Sciences Library, Ebling Library, UW-Madison, Madison, Wisconsin

Background: There is a growing recognition of the benefits of expanding the arts and humanities in the health sciences with evidence indicating engagement in the arts outside of the classroom or clinic impacts empathy, helps broaden views, and enhances the education and patient care experience. Recognizing this, school leadership tasked the Ebling Library at the University of Wisconsin to develop initiatives to connect the arts and humanities to our patrons. As a result, our library launched three new programs to integrate the arts into our community: a book/film discussion club; an onsite recreational reading collection; and an online arts journal.

Description: The Ebling Library led in the development and integration of three arts initiatives for our health sciences community: (1) a book/film discussion club (and corresponding website) providing participants at all levels a venue to discuss current and classic books and films on topics such as diversity, inclusion, racism, anti-racism, and well-being; (2) an onsite recreational reading collection (and corresponding website) offering patrons an accessible and browsable collection of popular non-fiction, fiction, poetry, and graphic medicine books focusing on topics in health and science, and includes sub-collections on emergent issues such as, diversity, antiracism, and well-being; (3) an arts/humanities journal that acts as a forum for our health community (faculty/clinicians, staff, and students) to showcase a variety of arts including the written word, visual arts (2D and photos of 3D art), and multimedia audio/video.

Conclusion: All three initiatives have been successfully launched. The book/film club to date has administered 14 online book/film discussions with a total of 210 participants. Sessions have been led by a variety of facilitators from our health community. The recreational reading collection is currently being established and now includes 450 items and is expected to be fully on display and available for circulation summer 2021. A corresponding website has been built using book cover images that can be sorted by sub-collection and each book cover includes information on shelf availability and links to relevant websites. Two issues of Corpus Callosum, our arts and humanities journal, have been successfully published with 52 works of art and a call for the third upcoming issue has generated 51 submissions. After the publication of our Fall 2020 issue, the journal website has recorded over 560 site visits. In addition to the online journal issues, special events are being planned to showcase works of visual, written, and audio art currently published in the journal.

Graduation Stops for Nothing: Pivoting to Develop an Online Interdisciplinary Seminar During Pandemic Stay at Home Orders

Practice Area: Education

Amy L. Corder, Research Support Librarian, Rudolph Matas Library of the Health Sciences, New Orleans, Louisiana

Laura Wright, MLIS, MPH, Research Support Librarian, Rudolph Matas Library of the Health Sciences, New Orleans, Louisiana

Keith Pickett, MLIS, Coordinator, Research Services and User Resources, Rudolph Matas Library of the Health Sciences, New Orleans, Louisiana

Background: On the first day of remote work due to COVID-19, the health sciences library received a request to develop an online interdisciplinary seminar for third- and fourth-year medical students. The goal of the interdisciplinary seminar program is to give medical students a well-rounded education by providing instruction that expands on the current curriculum, and it is included in graduation requirements. Three librarians discussed the informatics education needs for this population and created a seminar called “Research for Residency”. The seminar was created to fill the immediate need for online medical student instruction during stay-at-home orders.

Description: Planning for the IDS seminar began immediately. Consideration was taken to which skills would be most necessary for students beginning a residency program in the early days of a global pandemic. The seminar took place on April 8, 2020 via Zoom. The first portion of the seminar discussed the concepts of evidence-based medicine, general searching tips, and critical appraisal. An activity in which participants compared the information found in a clinical resource versus a Google Search followed. The second portion of the IDS seminar focused on productivity tools with an overview of EndNote X9 and Covidence. The third portion of the IDS seminar centered on research metrics including Journal Impact Factor, h-index, and altmetrics. Students were given an activity to find the three highest-impact journals in their field of choice using Web of Science.

Conclusion: Not only did this course help fulfill graduation requirements for students, it allowed the library to insert library-based research support content into the final months of the medical school curriculum (right before students begin residency). Plans are being developed with the Office of Medical Education to further integrate this content into the full four-year medical curriculum.

Distilled by the Library: The COVID-19 Resource Desk

Practice Area: Information Services

Carrie Grinstead, AHIP, Regional Librarian, Providence, Burbank, California

Danielle N. Linden, AHIP, Medical Library Manager, Burlew Medical Library / System Library Services, Orange, California

Heather J. Martin, AHIP, Director of System Library Services, Providence Health, Portland, Oregon

Background: In February 2020, library staff began sending daily PubMed alerts on the novel coronavirus to physicians, administrators, and other members of a large US health system. As the pandemic spread, we realized we needed to coordinate our approach. Senior administration soon requested help in “separating the wheat from the chaff,” to ensure that clinicians and researchers could efficiently stay abreast of the sheer amount of new research appearing daily. In response we launched a weekly collection of curated research and guidelines we called the COVID-19 Resource Desk.

Description: Library staff developed a PubMed algorithm to capture a full scope of COVID-related topics, from pre-clinical research to patient care, ethics, and public health. From hundreds of daily results, one librarian manually removes articles that do not directly focus on COVID-19 but rather discuss “____ in the time of COVID-19” or are otherwise out of scope. A small set of key articles are then identified to be included in the Resource Desk. In addition to PubMed results we include important preprints, news, and link to updates from the WHO, CDC, and FDA.

A small team uses a streamlined process that ensures manageable workloads and consistency during staff time off. This approach also allows us to engage several perspectives as we select content for the Resource Desk, while minimizing the conflict and delays that could result from a larger working group.

Conclusion: We recognized early that we were one of many groups – within and beyond libraries – who were curating breaking information on COVID-19. We added value to our Resource Desk by highlighting work by authors affiliated with our organization, and we increased visibility by promoting the Resource Desk through multiple channels: our website, the weekly Clinical Huddle, and direct emails to users who have opted in.

Our flexible, efficient model has allowed the Resource Desk to stay useful and relevant through every phase of the pandemic. The Resource Desk is downloaded consistently each week and further distributed via email by clinical leaders. As of February 2021 we continue to receive enthusiastic responses from high-level administrators.

Efficient ways to identify methodological search filters: developments with the InterTASC Information Specialists Sub-Group Search Filter Resource

Practice Area: Information Services

Carol Lefebvre, Independent Information Consultant, Lefebvre Associates Ltd, Oxford, England 12:00:00 AM

Julie M. Glanville, Independent Consultant, Glanville.info, York, England United Kingdom

Paul Manson, Information Specialist, Health Services Research Unit, University of Aberdeen, Aberdeen, Scotland United Kingdom

Sophie Robinson, Information Specialist, PenTAG, University of Exeter, Exeter, United Kingdom

Naomi Shaw, Information Specialist, PenTAG/HS&DR, ESMI, University of Exeter, United Kingdom

Background: Search filters, to find studies of a specific design, are an essential tool in systematic searching for studies for evidence syntheses such as systematic reviews. Finding search filters efficiently and deciding which ones to use are key aspects of literature searching for evidence syntheses.

This lighting talk describes the InterTASC Information Specialists' Sub-Group (ISSG) Search Filter Resource, which has been available since 2006, and outlines current developments that are being made to the site (https://sites.google.com/a/york.ac.uk/issg-search-filters-resource/home).

Description: The Search Filter Resource was developed by information specialists supporting evidence synthesis projects for the UK National Institute for Health and Care Excellence. It is maintained by information specialists who conduct regular searches of databases, journal tables of contents and websites to identify reports of new methods filters. As well as primary studies, the website also records performance reviews which are the most useful way to assess the relative performance of a range of filters. Following a recent revamp of the look and feel of the website, the resource is now being developed further with the inclusion of additional value-added information such as links to launch search filters in PubMed, links to comments and errata, highlighting comparative performance data visually and guidance on reporting filters. The Resource is also being actively disseminated on email lists and social media.

Conclusions: The Search Filter Resource has been providing access to methods search filters since 2006 to facilitate the use of search filters in evidence synthesis. Recent and ongoing developments to the website will make it an ever more useful and efficient way to identify filters

Engaging with evidence in languages other than English: reflections on researchers' priorities

Practice Area: Information Services

Kate Nyhan, Research and education librarian for public health, Cushing / Whitney Medical Library, Yale University, New Haven, Connecticut

Lauge Neimann Rasmussen, Knowledge broker, Educational Department at Steno Diabetes Center Copenhagen, Denmark

Background: In everyday literature searching and in formal evidence synthesis, evidence written in languages other than English (LOE) can play an important role. The fact that many multilingual scholars also publish in English could give Anglophone searchers and readers confidence that monolingual searches and workflows are sufficient. LOE evidence should ideally be treated as systematically and seriously as Anglophone evidence but everyone is limited by their language skills. How can information seekers deal with this challenge, and how can librarians advise them?

Description: We briefly explore best practices for engaging with LOE documents:

- choosing information sources which index them

- creating search strategies which retrieve them

- reading and appraising them

We also outline barriers that reviewers experience when engaging with LOE evidence. We then reflect on our experiences helping information seekers decide whether and how to engage with LOE evidence, and how to report their decisions.

We will discuss examples of information needs in clinical research and public health research. The decisions that researchers made in these cases were based on our counsel, their language skills, their resources, and their perceptions of LOE scholarship. We discuss how researchers navigated the benefits and limitations of trying to include LOEs in the presented cases.

Additionally, we provide a public Zotero bibliography of relevant methods papers at https://bit.ly/LOEevidence.

Conclusion: By sharing experiences -- successes and failures -- we will give listeners more tools to discuss the value and challenges of LOE evidence with information seekers. As information experts, we must understand not only the reasons to engage with LOE evidence and the techniques to engage with LOE evidence effectively, but also the priorities and needs of the information seekers whom we advise. Taking their priorities and needs into account lets us give more tailored advice and better support information seekers in engaging meaningfully with LOE evidence.

How Generating PICO and Clinical Questions from PubMed Abstracts Challenges Instruction of PubMed Search Strategies to First-Year Dental Students

Practice Area: Education

Peter C. Shipman, Dental Medicine and Cancer Librarian, Robert B. Greenblatt, MD Library / Research and Education, Augusta, Georgia

Amanda Nevius, MLIS, Research & Instruction Librarian - Dental Liaison, Hirsh Health Sciences Library, Boston, Massachusetts

Background: To use PubMed abstracts to illustrate PICO (Problem, Intervention, Comparison, Outcome) elements and then rank the PICO elements by importance when introducing PubMed database search strategies to first-year dental students.

Description: First-year dental students are naïve to the evidence-based dentistry (EBD) process, learning PICO elements, generating clinical questions from PICO, assigning question concepts to PICO categories, and planning and executing a search of PubMed from PICO. The director of the EBD course demonstrated how to identify PICO elements and generate clinical questions derived from eight preselected PubMed abstracts. In a subsequent class the dental librarian reused the abstracts to demonstrate how to create search terms from concepts, verify the accuracy of the PubMed translation of search terms and to combine searches from the search history with Boolean operators. The search strategy is derived from ranking the two or three most important concepts to search, searching individual concepts, and combining concepts from the search history with the correct Boolean operators.

Conclusion: More examples of clinical questions and PICO can be created from PubMed abstracts so students have more hands-on practice and repetition to identify PICO concepts, create search terms, verify PubMed automatic term mapping and search translations, and combine saved searches with Boolean operators. Ranking concepts by importance is simpler for new learners than assigning concepts to PICO categories. Combining concepts from a ranked list with Boolean operators is easier than applying Boolean operators to PICO categories.

I start rounding tomorrow. Do I need to know Greek?

Practice Area: Information Services

Shawn Steidinger, AHIP, Assistant Librarian for Clinical Services, Eccles Health Sciences Library, Salt Lake City, Utah

Background: How do you prepare for that first time rounding with clinicians? What if you do not have a science background or your only medical experience is as a patient? What if you could attend a “mini med school” to familiarize yourself with medical terminology, physiology, anatomy, pharmacy, nursing theory – wait! Let’s just start with the medical lingo that you might hear at the bedside or in the hospital halls.

Description: A mini med school for new medical librarians should teach you the basic prefixes, roots, and suffixes that make up the medical terminology that you will hear spoken among clinicians and noted in the medical record. Having a rudimentary understanding of value ranges for body temperature, pulse rate, respiration rate and blood pressure as well as common labs that patients undergo is handy when clinicians present you with their clinical questions and search requests. Refreshing your memory on the Greek and Latin vocabulary that you last used when studying for SAT/ACT tests will boost your comprehension as you search medical literature databases and scan article abstracts as well. Mini med school modules could be housed within the MEDLIB-Ed platform, and short quizzes and check-ins could be sent out monthly to reinforce deep learning.

Conclusion: A mini med school would benefit new medical librarians in many settings: hospitals, dental clinics, pharmaceutical firms, as well as academic health sciences. Lab values, anatomical terminology, and abbreviations found in clinical notes are not commonly taught in library science programs. Educating librarians on these topics could increase our value as members of a clinical team and make our searching more relevant to our requesters.

Laptops for Students of Migrant Farmworkers During a Pandemic

Practice Area: Global Health & Health Equity

Roger Russell, Associate Director, Laupus Library, Greenville, North Carolina

Mary Roby, Assistant Director for User Services, Laupus Health Sciences Library, Greenville, North Carolina

Elizabeth Ketterman, Director, Laupus Health Sciences Library, Greenville,

Jamie E. Bloss, AHIP, Liaison Librarian, East Carolina University, Greenville, North Carolina

Background: The COVID-19 pandemic highlighted existing structural vulnerabilities for migrant farmworker families, including profound inequities in educational access. In a previous NLM funded pilot project, we partnered with faculty from our university's Health Education and Promotion program, and local migrant assistance programs in one county to provide tablets, hotspots, and information literacy training. Half of the middle and high school participants in this pilot project had no internet access at home. Based on the data and lessons learned from this previous project we sought funding for a complementary project to extend impact and benefits to our regional migrant farmworker community.

Description: This project is made of three components: Providing laptops for migratory students, increasing information literacy through training, and disseminating an offline coronavirus and educational resource library with the laptops. Laupus library received CARES act funding to purchase 100 laptops. We partnered with state Migrant Education Program (MEP) staff in 4 counties, and with local Student Action with Farmworkers (SAF) group to both coordinate distribution of the laptops where they were most needed, and to develop the offline library. Our librarians developed and delivered health information literacy training for MEP and SAF staff. We further partnered with faculty in our university's Health Education and Promotion department to coordinate evaluating the effectiveness of the project through focused interviews with students, families, and teachers.

Conclusion: We plan to measure the qualitative impact of providing students with laptops in relation to their access to education, and their perception of success in learning through conducting a series of focus groups and one on one interviews with students, families, and teachers. These evaluative efforts will be coordinated by faculty partners in our university's Health Education and Promotion program.

Librarian contributions to a Manuscript Writing Intensive for health researchers

Practice Area: Information Services

Elizabeth Suelzer, AHIP, User Education and Reference Librarian, Medical College of Wisconsin, Milwaukee, Wisconsin

Background: In early 2020, an experienced academic writing coach developed a 10-week Manuscript Writing Intensive to support the dissemination efforts of the health researchers in our consortium of eight local institutions. The objective of this hands-on small group course was for participants to use the weekly two-hour sessions and 4 hours of homework to support each other in completing a publishable research report within the span of the course. The instructor contacted me, a medical librarian, for assistance and to explore opportunities for collaboration. This was exciting as there was growing interest to expand the library’s support for scholarly communications.

Description: The instructor met with me for an orientation to the library resources and staff expertise available for participants. We then met again to brainstorm segments of the course that I could lead. The planned in-person course pivoted to online when the COVID 19 epidemic emerged. I developed one-hour virtual presentations for the first two sessions that covered ORCID, citation managers, finding the right journal, and literature searching. Each presentation was followed by a discussion period where participants shared their publishing experiences and asked questions. I repeated this for all four cohorts. Overall course feedback highlighted the value of these librarian-led sessions. Even the course instructor commented that my presentations brought her up to speed on available resources to guide the manuscript writing process.

Conclusion: With the first iteration of the course deemed a success, plans were made to offer the series quarterly and expand the number of cohorts. To accommodate the increased number of sessions, I produced videos of my presentations as foundational information for assigned homework. The instructor also designed a worksheet based on my presentation on finding the right journal. These videos are posted on YouTube and our LibGuides, and are included in the course materials. Library collaboration on this dissemination initiative accelerated the development of the course, promoted participant learning, and showcased the expertise of librarians. With a call to develop a Grant Writing Intensive later this year using this same model, the instructor has contacted me to continue our collaboration.

Librarian Integration in the Development of Medical Simulation Experiences - a Case Study

Practice Area: Innovation & Research Practice

Darell Schmick, AHIP, Director of Library Services, Noorda-COM Library, ,

Maria E. Vazquez-Amaral, Assistant Dean, Medical Simulation, Provo, Utah

Background: There is no significant historical information regarding collaboration between medical simulation departments and libraries. In the development of a series of new scenarios for medical students, Medical Simulation Leadership and Library Leadership have partnered together to develop an interprofessional workflow for the institution.

Description: The checklist for creating a high-quality medical simulation scenario is known as the Duke Template (part of the Intellectual Property Commons): a protocol introduced by Duke University that has become the gold standard for medical simulation creation in immersive clinical settings. Medical Simulation Leadership and Library Leadership seek to proactively address a dearth of access by faculty scenario creators to refer to all assets available to them at their institutions. The creation of a modification to the Duke Template, one that includes the consultation of a librarian when developing student resources for clinical scenarios for simulation, has been introduced and will be a part of the checklist moving forward.

Conclusion: The collaboration will keep account of deliverables from the consultation with the librarian, which will be recorded by both Medical Simulation and Library leadership on this novel partnership.

Library Support for Health Justice Capitol Hill Day: Empowering Medical Student/Law Student Partnerships with Evidence

Practice Area: Global Health & Health Equity

Emily Alagha, AHIP, Clinical Information Specialist & Data Management Coordinator, Dahlgren Memorial Library, Georgetown University Medical Center, Washington, DC

Background: Medical-legal partnerships add lawyers to healthcare teams to increase patient access to legal services that improve health. The Capitol Hill Day project at the Georgetown University School of Medicine provides second-year medical students with the opportunity to gain the type of interprofessional advocacy experience supported by medical-legal partnerships. Students work in teams with law students to prepare for meetings with political representatives on health justice policies of their choosing. The program helps students experience the role doctors can play as health advocates beyond the patient level. The objective of embedding a medical librarian in the program is to strengthen the quality of evidence used in student advocacy efforts.

Description: While preparing for meetings with their representatives, students received guidance from faculty experts in medicine and law. The 2020 students were the first cohort to have the additional support of an embedded medical librarian available both on Canvas and in person at planning sessions. The embedded librarian delivered an information literacy presentation about finding evidence for health advocacy at the first session, and she facilitated small group discussions in two subsequent meetings. She also created a health justice Libguide that compiles evidence and data on the most popular student advocacy topics. The students produced an evidence-based advocacy handout and generated talking points to support their discussions with representatives. Capitol Hill Day meetings took place in March 2020. The program was assessed via student survey, and library support will continue for future cohorts with an increased emphasis on virtual outreach opportunities.

Conclusion: Embedding librarians within student advocacy projects provides new opportunities to support student development of evidence-based research skills. Health justice collaborations also provide libraries with the opportunity to support organizational social justice initiatives that have a lasting impact on community health.

Making the Leap from Newsletter to Blog: Lessons Learned

Practice Area: Professionalism & Leadership

Erin E. Reardon, AHIP, Medical School Librarian, University of Minnesota Health Sciences Library, Minnesota

Olivia Glotfelty-Scheuering, MLIS, EBP (CH), Manager, Library Services, UPMC Mercy, Pittsburgh, Pennsylvania

Background: For over 40 years, the Hospital Library Caucus has published a newsletter for its members on a regular basis. This newsletter has historically featured messages from the

caucus chair, editorials, information about MLA meetings, and other articles on topics of importance to hospital librarians. In 2020, the newsletter editors made the decision to take the next evolutionary step and transition our newsletter into a blog. Our objectives were to modernize the newsletter’s distribution, capture important conversations from the caucus listserv, and recognize the accomplishments of our caucus members on a more regular basis.

Description: Originally mailed to members, by 2020 our newsletter was being distributed quarterly as a PDF file via our caucus listserv. The editors and the caucus board suspected this was no longer serving the needs of our caucus, so we surveyed the members. Based on the feedback we received and the desire to modernize the newsletter content and format, we moved forward with launching a blog on our internal caucus portal on MLANET. Neither editor had experience managing a blog, so we consulted several resources for guidance. We reached out to other caucuses who used the blog function and MLA employees for instruction on using the blogging platform. We used past newsletter content and article length to shape our guidelines for what content the blog would contain, and we consulted best practices for social media to determine a posting schedule.

Conclusion: Evaluation of our blog’s impact is ongoing. For MLA ’21, we can compare self-reported readership from our survey and download clicks from past PDFs of our newsletter to six months of data on community interaction with our blog via view counts on our blog’s landing page and individual articles and comments on our posts. While we are more limited in what statistics we can see in the internal MLANET platform than we would be on an external blogging platform, the internal system is still the best choice for our caucus. We hope that our experiences can help other communities evaluate the communication needs of their members and launch their own blogs if they so desire.

Menstrual Equity for $22 a month: How one health sciences library brought free menstrual products to an entire university

Practice Area: Professionalism & Leadership

Donna Baluchi, Library Supervisor, Spencer S. Eccles Health Sciences Library, Salt Lake City, Utah

Background: Menstrual equity is easier than you think! This lightning talk will supply the audience with the data, budget, & discussion points in order to implement a similar program within any institution (public, academic, nonprofit, etc). This also presents menstrual equity as an essential part of DEI initiatives in order to remove the sexism and stigma around menstruation and provide cost-prohibitive health products to our communities.

Description: Health products necessary for menstruation are regularly criticized for being prohibitively expensive and inaccessible. It is not uncommon for those that menstruate to be surprised by their period starting. The health sciences library at a large university took year-

end unused funds to purchase menstrual products which would be freely available in all library bathrooms. After 18 months, and 250,000 patrons visiting the library, the cost to supply them averaged $22 per month. Outreach resulted in the campus Office of Diversity making menstrual products freely available within every bathroom on campus. This lightning talk will provide everyone with tools to advocate menstrual equity at any institution. Everyone should have the right to care for themselves easily and without shame, and libraries have the chance to be at the forefront of this movement.

Conclusion: The $22 per month average is based on the purchase of tampons, pads and liners in bulk and reflects pre-COVID budgets and library patron numbers. Thanks to outreach efforts, the cost for the menstrual products is no longer the responsibility of the library and supplied via campus facilities. Added efforts have led to the city making them freely available in public buildings and the airport. This success is now being followed up with a student-led grant proposal to make menstrual cups available for minimal cost to all students, staff or faculty interested in a more sustainable menstrual alternative.

NIH Data Management & Sharing Plans: Examining the 2023 DMSP structure

Practice Area: Innovation & Research Practice

Nina Exner, Ph.D., Research Data Librarian, Virginia Commonwealth University, Richmond, Virginia

Janice M. Hermer, MLIS, Health Sciences Librarian, ASU Library, Phoenix, Arizona

Douglas L. Varner, MS, MLS, AHIP, Assistant Dean for Information Management, Dahlgren Memorial Library, Washington, District of Columbia

Background: Late in 2020, the NIH released the Final NIH Policy for Data Management and Sharing (https://grants.nih.gov/grants/guide/notice-files/NOT-OD-21-013.html), to be effective in 2023. The presenters worked on a template for DMPtool to connect the Elements of an NIH Data Management and Sharing Plan (DMSP) with related guidance from other documents in one landing page. To prepare the new template, we needed to analyze the new Final Policy documents to extract key information for creating a template that all researchers could use. We would like to share what we learned about the new DMSP structure.

Description: The Final NIH Policy for Data Management and Sharing includes significant new requirements designed to facilitate research data management practices consistent with the FAIR principles. One difference between current NIH data sharing plans and the forthcoming ones is the structure. The new guidance requires more topics to be covered in the DMSP.

Researchers and research administrators often find it useful to have templates that combine the DMSP layout with relevant guidance in one place, which is why DMPTool provides a walkthrough of these plans. Immediately following the Final NIH Policy announcement, the DMPTool team received requests to develop a new template aiding researchers in adhering to the new policy. The DMPTool Editorial Board immediately began work developing this new template. This lightning talk reviews the new DSMP layout and other key guidance we identified for each DMSP section.

Conclusion: Data management (and sharing) plans depend on combining the DM(S)P format with related guidance and best practices. Combining these is not obvious from a brief examination of the policies, which is why a template tool is useful. This session will provide an overview of how the forthcoming DMSP sections combine with the related “Final Policy” guidance and guidance on best practices for RDM, in order to help attendees start thinking about their patrons needs in writing DMSPs for the forthcoming policy.

Nursing Student Success in an Online Learning Environment

Practice Area: Education

Jennifer McKay, Nursing Librarian, Alaska Medical Library, UAA/APU Consortium Library, University of Alaska Anchorage, Alaska

Background: Prior to March 2020, a hybrid state-wide undergraduate RN to BSN nursing program collaborates with a medical librarian at the University of Alaska Anchorage (UAA) to ensure that evidence-based practice learning outcomes are met. Second, third, and fourth-semester students complete carefully scaffolded assignments online in their Learning Management System (LMS) to ensure success in locating, evaluating, and ethically using information.

This lightning talk will describe specific instructional methods and how they were adapted to meet the needs of students who were abruptly moved to an entirely online learning environment.

Description: Students who complete the RN to BSN program are provided multiple opportunities to practice locating, evaluating, and ethically using information. Students are first introduced to the UAA/APU Consortium Library's nursing librarian and the topic of information usage rights by participating in discussion board posts within their LMS. Next, students become familiar with terminology related to searching, information access, and management/storage. These strategies culminate in a fourth-semester group assignment where students apply their knowledge to a final presentation on a research topic of their choosing.

Pre-pandemic, students completed these librarian-graded assignments online and were supplemented with Guide-on-the-Side tutorials, in-class instruction, and regular in-person office hours. Since the pandemic began, the librarian has seen improved student success with the librarian recording herself searching databases via Zoom, clearer rubrics, spreading assignments out over multiple units, and offering drop-in Zoom office hours.

Conclusion: Assignment metrics have shown that learning outcomes have improved over the last four semesters from March 2020 to the present. Also, connections with students have greatly improved through increasing the ways in which students can connect with the nursing librarian.

Preserving Primary Sources from Opioid, Tobacco, and Other Industries to Understand and Address Commercial Determinants of Health

Practice Area: Information Management

Kate Tasker, Industry Documents Library Managing Archivist, University of California, San Francisco Library

Background: This presentation examines a digital archives program to collect and describe opioid industry documents which are publicly released in litigation and preserve them for long-term public access and research. The program builds on a successful model to preserve tobacco industry documents, which since 2002 has supported over 1,000 publications about industry influence on health and led to groundbreaking change in national and global tobacco control laws and policies. The opioid industry and opioid epidemic are of widespread concern to health sciences librarians, as evidenced by education and public policy work by MLA, NLM, AAHSL, and other associations.

Description: The program brings together librarians, informationists, digital archivists, software developers, historians, and health scientists at multiple institutions, with the aim of developing a freely accessible digital archive of millions of opioid industry documents which can be used to learn from the opioid epidemic in order to improve and safeguard the public health. The archive currently includes over 3,300 opioid industry documents (over 131,000 pages) which can be cross searched with other collections of previously confidential materials from the tobacco, chemical, food, drug, and fossil fuel industries, including memos, reports, internal scientific studies, marketing and sales information, and public relations campaigns. These materials are used by scientists, journalists, lawyers, policymakers, and other stakeholders to investigate industry influence on public health. The program provides research and education services and also prepares data for digital health humanities and computational analysis projects.

Conclusion: This presentation will describe current program goals, metadata and digital preservation considerations, outreach, anticipated evaluation methods, and collaboration opportunities. Expected outcomes include: 1) public access to potentially tens of millions of opioid industry documents; 2) long-term digital preservation of industry documents; 3) increased support for scholarship on subjects including commercial determinants of health, pain management, addiction, conflicts of interest in the pharmaceutical and healthcare industries, health policy, and digital health humanities.

PRISMA-S: An Extension to the PRISMA Statement for Reporting Literature Searches in Systematic Reviews

Practice Area: Information Services

Melissa L. Rethlefsen, AHIP, Executive Director, Health Sciences Library & Informatics Center / University of New Mexico

Ana Patricia Ayala, AHIP, Research services librarian, Gerstein Science Information Centre, Toronto, Ontario   Canada

Shona Kirtley, Senior Research Information Specialist, EQUATOR Network, Centre for Statistics in Medicine, Oxford, England United Kingdom

Siw Waffenschmidt, Institute for Quality and Efficiency in Health Care, Head of unit, Information Management Unit, Cologne, Germany

Jonathan Koffel, Emerging Technology and Innovation Strategist, University of Minnesota Health Sciences Library, Minnesota

Background: PRISMA-S was developed over five years using a robust, consensus-based methodology inclusive of internationally representative librarians and information specialists, systematic review methodologists, journal editors, and PRISMA 2020 leadership. Released in 2020 and formally published in 2021, PRISMA-S builds upon the PRISMA reporting guideline and is designed as an international standard to guide transparent, reproducible reporting of literature searches in systematic reviews and other methods-based reviews.

Description: The heart of PRISMA-S is a 16-item checklist detailing what must be reported in a manuscript to fully and transparently report literature searches. The 16 items are categorized into four sections: Information Resources and Methods, Search Strategies, Peer Review, and Managing Records. To further detail what entails complete reporting for each item, an explanation and elaboration section includes, for each item, examples of good reporting taken from the literature, detailed explanations of why the item is important, further in-depth guidance, and the suggested location(s) for reporting. Because the needs of individual literature reviews vary greatly, not all PRISMA-S items may be needed for all reviews, but PRISMA-S provides guidance for the most commonly used search methods.

Conclusion: PRISMA-S provides a consensus-driven international standard for literature search reporting. PRISMA-S is highlighted within the newly released PRISMA 2020 Statement, giving it additional visibility. Using this new reporting guideline will enable librarians and information specialists to report reproducible, transparent searches for systematic reviews and other methods-based literature reviews. Next steps include working with journal editors to increase endorsement and recommendation of PRISMA-S. PRISMA-S is available at: https://doi.org/10.1186/s13643-020-01542-z.

Reaching Everyone: Accessibility in Library Email Marketing

Practice Area: Information Services

Claressa Slaughter, Outreach and Instruction Librarian at Rosalind Franklin University, Rosalind Franklin University of Medicine and Science Boxer Library, , Illinois

Charlotte Beyer, AHIP, Library Director, Rosalind Franklin University of Medicine and Science, North Chicago, Illinois

KatieRose McEneely, Electronic Resources Librarian, Rosalind Franklin University of Medicine and Science, North Chicago, Illinois

Chelsea Eidbo, Access Services Librarian, Rosalind Franklin University of Medicine and Science, Waukegan, Illinois

Background: The purpose of this lightning talk is to describe one library’s process for evaluating the accessibility of the library’s email marketing materials using principles of universal design for learning. This lightning talk will focus on why accessibility is important by giving easy tips to help libraries evaluate their own email marketing materials for accessibility.

Description: In 2020, the library’s marketing and outreach committee had a discussion about accessibility in the library’s marketing materials, specifically those sent via email during COVID. This initial conversation led to questioning if materials sent via email, such as infographics, could be read with screen readers and how we could use the universal design principles to see if materials were accessible.

After applying universal design principles, it was identified that infographics which were a fun and artistic way for outreach had a few issues in terms of accessibility. The committee then brainstormed ways to make them more accessible which we would apply to future materials such as accompanying email images with accessible word documents. This conversation led to the creation of a set of internal guidelines staff will use when creating email marketing.

Conclusion: With COVID and remote learning, email is the primary way the library can reach out to the university community. Marketing materials, such as infographics, can be a seemingly easy and fun way to convey information too, they are not always the most accessible medium. Using universal design principles when developing email marketing materials can help ensure that important library communication is accessible to everyone. The guidelines we developed during this committee will continue to evolve and change to suit our populations accessibility needs

Rapid Development of a COVID-19 Evidence Based Medicine Training Curriculum for a Frontline Primary Care Consultation Service

Practice Area: Clinical Support

Christopher Stave, Graduate/Clinical Education Librarian, Lane Medical Library, Stanford University, Stanford, California

Malathi Srinivasan, Clinical Professor of Medicine, Stanford University School of Medicine, Palo Alto, California

Bright Zhou, Medical Student, Stanford University, ,

Christopher Calkins, Fourth-year Medical Student, Stanford University School of Medicine, ,

Erika Schillinger, Vice Chief for Education, Primary Care and Population Health, Stanford University, California

Background: In March 2020, Stanford's clinicians faced the emerging COVID-19 pandemic with a panicked public and were overwhelmed by a rapid growth in unsynthesized medical literature about SARS-coV-2 biology, epidemiology, and treatment. In response, 16 medical/PA students and 12 primary care faculty mentors developed the Stanford Frontline Clinical Question Consult Service (SFCQCS), to provide evidence-based, peer reviewed answers to urgent COVID-19 clinical questions from frontline primary care providers. Collaborating with FCQCS, the Primary Care library liaison developed the curricular backbone of a series of online didactic sessions aimed at improving student’s ability to search, evaluate and synthesize COVID-19 related information.

Description: Within two weeks, we launched five didactic sessions focusing on COVID-19 resource identification, search strategy development, information alerts, evidence evaluation, and citation management. Evidence synthesis was challenging, as new information/misinformation was shared via pre-publication sites and news reports ahead of peer-reviewed publication. Students worked in seven content teams: Clinical Trials; Epidemiology; Healthcare Worker Safety; News; Testing; Symptoms; and Vaccines. Training was integrated with faculty-led critical appraisal workshops. In April 2020, Session 1 provided pre-designed COVID-specific search strategies for databases and other high-impact websites. Session 2 demonstrated three tools for keeping up with emerging literature: database alerts; e-TOCs; and RSS readers. Sessions 3-5 were hands-on workshops concentrated on improving the efficiency of reference capture, citing, and sharing via Zotero. In May, students/mentors and primary care faculty were surveyed regarding their program experience.

Conclusion: In three months, SFCQCS produced 87 evidence-based, peer-reviewed answers to critically important COVID-19 related clinical questions for primary care faculty. Through participation in library supported training, students were able to find and assess peer-reviewed and non-peer reviewed research more efficiently. 13 of the 16 SFCQCS students (81%) completed evaluation surveys with self-assessed achievement of SFCQCS learning objectives, including improved literature searching skills, exceeding 90% for all objectives. Students could also more effectively separate misinformation from accurate information about COVID-19. Lastly, through direct collaboration with the library, learners could more easily keep up with literature in their selected content area. The SFCQCS rapid program development, collaboration and success highlights the critical role of medical librarians in an academic clinical setting.

Reimagining Mentorship: A Virtual Mentoring Program for Medical Librarians

Practice Area: Professionalism & Leadership

Nell Aronoff, Associate Librarian, University at Buffalo Libraries/Health Sciences Library Services, Buffalo, New York

Heather S. Healy, AHIP, Clinical Education Librarian, University of Iowa Libraries/Hardin Library for the Health Sciences, Iowa City, Iowa

Emily J. Glenn, Associate Dean, McGoogan Health Sciences Library, Omaha, Nebraska

Background: Since 2003, the MLA Membership Committee facilitated an in-person mentoring program called Colleague Connection. Typically, a first-time MLA annual meeting attendee was paired with a more experienced attendee in order to help the first-time attendee navigate the meeting. The connection was focused on the one-time in-person meeting. Since the connection hinged on annual meeting attendance, members who could not attend were excluded. The virtual format of the 2020 meeting created an opportunity to rethink the Colleague Connection experience. Three members of the Membership Committee developed a virtual mentoring program in response to the format change.

Description: Promotion for the new virtual mentoring program began at the welcome event for MLA ‘20 in July 2020. The program was also advertised in MLAConnect, via the MLA Community Council, and through chapter listservs. For this version of Colleague Connection, new members could also opt to be paired with a peer instead of the traditional mentor-mentee pairing. Seventy-four new members and sixty experienced members signed up. Some experienced members mentored two mentees. Nine participants elected to be paired with fellow new members. Participants were encouraged to meet every month from October 2020 through January 2021. Colleague Connection coordinators sent monthly prompts to help provide structure to the mentoring process. Three pairs shared their experiences at a wrap up event hosted in January 2021. A survey was distributed to participants to gather feedback about the program.

Conclusion: Fifty-four percent of participants (n=73) completed the survey. Most pairs met four times and many indicated that they would continue to meet after the formal program ended. Open-ended comments included themes like communication, structure of the program, and expectations for participants. Based on feedback received from the survey and during the wrap up event, participants had a positive experience in the revamped Colleague Connection program and were glad to have to chance to connect with fellow MLA members. This more accessible mentoring model could be adopted by other groups and chapters within MLA.

A self-directed approach to improving liaison librarian services.

Practice Area: Information Services

Jeremy Gunnoe, MSLIS, Assistant Director of Operations/Medical Librarian, Howard University Louis Stokes Health Sciences Library, Upper Marlboro, Maryland

Background: There is a need to enhance and grow the library’s liaison services. While the library has librarian liaisons to the four medical colleges, these roles have traditionally acted in more of a support capacity. Services include performing orientation presentations, assisting the faculty with research, and some classroom involvement (when requested). This approach creates restrictions on the awareness of all of the services that the library can provide. To increase service offerings and enhance the liaison role, it is necessary to further engage with the individual colleges to allow the librarian to be more fully integrated into the college’s curriculum.

Description: The library supports four health sciences colleges and offers a liaison librarian to each. This program initially focuses on liaisons to the Colleges of Pharmacy and Nursing and Allied Health. Additionally, the Assistant Director wanted to offer the librarians ownership in the design and implementation of these programs. To improve the service offerings the librarians were each asked to perform literature searches to find examples of successful university liaison programs in their subject area. After compiling and reviewing this information, both librarians were then asked to create a plan and timeline for these programs which included SMART goals and established benchmarks. The liaison librarian to the College of Nursing chose to pursue a goal of becoming more embedded in the curriculum while the Pharmacy librarian chose to create a series of information literacy videos tailored to the College of Pharmacy.

Conclusion: It is expected that this program will allow the librarians to further engage with their respective colleges to enhance and grow the library liaison relationships.

Surfing the Health Sciences LIS Student Pipeline

Practice Area: Professionalism & Leadership

Zoe Unno, Education and Outreach Librarian, NNLM PSR, Rolling Hills Estates, California

Nora Franco, Consumer Health Librarian, Network of the National Library of Medicine Pacific Southwest Region / UCLA Biomedical Library, Los Angeles, California

Background: Diversity, Equity, and Inclusion (DEI) are hot topics and buzzwords in librarianship. These ideas for equitable work environments result in new committees or working groups tasked to explore and develop policies and programs to address these challenges. One way to remedy the inequities is to recruit and retain a diverse workforce. Health sciences libraries expand opportunities to diverse communities by training library and information science (LIS) students from groups that are underrepresented in health professions and biomedical research to pursue health sciences librarianship.

Description: A health sciences library created an award program to promote the value of diversity, equity, and inclusion in health sciences librarianship to library school students and

recently graduated health science librarians from Underrepresented in Biomedical Research (UBR) populations. The award provides funding for students to participate in meetings, conference sessions and other activities designed for them to learn the importance of health information outreach and services conducted by health sciences librarians. The awardees pursue an area of health librarianship that explores providing health information to underrepresented and underserved communities.

Conclusion: This talk will discuss the development of the award and highlight the experience of the first cohort.

Transforming Health Communication Instruction for Nursing Students through Information Literacy Classes

Practice Area: Education

Brenda M. Linares, AHIP, Health Sciences Librarian, School of Nursing, A.R. Dyke Library, University of Kansas Medical Center, Olathe, Kansas

Background: The nursing school liaison librarian taught three modules for an undergraduate course each fall semester for the last three years. In recognition of these efforts, the librarian was approved as a volunteer Educational Instructor to support collaboration and involvement in the class. The librarian-led sessions cover topics including Information Literacy, Searching and Scholarly Communications, and Health Literacy and Patient Education. The librarian has 1.5 hours for each module and is encouraged to incorporate relevant topics and issues within the instructional design. The modules include library resources, diversity and inclusion, cultural humility, and consumer and patient health resources.

Description: Building on the close relationship between the library and the School of Nursing, the liaison librarian reached out to faculty and noted that the Health Communication class (one of the first classes that juniors take) would benefit from incorporation of library resources and other areas of librarian expertise. The librarian started by teaching one module and over the years that participation has increased to three modules. The librarian incorporates slides and active learning techniques (e.g., PollEverywhere and role-play) to convey the importance of communication, research, and patient education. The techniques used will be outlined in this lightning talk. Due to this increased involvement, the librarian was recognized with a volunteer instructor appointment at the school. The professors of this class have shared the feedback they have received from students, which has been positive and led to continued participation.

Conclusion: Due to this increased involvement, the librarian was recognized with a volunteer instructor appointment at the school. The professors of this class have shared with the librarian the feedback they receive from the students, which has been positive and led to continued participation.

Transforming Our Diversifying Communities: Introducing the Global Health and Health Equity Domain Hub

Practice Area: Global Health & Health Equity

Linda M. Hartman, MLS, AHIP, Researcn Librarian, RQM+, Glenshaw, Pennsylvania

HelenAnn B. Epstein, MLS MS AHIP FMLA, Informationist, Virtua Health Sciences Library, Monroe, New Jersey

Background: In early 2019, MLA began its transformation. It was the aim to undue any silos within MLA. Special Interest Groups and Sections became Caucuses. 7 Domain Hubs were created and encouraged Caucuses to align with Domain Hubs. This abstract introduces the newly formed Global Health and Health Equity Domain Hub.

Description: This hub is pledged universally to issues of global health and health equity by fostering organizational and professional partnerships to improve the development, access, dissemination, and understanding of health information for and by diverse populations in the United States and globally to improve the health of populations." The hub’s professional practice area includes the development of health information professionals globally, equity in access to health information, and international collaborations for MLA and medical librarianship.

Conclusion: Ten caucuses have sent representatives. The Health Information Professionalism CE Committee also has sent a representative. The Hub plans to focus on medical racism and health equity worldwide. Also, it turns attention to environmental and/or climate justice and health equity with special concentration on disaster preparedness. Within the Healthy People 2030, the Hub concentrates on issues of health communications and health literacy. The Hub plans to reach out to the Librarians without Borders and the Cunningham Scholars. The Global Health and Health Equity is just getting started. It is vibrant dedicated group displaying diversity, equity and inclusion to all its members and to all its projects.

Using History of Medicine and Archival Collections to Celebrate Our Medical School’s 175th Anniversary

Practice Area: Education

Nell Aronoff, Associate Librarian, University at Buffalo Libraries/Health Sciences Library Services, Buffalo, New York

Matthew Gadziala, Assistant Librarian, University at Buffalo Libraries/Special Collections, Buffalo, New York

Background: The University at Buffalo was founded as a medical school in 1846. This year marks the 175th anniversary of the school and the university, which has grown exponentially since that time. In order to celebrate this milestone and draw attention to our History of Medicine Collection and University Archives, we created a guide using the LibGuides platform.

Description: The guide features four main tabs: Home, People, Places, and Resources for further exploration. The challenge was determining who and what to highlight given the long time span and variety of materials available. Our main sources for information included existing histories, archival photos, old yearbooks, the medical school’s magazine, and other online sources. Each tab has a similar banner and care was taken to make the guide visually appealing. To help draw peoples’ interest, the guide includes facts such as cost of attendance, the number of male and female students, and class “yells” (cheers). The guide was published on January 5, 2021.

Conclusion: Between January 5 and April 4, 2021, the guide was viewed 1,450 times. It was promoted on the medical school and general library listserv on January 5, 2021 and received 665 views that day. We have received positive feedback from medical school and library colleagues. The medical school's alumni and admissions offices were particularly interested in old photographs of the school and material for Black History Month in February. While the intent of the guide was to promote our History of Medicine Collection and University Archives, we have not seen a large uptick in email inquiries relating to these collections. We hope that as the guide is shared more widely through university, medical school, and library communication channels that this leads to more interest in using our special collections.

Using Little Free Libraries to Transform Access to Quality Health Information in Rural Communities

Practice Area: Information Services

Jane Morgan-Daniel, AHIP, Community Engagement and Health Literacy Liaison Librarian, Health Science Center Libraries, University of Florida, Gainesville, Florida

Margaret Ansell, AHIP, Nursing & Consumer Health Liaison Librarian, UF Health Science Center Libraries, Gainesville, Florida

Lauren E. Adkins, AHIP, Assistant University Librarian, UF Health Science Center Libraries, Gainesville, Florida

Susan Harnett, Medical Information Services Librarian, Borland Library, Jacksonville, Florida

Melissa L. Rethlefsen, AHIP, Executive Director, Health Sciences Library & Informatics Center / University of New Mexico, Florida

Background: Little Free Libraries (LFLs) are neighborhood book-exchange boxes. A public library partnered with a university health science library on a grant-funded project to add health

education components to 30 new public library-sponsored LFLs built on rural county properties. The objectives were to improve rural residents’ health literacy skills, increase access to quality health information for diverse populations, and raise awareness of NIH’s All of Us Research Program.

Description: The libraries collaborated to select and purchase 424 consumer health books in English and Spanish. Online consumer health materials were selected and printed. Laptops and Wi-Fi hotspots were also purchased, as the public library will collaborate with their county Health Department to conduct mobile health reference hours at the LFLs when possible. Health librarians conducted a one-day training for county health educators and public library employees on: health reference techniques; evaluating and using online health resources; health literacy; strategies for successful health tabling; and conveying the benefits of All of Us to patrons. The eHEALS eHealth Literacy Scale was administered before and after the training.

Conclusion: Nine LFLs have been installed in lower-income neighborhoods so far; all 30 should be completed by July 2021. To date, the most popular LFL location is at a sports complex with over 600 items circulated since April 2020. The most frequently restocked health resources are related to teaching children healthy habits. The eHEALS results demonstrated that the training day increased participants’ perceived ability to navigate health information online.

Although still ongoing, this project has already improved rural residents’ awareness of and access to authoritative consumer health resources. Overall, the LFLs and the planned health reference sessions will contribute towards reducing health information disparities amongst underserved populations, as county residents are empowered to make effective use of information for health decision-making.

The Virtual Library Treasure Hunt: Marketing Library Resources During COVID

Practice Area: Education

Sola Whitehead, Medical LIbrarian, VA Portland Health Care System, Portland, Oregon

Background: Due to the COVID-19 pandemic, the hospital library serving staff, patients, and family members was closed to in-person visitors from March 2020 to April 2021. However, the need remained to let staff know about electronic library services. In an effort to celebrate the library services, and create a fun, interactive way for hospital staff to become more familiar with the broad range of services and products that remain available through the library, a virtual library treasure hunt was created that could be completed at the leisure of the individual participating.

Description: The virtual treasure hunt was developed by first identifying a variety of electronic resources to highlight for hospital staff. This included how to find the complete list of

databases, searching for specific items in databases for hands-on learning, setting up remote access, downloading an app for reading full text subscriptions, requesting an interlibrary loan, and finding a specific e-book. The treasure hunt was broken into 10 sets of instructions, beginning with a slide deck that introduced the treasure hunt, directed a participant to the first resource, and gave directions to email the librarian with a specific subject line. Outlook email templates were created to auto-respond based on email subject lines, to provide the next “clue.” Participants were promised a chocolate bar for getting through all 10 resources.

Conclusion: The Library Treasure Hunt was introduced in October 2020, in conjunction with National Medical Librarians Month. To date, 10 individuals have completed all 10 steps of the treasure hunt for a chocolate bar. Others have started the hunt, but as it is participant-driven, individuals decide how far they want to go. The treasure hunt remains available to hospital staff. Feedback has been positive, indicating that participants learned about new-to-them resources and were excited to use them. While it may have been COVID that generated a need for a different kind of way to market library resources, it has become a fun way for staff to explore library resources they wouldn’t otherwise know about.
