# Supplementary material for: 121st Annual Meeting Medical Library Association, Inc. May 10–27, 2021
Source: J Med Libr Assoc. 2022 Jan 1;110(1):E1–E29. doi: 10.5195/jmla.2022.1438 (PMC8830401; doi:10.5195/jmla.2022.1438)
Supplement: Supplementary file 2 — Appendix 2: MLA '21 Poster Session Abstracts [file jmla-110-1-E1-s02.pdf]

Medical Library Association MLA ’21 Poster Abstracts

MLA ’21
MAY 21–26
#MLANET21
Transforming Our Diversifying Communities

vConference & Exhibits

Abstracts for the poster sessions are reviewed by members of the Medical Library Association National Program Committee (NPC), and designated NPC members make the final selection of posters to be presented at the annual conference.

POSTERS

Actively Archiving: Creating a web archive for future research into vaccine hesitancy, misinformation, and public health

Practice Area: Information Management

Samantha Abrams MLIS - Web Resources Collection Librarian, Ivy Plus Libraries Confederation, Brooklyn, New York

Barbara B. Cavanaugh MSLS - Asst. University Librarian for Health Sciences & STEM Libraries, University of Pennsylvania Libraries

Richard James - Nursing Liaison Librarian, University of Pennsylvania Biomedical Library, Philadelphia, Pennsylvania

Samantha Kaplan MLIS, PhD - Research & Education Librarian, Duke University Medical Center Library & Archive, Durham, North Carolina

Hannah Rutledge PhD, MLIS, AHIP - Director, Biomedical Library, University of Pennsylvania Libraries, Philadelphia, Pennsylvania,
Megan von Isenburg AHIP - Associate Dean, Duke University Medical Center Library and Archives, Durham, North Carolina

Lucy B. Waldrop MLIS, MA - Assistant Director, Technical Services Librarian, Duke University Medical Center Archives, Durham, North Carolina

Background: Content on the web is impermanent and unstable. In an era of increasing misinformation, "fake news," and the politicization of science, it is essential to capture transient information on websites. Web archiving offers a way to capture specified websites so that they can be used in future research to understand misinformation and public discourse on topics. This poster will highlight how this specific web archive can be used by researchers and how web archives in general can be created to address other important topics.

Description: The Vaccination in Modern America Web Archive was collaboratively developed by librarians from two health sciences libraries whose institutions prioritize vaccine research in the Ivy Plus Libraries Confederation. The poster will discuss how the web archive identifies, captures, and assigns metadata to webpages representing the current state of public discourse and contrasting approaches to authority on the topic in the United States, focusing on sites that are both pro- and anti-vaccination. The purpose of this collection is to capture potentially ephemeral information about vaccination that could be used by health services researchers, information scientists, sociologists, public health professionals, and others to understand the motivations, practices, and outcomes of health information and misinformation. Archivists provided essential expertise in web archiving. The project was approached from a public health and misinformation perspective and opposes the rhetoric of the anti-vaccination websites.

Conclusion: The web archive launched in February 2020. Both pro- and anti-vaccination sites were identified. “Anti” sites include those that indicate opposition or foster uncertainty about established guidelines for vaccination, as well as those that focus on chemical vaccine adjuvants or organic natural living as a replacement for vaccination. “Pro” sites include federal agency websites on vaccination and blogs on evidence-based parenting. Prominent natural living associations and not-for profits that have made anti-vaccination arguments or who have expressed sympathies to vaccine avoidance have been included to provide context around anti-vaccination information campaigns. It was not technically feasible to capture social media posts. The archive is being expanded to include pro- and anti- COVID-19 vaccination websites.

Adapting and planning novel services during and after the COVID19 pandemic

Practice Area: Education

Kelly Thormodson - Associate Dean and Director, Penn State University Harrell Health Sciences Library, Hershey, Pennsylvania

Alex Harrington - Access Services & Instruction Librarian, Harrell Health Sciences Library, Penn State, Hershey, Pennsylvania

Esther Y. Dell AMLS, AHIP - Scholarship Access and Support Librarian, Harrell Health Sciences Library, Hershey, Pennsylvania

Xiaoyu Sun MS - IT Generalist, Harrell Health Sciences Library, Research and Learning Commons, Hershey, Pennsylvania

R**obyn B. Reed MA, MLIS, AHIP** - Biomedical Informatics and Emerging Technologies Librarian, Harrell Health Sciences Library, Hershey, PA

Eliza Donne - Multimedia Specialist, Harrell Health Sciences Library, Hershey, Pennsylvania

Bradley A. Long MSLS - Embedded Health Sciences Librarian, Harrell Health Sciences Library, State College, Pennsylvania

Amy C. Knehans AHIP - Clinical Outreach, Liaison & Instruction Librarian, Library, Hershey, Pennsylvania

Benjamin Hoover - Associate Director and Coordinator of Innovative Technology Services, Harrell Health Sciences Library, Hershey, Pennsylvania

Background: In response to the COVID-19 pandemic, library faculty and staff evaluated and acted to meet the immediate and anticipated library services and instruction needs of College of Medicine campuses and the affiliated hospital. This included leveraging expertise in the library, partnering with groups within the institution and experimenting with new models of service. These efforts demonstrated the value of library personnel, spaces and services to the

communities served and has placed the library on sure-footing to provide library services and instruction post-pandemic.

Description: In spring 2020, faculty and staff of the library began to work through the new paradigm of the COVID-19 pandemic and its impact. First, library personnel evaluated how to provide library services remotely and what were the immediate and longer-term needs of the college main and regional campus and hospital. During the pivot to all online instruction, the library partnered with other departments to provide “just in time” training and support for virtual meeting tools. Librarians provided COVID-19 research support and created a COVID-19 Research guide in collaboration with library Faculty from across the university. Faculty and staff collaborated to create digital learning modules to provide customized library orientations to different categories of students and learners in the College of Medicine. These modules were created with the eLearning software Articulate Storyline and distributed in the learning management system Canvas. Finally, after experimenting with virtual tools and strategies, the library has been able to provide continuation of instruction, reference and other services virtually. Moving forward, the library will continue to leverage expertise and technology to meet the changing needs of our patrons.

Conclusion: The library adapted to meet the needs of the institution during the pandemic and will continue to leverage personnel, expertise, and technology to meet the changing needs in the future. This includes rethinking library instruction, space usage, and programming in an environment where not all students, staff, and faculty have returned to campus and instruction and curriculum could be radically changed.

Analysis of Print Journal Usage and Adaptation of Interlibrary Loan/Document Delivery Services in Response to COVID-19

Practice Area: Information Management

Phill Jo, AHIP - Head of Access Services/Associate Professor, University of Oklahoma Health Sciences Center, Oklahoma City, Oklahoma

Joy Summers-Ables AHIP - Director / Professor and Chair, Robert M. Bird Health Sciences Library / Health Sciences Library & Information Management, Oklahoma City, Oklahoma

Shari Clifton MLIS, AHIP - Professor / Associate Director & Head, Reference, Robert M Bird Health Sciences Library, Oklahoma City, Oklahoma

Tara R. Malone MLIS - Assistant Professor/Head of History of Medicine and Serials, Robert M. Bird Library, Oklahoma City, Oklahoma

Background: With the proliferation of COVID-19 cases in the spring of 2020, many libraries closed and provided limited or no access to their physical space. This impacted interlibrary

loan/document delivery (ILL/DD) services at the [redacted] library. The library, while closed, continued to offer these services and saw a marked increase in lending requests from other libraries, particularly for older items maintained in the print journal collection. This poster addresses the impact of COVID-19 closure on print collection usage, the service adaptations made to address it, and the potential benefits of preserving a large print collection.

Description: Access to the library’s print collection was limited from March to June 2020 during the building’s closure. During the second quarter of 2020, lending requests from other libraries increased more than 50% through the DOCLINE and OCLC systems. Requests requiring the utilization of the print collection increased more than two times over the same time period in 2019. Meanwhile, patrons’ document delivery requests filled from the print collection more than doubled by the fourth quarter of 2020 as a result of modified document delivery services, including the elimination of associated fees. To meet increased demand while ILL/DD staff were on administrative leave during the closure, on-site staff from multiple departments helped pull and scan physical journals. The collective effort helped ensure seamless access for other libraries and affiliated patrons who could not access the library’s physical collections.

Conclusion: During these challenging times, the [redacted] library was able to adapt services and continually meet the needs of patrons and borrowing libraries. This was possible in large part due to the retention of a sizeable print journal collection. Analysis of print journal usage during this time demonstrates the value of retaining such a collection in an age when many institutions have shifted to fully electronic, potentially decreasing years of coverage or incurring a total loss of access due to an unexpected interruption in service.

An Analysis of Publisher DEI Policies

Practice Area: Professionalism & Leadership

Lindsay Barnett - Collection Development and Scholarly Communication Librarian, Cushing/Whitney Medical Library, Yale School of Medicine, New Haven, Connecticut

Andrea E. McLellan AHIP - Head of Collections and Technical Services, Health Sciences Library, McMaster University, Hamilton, Ontario, Canada

Objectives: How are publishers, electronic resource providers, and grant funding agencies incorporating the values of diversity, equity, and inclusion (DEI) into their policies and business models? How might prioritization of these values improve the author experience and promote diversity in scholarship?

Methods: The authors evaluated the websites of major providers of medical education and clinical electronic resources and their popular imprints, in addition to major funding bodies of

medical research, for information representing approaches to DEI. This included reviewing both general policy sections, author guidelines, and site searching for DEI keywords.

Results: There is not yet an industry standard for inclusion of DEI principles into the policies of publishers, electronic resource providers, and grant funding agencies. The authors discovered inconsistent adoption of these values and the importance placed on them among the organizations evaluated. Results ranged from no mention of DEI to explicit support for social movements around DEI and established equity goals.

Conclusions: There is significant opportunity for publishers, electronic resources providers, and grant funding bodies to incorporate DEI principles in their business models. The authors intend this project to highlight this area and identify ways publishers can improve. As the project progresses, the authors hope to engage more library vendors in the conversation as well as information professionals who can add their unique expertise and experience to give the project more depth and help it grow.

Availability of LGBTQ-related Information at the MUHC’s Glen Site Patient Resource Centres

Practice Area: Information Services

Melissa Moleirinho - MISt Student, McGill University, Montreal, Quebec, Canada

Objectives: Members of the LGBTQ community, often marginalized by healthcare systems, have distinct information needs. This study aims to determine whether the adult and pediatric book collections of the Patient Resource Centres at the Glen Site of the MUHC in Montreal, QC contain LGBTQ-specific literature, and to what extent.

Methods: Using the publicly available online catalogues for two patient resource centres (PRC), searches for LGBTQ-related literature were completed using relevant MeSH terms and current use/common terminology to reflect probable consumer usage. The results in each catalogue were compared to each other - for each term and overall.

Results: Both catalogues demonstrated low numbers of LGBTQ-specific literature. Gender identity (gender diversity, transgender, etc.) literature was more prominent than sexuality-related information. Overall, the pediatric PRC housed more relevant literature. The low numbers of LGBTQ-specific literature suggest a lack of diversity and inclusion in both collections. The distribution of literature in the pediatric PRC points to the increased relevance of gender identity for childhood development. In general, the concern with gender identity and non-conformity may suggest the perception that these topics are most associated with medical outcomes.

Conclusions: This study identified that these Patient Resource Centre collections had low numbers of LGBTQ-specific print information. Future studies may endeavour to identify the particular needs of this patient/hospital population in order to more effectively gauge the ability

of the collections to meet their needs in a consumer health information context. In addition, in keeping with the trends towards digital library transformation and increased concern for diverse and inclusive collections, PRCs may employ this and related future studies to bolster and guide the overhaul of their collections.

Characteristics of research publishing, and registered clinical trials on the COVID-19 pandemic focus on Japan

Practice Area: Global Health & Health Equity

Mari Makishi - Information Services, NARASHINO Media Center for Research & Education, Media Net Center, Toho University, Funabashi City, Chiba, Japan

Objectives: The academic output of COVID-19 research has been proliferating. I aim to describe the characteristics of the current status of academic information distribution from journal articles and preprints and registries.

Methods: The data sources were PubMed, medRxiv and bioRxiv, the iSearch, ICTRP, PROSPERO from 09/01/2019 to 12/31/2020.No limitations were placed on study design and language. I screened and categorized studies according to i) publication type, ii) Research area, and iii) topics covered.

Results: Over 50,000 articles related COVID-19 were published until 11 Jan.2021 in PubMed. up 20% between Jun to Dec when compared with the same period in latest 3 years(2017-2019) average. The highest rate of increase was in September, at 31.4 %. Around 6% of those indexed in PubMed with MeSH or SCPs were dedicated to covid-19 topics. Ichushi-web showed an increase in reports about COVID-19 in September and October 2019. More than 12,000 of the COVID-19 articles published in 2020 were preprints(medRxiv and bioRxiv). Abou 54% of COVID-19-related protocols registerd in ICTRP ware via Clinicalrials.gov, which together with Indis's CTRI(10.7%) and China7s ChiCTR(10.5%), the second and third most popular protocols, accounted for 3/4 of the total.

Conclusions: PubMed differed in the rate of MeSH and SCPs assigned for specific journals. It may be necessary to take this into account when searching for literature related to COVID-19 in order to develop a search strategy. The speed of indexing in PubMed is increasing. Ichushi-Web was a time lag of about half a month and an overwhelming difference in the number of articles compared to PubMed. Cases reports in Japan began when the Infectious Diseases Society of Japan began publishing information related to the acceptance of passengers and cruisers aboard the Diamond Princess on its website. These were later published in a special issue of the Journal of The Japanese Association for Infectious Diseases and reported as papers. However, the fact that such information disclosure and sharing was attempted in Japan from an early stage, including cases that did not published, may have provided valuable

information to medical personnel who treated patients in the field. Similarly, the Japanese Respiratory Society and the Japanese Pediatric Society are also attempting to share cases. When searching for information from Japan, it is necessary to check not only literature databases but also these academic society sites and guideline sites.

Characterization of the highly cited articles published by a genetics research department: an exploratory study

Practice Area: Innovation & Research Practice

Mirian Ramirez - Research Metrics Librarian, Ruth Lilly Medical Library, Indianapolis, Indiana

Elizabeth C. C. Whipple - Assistant Director for Research and Translational Sciences, Ruth Lilly Medical Library, Indianapolis, Indiana

Objectives: To identify and assess highly cited papers included in the Web of Science Essential Science Indicators, this study looked at authors affiliated with a specific department at a School of Medicine from 2010-2019. For this study, we examined authorship characteristics, including female authorship trends, subject trends, and intramural and extramural co-authorship networks. This approach aims to highlight research impact trends to inform the department's leaders in decision-making for future publication and research strategy development directions.

Methods: We conducted a bibliometric analysis of publications from faculty in a specific department at a School of Medicine over the last ten years (2010-2019). The searches were conducted in June 2020. We used a three-phase approach to find those departmental articles ranked as "highly cited papers" in the Web of Science (WoS) "Essential Science Indicators" database: Phase 1. We queried Scopus to gather publications listing the author's departmental affiliation; Phase 2. Queried the WoS Core Collection for all the citations resulted in the Scopus search and limited the search to return only the publications identified as "Highly Cited" papers; Phase 3. Used PubMed to compile funding information due to its more standardized format of reported funding support. We utilized the OpenRefine tool to perform cleanup and cluster the author name lists and Excel to work with datasets of bibliometric data.

Results:

Conclusions: For the 2010-2019 time frame, a total of 1,077 articles (original articles and review articles) were published by this department, with 37 documents identified as Highly Cited, categorized by WoS Essential Indicators. Identified documents were categorized under ten research fields and were published in 17 journals, from diverse WoS subject categories, including Neuroscience, Oncology, and Genetics research fields. The results show that Highly cited articles were published in 17 high-impact journals ranked in Q1 and Q2. Indicative of that, the highly cited papers have a strong relationship between the impact factors. 38% of the

documents correspond to case-control studies. Topics covering "Genome-wide Association Study", "Genetic predisposition to disease," and Polymorphism, single nucleotide" are among the most used MeSH terms ."

The Citation Cloud of a Biomedical Article: Enabling Citation Analysis

Practice Area: Innovation & Research Practice

Neil R. Smalheiser MD, PhD - Professor in Psychiatry, Psychiatry, Oak Park, Illinois

Jodi A. Schneider - Assistant Professor, School of Information Sciences, University of Illinois at Urbana-Champaign, Champaign, Illinois

Background: Citation analysis is crucial for tracing the diffusion of knowledge. It has wide applicability to medical libraries, for instance to study research impact, to analyze the continued citation of retracted publications, and to find related articles via reference searching and citation tracking. Current tools are limited because they require expensive subscriptions (e.g., Web of Science, Scopus), lack a user-friendly public interface (e.g., Open Citations), or do not support backwards reference searching (e.g., Google Scholar). The objective of the Citation Cloud is to provide a fully open, user-friendly public interface for anyone to access or analyze citation data in biomedicine.

Description: We have built an extension to PubMed that allows any user to visualize and analyze important citation relationships around given article, using open citations provided by iCite, Microsoft Academic Graph, Semantic Scholar, Open Citations, PubMed Central, and ArnetMiner. The citation cloud allows tracking both backwards (“Articles Citing this Article”) and forwards via citation tracking. It also displays two important “sideways” citation-relationships: bibliographically coupled articles and co-cited articles. Two articles are bibliographically coupled articles when their reference lists overlap, and this remains constant over time. By contrast, two articles are co-cited if they are both cited by the same article(s). Co-citation is a measure of similarity that changes over time as newer articles may potentially co-cite new pairs of articles.

Conclusion: The Citation Cloud can be accessed by running any query on the Anne O’Tate value-added PubMed search interface http://arrowsmith.psych.uic.edu/cgi-bin/arrowsmith_uic/AnneOTate.cgi and clicking on the Citations button next to any retrieved article. One can view and export citations in each of the four different categories – citing articles, bibliography articles, co-cited articles, and bibliographically coupled articles – individually. Citation data is more easily available via the availability of open, user-friendly public interfaces. The Citation Cloud tool should greatly enable the study of biomedical citations by the scientific community. A preprint describing the Citation Cloud in detail has been posted in medRxiv.

Community Building with LGBTQIA+ Health

Practice Area: Information Services

Nora Franco - Consumer Health Librarian, Network of the National Library of Medicine Pacific Southwest Region / UCLA Biomedical Librar, Los Angeles, California

Background: The emphasis on relationship and trust building within the LGBTQIA+ community is inherent to creating an open environment where healthcare professionals, information workers, and consumers can freely ask questions and learn about health issues relevant to this population.

Description: The librarian created and taught an open access online course dedicated to LGBTQIA+ health information and utilizing specific feedback from participants, will elaborate and implement future course design focused on community building.

Conclusion: The future course design will be updated to include enhanced evaluations and outcomes. Pre-tests and surveys will be added in addition to the existing post-course evaluations. The emphasis on the course design will focus on adult learning style and theory, and methods to creating a collaborative environment where both student and instructor can engage as openly as they wish, using anonymous tools such as Padlet, Google Forms, and other online anonymous software.

Confronting Inequity: Social Justice Dialogue in a Health Science Library

Practice Area: Professionalism & Leadership

Gail Kouame MLIS - Assistant Director for Research & Education Services, Augusta University Libraries, Robert B. Greenblatt, M.D. Library, Augusta, Georgia

Darra R. Ballance MLIS - Assistant Professor, Augusta University Libraries, Robert B. Greenblatt, M.D. Library, Augusta, Georgia

Jacob Gallay - Clinical Information Librarian, Augusta University Libraries, Robert B. Greenblatt, M.D. Library, Augusta, Georgia

Natalee D. Reese - Nursing Information Librarian, Augusta University Libraries, Robert B. Greenblatt, M.D. Library, Augusta, Georgia

Peter C. Shipman - Dental Medicine and Cancer Librarian, Augusta University Libraries, Robert B. Greenblatt, M.D. Library, Augusta, Georgia

Lachelle Smith M.Ed., MSLIS - Instructor Librarian, Augusta University Libraries, Robert B. Greenblatt, M.D. Library, Augusta, Georgia

Shafer G. Tharrington Bachelor of Arts - Medical Library Research Associate, Augusta University Libraries, Robert B. Greenblatt, M.D. Library, Augusta, Georgia

Background: The purpose of this program was to demonstrate how a departmental social justice discussion group was successfully created, and how each department member was able to discuss health-related social justice narratives and perspectives with personal meaning in the discussion group.

Description: In the aftermath George Floyd’s death, the Assistant Director of Research and Education Services at a health sciences library proposed devoting a portion of staff meetings to discuss issues in social justice and anti-racism. Each department member would generate a topic and organize readings or links to media in an internal LibGuide. Initially, there was a total of seven discussions, each lasting an average of twenty minutes. Each staff member described their motivation in selecting their topic and accompanying resources and led the subsequent discussion. Discussion topics included white fragility, racial disparities surrounding leg amputations of Black diabetes patients in Mississippi, transracial adoption, local food deserts, white privilege in medical school education, black transgender violence and discrimination, and pipeline institutional racism. The readings and discussions revealed marginalized group perceptions and reality are not necessarily willingly acknowledged/addressed by the privileged group.

Conclusion: Participants felt the topics were timely, thought-provoking and useful in understanding current imbalances in social equity in health-related areas. Each department member could identify and share a social justice area of concern. Many of the topics are addressed in critical librarianship scholarship, and lessons from the discussions could be applied to increased understanding of, and service to, marginalized users of their library’s community. Team members agreed to continue the discussions at staff meetings once per month on broader diversity and social justice topics. The topic of food deserts was identified for follow-up action because of the need in the residential area adjacent to the health sciences campus.

COVID-19 Educational Support Team: Librarians, physicians, and medical students collaborate to synthesize COVID-19 research for clinicians

Practice Area: Clinical Support

Kaitlyn Van Kampen - Kathleen A. Zar Clinical Library Resident, University of Chicago, John Crerar Library, Chicago, Illinois

Debra A. Werner - Director of Library Research in Medical Education, University of Chicago, John Crerar Library, Chicago, Illinois

Maggie Collison MD - Maggie Collison, Clinical Instructor, Infectious Disease, University of Chicago, Chicago, Illinois

Vineet Arora - Assistant Dean, Pritzker School of Medicine, University of Chicago, Chicago, Illinois

Background: The onset of the COVID-19 pandemic created challenges for clinical teams in staying up-to-date with the vast amount of information being published about the virus. In order to best care for their COVID-19 positive and suspected patients, clinicians at the University of Chicago Medical Center needed information to be collected, synthesized, and disseminated for easy access. The clinical librarians were contacted to assist in finding and organizing articles, along with four medical students and a resident. This team’s objective was to support frontline clinicians and help guide decision-making in clinical care by answering clinical questions through critical reviews of COVID-19 literature.

Description: The clinical librarians were contacted in March 2020 to assist with finding COVID-19 literature. Due to the volume of material, four medical students and a resident were recruited to form the COVID-19 Educational Support Team. The team both summarized important articles and answered specific COVID-19 clinical questions emailed by clinicians. The librarians instructed the students on using databases and pre-print servers and critically appraising the information. The queries and article summaries were posted to a public webguide created for clinicians. The volume of questions led to the transformation of this service into a medical school elective in May with ten new students. The program was evaluated through a REDCap survey of clinicians who used the service to determine their view of usefulness. Students were also surveyed to determine elective improvements for the future.

Conclusion: From mid-March through May, a total of 90 questions were answered and 325 articles were summarized. This service was viewed positively by clinicians, with 100% of respondents expressing satisfaction and 78% reporting a change in thinking due to the syntheses. The success of the initial cohorts led to the development of an approved medical school clerkship for a similar synthesis service for non-COVID requests. With feedback from the clinician survey and the students who participated in the initial group, the clerkship was expanded to include three hospitalist mentors with clinical expertise who will work with the librarians to give didactic sessions along with the synthesis service.

Creating a Directory of Information Literacy Mapping in Health Sciences Disciplines

Practice Area: Education

Nena Schvaneveldt AHIP - Assistant Librarian, Eccles Health Sciences Library, Email: nena.schvaneveldt@utah.edu, Salt Lake City, Utah

Brandi Tuttle AHIP - Research & Education Librarian, Duke University Medical Center Library & Archives, Email: brandi.tuttle@duke.edu, Durham, North Carolina

Christine Andresen MLS, MSIT - Research & Education Informationist, MUSC Libraries, Charleston, SC

Angela Murrell - Instruction & Research Librarian, Scripps Research Libraries, Email: amurrell@scripps.edu, La Jolla, California

Kristine A. Petre AHIP - Health Sciences and Information Literacy Librarian, Reeves Library, Bethlehem, Pennsylvania

Sarah Wade AHIP - Assistant Medical Librarian, Campbell University School of Osteopathic Medicine, Lillington, North Carolina

Background: Information literacy (IL) instruction permeates health sciences education. There have been numerous attempts to map IL concepts to each professional academic discipline to improve IL instruction and advocate for librarian involvement in the curriculum. The Medical Library Association (MLA) recently formed the Education Domain Hub. One of the Hub's first projects focused on creating resources for mapping IL concepts to program-specific educational standards and objectives, thus forming the IL Mapping Workgroup. The group decided to conduct an inventory of IL mapping efforts already in place, to encourage collaboration and reduce redundant efforts through a directory on the MLA website.

Description: The group’s first task was to inventory existing IL mapping efforts, whether they may be small scale, such as at a single institution, or larger scale, such as of an entire discipline. To create a list of IL mapping efforts, the group designed a survey to be completed by the contact person on each mapping project. The survey was designed by collaborative input from workgroup members and was approved by consensus before being tested in the Education Domain Hub. Following additional refinements, the survey progressed to the development of the prototype. The prototype will be tested by volunteers outside of the Education Domain Hub. The subgroup also devised a workflow to be implemented when creating and updating the directory of IL mapping efforts, which will be housed on the MLA website.

Conclusion: Once finalized, the prototype will be tested by volunteers who have undertaken mapping efforts to ensure the survey is both clear and functional. Following revisions, the survey will be widely distributed. It will remain available on the MLA website so future IL mapping efforts can be included in the directory. Ultimately the directory will serve as a resource for all information professionals involved in IL curriculum development and integration, whether by identifying potential collaborators, or by adapting existing IL mapping efforts to additional disciplines. The survey and directory will be accessible to MLA members

as well as non-MLA members with a free MLANET login. The Education Domain Hub will ensure the directory is marketed and kept up-to-date.

Creating a Handbook on New Diabetic Medications to Educate Clinicians

Practice Area: Clinical Support

Lindsay E. Blake AHIP - Clinical Services Coordinator, UAMS Library, Little Rock, Arkansas

Julie M. Gerlinger - Clinical Consultant, Cerner, Arkansas

Background: The objective of this project was to create and distribute a handbook of currently used diabetic medications to assist in education of primary care clinicians. Diabetes is a common co-morbidity that clinicians see in both hospitalized and clinic patients. A plethora of new diabetic medications have emerged on the market during the last few years, but many primary care clinicians are still trying to navigate the stream of information to decide which of these drugs may be beneficial to their patients.

Description: A Clinical Librarian and Registered Nurse (RN) Case Manager sought and received funding from the Network of the National Library of Medicine (NNLM) to create, market, and distribute a booklet detailing currently used diabetic medications, including many newer medications, which may not be as well known. The booklet contains pharmacologic information such as medication onset, peak, and duration as well as special precautions or possible adverse effects. Generic and Trade names as well as Medicaid coverage are included. The booklet was produced in three sizes, two made to fit in clinician white coat pockets and one full page. The booklets where first distributed locally and then at regional medical centers and community hospitals around the state. Clinicians were emailed a survey one month after they received the booklets to review use and educational benefits as well as gain feedback.

Conclusion: The Diabetes Medication Handbook has been well-received by residencies throughout Arkansas. Over 100 booklets were distributed in late 2020. Changes in Medicaid required an update for 2021 with over 200 booklets requested before printing and with around 500 shipped. Future projects will include the creation of a diabetic medication 'Menu' which will allow clinicians to work with patients more easily on choosing the best medications for their lifestyle and budget.

Creating a Learning Community: Developing Critical Thinking Skills in First Year Psychology Majors

Practice Area: Education

Alessia Zanin-Yost AHIP - Health Sciences Librarian, Slippery Rock University, Slippery Rock, Pennsylvania

Background: The poster illustrates the creation of a seven-credit cluster that included research skills, writing, and psychology to develop research and content competencies in first-year students majoring in psychology at a four-year institution. The faculty designed assignments that would build on each other and blend skills, knowledge, and critical thinking to allow students to understand the importance of connecting learning through the curriculum and reinforce content knowledge through multiple exposures and applications. The poster illustrates the planning, implementation, results, and lessons learned/challenges.

Description: Planning took a year by defining common skills students should have gained by the end of their first semester. Coordination of assignments was critical in ensuring success. The aim was to teach students how to research, write, and create an empirical research report and poster. A scaffolded approach was used to allow learning and applying concepts in different circumstances. For example, in the research skills course, the students learned to write an annotated bibliography, expanded in an annotated bibliography in the writing class, and elaborated in the psychology course. At each step, students received multiple feedback from the faculty. The project's evaluation included various formats: work assessment, discussions among the faculty, reflection papers, and overall improvement of the students during the semester.

Conclusion: Some of the challenges encountered by the faculty were unexpected. For example, due to COVID, the courses had to migrate to a virtual environment. Some issues were unforeseen; for instance, it took students a while to understand that the three classes were part of the cluster, resulting in many emails. Despite this, the faculty saw an increased level of knowledge during the semester. Comments from students reflected they supported this cluster approach and how beneficial it was to them. The faculty is reworking the project to correct the issues encountered, and the cluster will be re-offered in fall 2021.

Creating Library Collections to Support the Teaching and Learning of LGBTQIA+ Cultural Competencies for Students in the Health Sciences

Practice Area: Information Management

Janina Mueller - Metadata and Digital Initiatives Librarian/ Assistant Professor, Massachusetts College of Pharmacy and Health Sciences, Boston, Massachusetts

Background: It's estimated that 4.5% of Americans identify as LGBTQ+, yet many healthcare workers are insufficiently trained to provide comprehensive healthcare to these patients. The Massachusetts College of Pharmacy and Health Sciences University (MCPHS University) offers over 100 health sciences programs to students. Last spring, the MCPHS Library built a collection of LGBTQIA+ resources for its health sciences students and faculty. The goal of the collection is to raise awareness of the healthcare needs of LGBTQIA+ patients and to complement the health science curricula at the school.

Description: This idea for this collection came after attending the symposium “Communities in Crisis: Libraries Responding to the Opioid Epidemic” in Devens, MA, in November 2019. A number of libraries presented innovative programs they set up in their communities to provide assistance, support and educational resources around the opioid epidemic in New England. After this conference, the MCPHS Library secured funding from the IMLS to purchase video materials that raise awareness of mental health issues affecting LGBTQIA+ populations.

Conclusion: We initially envisioned promoting this collection through a number of community screenings of documentaries purchased with the Social Infrastructure grant. The idea was to engage students and faculty through film screenings and discussions around issues affecting LGBTQIA+ patients. Due to the Covid-19 pandemic, these screenings and discussions are postponed. The collection has primarily been promoted to MCPHS faculty and departments; instead of community events, the collection is currently primarily used in classes, including nursing.

Creation and Use of Social Ecology Based Interventions via a Collaboration of Library, Visual Communication and Occupational Therapy Departments to Improve Health Literacy

Practice Area: Global Health & Health Equity

Daniel Verbit - Scholarly Communications and Digital Initiatives Librarian, Gutman Library/ Academic Commons / Jefferson (Philadelphia University and Thomas Jefferson University), Philadelphia, Pennsylvania

Beth Shirrell - Program Director & Assistant Professor, Visual Communication Design, Thomas Jefferson University, Philadelphia, Pennsylvania

Monique Chabot - assistant professor of occupational therapy, Thomas Jefferson University, King of Prussia, Pennsylvania

Low health literacy has a direct impact on the health outcomes of non and low-English speaking populations. The social ecology theory supports the influencing of various social systems to increase engagement with health literacy materials. The poster shows a librarian sparked synergy of graphic design and occupational therapy systems as potential solutions to address specific health literacy and access issues. To influence the social environment around people to improve health literacy, occupational therapy students worked with visual communication students in their systems design integration course with the support of the Occupational Therapy liaison librarian. The social ecology theory, as established by Urie Brofenbrenner, looks at the idea that external factors influence people in our physical and social environments. These factors encircle a person and go from individual factors in concentric circles into the community and the overarching policies that cover those communities. It acknowledges the complexity surrounding each and every one of us through various levels of influence. The professors and community members felt this interdisciplinary collaboration of the systems was successful. The student collaboration yielded traditional health literacy materials such as flyers and brochures and innovative materials such as phone applications, kit boxes for social support programs, graphic novels, advertising campaigns, and interactive community murals. These were created to address the needs of a variety of populations ranging from children with asthma, children living with trauma, teenagers with mental health concerns, night shift worker health disparities, accessibility on public transit, and caregivers of people with dementia within the Hispanic population.

Creation of an Institutional Quality Assessment Tool Repository

Practice Area: Information Services

Leila S. Ledbetter AHIP - Research and Education Librarian, Duke University Medical Center Library & Archives, Durham, North Carolina

Steph Hendren - Research and Education Librarian, Duke University Medical Center Library & Archives, Durham, North Carolina

Background: Our library's comprehensive literature search service primarily focuses on expert searching and guidance on general evidence synthesis methodology. We have experienced an increase in the number of questions about quality assessment tools. Our practice is to suggest the most commonly used tools. However, teams sometimes reported these tools did not meet their needs. To solve this problem, we created a repository and finding aid of quality assessment and risk of bias tools. This allowed us to become more familiar with the array of

tools available, along with their purposes and features, and ultimately was helpful for our research teams.

Description: To construct our repository we conducted a literature search for quality assessment and risk of bias tools. We began compiling a list that provided the tool's name, description, citation, intended use, whether it was validated, and any notable details we discovered when researching the tool. We included tools whose purpose was to evaluate the methodological quality or risk of bias of study designs. In most cases, we omitted critical appraisal tools, which we defined as anything that was designed solely to be used by clinicians or students to evaluate the quality of an article for class work or patient care. Our final product is a comprehensive, searchable table that is freely available on our systematic review services guide.

Conclusion: There are a wide variety of quality assessment and risk of bias tools available to meet most study designs. Reporting guidelines such as STROBE, which are designed to aid in the reporting of research, and not specifically for quality assessment, were not added to the repository. It was frequently difficult to determine if a tool was validated, though many common tools have had their interrater reliability tested. Where possible, we prioritized gathering risk of bias tools as per Cochrane Handbook's recommendations. We included notes that added additional information about usability or purpose. The next planned step is to gather qualitative feedback from our researchers about their experience with the individual tools. Having a single searchable resource that captures the characteristics of a variety of tools can be a helpful for librarians or researchers conducting a systematic review outside their field of practice.

Critical Data Literacy: Addressing race as a variable in a preclinical medical education session

Practice Area: Education

Susan Koenig - Reference Specialist, Himmelfarb Health Sciences Library, Redmond, Washington

Stacy B. Brody - Reference and Instruction Librarian, Himmelfarb Health Sciences Library, Washington, District of Columbia

Background: Librarians provide instruction to medical students throughout their first and second years of preclinical medical education. This poster examines the development of teaching materials to support librarian-led instruction in critical data literacy, clinical algorithms, and the inclusion of race as a variable in risk calculators.

Description: Race is a social construct and a poor proxy for genetic differences. However, race is used in clinical calculators. Librarians provide an informatics session introducing the Framingham Study, later cohort studies, and the Atherosclerotic Cardiovascular Disease

(ASCVD) Risk Calculator to first-year medical students. This calculator includes race as an input and over or underestimates risk for certain groups. With the goal of fostering critical conversations about the use of race as a variable in research and practice, teaching materials were enhanced with descriptions of the cohort populations on which the calculator is based and the calculator development process. Librarian instructors were provided with pre-session materials describing problems with race-based medicine. A pre-session discussion and post-session debrief with librarian instructors were used to evaluate the usefulness of the updated teaching manual, slides, and supplementary readings.

Conclusion: Revisions were made to teaching materials for a librarian-led informatics session. Librarian instructors were provided with talking points and key questions to encourage medical students to be critical data consumers and critical users of clinical algorithms. The session was delivered to approximately 200 students across 14 WebEx sessions, lasting 50 minutes.

Data Analysis: using the 4 C’s framework to highlight researcher options

Practice Area: Information Services

Carrie L. Iwema AHIP - Coordinator of Basic Science Services, Health Sciences Library System, Pittsburgh, Pennsylvania

Background: Data analysis includes iterative steps that result in four sequential categories of collected data: raw, cleaned, processed, and analyzed. As there are numerous and varied resources used at each stage of data collection, it is a challenge to identify what approach to take to satisfy a researcher’s specific needs. Many research institutions provide support for these resources, and biomedical librarians are ideally situated to provide guidance to researchers on institutional resource identification, evaluation, and selection.

Description: While seeking out data analysis resource options at a large health sciences research university, I determined that the choices could be organized into four broad categories for analyzing bioinformatics data. I named this framework the “Four C’s” as it covers: (1) paying someone else to analyze the data (Core labs), (2) working with another researcher (Collaboration), and analyzing the data yourself either by (3) learning to program (Coding) or (4) using out-of-the-box software (Commercially licensed tools). I then developed a class and libguide on the Four C’s to highlight the pros and cons of each and to instruct researchers on where to find relevant university resources. To assist with these third and fourth stages of the data analysis process, also covered are institutional resources supporting computational needs and rigor and reproducibility considerations.

Conclusion: This poster presents the Four C’s framework for identification of institutional resources for data analysis. Although the examples shared are specific to bioinformatics data analysis at a fortunately resource-rich university, the framework can be used as a tool for

librarians at an institution of any type or size to provide support and help identify resources specific to their own researchers.

Data-Related Support in U.S.-Based Health Sciences Libraries: An Analysis of AAHSL Member Sites

Practice Area: Information Services

Kerry Sewell - Research Librarian for the Health Sciences, Laupus Library, Email: browderk@ecu.edu, Greenville, North Carolina

Jeffrey G. Coghill AHIP - Outreach Librarian, Laupus Library, Cell Phone: (252) 702-8644 Email: coghillj@ecu.edu, Greenville, North Carolina

Objectives: The purpose of this poster is to study the extent to which AAHSL member libraries have pivoted to provide data services, either broadly speaking or more specifically, through provision of data visualization ("data viz") services or data management services, for their respective constituencies.

Methods: The websites of all U.S.-based AAHSL member libraries were surveyed to determine the level and types of data-related support available. Site specific searches were carried out for the following: Data visualization Data services Data management We looked for informational support on data visualization, data services, or data management. The broader organization's website was examined to determine if data support was available outside of the library. The Carnegie Classification Code for each member library was collected in order to assess the relationship between Carnegie Classification and level of data support within a health sciences library. Frequencies were run for variables recording the presence or absence of data support elements. Chi-Square tests were run to determine the relationship between clustered Carnegie classifications and the presence or absence of specific elements of data support in Health Sciences Libraries.

Results: AAHSL member library websites had a low frequency of providing explicit information on data services, with only about one third (35%) of libraries reporting data consultation or data management services. Less than one fifth of libraries (19.8%) described having data visualization services. Informational support related to data was somewhat more prevalent than formalized data services, with almost 40% of libraries providing guidance on data management, almost a third of libraries (36.5%) providing information about data resources, and roughly one quarter of libraries offering informational support related to data visualization. There was a relationship between Carnegie classification and data-related support.

Conclusions: Published literature indicates a higher level of engagement in data-related services and informational support in health sciences libraries than the authors found in their survey of AAHSL member websites. Even on library websites describing data services,

services were often sparsely detailed. While informational support related to data was more commonly found, the informational support was often unclear in its scope, or served as a pathfinder to external resources. Data services appear to be offered outside of health sciences libraries with some frequency, but typically do not include data management support, indicating a gap that health sciences libraries are ideally situated to fill. The higher likelihood of providing services at research/doctoral institutions may result from new data management requirements among funders.

Designing an Open Repository of Educational Resources for Health Information Professionals

Practice Area: Education

Andrea C. Kepsel AHIP - Health Sciences Educational Technology Librarian, Michigan State University, East Lansing, Michigan

Christine Andresen MLS, MSIT - Research & Education Informationist, MUSC Libraries, Charleston, SC

Margaret Ansell AHIP - Associate Chair, Gainesville, Health Science Center Libraries, Gainesville, Florida

Virginia F. Desouky MLS, MA - Scholarly Engagement Librarian, Health Sciences Library, West Virginia University, Morgantown, West Virginia

Hilary M. Jasmin - Assistant Professor/Research and Learning Services Librarian, University of Tennessee Health Science Center, Health Sciences Library, Research and Learning Services, Memphis, Tennessee

Irene Machowa Lubker, PhD, MLS, MPH AHIP - Research and Education Informationist, Medical University of South Carolina Libraries, Charleston, South Carolina

Kristine A. Petre AHIP - Health Sciences and Information Literacy Librarian, Reeves Library, Bethlehem, Pennsylvania

Janette Pierce - Head of Research, Education and Outreach, Temple University Health Sciences Libraries, Philadelphia, Pennsylvania

Nena Schvaneveldt AHIP - Assistant Librarian, Eccles Health Sciences Library, Salt Lake City, Utah

Denise Shereff - Instructor II and Coordinator of Fieldwork and Assessment, University of South Florida School of Information, Tampa, Florida

Stephanie M. Swanberg AHIP - User Services Librarian, Moustakas Johnson Library, Michigan School of Psychology, Farmington Hills, Michigan

Brandi Tuttle AHIP - Research & Education Librarian, Duke University Medical Center Library & Archives, Durham, North Carolina

Hong-nei Wong DVM, MLIS, MPVM - Medical Education Librarian, Lane Medical Library, Stanford University, California

Andrea L. Wright - Clinical / Technical Services Librarian, University of Alabama Health Sciences Library, Tuscaloosa, Alabama

Tenley N. Sablatzky - Medical Librarian, Prairie View A&M University, Undergraduate Medical Academy, College Station, Texas

Background: Health information professionals routinely conduct a variety of instructional activities in support of health sciences education and research, clinical practice, and patient education. An open repository of resources can help prevent health information professionals from reinventing the wheel and instead build on the work of their colleagues. The Medical Library Association (MLA) recently formed the Education Domain Hub. One of the first projects the hub elected to pursue was to create a repository system and structure to collect educational resources for health information professionals. The Education Hub Open Repository workgroup was formed to accomplish this objective.

Description: The workgroup reviewed similar education repositories such as MedEdPortal and past MLA-based efforts like the Center for Education and Research. The group also consulted with MLA staff to determine the scope and logistics of hosting a repository on the MLA website. To guide the design of the repository, the group created a vision document that describes the repository’s purpose, audience, scope of content, and benefits. The group then developed a submission form that included questions regarding the resource’s purpose, audience, topic, accessibility, and copyright status. A prototype submission form was piloted on the MLA website, and is being refined based on feedback from the workgroup and volunteer testers. The workgroup also discussed and drafted guidelines for the process of maintaining the repository and the workflow of evaluating submissions.

Conclusion: Upon finalization of the submission form and evaluation process, a submission review group will be recruited. It is anticipated that submissions will be accepted by MLA members in Summer 2021. After a pilot period, which may result in additional changes to the submission and review process, the form will be opened permanently on the MLA website. The resulting open repository of educational resources will be available to anyone with a MLANET login, including health science educators who are not MLA members, and serve as a venue for health information professionals involved in instruction to disseminate the results of their instructional labor.

Developing and Conducting a DEI Language Inclusivity Assessment on a Health Science Library’s Website

Practice Area: Professionalism & Leadership

Jane Morgan-Daniel AHIP - Community Engagement and Health Literacy Liaison Librarian, Health Science Center Libraries, University of Florida, Gainesville, Florida

Matthew Daley - Web Designer, UF Libraries, Gainesville, Florida

Mary E. Edwards MLIS, EdD - Liaison Librarian, UF HSC Library, Gainesville, FL

Lori H. Eubanks - Office Assistant, UF Health Science Center Libraries/Associate Dean for Research and Fackler Director's Office, Bronson, Florida

Hannah F. Norton AHIP - Chair, Health Science Center Library - Gainesville, University of Florida, Gainesville, Florida

Nina Stoyan-Rosenzweig MA - Senior Associate in Libraries, Health Science Center Libraries, Gainesville, FL

Objectives: The Diversity, Equity, and Inclusion (DEI) Team at a university health science library sought to conduct a language inclusivity assessment of their library’s public-facing website. The project’s objectives were to 1) create a checklist for inclusive language, 2) review the library’s webpages using the checklist, 3) update the webpages accordingly, and 4) circulate the checklist to the university’s other libraries for potential use. Language inclusivity was defined as “language that is free from words, phrases or tones that reflect prejudiced, stereotyped or discriminatory views of particular people or groups” (British Columbia Public Service).

Methods: First, the team searched for literature relating to best practices for language inclusivity. Nineteen useful resources were located, from which the team compiled a 32-item checklist, covering demographic information collected in webforms, as well as identification of gendered language, stereotypes, ableist language, and out-of-date terminology. With this checklist, the team reviewed the 33 webpages of the library’s website, not including LibGuides or links to external webpages. Each webpage was assessed by one of five team members, who used a spreadsheet pre-populated with the checklist to record language that needed to be changed. The team also made a note of DEI-related content that could potentially be added, based on the language inclusivity literature we had consulted before beginning the assessment.

Results: Nineteen instances were recorded for which improvements needed to be made, encompassing the following topics: adding pronouns for employees who opt in; referring to invisible disabilities on our “users with disabilities” page; using student-first language instead of faculty-first; spelling out acronyms; and removing outdated language with negative

connotations such as “earmarked”, as this term has been associated with enslavement. The team’s recommendations for website content additions included creating a DEI statement; developing a code of conduct for the library’s online and physical spaces; and adding information about parking, assistive technologies, and emergency evacuation procedures to our “users with disabilities” page.

Conclusions: The website updates are currently underway and the checklist has been circulated to the six other university libraries as planned. The next stage of the project is to expand the website review to include the library’s Archives pages; this poses the additional challenge of recognizing and appropriately delineating historical language where appropriate, so our library’s Archivist has joined the project team. Following completion of the Archives pages, the DEI Team plans to use the checklist to begin assessing our library’s LibGuides, signage, and non-digital flyers and forms.

Discussing artificial intelligence tools with information seekers: insights from practice

Practice Area: Information Services

Kate Nyhan MLS, IPI - Research and education librarian for public health, Yale University / Cushing/Whitney Medical Library, New Haven, CT

Background: This poster grows out of failure: my attempts to persuade researchers to use active machine learning tools for screening in systematic and scoping reviews. The novelty of AI in evidence synthesis methods is a real and perceived barrier, but other applications of AI already exist and are adopted with enthusiasm by information seekers. In this poster, I explore AI tools relevant to medical library users, including innovative tools (summarization engines and citation sentiment analysis), tools so common that we hardly think of them as “AI” (relevance ranking, article recommendation engines, and automated indexing recommendations), and tools relevant to evidence synthesis.

Description: With the ultimate goal of demystifying AI, I explore tools including Scholarcy, Scite, ASReview, ContReview, NLM’s Medical Text Indexer, Embase’s automatic indexing algorithm, and AI features embedded in databases including PubMed, Scopus, and Web of Science. For each AI tool, I describe the purpose, target users, ease of use, limitations, and potential value, based on my experiences using these tools and discussing them with students and researchers. These examples will demonstrate that AI is not the future, but the present; and that library users can come to trust librarians for user-centered advice about how to use AI tools effectively.

Conclusion: The buzzwords deep learning, machine learning, and AI surround us, and libraries are creating novel applications such as recommendation engines and chatbots. But while a few librarians are building AI tools, many librarians are giving library users advice about

using AI tools -- some familiar and some innovative. In the context of reference, liaison, and research support, librarians have opportunities and responsibilities to help information seekers choose and use innovative AI tools, to recognize AI tools that already feel familiar, and to evaluate the familiar tools as well as the innovative ones.

Effectiveness of Evidence-Based Medicine Instruction in Undergraduate Medical Education

Practice Area: Clinical Support

Dan Stuart AHIP - Librarian, Clinical Faculty Associate, Preston Smith Library / Texas Tech University HSC, Lubbock, Texas

John Griswold - Texas Tech University HSC, Texas

Objectives: While literature exists appraising evidence-based medical (EBM) education, only a limited amount has examined the effectiveness of undergraduate clerkships. This study assesses undergraduate clerkships during the surgery rotation at a medical school by evaluating resident experience with prior EBM instruction.

Methods: Two separate electronic questionnaires were developed for submission to five recent graduating classes and their residency program directors. Evaluation of the results was gathered from both residents and directors on EBM competencies.

Results: Results were compiled using electronic surveys in Qualtrics. These surveys indicate a positive correlation between resident approaches to clinical interventions. They also reflect the value of EBM education for residents and their program directors.

Conclusions: The incorporation of EBM coursework in the third-year surgery rotations positively impacts the research practices of medical professionals in residency. Based on feedback from both residents and their directors, EBM instruction facilitate a better approach to clinical situations.

The Empathy Project & COVID-19: Reducing Barriers through Online, Case-Based Training Modules for Library Workers

Practice Area: Professionalism & Leadership

Eugenia Opuda - Health & Human Services Librarian, Dimond Library, University of New Hampshire, Portland, Maine

Megan Bresnahan - Life Sciences and Agriculture Librarian, University of New Hampshire, Concord, New Hampshire

Benjamin Peck - First Year Experience and Student Success Librarian, University of New Hampshire, Portland, Maine

Elizabeth Fowler - Research and Instruction Services Coordinator, University of New Hampshire, Durham, New Hampshire

Kristin Dhabolt - Web Content Manager, University of New Hampshire, Durham, New Hampshire

Jenna Riley - Project Coordinator, Institute on Disability, UNH, Durham, New Hampshire

Background: Underrepresented and marginalized communities experience barriers to seeking assistance in library settings. The COVID-19 pandemic intensifies these challenges and further underscores social inequities and bias. Libraries, as centers of community, must respond empathetically, equitably, and inclusively as they maintain physical and virtual services amid a pandemic. This poster will describe the development of an open and inclusive online training, at the University of New Hampshire (UNH), about empathy and compassion fatigue in an academic library setting. The poster will also note how that project serves as a foundation for building an expanded training on the same topics, funded by a IMLS CARES Act grant, which will be open to all libraries and museums nationwide.

Description: A team of library and academic technology workers at UNH received internal campus funding to develop self-guided, open, and interactive online empathy training modules using video, text-based case studies, and interactive tools for workers at UNH Library. With guidance from a prominent library social justice consultant and collaboration with a social justice acting troupe, the team created modules to highlight marginalized voices, provide tools to identify and address compassion fatigue, and present strategies for applying social empathy in academic library settings. This internal project provides a structure for an expanded, IMLS-funded training module that focuses on how library and museum workers can use social and institutional empathy to address inequities during a global pandemic. The IMLS-funded Empathy Project, available nationwide in March 2022, will equip participants with the language and tools to respond equitably and inclusively to visitors from marginalized communities. All planning materials will be openly available for others to use to replicate and adapt.

Conclusion: This poster will describe the lessons learned from the development of the UNH training modules and the next steps for the IMLS-funded Empathy Project. By learning about the overall approach for the two projects, poster session participants can reflect on the practice of empathy as part of library services and be aware of future training opportunities. Participants will be able to: 1. Acknowledge the impact of COVID-19 on the mental health of historically marginalized populations and how libraries and museums may contribute to those impacts; 2. Explain how to apply skills, strategies, or models to implement institutional and social empathy; 3. Self-identify and mitigate compassion fatigue; and 4. Have access to open, online training modules and planning documents to use and adapt for their own needs.

Encouraging Employee Connection and Well-Being in a Virtual Workspace

Practice Area: Professionalism & Leadership

Hannah F. Norton AHIP - Chair, Health Science Center Library - Gainesville, University of Florida, Email: nortonh@ufl.edu, Gainesville, Florida

Ariel F. Pomputius AHIP - Health Sciences Liaison Librarian, University of Florida Health Science Center Libraries, Gainesville, FL

Background: When the University of Florida Health Science Center Libraries (UF HSCL) closed our physical space to address the global pandemic in March 2020, library employees struggled to connect while physically isolated and interacting strictly virtually. While the virtual workspace quickly became very robust and efficient under these unusual circumstances, employees experienced difficulties and disconnect, as noted in meetings and through anonymous surveys. Administration and the Wellness Team at UF HSCL found innovative ways to encourage communication and build camaraderie among library employees. Consistent messaging, games, and collaborative efforts helped employees feel less alone while working in their socially-distant environments.

Description: In order to address employee concerns about their lack of connection, library administration and the Wellness Team created a variety of communication strategies and team-building activities. Library administration gathered feedback through weekly anonymous surveys to get an updated understanding of how employees were coping with remote work and address suggestions and concerns quickly. A member of library administration sent daily emails to employees, including both work-related information and wellness-related links (covering physical, mental, and intellectual health topics). Employees contributed photos based on various fun prompts (e.g. pets, childhood photos) and library-wide meetings began with games of matching employees to their submitted photos. The Wellness Team solicited suggestions for work-appropriate songs for a communal Library playlist and created a Library cookbook based on recipes submitted by employees.

Conclusion: While email and video-conferencing communication among employees working from home was near constant, library administration considered it important that some of this communication consistently address employee wellness. Anecdotally, some employees expressed appreciation for this kind of messaging or for particular links shared. Encouraging fun discussion and laughter at the beginning of team meetings and sharing favorites in collaborative efforts helped employees connect in new ways in a primarily virtual workspace that they may not have been connecting before while in a physical space. These innovative efforts used the shift in workspace as an opportunity to bring employees closer at a time when they were intentionally distant.

Evaluating a Current Awareness Search Service

Practice Area: Information Services

Nancy A. Clark MLS - Director, Library Network Office, Dept of Veterans Affairs, Dallas, Texas

Edward J. Poletti AHIP - Chief, Learning Resources, Learning Resources, Little Rock, Arkansas

Priscilla L. Stephenson AHIP, FMLA, AHIP, FMLA - Library Director, James A. Haley Veterans; Hospital, Tampa, FL,

Lisa Beyer - VSFS Intern, Department of Veterans Affairs, Cincinnati, Ohio

Sarah L. Carnes - Health System Specialist, Bedford VAMC, Bedford, Massachusetts

Background: For several years librarians in our VA Library Network (VALNET) have provided current awareness searches on healthcare topics of interest to the agency. All VHA employees may elect to subscribe to these alerts. Depending on the volume of literature, PubMed search results are filtered and emailed on a weekly, bi-weekly, or monthly basis to subscribers. We initiated this project to develop an evaluation of the service to determine the level of satisfaction, relevancy, and perceived impact of the subject searches. Our goal was to assess whether the search service provides value for subscribers and if it should be continued and improved.

Description: A 9-question electronic survey was developed and tested in 2019. Subscribers to three alert topics were surveyed each month between January 2020 and January 2021. Each survey was open for one week. A total of 12,976 subscribers to 34 topic searches received surveys. Respondents were asked to self-identify from a list of health professions. Other questions asked subscribers about their level of satisfaction with the service, whether the alerts provided relevant information, and if the alerts had a positive impact on their work. Results of individual surveys were compiled and analyzed, including free text comments on 2 of the 9 questions.

Conclusion: Respondents indicated that the alerts were relevant to the subject and the participants’ interests (97%) and that they significantly impacted the ability to perform their work (68%). Satisfaction with the alert service was rated good to excellent by 92% of respondents. Subscribers’ comments provided useful suggestions to improve the service. When subscribers requested an easy option to cancel their subscriptions, an updated cover message was added with an opt-out link. Comments on another alert complained about the ‘overwhelming’ number of citations sent with the alerts, prompting a review and revision of the search strategy.

Excluding Animal Studies from Systematic Review Searches: Can We Safely Advise Using a Non-Validated Filter?

Practice Area: Information Management

Celine Soudant - Research Informationist II, Memorial Sloan Kettering Library, New York, New York

Marina Chilov - Research Informationist II, Memorial Sloan Kettering Library, Brooklyn, New York

Kendra Godwin - Research Informationist II, Memorial Sloan Kettering Library, New York, New York

Johanna Goldberg MSLIS - Research Informationist II, Memorial Sloan Kettering Library, Bronx, New York

Objectives: Literature searches for systematic reviews (SRs) should be designed rigorously to retrieve all possible evidence relevant to the research question. To ensure efficiency, they also need to balance sensitivity and specificity. While working on SR search strategies, we have often been asked to apply a filter to exclude animal studies. We set out to bring awareness to other librarians and SR teams on the rigor of applying the filter to their searches by testing three literature searches in two databases to determine whether this filter effectively retains human studies.

Methods: We tested two in-house searches from our librarians and one search from a Cochrane review. We ran each search in Embase (Elsevier) and either PubMed or MEDLINE ALL (Ovid), and adapted the filter listed in the Cochrane Handbook for each database. After running the searches with and without the filter, we exported the excluded references from each search and database instance into its own review within Covidence. Two independent reviewers screened the titles and abstracts for potential studies with human participants and conducted the full-text screening of the remaining records. Conflicts at each stage were reached by group consensus. We downloaded the Covidence data of included studies to an

Excel file, extracted the subject headings from the database where each record was found, then compared this indexing with the record in other databases.

Results: We screened 568 records and identified 63 studies as having human participants. Of the 63 studies, 61 were from Embase (53 conference abstracts, three conference reviews, four articles, and one review), and two from MEDLINE ALL (one letter and one article). One article was incorrectly indexed in both Embase and MEDLINE ALL. Three articles were incorrectly indexed in Embase and correctly indexed in MEDLINE ALL. The letter was correctly indexed in Embase and incorrectly indexed in MEDLINE ALL.

Conclusions: Our small sample set of 63 studies did not reveal one subject heading that could be added to the filter to improve performance. As most of the wrongly excluded studies are conference abstracts from Embase, using the filter would be a lesser problem for SR teams excluding conference abstracts. SR teams should be aware that all Embase articles undergo automatic indexing when in press or in process, but conference abstracts do not undergo later manual indexing. The issue with wrongly excluded studies that are not conference abstracts might be mitigated by searching across multiple databases.

Experiential Education Goes Virtual: Challenges Turned Into Opportunities

Practice Area: Education

Mariana Lapidus - Reference Coordinator, MCPHS University, Boston, Massachusetts

Background: The current pandemic has significantly disrupted the everyday activities of healthcare institutions across the United States, affecting students’ advanced pharmacy practice experiences (APPE). Most pharmacy students were no longer able to be physically placed on their practice sites. In order to meet the accreditation standards, the Accreditation Council For Pharmacy Education has allowed virtual APPEs to be incorporated into the schools of pharmacy curriculum. This poster describes methods of integrating library instruction in virtual APPE education and making it an essential component of the curriculum, focusing on the advantages of the remote delivery format, the sessions’ content, and technology utilized.

Description: The Library liaison to the Department of Pharmacy Practice has been offering small-group review sessions for students on clinical rotations for the last 15 years. Though revisiting the essential drug information resources significantly benefited pharmacy students’ knowledge retention and increased their chances to perform well, only two pharmacy faculty members have incorporated the library instruction as the required component into their APPE curriculum. The new synchronous virtual rotations format presented a unique opportunity for the pharmacy librarian to reach out to a wider group of faculty and transformed the library review into the 1,5 hours required virtual session for the 6th year pharmacy students. Before the new format delivery was implemented, only 16 students were able to attend the library

reviews annually, compared to almost 300 students who took advantage of the virtual content delivery during the last year.

Conclusion: Due to the productive collaboration between library and pharmacy faculty, the number of pharmacy students who have benefited from attending virtual drug information review sessions conducted by the librarian in 2020 has significantly increased. Though no formal assessment of the sessions has been done, feedback provided by students and faculty orally and by email was extremely positive. The virtual sessions’ content and the instruction methods were highly praised by several pharmacy faculty members, who have recorded the review and made it available for their students on Blackboard. Synchronous virtual content delivery allows academic librarians to reach out to a wider audience of learners and successfully integrate information literacy instruction into a variety of courses, including experiential education.

Exploring the Expectations and Preferences of Users in Regards to Health Sciences Library Subject Guides

Practice Area: Education

Anna Biszaha - Assistant Professor/Research & Education Librarian, The Ohio State University Health Sciences Library, Columbus, Ohio

Objectives: Many libraries maintain a series of online subject guides to provide resources and information to their patrons. There is a general consensus that guides are considered most effective when they are appropriately tailored to the needs of the target users; however, such guides can take considerable time and effort to create. As such, it is imperative to critically evaluate and reflect on their effectiveness. This study aims to explore and describe user’s expectations and preferences in relation to subject guides and perform an initial investigation into whether student and clinical users might engage with the guides differently.

Methods: An anonymous survey was administered to collect data about user’s prior experience, expectations, and preferences regarding the subject guides. For those with prior experience using the guides, respondents were asked specific questions about the last time they used a guide, as well as broader questions about what they expect from the guides in general. For those participants who had not used a guide in the past, they were asked a series of questions about how they might imagine a hypothetical “library subject guide”. All students, faculty, and staff within the designated health sciences colleges and the medical center were eligible to participate in the survey.

Results: 206 responses were collected over the course of 4 weeks. The majority indicated they had used the HSL or its resources before, but not the subject guides. The most common technology used to access library resources was a laptop/desktop. Both guide users and non-

users ranked quick and easy links as the most important feature of a guide; however, they differed when it came to the types of information they expected to find, the overall purpose of a guide, and what motivates them to seek one out. Clinical users represented only 5.8% of the total response pool, making it difficult to compare them to student users.

Conclusions: By considering broad user expectations when engaging with library subject guides, this survey helps provide guidance and insight for those charged with developing them. One of the biggest opportunities for future impact may be simply increasing awareness of the guides and subsequent word-of-mouth about them.

“A Few Words on Data”: Promoting Research Data Management Through Existing Campus Communication Channels

Practice Area: Information Services

Karen D. Barton AHIP - Biomedical Research Liaison Librarian, Duke University Medical Center Library & Archives, Durham, North Carolina

Shadae Gatlin MLIS - Repository Services Analyst, Duke University Libraries, Durham, North Carolina

Ciara Healy MLIS - Librarian for psychology & Neuroscience, Mathematics and Physics, Research and Instructional Services, Durham, North Carolina

Sophia Lafferty-Hess - Research Data Management Consultant, Duke University Libraries, Durham, North Carolina

Lee Sorensen - Librarian for Visual Studies, Duke University, Durham, North Carolina

Background: A research data working group of librarians and other information professionals developed a goal to promote research data management, reproducibility in research, and related library resources and services to researchers across disciplines on a university campus. Five members of the research data working group formed a communications subteam and determined the type, format, and frequency of messages that would be sent and identified specific campus communication channels that reached the target audience. Upon considering potential options for communicating with audiences on campus, the working group adopted a plan to partner with various campus content creators.

Description: The working group's communications subteam members asked gatekeepers of campus communication channels for permission to send messages that could be remixed and redistributed to their respective electronic mailing lists and social media platforms. A workflow was developed in which two short blurbs are written and distributed directly to campus content creators for several communication channels every month. The subteam plans to evaluate how many members of the campus community are reached monthly through this new

communication initiative, the ways in which messages have been remixed for specific audiences, and what value content creators find in this initiative. They will also assess how their communication plan aids the working group in meeting various short and long-term goals as outlined in a logic model.

Conclusion: According to responses to a short survey of campus content creators, in which two-thirds responded, the messages distributed by the research data working group and subsequently by campus content creators have the potential to reach more than 25,000 people. It should be noted that not all messages provided by the working group are shared by every content creator every month; each selects which messages will be appropriate for the audience. Additionally, some members of the campus community may see messages multiple times if following or subscribing to multiple communication channels. Feedback from content creators regarding the value that the messages have for the campus community was very positive and enthusiastic. This initiative to promote research data management and related information assists the working group with meeting its goal to develop strategies for outreach and promotion of RDM and campus research data repository services. The working group concludes that the initiative has been successful thus far, though the team would like to identify ways to increase reach and engagement.

Find the Studies Without the Noise: Validation of the Performance of a Pragmatically-Designed PubMed Methodological Filter

Practice Area: Information Services

Nancy Firchow, MLS - Senior Librarian, California Environmental Protection Agency, Office of Environmental Health Hazard Assessment, Vacaville, California

Melinda Davies, MAIS - Associate Research Informationist, Kaiser Permanent Center for Health Research, Portland, Oregon

Objectives: Comprehensive review searches can generate a high volume of results, including many non-empirical publications that present no original patient/participant research data and will be screened out during the title/abstract review phase. Using a set of included studies from previously published systematic reviews (SRs), we demonstrate the relative recall performance of a pragmatically-designed PubMed search filter, the Non-Empirical Publication (NEP) filter, intended to distinguish and exclude non-research publications from comprehensive searches and still retrieve publications of interest.

Methods: We identified 32 SRs published in 2017 and indexed in PubMed which reported their included studies. The focus of the SRs included treatment, epidemiology, diagnosis, and other. The SRs included studies of a broad range of methodologies, including RCTs, non-randomized

clinical trials, observational studies, and case reports. We measured the ability of the NEP filter to retrieve the PubMed-included studies included in these reviews.

Results: The 32 SRs included a total of 1172 studies, of which 1062 were found in PubMed by known-item searching. The NEP filter demonstrated a relative recall of 0.98 across all 32 reviews. Relative recall for SR subsets was as follows: Treatment SRs - 0.97; Epidemiology SRs - 0.98; Diagnosis SRs - 0.98; Other SRs - 0.99

Conclusions: The NEP filter performs very well in retaining studies of interest using a relative recall method. It holds its high recall rate across SRs of different types and across included studies of a broad range of methodologies. Work is continuing to establish sensitivity, precision and reduction in number needed to read.

A Flipped Classroom Approach: Transforming Library Instruction for Evidence-based Occupational Therapy

Practice Area: Education

Leah Cordova - Assistant Professor | Research and Learning Services Librarian, University of Tennessee Health Science Center, Memphis, Tennessee

Background: 2020 proved to be a year of both struggles and opportunities. Many librarians across the United States switched gears and adapted services to better support the needs of their users in an online environment. This poster outlines a liaison librarian’s integration of library instruction in an online evidence-based occupational therapy course utilizing a flipped-classroom approach.

Description: Prior to 2020, library instruction sessions for evidence-based occupational therapy courses consisted of traditional librarian-led lectures with students begrudgingly participating in active learning activities. The pandemic afforded this librarian an opportunity to transform what was once didactic lectures into interactive learning modules applying a flipped-classroom approach to instruction. Modules were created using LibWizard and consisted of self-paced reading, instructional videos, guided learning, and reflective questioning. The content of the modules included breaking down clinical cases into a searchable clinical question, developing database searches utilizing a PICO table, searching for literature in relevant databases, and reporting search strategies on the course worksheets. Completion of the modules before the scheduled synchronous session was required by the course instructor. Additionally, following the completion of the modules, students were asked to pose questions for the librarian on the course’s online discussion board.

Conclusion: Utilizing a flipped-classroom approach allowed for students to engage with the lecture content before the synchronous session, allowing for a more interactive in-class session and deeper learning experience for the students. Synchronous sessions consisted of a brief recap from the librarian and then an otherwise open discussion from the students followed by an open workshop. The librarian-led content for the synchronous sessions was tailored to the unique needs of the students in the course based upon their module activities

and discussion board posts. Through this project, the librarian gained invaluable insight into the information needs of the occupational therapy students and was able to form more meaningful connections with students than previous lecture-style sessions would have allowed.

Fostering a Platform Agnostic Data Discovery Community: The Data Discovery Collaboration

Practice Area: Information Management

Michelle Yee - Data Catalog Coordinator, NYU Health Sciences Library, New York, New York

Nicole Contaxis - Data Librarian, Lead of Data Discovery, NYU Health Sciences Library, New York, New York

Donna S. Gibson - Director of Library Services, Memorial Sloan Kettering Library, New York, New York

Background: The Data Discovery Collaboration (DDC), formerly known as the Data Catalog Collaboration Project (DCCP), was rebranded in February 2020 to become a platform agnostic community aiming to support the discovery of data and other research outputs. Restructuring the organization to focus on interoperability between systems, skill and knowledge sharing, and community support, the DDC consists of librarians, developers, and other information professionals who work together to address challenges related to data discovery, including metadata development and standards, software development, data cataloging practices, and advocacy for improving data discovery across the research community.

Description: Through 2020, the DDC worked together to establish a governance structure that would better facilitate collaboration, disperse leadership responsibilities, and move towards a platform-agnostic community as opposed to focusing on one data discovery system. To accomplish this goal, the DDC organized a retreat in February 2020. Two representatives from each member institution were invited to participate in structured discussions that were organized with the help of change management consultants. The retreat culminated in the creation of a Steering Committee, tasked with realizing the goals set out during the retreat. The Steering Committee met throughout the year and accomplished the following goals: created four Core Groups to address specific needs, developed new membership requirements both for institutional and individual members, and established decision making protocols. Feedback was solicited throughout this process, and the new structure was shared in February 2021.

Conclusion: As members of the DDC embrace the new governance structure, the group plans to measure how the new structure has or has not helped build a supportive and engaged community, including updates made to shared code, changes made to shared metadata schemas, metadata standards investigated and implemented, co-authored publications, co-

authored conference presentations, and assistance provided between members to support and advocate for data discovery at local institutions and more broadly. Providing mutual assistance to individuals and institutions as they contend with unique environment and data sharing use cases, the DDC aims to provide the tools and space needed for a welcoming and dynamic community of information professionals interested in improving data discovery.

Graphic Medicine and Diversity, Equity, and Inclusion

Practice Area: Information Services

Renee A. Torres - Instructional Associate, Coastline College, Yorba Linda, California

Background: This poster examines how Graphic Medicine resources, titles, and workshops can expose individuals to diverse voices and lead to ongoing discussions related to diversity, equity, inclusion, and social justice within healthcare. Individuals can connect with this visual storytelling on an impactful emotional level. By featuring diverse titles, readers can begin to look at healthcare related stories from different perspectives. Providing access and programming related to Graphic Medicine addresses the goals of universities and hospitals to be more equitable and inclusive, leading to better interactions between clinicians and patients.

Description: This preliminary project was funded through the Network of the National Library of Medicine (NNLM) Pacific Southwest Region (PSR) BIPOC LIS Student Professional Development Award. The purpose of this award is to promote diversity, equity, and inclusion within health science librarianship and the project associated with this award puts forth the argument that Graphic Medicine can be used in healthcare and health sciences education to help nurture culturally competent care and empathy. Work on this project began by investigating available Graphic Medicine titles within the existing collections, formulating a library research guide, and presenting the findings to the library staff. Upon completion of this, a collaborative workshop was developed and presented during the Diversity, Equity, and Inclusion Week at the university with the goal of highlighting how this unique medium explores DEI themes to encourage reflection and growth.

Conclusion: Highlighting Graphic Medicine collections, conducting outreach related to it, and collaborating with LIS professionals helps support, expand, and improve access to approachable and thought-provoking materials that support an institution’s long term goals for inclusive education and healthcare. Graphic Medicine outreach, initiatives, and workshops have the potential to spark conversations and reflection related to DEI and allow for dynamic collaborations between library staff, faculty, students, and other stakeholders. This preliminary project demonstrates how LIS professionals can contribute to ongoing initiatives to address issues related to equity and social justice.

Gross Anatomy in the Library: Evaluating the Impact of Anatomical Models in Academic Health Sciences Libraries

Practice Area: Education

G.J. Corey Harmon, MA, MSLS AHIP - Head of Access Services, Laupus Health Sciences Library, Greenville, North Carolina

Mary Roby - Assistant Director for User Services, Laupus Health Sciences Library, Greenville, North Carolina

Background: Laupus Library began its anatomical model collection in the late 1980s with one model. In intervening years, the collection has grown to 236 models ranging from single organs to entire bodies. As of March 25, 2021, the circulation count for the models was 21,571. During the 2018-2019 academic year, we observed that several of the models had become too damaged to continue circulating and we evaluated whether to replace them or slowly discontinue the service. Based on circulation numbers, as well as anecdotal evidence, we chose to replace the damaged models and expand the collection further.

Description: We supplemented our previously collected data with empirical methods to provide additional evidence to inform future decisions about continued investments in the anatomical model lending program. We examined the types of patrons using the models, each model’s usage statistics, and their cost per use. As a comparison, we looked at the statistics for our virtual anatomy subscriptions. We surveyed the students who check out the physical models regarding their assessment of this program’s value to them. Finally, we surveyed all Association of Academic Health Sciences Libraries (AAHSL) members about their physical model collection and its usage in addition to searching the literature to find other evidence of the value that models provide in health sciences education.

Conclusion: Our data shows that 80.39% of our models are checked out by undergraduates at our institution while graduate students account for 13.72% and faculty/staff make up 3.17%. Nursing students accounted for 62% of the model checkouts and 91% of students surveyed believed the models positively impacted their learning outcomes. We also surveyed all AAHSL libraries (n=181) and received responses from 89 (49%). Nearly 61% reported having at least one model with 9 having over 100 models. 77.53% reported having e-Anatomy resources as well. Responses about circulation ranged from hardly at all to several thousand per year. The model collection is a high use distinctive feature of Laupus Library and the data supports our decision to invest in it.

Health Literacy Initiatives for Library Employees

Practice Area: Information Services

Semhar Yohannes - Science Reference and Instruction Librarian, University of Maryland, Baltimore County (UMBC)/Reference Department, Baltimore, Maryland

Aimee Plaisance - Catalog Librarian, University of Maryland, Baltimore County (UMBC)/Technical Services Department, Baltimore, Maryland

Background: Shortly after the Library’s employees began to work from home due to the COVID-19 pandemic, a new health literacy working group was formed to explore how best to use the Library’s resources to respond to the current public health crisis. The working group’s mission was to improve the health of library employees by making health information easy to understand and use.

Description: In this poster, we describe the initiatives implemented by the working group since its inception, including creating a charter, co-sponsoring a health literacy article discussion series, publishing an online semesterly newsletter, and implementing a health literacy lecture series. We also discuss our reasons for creating an in-house working group rather than depending on campus health literacy resources, our experiences after the first full semester of this working group being active, and our plans for future initiatives and possible collaborative partners.

Conclusion: The initiatives from the first semester were well-received and met the goals laid out in our charter. We will continue to practice our current initiatives and expand to other activities as time and resources allow.

Health Literate Patient Education Handouts to Reduce Health Disparities During the COVID-19 Pandemic

Practice Area: Clinical Support

Sallie Willcox - Patient Education Librarian, NYU Health Sciences Library, New York, New York

Anne Foltin - NYU Health Sciences Library, New York, New York

Estella Natal - Health Literacy Specialist, NYU Langone Health, New York, New York

Background: The Covid-19 pandemic has created a need for people to access, understand, apply and internalize health information rapidly. It is essential that patients be Health Literacy

proficient - to be able to obtain, comprehend and take action to prevent Covid-19 contagion and/or avoid spreading it as well as changing everyday behaviors and practices. As this was a novel coronavirus, patient education information needed to be created and disseminated expediently.

Description: The Health Sciences Library and Patient Education Dept recognize that patient education materials in Plain Language and other Health Literacy best practices ensure that patients from vulnerable populations understand Covid-19 information and precautions. Physicians, nurses, librarians and health literacy specialists teamed up in a swift and highly coordinated manner with clinicians and administrators from various areas and departments to create our growing collection of handouts. Accessible across campuses through our digital Patient Education Handouts Library and via the EHR, many of these handouts were translated into several languages (Arabic, Chinese, Russian, Spanish, etc.) for enhanced accessibility to more patients and families.

Conclusion: Covid-19 has been disproportionately affecting communities of color and other vulnerable populations. When patients from these populations have understandable and accessible patient education information, they are empowered to keep their families safe and their neighborhoods as well. These efforts diffuse into the community at large and can mitigate spread, despite larger systemic issues that can make these communities more susceptible. These efforts are essential for reducing health disparities. The library and Patient Ed Dept. intend to expand their goal of helping make the parent institution a Health Literate Health Care Organization – creating and providing access to patient-centered materials for improved health outcomes and health equity.

Health System Growth and the Effects on Academic Libraries – A Pilot Study

Practice Area: Professionalism & Leadership

Kelly Thormodson - Associate Dean and Director, Penn State University Harrell Health Sciences Library, Hershey, Pennsylvania

Lori B. Cisney - Collection Development and Digital Resource Management Librarian, Harrell Health Sciences Library, Hershey, Pennsylvania

Benjamin Hoover - Associate Director and Coordinator of Innovative Technology Services, Harrell Health Sciences Library, Hershey, Pennsylvania

Objectives: The growth of health systems effects libraries in multiple ways: 1. adds to the number of beds as well as the physical locations, 2: adds to the number of physicians, residents, primary caregivers and other allied health care workers as well as general staff and 3: these additions can impact technology and budgets as well as library services and

collections. This pilot study aims to gather and analyze specifically what are the barriers, challenges, misunderstandings and successes to academic libraries affiliated with growing health systems and to evaluate whether a larger study would be beneficial to the profession.

Methods: A qualitative focus group with 4 library directors (or their designate) will occur. The focus group will be over Zoom and will not be recorded but the facilitator will document the data shared. The research will be analyzed for common denominators and to look at whether a larger study should be undertaken. This research is important to academic library leaders that need to adhere to resource contracts and balance budgets but as well to liaisons who are in the tough position of having to evaluate who they can help and what they can do within the agreements set forth by such institutions. The Association of Academic Health Science Libraries (AAHSL) currently has a task force looking at best practices for libraries in growing health systems and agreed this pilot study was complementary to their work and not duplicate.

Results: Growing health systems create a variety of challenges for academic libraries. Those areas affected by this growth include: technology and systems, communications, politics, collections, services and funding. The findings show that a larger scale survey to collect date more broadly would be beneficial as each institution is so unique in its approach to these issues.

Conclusions: The findings show that further large-scale research needs to be done but also there are many smaller facets that would benefit from exploration as well. Specific questions about best practices for licensing resources have been tackled by AAHSL but many technology and services challenges are among the questions that still need to be studied. Questions such as who is responsible for being the gatekeeper to library access? Which services are provided to all vs affiliates vs clinical patrons?

Highlighting Faculty Research: The Researcher Profile Audit Service

Practice Area: Information Management

Tom Harrod AHIP - Research Support Librarian, Himmelfarb Health Sciences Library, Washington, District of Columbia

Sara Hoover - Metadata and Scholarly Publishing Librarian, Himmelfarb Health Sciences Library, Washington, District of Columbia

Background: Numerous systems have emerged which capture a researcher’s scholarly output, including ORCiD, Scopus, Web of Science, and Google Scholar. When looking for information on faculty publications, we frequently find inaccurate and incomplete profiles as well as duplicate profiles. Having accurate researcher profiles is important for differentiating publications of authors with similar names, complying with funder mandates, and demonstrating impact for promotion and tenure. Consequently, the Himmelfarb Library sought

to help researchers take control of these profiles. The objective of this poster is to demonstrate how the Researcher Profile Audit Service helped researchers improve the quality of their author profiles.

Description: The Himmelfarb Health Sciences Library at the George Washington University serves the needs of students, faculty, and staff from the School of Medicine and Health Sciences, the School of Nursing, and the School of Public Health. As a result of the challenges outlined above, librarians at the Himmelfarb Library developed a service to help patrons improve their researcher profiles, the Researcher Profile Audit. We identified six platforms (Scopus, ORCID, Web of Science, Google Scholar, Publons, and ResearchGate) as our initial area of focus. We created a service description, intake form, and dissemination strategy for a six month beta test. The Researcher Profile Audit Service was launched on November 4th, 2020 and was offered to all faculty. Recipients of pilot awards from the CTSI-CN were specifically invited to participate as a part of their program. To date, we have performed 32 audits.

Conclusion: 2020 saw an unprecedented growth of scientific journal content thanks to research associated with the Covid-19 pandemic. More than ever it is important for researchers to have accurate researcher profiles. While still in the beta phase, our Researcher Profile Audit Service has received positive feedback from faculty. Librarians working with the Researcher Profile Audit Service are reconceptualizing the services that libraries provide in order to help make institutional content more visible, while also building new relationships with faculty. This service reinforces the central role that the library can play in regard to the dissemination of content in emerging systems.

A holistic approach to anti-racism resources in Medicine, Nursing, & Health Sciences: From the workplace to the classroom

Practice Area: Education

Jillian Silverberg - Research & Instruction Librarian, Edward & Barbara Netter Library, Quinnipiac University, West Hartford, Connecticut

Rachel C. Lerner AHIP - Reference & Instruction Librarian, Edward & Barbara Netter Library, Quinnipiac University, Hamden, Connecticut

Background: Part of the university’s 10-point plan to advance racial justice is “advancing equity, inclusiveness, and anti-racism as cornerstones of who we are.” As part of that initiative, the library discussed creating a resource that would target institutional needs. Simultaneously we were formally approached by multiple Diversity, Equity, and Inclusion-focused groups to pursue a similar project. Based on those stakeholder conversations, librarians designed a guide using democratic workspace (covering both academic and clinical settings) and anti-racist pedagogical materials as cornerstones of a holistic anti-racism resource guide. Content was consciously grounded in the context of Medicine, Nursing, & Health Sciences.

Description: In late spring 2020, the library received requests from University DEI groups to share resources & create a space to house information on anti-racism. Librarians evaluated subscription resources available through the University, assessed similar resources from other institutions, and searched for tools from organizations. Re-occurring themes and information gaps in existing resources were identified, and six topic areas were selected: Pedagogy, Academic Workplace, Bias, Disparities in Healthcare, Inclusive Dermatology, and Racism in Clinical Care. Ultimately, the guide provides resources that address and confront racism and bias within the curriculum (with resources for faculty to use in the classroom), the academic workplace (including how to create democratic workspaces and how to be an ally), and, more specifically, within the disciplines of Medicine, Nursing, & Health Sciences.

Conclusion: An active effort was made be sensitive to the topic and acquire a diverse range of input, including feedback from representatives from each school, the faculty senate, and DEI committees on campus. Suggestions have been and continue to be incorporated, including adding in a new section titled “Historical Perspectives in Medicine, Nursing & Health Sciences.” We intend to evaluate user metrics at the resource level. We hope to identify the most frequently used resources across the guide, as well as for each individual section. We will conduct focus groups among faculty and staff to assess usability and integration within the curricula. These are planned for the end of the spring semester.

Identifying the most important databases for "One Health" Related Systematic or Scoping Reviews

Practice Area: Global Health & Health Equity

Erik Fausak - Student Services Librarian, University of California, Davis, Davis, California

Objectives: Identify the most important databases to search when conducting a One Health Systematic or Scoping Review.

Methods: Methods include four approaches: 1) Identify any scoping or systematic reviews that have One Health as a topic and record what databases have been used. 2) Conduct a search of journals on the One Health Platform (and if applicable, other One Health resources that list important journals) to see what databases cover the largest quantity of the listed journals. Ulrich's Periodical Directory will be primarily used to identify database indexing.

Results: 114 synthesis reviews were selected across 5 databases (PubMed, Scopus, Biosis, CabDirect, and Web of Science Core Collection. Highest percentage databases used in these reviews were the following (percentage of total databases): Medline (83% via PubMed) 24% Web of Science Core Collection 14% Google Scholar (Search Engine) 10% Scopus 8% Embase 4% Cab Abstracts + Global Health 4%. The second method could only identify 11 of the 18 journals from the One Health Platform as being indexed. Of the 11 indexed journals, the

following databases have indexed the most of the one health journals: Scopus 82% CAB Abstracts/Global Health 73% Web of Science 45% Biosis 45% Embase 45% PubMed 36%

Conclusions: The One Health approach is extremely important for the future of Public Health. Identifying which databases best support such a multidisciplinary approach will be extremely important to any librarians that are creating One Health guides or conducting a systematic or scoping review. Many of the studies examined had a very inconsistent manner of identifying databases and in some cases, some reviews seemed to use redundant databases like Medline (OVID) and PubMed. Many synthesis reviews utilized a multidisciplinary database and regionally focused databases like African Journals Online.

Immersing Students in Graphic Medicine! Bringing a New Elective to Life

Practice Area: Education

David Charles Duggar AHIP - Head, Library Liaison Program, LSU Health Shreveport Health Sciences Library, Bossier City, Louisiana

Erin E. Ware - Reference Librarian, Health Sciences Library/ Louisiana State University Health Sciences Center- Shreveport, Shreveport, Louisiana

Background: This poster describes the attempts and experiences introducing a new Immersion Elective for freshman medical students as part of the spring 2020 and spring 2021 semesters. Two faculty members from the health sciences library at a public medical school combined talents to present an eight hour elective for up to eight freshman medical students to choose as part of the spring semester Immersion course. The project topic, graphic medicine, was new to the school and was based on Comics for Health and Medicine, one of the educational resources from the National Library of Medicine’s 2018 Graphic Medicine exhibit.

Description: Following a call to sponsor and propose projects for Immersion 2020 in late October a proposal topic was submitted in mid-November, which was immediately requested in final proposal form by end of the month. The project’s title, goal, description, sign-up limit, and coordinator were presented as one of the electives freshman medical students could select as part of their Immersion 2020 courses. Coordinators were notified in early January of class size and attendees names to contact to schedule the preparatory assignments and in-person sessions between January and May. Students were required to complete an evaluation in mid-May about each course taken as part of their Immersion 2020 experience.

Conclusion: Three students selected Graphic Medicine for Immersion 2020. Three in-person sessions were planned but due to the pandemic only one was held. All three preparatory assignments were distributed. Student feedback was favorable for revising to offer as part of Immersion 2021. Following revisions, Graphic Medicine was again offered as an elective. Six

students have selected Graphic Medicine for Immersion 2021. Three in-person sessions are planned during February – April. Results will be presented in the poster.

Impact of Covid-19 on Academic Health Sciences Library Programs and Services

Practice Area: Innovation & Research Practice

Bart Ragon - Interim Director, Claude Moore Health Sciences Library, Charlottesville, Virginia

Elizabeth C. C. Whipple - Assistant Director for Research and Translational Sciences, Ruth Lilly Medical Library, Indianapolis, Indiana

Melissa L. Rethlefsen AHIP - Executive Director & Professor, Health Sciences Library & Informatics Center, Gainesville, FL

Objectives: The purpose of the study is to investigate the impact of COVID-19 on the programs and services provided by Academic Health Sciences Libraries. Findings from the study aim to strengthen what is known about the needs of Academic Health Sciences Libraries during a crisis and identify best practices for responding to future events.

Methods: This multi-site study will use a mixed-method approach to capture the evolution of Academic Health Sciences Libraries during this quickly changing crisis. The study involves a qualitative survey administered using Qualtrics to Academic Health Sciences Libraries to capture current evolutions of programs and services. Consent for the survey was collected as part of the survey instrument. A follow up survey was administered to track updates from Academic Health Sciences Libraries as the situation continued to evolve. A phenomenological approach was utilized to better develop a shared experience of academic health sciences libraries throughout the pandemic. The data was analyzed using DeDoose, an online platform for analyzing qualitative and mixed methods research. Two researchers coded emergent themes from the data, with a third researcher verified a sample of the coding to ensure inter-rater reliability.

Implementing a Novel Service Model for Writing Support at an Academic Health Sciences Library

Practice Area: Information Services

Lily Martin - Reference & Instruction Librarian, Levy Library, Icahn School of Medicine at Mount Sinai, Ridgewood, New York

Rachel Pinotti AHIP - Director, Library Education & Research Services, Levy Library, Icahn School of Medicine at Mount Sinai, New York, New York

Background: Writing and publishing are essential skills for careers in academic medicine and biomedical science. While the library has many services to support different aspects of the research and publication process, there was a lack of support for the writing aspect of the process. Furthermore, as a standalone graduate school, there was no writing center to which we could refer writing help inquiries. To address this gap, we set out to develop a Writing Support Service aimed at helping students and trainees at our institution improve the flow, structure, and style of their writing in a collaborative and confidential setting.

Description: We developed a proposal which included a needs assessment with SWOT analysis, an implementation and assessment plan, a budget, and a timeline. Our proposal was greeted enthusiastically and greenlighted by library leadership. Using various LibApps, we developed a website (LibGuide), an appointment request form (LibWizard), a post-consultation satisfaction survey (LibWizard), and a service utilization tracking form (LibInsight). The website clearly lays out service parameters and expectations for users of the service as well as policies regarding eligibility, scheduling, and cancellation. Appointments are provided by a qualified consultant with science writing expertise, while service administration (including recruiting writing consultants, promoting the service, and triaging writing appointment requests) is managed by library staff. The effectiveness of the service is evaluated through analysis of usage statistics and user satisfaction surveys.

Conclusion: The library’s Writing Support Service has enjoyed robust and increasing usage since its launch in September 2018. The highest proportion of users have been medical students followed by graduate students and residents/fellows. Writers most frequently utilize the Writing Support Service for help with personal statements and other application-related documents as well as manuscripts intended for publication. Feedback from post-consultation surveys demonstrates that the library’s Writing Support Service has been successful in enhancing the writing skills of students and trainees, with 90% of post-consultation survey respondents indicating that what they learned in the session has helped them improve their writing. The implementation and success of this program demonstrates that libraries can facilitate writing support for students at the graduate level.

Information Literacy Session Attendance and Library Website Visit Frequency: Impacts on Awareness of LibGuides among Undergraduate and Graduate Health Professions Students at an Urban Campus

Practice Area: Education

John Carey AHIP - Head, Health Professions Library, Hunter College Libraries, New York, New York

Ajatshatru (A.J.) Pathak - Health & Informatics Librarian, Health Professions Library Hunter College-City University of New York, Bellerose, New York

Sarah C. Johnson - Social Sciences Librarian, Hunter College (City University of New York), Cincinnati, Ohio

Objectives: This poster compares the impact of visiting the library’s website with that of information literacy session attendance on health professions students’ awareness of library-created LibGuides.

Methods: The data were drawn from a paper-based, in-person survey conducted in 2017 among undergraduate and graduate health professions students at a large, urban, public institution. The researchers recruited 100 health professions students during morning, afternoon, and evening hours at the health sciences campus of Hunter College, a senior college within the City University of New York system. The researchers asked participants to complete a survey inquiring about their use, perceptions, and awareness of Springshare’s LibGuides. The instrument also collected data on participants’ attendance at library instruction sessions, frequency of visits to the library website, field of study, academic year, and demographic factors such as age, gender, and race or ethnicity. The authors used IBM SPSS Statistics version 27 to analyze responses.

Results: About 45% (n=20) of participants who visited the library’s website at least once per week were aware of LibGuides, with awareness dropping to 25% (n=7) among those who visited the website once per month. Among participants who attended information literacy sessions, awareness reached 54% (n=13) among undergraduate and 64% (n=9) among graduate health professions students. Awareness remained low among participants who had not attended information literacy sessions (21%, n=13) or had never visited the library website (11%, n=1). Results show the influence of gender, age, and race or ethnicity on the association between respondents’ library workshop attendance and LibGuides awareness.

Conclusions: The results have three main implications for urban health sciences libraries serving diverse communities. First, instruction librarians should employ LibGuides when teaching information literacy sessions to increase awareness of these resources among students. Second, library staff should encourage patrons to visit the library’s homepage to find information resources, as many students do not directly go to the library’s site when seeking

information. Finally, library staff should promote LibGuides at orientation sessions of higher education opportunity programs at campuses to increase awareness among students from underrepresented populations.

Integrating Cultural Humility into Online Grant-Writing Instruction

Practice Area: Professionalism & Leadership

Jarrod Irwin - Community Health Coordinator, Network of the National Library of Medicine, Region 1, Baltimore, Maryland

Background: The purpose of this project was to integrate cultural humility concepts into the Network of the National Library of Medicine's existing self-paced, online course about writing grant proposals for health outreach projects. Cultural humility involves ongoing openness to learning from the experiences of others, particularly members of marginalized groups. This attitude is important for educational offerings because it promotes a positive learning environment for participants with diverse backgrounds and experiences. Although the connection between cultural humility and grant-writing might not be immediately obvious, the collaborative nature of writing a grant proposal and conducting a grant-funded outreach project suggests that cultural humility concepts may be valuable in grant-writing instruction.

Description: A NNLM staff member revised the course's discussions of collaborative work and relationships with partner organizations for grant-funded health outreach projects. The course now emphasizes including people from a health outreach project's target audience in every step of the project, from planning to evaluation. It recommends forming relationships with groups serving these audiences outside of any grant application process, so these partners are not treated as a mere means of winning a grant. The revised course also encourages members of project teams who are from more privileged backgrounds to be open to correction if they say or do something insensitive, because fostering a welcoming environment requires a lifelong learning process that inevitably involves making mistakes. Additionally, microaggressions, such as statements that assume participants come from a specific cultural background, were removed throughout the course.

Conclusion: In the first nine months that the updated course was available, 57 participants enrolled in it, which was an increase of 21% over the same period one year earlier. An evaluation survey at the end of the course found that 100% of respondents who took the updated course reported that it would be useful to their work, compared to 85% of respondents who took the previous version of the course. The course completion rate did not differ between these periods. These results suggest that cultural humility concepts may be seen by participants as a valuable addition to online, self-paced professional-development courses, even those devoted to topics where the connection to diversity and equity is not immediately apparent.

Integrating Library-Related Competencies into Residency Curricula

Practice Area: Clinical Support

Deborah J. Rhue - Clinical Services Librarian, UNM Health Sciences Library and Informatics Center, University of New Mexico, Albuquerque, New Mexico

Jonathan D. Eldredge MLS, PhD - Professor, HSLIC RECIS, Albuquerque, NM

Objectives: Health professions education focuses on ensuring that all graduates possess needed competencies. The Accreditation Council for Graduate Medical Education (ACGME) sets standards and monitors the progress of medical residents as they advance toward medical practice in their specialties. Medical specialties differ on how they define and measure progress in their residents meeting their competencies within a standardized ACGME framework. Clinical librarians need to train medical residents in certain competency areas, but until now, no one has assembled these competency statements in one central place. This project analyzes the competencies related to informatics, library, and Evidence Based Practice (EBP) skills to identify core and optional curricular elements that can be integrated into different medical specialty residencies.

Methods: The authors identified and collected key competency documents from ACGME and those specialties representing 2% or more of the residencies in the US. They compared and contrasted these competencies related to informatics, library, and EBP skills. Most competencies statements were categorized under the fifth broad ACGME competency area of “Practice Based Learning and Improvement.” The authors then used their combined years of experience as a clinical librarian, a graduate medical education associate program director, informaticist, EBP instructor, and curricular expert to design core and optional elements to meet these competencies.

Results: The authors have been developing a generic curriculum for residents that can be adapted to different specialty programs. The curriculum follows a progression from lower-level milestones to more advanced milestones. It also includes optional training modules as needed by specific specialty programs.

Conclusions: The authors will discuss their curriculum at the MLA Annual Meeting.

International Outreach Services at Illinois State Fire Academy Library

Practice Area: Information Services

Lian Ruan - Head Librarian/Director of International Programs, Illinois Fire Service Institute Library, Champaign, Illinois

Shuyi Liu - International Outreach Services Librarian, Illinois Fire Service Institute Library, Champaign, Illinois

Purpose:The poster is to discuss the International Outreach Services at the Illinois Fire Service Institute (IFSI) Library in six areas: Chinese Librarians Scholarly Exchange Program (CLSEP), Visiting Scholar Program, fire services training programs, Zoom activities, research collaboration, and delegation visits from and to other countries.

Description: The CLSEP has been running successfully for 15 years (2005-2019), and 423 librarians from 145 organizations participated in the program. Since 2005, the library has received 144 visiting scholars in LIS, Fire Service, and Emergency Management from 80 institutes in China, Brazil, Netherlands, and South Korea. Since 2005, the library has supported IFSI to deliver 5 training programs and trained 193 first responders in China and at IFSI as well as 2 firefighters from South Korea and 1 fire investigator from Brazil. Since April 2020, the library has organized 23 online activities via Zoom with a total of 507 participants throughout the Pandemic. The library has collaborated with research institutes and published two special issues and a book and coordinated IFSI research collaboration in fire emergency services with China. Since 2006, 35 delegations from China have visited IFSI and IFSI colleagues have been invited to China, Europe, and Southeast Asia, and visited 33 cities in China.

Methodology: Data has been collected and analyzed through surveys from participants, interviews, and observations.

Results/Outcomes: The IFSI Library’s International Outreach Services have built a viable global community of 762 individuals, consisting of 423 librarians, 144 scholars, 195 first responders from 186 organizations that include libraries, universities, research institutes, and fire organizations as programs continue growing. CLSEP has become a well-recognized program in China and one of the largest international librarians training programs in the U.S. Strong partnerships have been developed to support the program, including the university libraries on the East Coast, Midwest, West Coast, and public libraries in the U.S. Most of the librarian participants got promoted after studying in CLSEP. The Visiting Scholar Program has contributed to the scholars’ research and professional careers by furthering close research collaborations. The training in fire emergency services has enhanced local responders’ practices with successful responses.

Keeping Pace with COVID-19 - Federal Library LibGuides During the Pandemic

Practice Area: Information Services

Sarah C. Clarke AHIP - Medical Reference Librarian, Darnall Medical Library, Walter Reed National Military Medical Center, Bethesda, Maryland

Sharon Han AHIP - Associate Fellow, Network of the National Library of Medicine, Los Angeles, California

Emily Shohfi AHIP - Clinical Medical Librarian - Assistant Professor of Medicine, Darnall Medical Library, Walter Reed National Military Medical Center, College Park, Maryland

Objectives: Last winter, the world saw the emergence of the severe acute respiratory syndrome coronavirus 2 (SARS-CoV-2), causing the coronavirus disease (COVID-19). A year later, and cases continue to increase. In the last decade, librarians have been increasing their roles in infectious disease response, as seen during previous outbreaks. There is no existing literature on the use of the widely-used library website platform LibGuides, in response to infectious disease outbreaks. Government agency libraries disseminate information, differing in content by audience. We set out to see how Federal Libraries use LibGuides to distribute COVID-19 information throughout the ongoing pandemic.

Methods: The research protocol and survey were approved by the Walter Reed National Military Medical Center IRB and the Defense Health Agency. Twenty survey questions were designed - two questions screened for participants appropriate for the study, and three questions collected broad demographic information to assist in identifying duplicate responses from individual libraries. Remaining questions focused on the creation, curation, and maintenance of COVID-19 LibGuides. Questions were peer-reviewed by colleagues. The survey was created in Max.gov, a Federal Government data collection and analysis tool. The survey was made available for three weeks, and authors sent invitations to potential participants through emails (including listservs) and personal social media accounts. We exported collected data into Excel to quantify and visualize results. Long form answers were manually reviewed and tagged thematically based on responses. Shared data will be blinded.

Results: Thirty-four percent of respondents reported that their library uses LibGuides; 45% of these respondents said they spent 10+ hours creating their LibGuide, and 60% of respondents spent

Conclusions: We are currently forming/writing our conclusion. Data analysis is ongoing, but initial findings show that, while Federal Librarians do not have universal access to LibGuides, most are curating content specific to COVID-19 through some platform. These LibGuides are used to share COVID-19 information most relevant to a library’s target audience and often include other U.S. Government-created resources. Because of LibGuide’s ease of set-up and

external access, libraries can quickly add content or pivot to meet demands of patrons. Due to the rapid, ongoing response to the pandemic, many respondents have not yet considered long-term plans to preserve COVID-19 LibGuides. Future research may include development of best practices for creating LibGuides as part of library rapid response to public health emergencies.

LibGuides as a Dissemination Platform for Medical Research Grants

Practice Area: Information Management

Simon Robins - Reference Librarian, Northeast Ohio Medical University Library, Rootstown, Ohio

Background: From 2016 to the present, Northeast Ohio Medical University's (NEOMED) Department of Family and Community Medicine has received grant funding from the Health Resources & Services Administration (HRSA) of the U.S. Department of Health and Human Services to improve the primary care workforce by enhancing inter-professional training for primary care clinicians, teachers and researchers, with specific attention to underserved populations, across the care continuum. In late 2019, the grant leadership approached NEOMED Library to create a central website to preserve and disseminate the instructional content created through this grant in a way that would be seamless for the end user.

Description: Using LibGuides, a single site was created to host a collection of modules authored by faculty members who led the grant. Content was organized according to 5 themes (Health Disparities, Population Health, Health Care Transformation, Research, and Provider Wellness). Each theme has its own table of contents, followed by sub-pages containing embedded presentations, video lectures, and other learning objects. At the beginning of the site, a landing page, about page, and team members page with contact information was created to provide participants with context on the project. Branding for NEOMED’s College of Medicine and HRSA were included on these pages. Usability heuristics and information architecture principles were considered when designing the layout and structure of all pages. To ensure seamless access for non-NEOMED participants, this site was not restricted; anyone with the link can access it without having to login.

Conclusion: Since its creation in January 2020, the site has had 2,341 pageviews. The grant team sees this as a success, considering the small group of users who are grant participants. Feedback from these participants was positive, with emphasis on the overall appearance and structure of the site. Once the grant ends in 2022, the site will transition from being a platform for grant participants to access materials, towards a site that is used to store the modules for usage in other adjacent projects, such as faculty development or continuing education. The library sees this as a success and recommends it as a model for other libraries to consider when aiming to preserve and disseminate scholarly content created through grants.

Librarian as Curriculum Mapper on a Curriculum Mapping Team

Practice Area: Education

Heather A. McEwen - COM Curriculum Mapper/Assistant Professor of Family and Community Medicine, College of Medicine, Rootstown, Ohio

Cristina Basaran MAED - Curriculum Resource Manager, Office of Medical Education, Rootstown, Ohio

Background: Curriculum mapping is the process of recording a medical school’s curriculum and providing information about competencies, gaps, and redundancies. It is a tool for curriculum development and integration, accreditation, and evaluation. Librarians have traditionally provided help with creating controlled vocabulary lists for maps. It is less common to have a librarian serve both in the role of curriculum mapper and as the faculty member on the curriculum mapper team. As a mapper, librarian searching, teaching, collaboration, and data management skills are utilized in a non-traditional setting.

Description: A curriculum mapping team shared information input within the map. Course and session level information is added to the map each year along with any relevant keyword mappings. Hierarchies of learning objectives from the program, course, and session levels were created. Learning and assessment sessions are labeled with MedBiquitous controlled vocabulary. The database can be searched for information like other databases. Information from the database can be used in curriculum reports, accreditation reports, and educational grant paperwork. The mapping team works with administration and faculty to provide information needed to determine if topics are covered within the curriculum and determine level of coverage.

Conclusion: Projects completed and deadlines met have been used to illustrate the importance of the information within the database. The major outcome for the map is the completion and submission of the annual AAMC curriculum inventory report. A second outcome included completion of data information requests for College of Medicine-related reports and faculty requests as requested. Lastly, course directors and coordinators checked the accuracy of information added to the map for their courses.

Librarian Engagement with the Cochrane US Network

Practice Area: Innovation & Research Practice

Nina L. McHale AHIP - Head, Education & Research, Strauss Health Sciences Library, University of Colorado, Aurora, Colorado

Lisa K. Traditi AHIP - Deputy Director, Health Sciences Library/University of Colorado Anschutz Medical Campus, Aurora, Colorado

Background: Established in 2019, the Cochrane US Network “works to promote evidence-based decision making in medicine, public health, and healthcare.” The Cochrane US Network is comprised of sixteen affiliate institutions, three review groups, and three satellite review groups, many of them located on medical or health sciences campuses across North America. At the University of Colorado Anschutz Medical Campus, an affiliate and a satellite review group were established.

Description: The CU Anschutz affiliate director and co-director reached out to campus librarians at the Strauss Health Sciences Library, and a fruitful collaboration coordinating and organizing a successful lecture series of Cochrane-related topics was the result. The CU Anschutz review group director also initiated conversations with the Strauss Library, with an eye towards future collaborations. Curious as to if, and how, other librarians were becoming involved with their own affiliates or review groups, the presenters, who are the deputy director and the head of the Education & Research department, created a survey to investigate librarian involvement at other Cochrane US entities located on academic health sciences campuses. This poster presents the results of that survey.

Conclusion: Three-quarters of the survey respondents indicated that they were engaged with the non-librarian Cochrane groups on their campuses; they indicated that they attended meetings, worked on reviews/evidence synthesis projects, and taught or presented about related skills. One-quarter of the respondents indicated that they were not involved with their Cochrane entity, citing the following reasons: lack of time/capacity, gaps in knowledge, and lack of communication. Free text responses were also gathered, and common themes included the opportunity for collaboration, both with the Cochrane units and among health sciences librarians generally; demonstrating the value of librarians’ skills and work within the larger Cochrane community; and concern about capacity to support the Cochrane units in addition to the “normal” work of librarian positions and work/library units.

Librarians Helping to Combat Organizational Health Literacy Through an Updated Health Literacy Assessment Tool

Practice Area: Innovation & Research Practice

Kelsey L. Grabeel AHIP - Assistant Director, Preston Medical Library / Health Information Center, University of Tennessee Graduate School of Medicine / University of Tennessee Medical Center, Knoxville, Tennessee

Robert E. Heidel PhD, Stat - Associate Professor of Biostatistics, Surgery, Knoxville, Tennessee

Sandy Irene Oelschlegel AHIP, Tennessee

Rima Rudd - Harvard School of Public Health

Objectives: Health literacy continues to be a challenge libraries and organizations face. To help address organizational health literacy, the University of Tennessee Medical Center (UTMC) created a Task Force and utilized the Health Literacy Environment of Hospitals and Health Centers (HLEHHC) tool to assess UTMC’s health literacy attributes. When using this tool, it was discovered that some of the questions needed to be updated. This will discuss the process in which the UTMC Task Force and original author Rima Rudd revised and updated the HLEHHC to create the Health Literacy Environment, version 2 (HLE2).

Methods: This update was a two year collaborative process. An extensive literature review on health literacy was performed. Each section of the HLEHHC was reviewed, as well as each question, leading to question updates and removals. Changes were made to the rating scale to better reflect the questions. The new tool undertook a peer review process, followed by more updates. After those changes were made, the tool was pilot tested. Additional updates were then made based on pilot testing.

Results: The HLE2 is composed of five new sections: Organizational Policies, Institutional Practices, Navigation, Culture and Language, and Communication. The rating scale is yes/no as well as a frequency Likert scale. Directions are located at the beginning of each section along with how to score.

Conclusions: Through this collaborative process, Rima Rudd and the UTMC Task Force updated the HLEHHC to include timely questions and specific directions for use. Librarians are at the forefront helping to address the challenge of organizational health literacy. The HLE2 is available for use to analyze literacy-related barriers for organizations.

A Library Curated Pediatric COVID-19 CME and Clinical Decision Support Center

Practice Area: Innovation & Research Practice

Kristina M. Flathers - Manager, Library Services, Medical Library, Wilmington, Delaware

Victoria L. Clifton - Librarian, Mayo Clinic / Nemours Children's Specialty Care, Jacksonville, Florida

Gerald J. Murray - Web Developer, Nemours, Wilmington, Delaware

Background: The rapid pace of publication regarding SARS-CoV-2 (COVID-19) left physicians and researchers grasping for access to accurate, clinically significant information to aid in patient care. Serving as the medical library for a multistate pediatric health system, including 40 primary care practices, pediatricians rely on our services to cut through the clutter and provide evidence-based clinical decision support. Our medical system also provides world class pediatric continuing medical education (CME) related activities for internal and external users. The medical library sought to leverage our relationship with CME to create a curated, bi-weekly updated COVID-19 pediatric clinical and practice support resource center.

Description: Hospitals and primary care practices are facing a constantly evolving health crisis, reevaluating the practice of medicine, pivoting towards telemedicine, altering appointment structures, and working with the CARES act to obtain PPE and financial support. Utilizing an evolving PubMed search, we evaluate new articles on a daily basis to add to our site. Article decisions are based on several factors, including: new information, accessibility, stability of DOI, and article type. Systematic reviews, reviews and research studies are given precedence over viewpoints, correspondence and commentary. The literature selected directly addresses knowledge gaps particular to pediatrics, continually identifying constantly evolving updates to patient care standards by highlighting articles that are particular to: MIS-C, Vaccines, Academic Medicine, and Practice Changing Updates. Utilizing CME site metrics, we experienced consistent growth with 5,908 hits from April through December 2020, averaging 490 unique users per month.

Conclusion: As hospitals became overwhelmed with COVID-19 patients and protocols, the medical literature underwent explosive growth. A basic search of (SARS-CoV-2 OR COVID-19) AND pediatric age keywords, limited to English, provides 12,390 results limited to the 2020 calendar year. We sought to create a central location with curated, free articles to answer clinical questions and support practice changes throughout the pandemic. Leveraging the flexibility and reach of our health system’s CME conference portal to deliver a central location for pediatric-centric COVID-19 literature provided an alternative means to earn CME for clinicians while growing the medical library’s organizational footprint. Future research is

necessary to see if CME relationships are applicable as a marketing tool for physicians outside of a health crisis.

Library Instruction in the College of Pharmacy: A Flipped Classroom Model

Practice Area: Education

Breck Turner - Library Specialist, University of Kentucky Medical Center Library, Lexington, Kentucky

Cayla M. Robinson - Graduate Assistant, University of Kentucky Medical Center Library, Lexington, Kentucky

Background: The purpose of our program was to provide asynchronous, flipped-classroom instruction for Third-Year Pharmacy students to supplement the in-person portion of their course. Five tutorials, covering PubMed, Cochrane Library, ClinicalTrials, SCOPUS, and ECRI Trust, were created around drug information questions. Our aim was to provide a framework for searching to support the evidence-based medicine curriculum and include real, searchable, research questions to simulate an advanced search.

Description: We developed scripts in consultation with the College of Pharmacy liaison librarian to cover database searching skills for evidence-based pharmacy related topics. Pre-test questions were then created to ensure that students watched these tutorial videos before the in-person class. We recorded videos from those scripts using the Camtasia video editing software. These tutorial videos were then uploaded to a module for the Canvas course shell for the class. Students then watched the tutorial videos and completed the pre-tests. Finally, the College of Pharmacy liaison librarian presented more advanced searching techniques during the in-person class. These concepts built on what was introduced in the five tutorial videos they viewed as pre-class work.

Conclusion: The primary outcomes from this project were the creation of tutorial videos to be used in various contexts. The videos are able to introduce search skills in scholarly databases for evidence-based medicine and can be applied cross-discipline. The scripts and pre-test questions were also saved so the tutorials could be updated if the need arises. Outcomes for the instruction itself were to enable the pharmacy liaison librarian to cover more advanced searching techniques in the in-person class.

Library Instruction Without Borders: Virtual Research Consultations

Practice Area: Information Services

Mariana Lapidus - Reference Coordinator, MCPHS University, Boston, Massachusetts

Background: Reference librarians at a small private university have been regularly offering individual research consultations for students, but these personalized meetings were never categorized or assessed as a separate type of service. With expanding the number of online programs and to better meet students’ information needs, in October 2019 the library has implemented an online Research Consultation request form for scheduling consultations with the librarians. At that time, students could request in-person, phone or online session. During the pandemic, the demand for virtual Zoom consultations has significantly increased, making it essential for librarians to provide effective scheduling and correct statistical data.

Description: The Research Consultations dataset was added to the Springshare LibAnswers, which serves as an online platform for collecting all types of reference transactions. Nevertheless, quantitative analysis completed by the end of the year showed that statistical data did not reflect the correct number of consultations: some librarians entered the sessions as regular reference questions in the Reference Analytics dataset; others categorized them under Instruction in the separate Access database. To eliminate these discrepancies, a concise table highlighting the differences between the three types of library services (Reference, Research Consultations and Instruction) was compiled and approved by the Library to ensure that the staff defines each reference transaction correctly, and statistics is consistent with the number of provided sessions. In addition to this table, the poster will highlight methods used to schedule consultations, important trends identified, and the library future goals.

Conclusion: Research consultations remain an important but often overlooked part of reference services offered by academic libraries. Students appreciate an opportunity to meet with a subject librarian face-to-face and get individualized assistance with finding information for their assignments or research. This type of information literacy instruction can be especially effective in helping students to define a research topic, improve literature search strategy, and identify the best resources. Such online software as Zoom allows students to watch librarians utilize online resources in real time and helps them to master the best practices of literature searching and evaluation.

Library Involvement in a New Medical School – The 1st Four Years

Practice Area: Information Services

Lisa A. Marks AHIP - Director, Library Services, Mayo Clinic Libraries-Arizona, Scottsdale, Arizona

Background: This institution’s medical school opened in 1972 on its original campus. Since then, the institution has grown to a multi-state health care system. It was decided to open a branch of the existing medical school, including a library, on another campus in a different state to create a national medical school. The first class was admitted and began classes in July 2017. This inaugural class will graduate this year, May 2021.

Description: This poster will describe librarian involvement and services provided to the medical students from creating a medical school library space to working with students in several ways including providing reference services, literature searching and research assistance, mentorship for selectives and instruction during the EBM block of medical school.

Conclusion: Outcomes are to provide a foundation for lifelong library use as students complete medical school and go on to their residencies, fellowships and careers.

Maintaining an unstaffed library: Adapting to Covid-19 and bringing lessons learned into a post-Covid world

Practice Area: Professionalism & Leadership

Erin RB Eldermire - Head, Veterinary Library, Cornell University, Ithaca, New York

Background: In March 2020, as the world adapted to sweeping lockdowns due to the onset of the Covid-19 pandemic, academic libraries were shuttered. Slowly, as we learned to cope with Covid-19, libraries reopened, though most did so with restricted access and services. This poster describes how, in August 2020, one small medical library re-opened its spaces and collections using an unstaffed model. Important lessons have been learned that can inform library operations after the threat of Covid-19 subsides.

Description: The Flower-Sprecher Veterinary Library supports the College of Veterinary Medicine at Cornell University, and serves a population of approximately 1500 faculty, staff, and students. As the Fall 2020 semester approached, access to the Veterinary Library was deemed an essential need for students, which houses multiple copies of textbooks that support student learning and provides ample study space. To ensure the safety of our skeleton crew of 3 library staff, we opened our doors using an unstaffed model. The library is accessible to

members of the college via card swipe from 8am - 2am, 7 days/week. Public computers and printers were removed but a scanner is available. Patrons can check out books by request using contactless pickup. Originally, basic services were carried out by staff during hours that the library was closed, but as vaccinations became prevalent, this practice dissolved. Basic services include checking out books, scanning materials, and reshelving items.

Conclusion: Between August 1, 2020 and January 1, 2021, over 8,000 users have entered the Veterinary Library (compared to 38,000 entries in the five months preceding the pandemic onset). Evidence that textbooks are being used in-house is often seen. To date, we know of no books or resources that have gone missing. Following the success of this model, we will experiment with a hybrid staffing model as the pandemic subsides, in which our library is staffed for busier times but unstaffed when traffic is low. If successful, this will contribute to significant savings for our limited budget. Statistics and insights shared in this poster can inform operating statuses of other libraries that serve similar populations.

Making Space for Wellness: Grant-funded Projects to Support Visitor Well-being in the Library

Practice Area: Information Services

Chloe Hough - Evening Circulation Supervisor, University of Florida Health Science Center Library, Gainesville, Florida

Ariel F. Pomputius AHIP - Health Sciences Liaison Librarian, University of Florida Health Science Center Libraries, Gainesville, FL

Background: Libraries can support the well-being of their visitors through wellness collections of resources and mindfulness programming, but can also offer wellness in their spaces. The project team of [institution] pursued multiple internal-grant funded initiatives to support visitor wellbeing. One phase of the program introduced desk treadmills, and the other introduced a free-standing cell phone charging locker, with assessments planned to determine how effective visitors feel these new features are at improving their wellbeing. The new equipment would allow for students studying for long hours an opportunity to increase physical activity, as well as take a break from distracting technology.

Description: These grant programs stemmed from the desire to support the physical and mental well-being of library visitors. Equipment was purchased and installed when the library reopened to visitors in its physical spaces. Research was conducted on the self-described wellness needs of library visitors which confirmed the importance of these initiatives, but data was also collected from users about the perceived effectiveness of the charging locker to reduce digital distraction and the underdesk treadmill to increase focus. In order to encourage use, the project team developed a Walking Challenge component to the treadmill assessment- a walking contest will be held between six health science colleges, with the winning college

receiving a trophy. Entering steps for the competition will serve as incentive to fill out the full survey associated with treadmill usage.

Conclusion: While data collection and analysis is currently on-going, the project team knows that there is interest in wellness support at the libraries based on previous surveys. The completed data analysis will help the libraries assess the perceived effectiveness of these programs and decide whether to move forward with acquiring more equipment to make library spaces more conducive to visitor well-being.

Managing relationships during a collection transformation: Lessons learned from a consortium withdrawal

Practice Area: Professionalism & Leadership

Rachel Helbing AHIP - Director of Library Services for the Health Sciences, University of Houston, University Libraries, Houston, Texas

Background: In 2019, our library withdrew from a long-standing consortial health sciences collection agreement. Per the agreement, most programs on our campus did not have access to the resources provided by the consortium, so we withdrew in order to direct those funds toward health sciences resources that would be available campus-wide. This decision resulted in some dissatisfaction with the library and a perceived loss of identity and status for the programs that did have access to the consortial collection. We needed to manage complex relationships through this change in order to regain library users' trust and satisfaction.

Description: We learned important lessons about change and relationship management while working with both library administration and library users through this transition. The process of withdrawing from a consortium requires advance planning, data analysis, and - most importantly - strong communication with library users. This poster will provide tips and best practices for collections change management, which are included in the Conclusion section of this abstract.

Conclusion: Provide relevant data in an easy-to-digest format to explain the decision. Do not assume that silence after the announcement of a change equals acceptance of the change. Develop allies among the library users. Have one-on-one conversations with stakeholders. Listen empathetically to the concerns of users and address them whenever possible. Make sure the library's message is consistent. Take responsibility for the decision and be clear that it is final. Focus on the positives and opportunities for growth without glossing over the negatives. Advocate for the equity of campus-wide access over the prestige of being part of a well-known consortium. Conduct proactive marketing to ensure users are aware of existing resources. Seek support from library and university administration as needed.

Mapping the Impacts of COVID-19 on the Work-Life Balance of Academic Librarians Who Are Parents of School-Aged Children

Practice Area: Professionalism & Leadership

Beth Auten AHIP - Health & Human Services Librarian, J. Murrey Atkins Library, Charlotte, North Carolina

Mary E. Edwards MLIS, EdD - Liaison Librarian, UF HSC Library, Gainesville, FL

Hope Y. Kelly - Online Learning Librarian, Virginia Commonwealth University Libraries, Mechanicsville, Virginia

Objective: Academic librarians at three public universities in the Southern United States who are parents of school-aged children have experienced changes in their work, parenting, and the balance between them during the lockdowns, quarantines, and closures necessitated by the current pandemic. Many librarians have been affected by these conditions, and there are variations in the support and guidance provided by institutions, and the impact of governmental policies.

Methods: To begin, we selected a purposive sample of 11 public, SACS-accredited universities from the 11 states in the Southeastern U.S. We compiled university policies related to the Federal Families First Coronavirus Response Act (FFCRA) and other institutional support made available to librarians. Since leave policies have been updated since FFCRA provisions ended on December 31, 2020, we compared archived policies with policies in effect in 2021. We reviewed the text of these policies to determine how FFCRA was applied, and whether any other provisions were made for working parents after FFCRA ended. In order to see how these policies were applied in practice, we surveyed librarians at the selected institutions.

Results: Our results from the policy review are presented as an interactive table with links to available policy sites. For each institution, it shows whether a guidance page on FFCRA was available in 2020, whether there was an extension of leave after the federal act expired, information on leave specifically related to school closures; and whether policies applied to parents who chose to keep children out of school.
As of May 2021, we have received survey responses from seven states. Themes were drawn from the open-ended answers we have received so far, and several relevant quotes have been broken out to authentically express the experiences of the participants.

Discussion: Data collection is ongoing for this study, but based on the responses we have received at this time, there have been positive and negative impacts resulting from the leave policies, and remote and flexible work arrangements, implemented in response to the pandemic.
Many participants observed a breakdown of boundaries between work and family time, and

frequent interruptions to their work. However, most participants were not concerned that promotion or tenure would be impacted, due to policies implemented by their institutions. Participants also indicated that flexible leave olicies are well received and made it possible for them to learn new skills and remain productive.

Maximize use of library electronic resources to help students achieve better board examination scores through LibGuides and collaboration with a pharmacy educational specialist

Practice Area: Education

Wendy Wu - Information Services Librarian, Shiffman Medical Library, Detroit, Michigan

Karen Gessler - Pharmacy Education Specialist, Michigan

Background: The library subscribes to many e-textbooks and Q-bank tools to facilitate pharmacy students’ preparation of North American Pharmacist Licensure Examination (NAPLEX). Because these resources are included in various subscription packages, making them discoverable and easily accessible are important to make best use of library resources. This poster will discuss how to promote NAPLEX resources using LibGuides and deliver the specially designed quiz assignments to the students at the point of preparation and address the collaboration between a librarian and pharmacy educational specialist.

Description: Resources the library subscribes for NAPLEX preparation were selected, organized, and displayed based on faculty recommendation, students' feedback, and LibGuides best practices tips. Students can choose online Q-bank questions for practice by topics, pharmacy education competencies, disorders, etc. from this one-stop-shopping NAPLEX preparation LibGuides page. To further promote the use of library resources and increase students’ examination scores, the educational specialist identified a variety of pharmacy education competencies under NAPLEX Blueprint and created assignments that would align with these competencies for P4 students. The assignment, including 10-15 quiz questions selected from NAPLEX resources, was distributed to a student group prior to board examination. Each week students would receive and conduct a new competency-based assignment, review correct answers with rationale and get feedback on their performance.

Conclusion: The team collaboration plays a key role to ensure fast access to core NAPLEX resources and systematic delivery of Q-bank questions to students at the point of preparation, and thus advanced the usage of library resources and led to greater student success.

Medical Librarians and Environmental Justice: Creating an Environmental Health Literacy Program on Air Quality in the Historic Environmental Justice Community of Camden, New Jersey

Practice Area: Global Health & Health Equity

Benjamin Saracco - Research and Digital Services Librarian, Cooper Medical School of Rowan University Library, Camden, New Jersey

Rachel King - Cooper Medical School of Rowan University

Kathryn Cruz - cfet.org

Elizabeth Cerceo - Cooper Medical School of Rowan University

Mohammed Jawaad Hussain - Pediatrician, Cooper University Health Care, Camden, New Jersey

Jennifer Kitson - Cooper Medical School of Rowan University

Zachary Christman - Cooper Medical School of Rowan University

Background: This poster presentation will cover a recent NNLM-funded grant project entitled "Clearing the Air: Educating the Camden Community about Air Quality and Respiratory Health". Camden, NJ has long fit the stereotype of the struggling post-industrial American city. Residents live with many environmental injustices not experienced by wealthier areas and the city is home to numerous industrial polluters. The purpose of this project is to provide the residents of Camden with environmental health information that will empower them with information on the links between the air quality in their community and their respiratory health.

Description: The Cooper Medical School of Rowan University (CMSRU) Library planned a community outreach program that informed members of the public about the impact that air quality has on health in general, and its specific role in triggering asthma and other respiratory diseases. This health information will be presented in easy-to-understand, user-friendly ways that will make it accessible to all. The centerpiece of the project will be a free, public website that showcases National Library of Medicine information resources such as MedlinePlus's respiratory health topics. Additionally, there will be a series of expert-led environmental-health community events that can, in order to best meet the needs of attendees, will be delivered as recorded teleconferences. A select number of attendees will also be provided handheld particulate matter monitors with citizen-science take-home worksheets.

Conclusion: Intended outcome of this project include: - Provide background on the unique environmental health problems affecting Camden residents, specifically those involving air pollution. - Promote the use of high-quality health resources, such as MedlinePlus and other high quality, vetted sources of environmental health information. - Learn a new baseline for what a healthy local environment looks like. - Know where to go to report environmental

hazards of all types in the city. - Learn of opportunities to become more civically engaged in the environmental health of their neighborhoods. -Learn what particulate matter is and what harms it can cause. - Learn to interpret particulate matter readings. - Learn about the effects of industrial odor pollution and olfactometry.

The Medical Librarian’s Mentorship in Research Scholarship for Medical Students in Undergraduate Medical Education

Practice Area: Innovation & Research Practice

Misa Mi AHIP - Professor, medical librarian, Oakland University William Beaumont School of Medicine |Medical Library, Rochester, Michigan

Background: Health science librarians are integral to educational programs at their home institution. There is abundant literature on information literacy instruction. Existing mentorship programs are most tailored to librarians’ professional development, instituted by individual libraries or professional organization. There is a paucity of literature on librarians’ mentorship for students. The aim of the poster is to illustrate three types of librarian mentor-mentee relationships, introduce a process and practice for mentorship, demonstrate outcomes, and share tips and strategies for the mentor role as a librarian to promote the contribution of a medical library in scholarship of discovery.

Description: The OUWB Medical School annually recruit mentors for a required student research Capstone research project program and the Summer Internship Program. The Capstone research project is a mandatory four-year program for all medical students. The Summer Internship Program is for medical students following the completion of their first year of medical school. The internship opportunities are meant to foster both personal and professional development. Apart from the two programs, there are medical students who are driven and embark on additional research projects to pursue their interests in a specialty of their future choice. The medical librarian’s mentorship meets students’ needs in conducting research projects out of the scope of traditional bench research or clinical research and introduces them to different research methodologies such as systematic review, rapid review, evaluation studies in the fields of consumer health informatics and medical education.

Conclusion: Through the librarian’s mentorship, students learn research skills, information seeking/management skills, develop a better understanding of research methodologies, and cultivates a deep appreciation for the role, skill sets, and knowledge of the librarian. Furthermore, students get to involve in scholarly activities and disseminate their project outcomes at professional conferences or via submissions for peer-reviewed journals. The librarian’s mentorship provides a unique learning opportunity for students to develop professionally in areas of research, library skills, lifelong and self-directed learning. The outcomes resulted from the mentor-mentee relationships enhance students’ qualification for their future residency program in a specialty of their choice. Moreover, the librarian’s mentoring

of medical students promotes the librarian’s important role and contributions in developing future physician investigators.

Medical Student Question Formulation Skills Development: A Randomized Controlled Trial

Practice Area: Education

Jonathan D. Eldredge MLS, PhD - Professor, HSLIC RECIS, Albuquerque, NM

Melissa Schiff MD, MPH - Professor, Internal Medicine, Albuquerque, New Mexico

Jens Langsjoen MD, MSc - Hospitalist, Internal Medicine, Albuquerque, New Mexico

Objectives: Eldredge et al. reported at MLA 2020 on their quasi-experiment involving a new FAC (Focus, Amplify, Compose) rubric for assessing medical students’ Evidence Based Practice (EBP) question formulation performance. This new rubric, when linked to complementary hands-on training, improved student scores. This study builds on the previous study by measuring whether the rubric alone, or when combined with a 25-minute intervention, will be more effective in teaching second-year medical students on how to formulate effective EBP questions.

Methods: Randomized Controlled Trial. 80 second-year medical students were randomized into either an intervention group or a control group prior to the beginning of the block. The authors administered a pre-test to all students that presented them with a clinical vignette and asked them to formulate an EBP question. Following the pre-test, all students were shown the question formulation rubric and it was briefly explained. Students in the intervention group were taught how to formulate EBP questions using the rubric followed by another 30 minutes of EBP search training. Students in the control group only received the EBP search training in their small group labs. All 80 students took the post-test in which they formulated a question in response to a clinical vignette. Statistical analysis will involve a two-sample paired t-test to test between-group differences.

Results: The authors collected their pre-test and post-test data for the intervention and the control groups during December 2020. The authors will report their findings at the MLA 2021 Annual Meeting.

Conclusions: The authors will offer their conclusions during the MLA 2021 Annual Meeting.

Multiple Probabilistic Automated Publication Type Taggers to Index Biomedical Articles and Support Evidence Synthesis

Practice Area: Information Services

Neil R. Smalheiser MD-PhD - Professor in Psychiatry, Psychiatry, Oak Park, Illinois

Jodi A. Schneider - Assistant Professor, School of Information Sciences, University of Illinois at Urbana-Champaign, Champaign, Illinois

Aaron M. Cohen - Professor, Oregon Health & Science University, Beaverton, Oregon

Objectives: Information specialists have developed various search hedges and filters in order to retrieve studies with different publication types and study designs (e.g., randomized controlled trials, cohort studies, case control studies, etc.) for information retrieval. This is particularly important for evidence synthesis which requires comprehensive retrieval. Manual indexing is neither rapid enough, accurate enough, nor consistent enough across bibliographical databases. Therefore, we trained a set of automated machine learning based probabilistic taggers for 50 publication types and study designs. Probabilistic scores are more flexible than binary yes/no tags, and have thresholds that can be tailored for specific use cases.

Methods: Training data was based on human-related PubMed article records from 1987-2014. Title and MEDLINE/PubMed information such as MeSH terms and MeSH Publication Types were used to create labeled training sets. Predictive features included title and abstract bigrams, journal similarity, and various measures of pairwise similarity across two articles (e.g., important word scores, implicit term similarity scores, and Paragraph2Topic vectors). Taggers were trained using liblinear SVM, recalibrated on 2015 PubMed data, and then tested on manually assigned PubMed publication type data from the years 2016 and 2019. We measured performance with the area under the curve of the Receiver Operating Characteristic (AUC-ROC), which measures the tradeoff between maximizing true positives and minimizing false positives. We also carefully reviewed manually several of the most important publication types for systematic review, including Cohort Studies, Cross-Over Studies, Cross-Sectional Studies, and Randomized Controlled Trials.

Results: Overall the AUC-ROC performance is very high: The best performing tagger was TWIN_STUDY with an AUC of 0.996, and most of the taggers have AUC values above 0.9. For Cohort Studies, Cross-Over Studies, Cross-Sectional Studies, and Randomized Controlled Trials, when articles lacked NLM indexing but were strongly predicted as such by our model, manual review verified that the model was correct in the majority of cases.

Conclusions: We have created a dataset for all PubMed articles having abstracts of their probabilistic prediction scores for 50 Publication Types and study designs. These will be made publicly available for download at

http://arrowsmith.psych.uic.edu/evidence_based_medicine/index.html. These have also been incorporated into the Anne O’Tate tool http://arrowsmith.psych.uic.edu/cgi-bin/arrowsmith_uic/AnneOTate.cgi: When a user makes a PubMed query, the list of retrieved articles are displayed according to both NLM-indexed Publication Types as well as our predicted publication types and study designs. Publication type taggers may be of particular interest for systematic review teams and in resource-constrained settings such as rapid reviews.

No MeSH, No Filters, and No Librarians: Oh My! An Examination of the Search Strategies Used in 100 Published Systematic Reviews

Practice Area: Information Services

Jennifer C. Westrick AHIP - Library Research Information Specialist, Library of Rush University Medical Center, Westchester, Illinois

Patricia Chavez - Library Research Information Specialist, Library of Rush University Medical Center, Chicago, Illinois

Objectives: The increased popularity of systematic reviews has resulted in a wide variety of methods used to construct the literature search for these publications. Our project examines the components of 100 literature search strategies used in published systematic reviews to determine if specific key elements were included in the search strategy. To our knowledge, no research exists that gathers data on this specific topic.

Methods: We identified 100 citations that were indexed in PubMed and included the following criteria: the words “diabetes” and “systematic review” were in the title, and they had been tagged as a systematic review by MEDLINE indexers. Diabetes was chosen to allow a wide variety of clinical areas, including pharmaceutical, therapeutic and behavioral. We limited results to those published in 2019 and selected the first 100 results, sorted by relevance. We analyzed these reviews to determine how the literature search string was constructed, including whether MeSH terms were used, if the results were limited to the title/abstract fields, if guidelines or checklists such as PRISMA (Preferred Reporting Items for Systematic Reviews and Meta-Analyses) or MOOSE (Meta-analyses Of Observational Studies in Epidemiology) were purported to have been followed, if a reproducible search strategy was included, the average number of databases used, and whether a librarian was a co-author or acknowledged in some other way.

Results: 55% of these reviews included a reproducible search string (many provided just keywords, and some included no information about the literature search strategy). 48% of researchers used MeSH terms, and 23% used field codes to filter by title/abstract or text word. The average number of databases searched was 3.8, and 85% of reviews stated that they followed PRISMA or MOOSE guidelines or checklists. Only 11% of the reviews acknowledged

librarian involvement. Of the reviews associated with a librarian, 73% used MeSH terms and 56% used field codes.

Conclusions: Our analysis of the components of 100 literature search strategies used in published, indexed systematic reviews shows that consistency is lacking. Our research does not attempt to recommend the elements to include in a search strategy, as some elements are appropriate for one review but may not be appropriate for others. Rather, using visuals such as charts and graphs, our poster illustrates how often the literature search strategies of 100 published systematic reviews used MeSH terms, field code searching, and other common key components of a literature search strategy.

'No One Measure to Rule Them All:' Teaching JIF Alternatives

Practice Area: Information Services

Jess N. Newman MSIS - Research Data & Scholarly Communications Lead/Assistant Professor, University of Tennessee Health Science Center, Memphis, TN

Background: The presenter developed a new workshop titled "Research Impact Demystified" to educate both faculty and students on research impact metrics. Objectives include identifying examples, purpose, and limitations of both common and alternative metrics and understanding the difference between journal, article, and author level metrics. The goal of the workshop is to help researchers locate and compile the correct metrics for their purpose and to increase their impact by improving the communication and reach of their work through the use of scholarly profiles and author identifiers.

Description: The impetus for this new workshop was non-renewal of the library's subscription to Web of Science and the Journal Citation Reports, upon which many faculty are reliant for the Journal Impact Factor in particular. The workshop and related research guide are organized around three bibliometric analysis levels: article, author, and journal. By building from raw citation counts at the article level to more complex calculations such as journal rankings learners may easily follow the logic of many impact metrics. In addition to biweekly webinar sessions, in the first month of advertising and offering the workshop three departments have requested presentations for both graduate student and faculty audiences. Attendees to the webinar are asked to complete a post-session survey to measure general feedback. Future course-integrated sessions will be evaluated on the stated learning objectives through a pre- and post-survey.

Conclusion: The workshop will be evaluated on the stated learning objectives: identifying examples, purpose, and limitations of both common and alternative metrics; sources for research impact metrics; understanding journal vs article vs author level metrics; choosing and compiling the correct metrics for the purpose; and tracking and enhancing research visibility with online profiles and scholarly identifiers. Additionally, the author anticipates that faculty use

of Scopus source metrics and scholarly profiles such as ORCID will increase and will measure this usage where possible in order to target future outreach and service efforts.

Online Academic Days: Celebrating Student and Resident Scholarly Activity During a Pandemic

Practice Area: Information Management

Leanna Stager AHIP - Scholarly Publications Librarian, Zucker School of Medicine at Hofstra/Northwell, Hempstead, New York

Stacy F. Posillico MLS, JD - Senior Librarian, Northwell Health Libraries, Hempstead, NY

Background: For many years, our School of Medicine and Health System has held Academic Days. These highly attended in-person events took place at the School of Medicine and hospitals throughout the system, showcasing the scholarly work of our many graduate and undergraduate academic trainee programs. Medical students and residents are invited to submit abstracts and posters of their current research for display, and often the best contributions are recognized and a keynote speaker is invited. Due to the response to COVID-19, many events at the School of Medicine and Health System, such as Academic Days, were required to be fully online.

Description: Not wanting to miss the opportunity to celebrate our students’ and trainees’ successes, the School of Medicine Library worked with the Academic Competition and Research Day Planning committee at the system level and one hospital site to leverage the existing Bepress repository, Academic Works, to display the students’ and trainees’ scholarly work online. As a complement to the event program, Librarians created pages for the events that matched the overall style of the repository but allowed for custom banners, organization, and metadata requirements for each event depending on their needs. Full-text posters and abstracts were digitized and added to each program’s event site. Librarians restricted full text access to the scholarly work by IP address to ensure only affiliated users could download them while also creating a publicly available metadata record to ensure findability.

Conclusion: Both online events were a resounding success and planning is already underway for the Health System’s 2021 Academic Competition. Creating these event pages as a part of Academic Works has increased awareness of the repository in general, driving traffic to the site following the high numbers of views made around the time of the online events for each Academic Day. With the 2020 events available as an example, other graduate medical education programs have expressed interest in developing similar pages to highlight their Residents’ research. The Library anticipates the creation of these online showcases will continue even when in-person attendance is once again permitted.

Orienting Medical Students to the Medical Library – A First Critical Step in Developing Students into Lifelong and Self-Directed Learners

Practice Area: Education

Misa Mi AHIP - Professor, medical librarian, Oakland University William Beaumont School of Medicine |Medical Library, Rochester, Michigan

Stephanie M. Swanberg AHIP - User Services Librarian, Moustakas Johnson Library, Michigan School of Psychology, Farmington Hills, Michigan

Keith Engwall MSLIS, AHIP - Medical Librarian, OUWB School of Medicine Library, Oakland University

Nancy Bulgarelli - Director, Medical Library, Medical Library, Oakland University, Rochester, Michigan

Background: The pandemic has transformed the delivery of library services, prompting immediate needs to change existing modalities for reaching out to students, faculty, and staff. Library orientation is essential for promoting and teaching about library services, resources, and instructional programs. How do librarians provide the best orientation experience for students and set the initial important stage for students to recognize the value of the library and to utilize library services and resources to their advantage? This presentation will describe how one medical library innovated its approach to providing a successful library orientation for M1 medical students during the pandemic.

Description: The OUWB School of Medicine provides a mandatory one-week orientation for new medical students during the first week of each August. The medical library orientation integrated into the medical school orientation serves to orient students to their new learning environment. The Library launched its virtual orientation planning phase at the cusp of the pandemic. As the school orientation became packed with a wide array of sessions and events, the entire library orientation time slot was cut to 20 minutes of virtual time with 125 medical students using WebEx. It became unfeasible to provide an effective learning experience for all students merely through a synchronous virtual library orientation. The final redesigned orientation included virtual space and website tours using Adobe, a pre-recorded introduction video to the library information mastery instructional theme, small group, 20-minute synchronous sessions where students met their “personal librarians” for a brief overview of services and a Q&A, and instructional help sheets outlining how to locate learning resources.

Conclusion: The library pivoted to designing a blended library orientation and successfully executed the plan that resulted in a seamless, effective, and efficient virtual orientation. It optimized medical students’ learning experience and ushered them on their way to becoming lifelong and self-directed learners.

Pivoting Clinical Librarian Integration in the Wake of The Pandemic

Practice Area: Clinical Support

Emily Shohfi AHIP - Clinical Medical Librarian - Assistant Professor of Medicine, Darnall Medical Library, Walter Reed National Military Medical Center, College Park, Maryland

Michele L. Mason-Coles - Clinical Librarian - Assistant Professor of Pediatrics, Walter Reed NM Medical Center - Darnall Medical Library, Bethesda, Maryland

Background: Prior to COVID-19, our library had multiple librarians actively rounding with clinical teams to assist with evidence-based clinical decision-making in patient care and involvement with weekly in-person didactics. Our library continues to serve health care providers clinically and through research support. An ongoing discussion at our library and others nearby centers on reintegration of in-person services, dependent on safety and support from programs. We outline how we have pivoted clinical librarian services in the wake of the pandemic, and our best tips for new and returning clinical librarians in a mid- and post-pandemic world.

Description: Since transitioning to virtual presence, we continue to maintain participation with clinical teams in a variety of formats to increase accessibility and attendance. Parallel to this, we have had discussions with other medical librarians regarding best practices in clinical services and how to become involved while serving in a virtual capacity. We suggest taking an active participatory role in didactics, using these as opportunities for effective teaching and “presence.” We also find checking in with rounding teams weekly by email or phone provides an opportunity to support residents, fellows, and attendings for challenging clinical questions. We anticipate that after the pandemic, our services will continue to expand and will incorporate new ways of reaching out and meeting the needs of our patrons. We will address responsible use of PPE, changes to rounding format and frequency, and changes to instruction methods.

Conclusion: Clinical rounding services will inevitably look different from pre-pandemic years after the COVID-19 pandemic subsides. From a mid- and post-pandemic standpoint, we will address questions ways for librarians to continue to serve patrons in clinical settings, from continuing existing services in new formats to expansion into new service territories. We will discuss ways to work with our hospital’s departments to minimize threats of COVID-19 exposure to Clinical Librarians upon reintegration, and anticipated changes to rounding format, frequency, and procedures. We will also discuss ways to re-establish integration or create personal rapport for clinicians with whom we have only had virtual or intermittent communication, as this is a vital way for our library to stay current and relevant.

Pod Save You: Assisting the Transition to Audio-Based Asynchronous Learning

Practice Area: Education

Brandon Patterson - Technology Engagement Librarian, Eccles Health Sciences Library, Salt Lake City, Utah

Bryan Hull - Program Manager, Digital Publishing, Spencer S. Eccles Health Sciences Library, Salt Lake City, Utah

Background: In 2018, an academic health sciences library in the mountain west developed a multimedia studio for students, faculty, and academic staff. Educational projects needing video, audio, and lecture capture could utilize a one-button studio for recording video sessions, microphones for audio, and various screen capture software for lectures. Since the pandemic, this service has seen rapid growth due to academic lectures going exclusively online. This year we launched a dedicated Podcasting Suite to accommodate the increase in students and faculty needing to record lectures or podcasts for others in the medical profession.

Description: This poster will outline the process of creating the podcasting suite and provide equipment rosters and methods other libraries may consider for establishing their own podcast studio. We’ll also cover the administration of the studio including: handling reservations, COVID-19 guidelines, use assessment, and integration into the library’s strategic directions. Finally, we’ll cover creating instructables and quick start guides that assist patrons in accomplishing their podcast creations.

Conclusion: Podcasts created in the space range from topics about teaching strategies in medicine to diagnoses and treatments of skin disorders. The Podcasting Suite has proven to be another way library’s can provide valuable services for asynchronous learning and student projects. We’ve heard from students, staff and faculty that they appreciate the ease of the service and the support behind it. A feedback loop was developed so we can constantly adapt the space to meet the needs of our users where we respond to requests every quarter.

Predatory Publishers: What to Know and How to Avoid Them

Practice Area: Information Services

Lara Lasner-Frater - Electronic Resources Librarian, Sirota Library/Touro College of Osteopathic Medicine/College of Pharmacy, New York, New York

Susan Lesser - Evening Librarian, Sirota Library, New York, New York

Background: A predatory publisher is an opportunistic publishing venue that exploits the academic need to publish but offers little reward for those using their services. Predatory publishers exploit the emerging acceptance of open-access academic journals by making it attractive to publish.The ultimate goal of these publishers is to make a profit -not to promote scholarly research. They have no concern for the quality of work published and typically lack the peer-review process that legitimate journals provide.

Description: This poster shows the Definition of predatory publishing (including journals and conferences). Why it is important for researchers to be aware of them, Examples of predatory publishing, How to spot a predatory publisher. How to tell the different between a legitimate Open Access Journal and a predatory one.

Conclusion: Predatory publishing is especially damaging for biomedical and pharmaceutical fields due to a lack of comprehensive peer review. It helps create a rise in pseudo science and perpetuates bad research. It's important for medical librarians to remain vigilant that researchers avoid predatory publishers.

Preprints and COVID-19: Analysis of 2020 Pandemic-related Works from a Single Institution

Practice Area: Information Services

Konstantina Matsoukas - Research Informationist III, Medical Library, Memorial Sloan Kettering Cancer Center, New York, New York

Jeanine L. McSweeney MLIS - Associate Librarian, Scholarly Communications, Memorial Sloan Kettering Cancer Center Library, New York, New York

Donna S. Gibson - Director of Library Services, Memorial Sloan Kettering Library, New York, New York

Robin O’Hanlon - Associate Librarian, User Services, Memorial Sloan Kettering Library, Astoria, New York

Marina Chilov - Research Informationist II, Memorial Sloan Kettering Library, Brooklyn, New York

Johanna Goldberg MSLIS - Research Informationist II, Memorial Sloan Kettering Library, Bronx, New York

Lindsay M. Boyce - Research Informationist, Memorial Sloan Kettering Cancer Center Library, New York, New York

Objectives: The coronavirus pandemic accelerated the acceptance of preprints by the clinical research community, with the need to rapidly share new research findings even leading to the inclusion of COVID preprints in PubMed/PMC. This occurred even though preprints are not properly vetted, final published versions of works, but rather, preliminary scientific research reports that have not yet been evaluated and certified by peer review. To enhance our understanding of health science preprint server posting adoption, we characterized/analyzed the 2020 research manuscripts related to COVID-19 posted to preprint servers by researchers at our institution.

Methods: Thirty-three preprints meeting our inclusion criteria were identified via targeted searching. The preprint server, PubMed/PMC, and journal publisher records were consulted to verify: manuscript posting, revision, and publication dates; author and affiliation information; linkage to the final published versions on the preprint and PubMed/PMC records. Altmetrics and Dimensions, both products of Digital Science, were used to capture online attention and any citations received by the preprints and their subsequent peer-reviewed articles.

Results: Of the 33 preprints, two were duplicates: one a revised preprint version with title change, the second a submission of the same preprint to two different servers. Eight different servers were used overall, with 51% posted to medRxiv and 24% to bioRxiv. PubMed indexed 52% of the preprints, with 19/31 preprints eventually being published in a peer-reviewed journal. 11/19 published papers were linked to from their preprint server record and 79% were published in NPG, Elsevier, Springer, or Wiley journals. Of the 31 unique preprints, 90% included co-authors from other institutions, with 14/31 involving international collaborators. Of the 19 preprints published in journals, posting to a server accelerated the release of the manuscript to the public record by a mean of 102.3 days. Preprints indexed in PubMed (16/31) garnered a mean of 2.8 times more citations than preprints not indexed in PubMed (15/31).

Conclusions: During COVID times, it was assumed that the motivation for posting to a preprint server was closely tied to a desire to get information out as widely and as quickly as possible. Our findings suggest that posting a manuscript as a preprint was generally worth the effort in terms of accelerating research sharing and visibility. Future research exploring whether authors who utilized preprint servers during the pandemic will continue to do so post-COVID will be interesting for establishing if this experience will have changed researcher behavior with regards to preprints going forward.

Providing Document Delivery/Interlibrary Loan services from within a Pandemic: Filling in the Gaps?

Practice Area: Information Services

Vida M. Vaughn - Director, Kornhauser Health Sciences Library, University of Louisville, Louisville, Kentucky

Kathy L. Rogers - Interlibrary Loan Manager, Kornhauser Health Sciences Library, University of Louisville, Louisville, Kentucky

Felix Garza Staff - Head, Public Access Services, Kornhauser Health Sciences Library, University of Louisville, Louisville, Kentucky

James Adler - Library Asst., Kornhauser Health Sciences Library, University of Louisville, Louisville, Kentucky

Background: On March 11, 2020 the World Health Organization (WHO) characterized the alarming spread and severity of the coronavirus disease 2019 (COVID-19) as a pandemic. Responding to university directives, as well as professional association recommendations, many academic libraries not only suspended in-person services but also barred access to physical collections for Inter-library loan purposes. Such decisions had a significant impact on interlibrary loan (ILL) operations around the world. This poster describes how University of Louisville's Kornhauser Health Sciences Library (KHSL) not only maintained a continuity of ILL services but also expanded document delivery services worldwide by fulfilling requests for copies of items within the print collection.

Description: Upon approval of the KHSL Director, the Manager of ILL services worked remotely while a staff member came to the library two or three times a week to download print materials requests from the system. The staff member pulled and scanned the requested items from the physical collection. The items included journal articles and book chapters. Pdf copies of the scanned items were delivered electronically to the patron. Usage statistics were kept in order to measure the impact of the new delivery model on overall ILL outcomes. The restriction on sharing actual print items through the postal service remained in place. The risk of the staff member being exposed to the virus was minimized by the implementation of restricted access to the library and the surrounding buildings.

Conclusion: From 2019 to 2020, lending requests increased by nearly 1,000 during the time periods of March 16 - July 2. Many of the lending requests originated from libraries KHSL had not served in the past; including libraries from Denmark, Sweden, Holland, Great Britain, Hungary, Australia, Canada, Japan, South Korea. There was a marked increase in the number of requests from libraries within the United States. The expansion of document delivery services resulted in a significant increase in ILL revenue generated from exchanges with non-reciprocal libraries. KHSL administration noted a significant increase in ILL lending receipts.

Rapid Response: Librarian Integration Into An Expedited Pandemic Medicine Elective

Practice Area: Information Services

Gail Kouame MLIS - Assistant Director for Research & Education Services, Robert B. Greenblatt, M.D. Library, Augusta, GA

Jacob Gallay - Clinical Information Librarian, Robert B. Greenblatt, MD Library, Augusta, Georgia

Background: Describing how two librarians contributed to a new graduate medical school elective course developed dynamically in the midst of the COVID-19 pandemic.

Description: Shortly after the COVID-19 outbreak, the Assistant Director for Research & Education Services attended a clerkship curriculum committee meeting for the medical college where a four-week Pandemic Medicine elective was proposed. Clerkship rotations were canceled due to the pandemic, so the elective would provide opportunities for graduate medical students to participate in service learning and contribute to pandemic response efforts. The leaders and faculty members who proposed the elective suggested five areas of focus: information services; mental health and wellness; PPE taskforce; supporting medical education; and telemedicine. Immediately after the meeting the librarian contacted those organizing the elective and offered to contribute, particularly to the information services component. Both the Assistant Director for Research & Education Services and the Clinical Information Librarian were invited to serve as faculty advisors for the students leading the information services aspects of the elective.

Conclusion: The librarians attended weekly planning meetings with the information services group leaders, compiled lists of trustworthy resources for the students to consult, and performed literature searches. Within the information services area, the students chose to create relevant infographics in multiple languages, produce “mythbusters” information about COVID-19, develop a knowledge base from the literature using Zotero, and post a portal website. The website houses all the student-created content, along with local and national statistics about COVID-19. The website received recognition in the local press and university communication channels. Librarians were successfully integrated into a medical school elective course developed in response to the COVID-19 outbreak.

Repurposing Medical Library Standards: Effects of Covid-19

Practice Area: Global Health & Health Equity

Sangeeta Narang - Librarian Selection Grade, Branch Library, CRHSP, AIIMS, New Delhi, Delhi, India

Objectives The year 2020 has been an exceptional year for the mankind. Medical librarians are experiencing several health information adversities during this rapidly evolving phase of Covid-19 pandemic such as infodemic, proliferation of misinformation, disinformation, retraction of published papers and mushrooming predatory journals. In such situations, the medical librarian's role as a purveyor of authentic information to diverse communities, and assisting health professionals in synthesising evidence from the previously published studies have become intense. To understand extent and coverage given to these aspects of health information in medical libraries , we studied the library standards designed by medical library associations and related research papers published in past two decades.

Methods Over a period of six months (June 2020 to December 2020) author did several searches on PubMed and Google Scholar to find research articles on the standards of medical libraries. The search was also conducted on Google search engine for medical library association and the standards were combined to get the desired results. Only those professional medical library standards and research papers published since 2000 from advanced nations were finally considered for this study.

Results Considering increasing information disruptions taking place in the health ecosystem, these is need to repurpose existing medical library standards by the respective associations in all countries. Seeing the deficiencies in medical library standards in the low and middle income countries including India, author proposes set of standards to develop medical libraries in all these regions. These are based on available standards from advanced nations, also incorporating the latest advancements in public health information environment.

Conclusions Revision of medical library standards and their adoption in low and middle income countries including India will fortify medical librarianship education and enhance the existing vision of medical libraries in academic health institutions and hospitals across the regions and countries.

Research Data Management Librarian Academy (RDMLA): New Units, Partnerships, and Growing Impact

Practice Area: Education

Ashley Thomas - RDMLA Librarian, Countway Library of Medicine, Harvard Medical School, Boston, Massachusetts

Zhan Hu - Doctoral Student, School of Library and Information Science, Boston, Massachusetts

Rong Tang - Professor, School of Library and Information Science, Boston, Massachusetts

Background: Launched in October 2019, the Research Data Management Librarian Academy (RDMLA) meets librarians’ RDM training needs. Collaboratively developed by librarians, LIS faculty, and industry professionals, RDMLA directly addresses the learning gaps in RDM training discovered in a 2018 needs assessment survey and environmental scan. Practicing librarians responded to questions regarding their RDM training needs, challenges offering RDM services, and essential RDM skills needed for professional success. The findings provided insights into RDM practices, the state of RDM training, and exposed missing areas, key among them, a holistic, accessible, self-paced, and expandable curriculum.

Description: The RDMLA curriculum framework consists of eleven units addressing expressed RDM needs while avoiding redundancy in current training offerings. The first eight units were released in October 2019 and an additional three units were made available in late 2020. The RDMLA continues to grow and provide new content through new partnerships. RDMLA has partnered with Simmons University School of Library and Information Science to offer course CE credits. Partner institution National Taiwan University is nearing the completion of a traditional Chinese language version that will be available on Canvas Network in September/October 2021, after quality control and accessibility reviews. Additionally, the RDMLA will begin introducing mini-modules - brief video lectures with open-access readings - that discuss trending topics in RDM. This upcoming product has fostered new partnerships with the National Institutes of Health and Indiana University Purdue University Indianapolis. RDMLA’s impact is global. It is featured on the NNLM’s online courses and tutorials and ALISE COVID-19 LIS resources, as well as a selected course for the MLA’s Data Service Specialization. University libraries feature RDMLA as an important resource for RDM (e.g., Northwestern University, Carnegie Mellon University, Florida State University, University of Virginia, Victoria University of Wellington, University of Manitoba, among others).

Conclusion: RDMLA currently has more than 5,000 registered learners from 158 countries. The RDMLA team is committed to continuously providing comprehensive RDM training with new unit development and translation partnerships. Furthermore, RDMLA is more than just another online course; it is being incorporated in LIS courses, used as a professional resource, and is part of specialization or continuing education. Through the RDMLA, we seek to build a

global community of practice of RDM and further advance RDM practice in libraries and beyond.

Researchers’ experience and attitudes toward predatory journals and research productivity in Taipei Medical University, Taiwan

Practice Area: Education

Li-Chuan Huang - Librarian, Taipei Medical University Library, Taipei, Taiwan (Republic of China)

Ya-Fang Hsiao - Librarian, Taipei Medical University Library/Division of Knowledge Services

Shu-Yuan Siao - Head, Division of Knowledge Services, Taipei Medical University Library, Taipei, Taiwan (Republic of China)

Tzu-heng Chiu - Director, Taipei Medical University Library, Taipei

Hsiao-Fen Yu - Librarian, Taipei Medical University Library, Taipei

Chi-Ju Chiu - Librarian, Knowledge Services Sec., Taipei Medical University Library, Taiwan

Yu-Ying Lee - Librarian, Division of Knowledge Service, Taipei, Taiwan (Republic of China)

Objectives: The purpose of this study is to investigate the submission status as well as the problems faced by the Taipei Medical University (TMU) researchers, from 11 colleges, center of General Education, 11 university-level research centers, as well as the 3 affiliated hospitals.

Methods: This study was based on a questionnaire survey sent to TMU faculty and researchers for a 2-week period via email. Major contents of the questionnaire related to the following 3 questions: (a) the experience of publishing in predatory journals; (b) with regard to predatory journals, respondents expect what kind of support from the library and TMU; (c) the perspective and suggestions for changes in the Impact Factor(IF), which exists in the year of submission, but was suppressed/dropped by SCIE/ SSCI JCR or the value dropped dramatically when the year they need to calculate the quantitative research productivity.1,286 questionnaires were sent and 114 valid questionnaires were returned, resulting in a response rate of 8.9%.

Results: The main findings were: (a) All of the respondents have the experience to be invited by an unfamiliar journal, and approximately 80% of them have ever been invited as a reviewer. Furthermore, over a half of them have ever been invited as an editor; (b) 16% of the respondents have accidentally published articles in a predatory journal; (c) As to issues related to predatory journals, 60% of the respondents expect the library to provide blacklist and whitelist, as well as promotion events and advisory services. And hope that the university could

provide negotiations and legal assistance when they want to withdraw those articles; (d) 15.8% and 30.7% of respondents experienced changes in the IF (down to zero or decrease dramatically) which affect the result of their research productivity calculation, respectively. (e) More than 50% of the respondents hope the university to modify the calculation method or the year selection of the IF, to avoid research effort in vain or unable to reach the expected score.

Conclusions: In summary, predatory journals are very often open-access journal, but journals without IF or suppressed/dropped by JCR are not necessary to be a predatory journal. Based on the research findings, the TMUL will strengthen the following measures to help not accidentally submit articles to predatory journals: provide the existing reference list of predatory journals, encourage faculty and researchers to apply for our initial inspection services of predatory journals to verify their background, and provide customized advisory services for each case.

Roots of Medicine: A Collaborative Medicinal Garden Project

Practice Area: Education

Matt R. Regan - Clinical Education Librarian, Hardin Library for the Health Sciences, Iowa City, Iowa

Chris Childs - Clinical Education Librarian, Iowa City, Iowa

Damien Ihrig - Curator, John Martin Rare Book Room, University of Iowa Hardin Library for the Health Sciences, Iowa City, Iowa

Elizabeth J. Kiscaden AHIP - AVP, Library Services, Creighton University, Omaha, Nebraska

Background: In an effort to increase the visibility of rare book room holdings, and to foster inclusion of history of medicine materials in curricula across campus, we proposed to create a medicinal garden with accompanying digital exhibit. In collaboration with representatives from a local gardening group, the college of Pharmacy, and a horticulture expert from a partner institution, a medicinal garden was planted. Informational signs were created with QR codes connecting to a custom website with information on each plant--including images and text from digitized rare books showing historic use, syntheses of contemporary medical research, and links to additional information.

Description: While many medical libraries have rare book rooms or special collections, encouraging their use or incorporating those collections into curricula can often be a challenge. In response to this, librarians at Hardin Library at the University of Iowa have been working on creating an interactive medicinal garden that will promote these collections. Our poster will include descriptions of the process, images of the garden, examples of the physical plant tags with QR codes, and examples of plant records included in the accompanying website. We will discuss how we acquired funding, partnered with stakeholders, and identified faculty who plan

to incorporate the garden into their courses, as well as how we overcame numerous challenges along the way.

Conclusion: This program is still in progress. Once completed, we will evaluate the effectiveness of the project through web analytics for the accompanying website, evaluation of course integration, and tracking of rare book room events, visits, and reference transactions connected with the medicinal garden project.

Safety Ambassadors & Concierge Service for Student Study in times of COVID

Practice Area: Information Services

Meredith I. Solomon MLA, AHIP - Senior Outreach Officer, Countway Library of Medicine/Outreach Office, Quincy, MA

Yasmina Kamal - Outreach Coordinator, Countway Library of Medicine, Harvard Medical School, Boston, Massachusetts

Background: The purpose of this poster is to share the development of a Concierge Service which Countway Library of Medicine implemented to allow student study reservations during COVID-19.

Description: Countway Library implemented a Concierge Service to welcome students from the Harvard Longwood Campus back to the library amid COVID-19 restrictions. A seat reservation system was created, and current staff and newly hired part-time staff greeted students and helped them to access our newly renovated space. Having access to study space in Countway Library allowed students to step outside of their isolated homes and dorms into a collaborative space with proper physical distancing and safety protocols in place. This poster will discuss the steps taken to design and establish the Concierge Service Initiative, as well as usage analytics and guidance for similar initiatives.

Conclusion: Countway Library is constantly monitoring this service revising guidelines and usage policies as appropriate. Based on the data collected, as well as anecdotal evidence, this Concierge Service has been a valuable and highly appreciated resource for Harvard Longwood Campus students.

Search Club Supporting Knowledge Exchange and Collaboration

Practice Area: Innovation & Research Practice

Anna M. Ferri - Research & Learning Librarian, Roseman University of Health Sciences University Library, Henderson, Nevada

Megan De Armond MSLIS, MFA - Research & Instruction Librarian/Assistant Professor, Jay Sexter Library, Henderson, NV

Background: As two academic health science libraries serving similar, geographically adjacent institutions that are actively enhancing their research outputs, we created a Search Club to improve our skills in meeting library search requests and systematic review support. The Search Club developed out of an existing collaboration that had proven to be beneficial to our respective institutions and colleagues. The goals of our Search Club were to review and appraise published systematic review searches, improve skills related to comprehensive bibliographic searching and collectively develop a toolkit for systematic reviews that would be tailored to the scope and scale of our respective institutions.

Description: Building off of an existing collaboration, the authors invited colleagues from our respective institutions to join a Search Club for quarterly meetings. For our initial meetings we spent the first hour reviewing a pre-assigned published systematic review search strategy and methodology and the second hour working on a similarly themed research question and strategy. Both authors and one other Search Club member had completed outside systematic review training from different organizations with variation in coverage. Working from those variations, we appraised the research questions, search strategies, and methods section to improve our overall understanding of comprehensive search development. As the initiators of the Search Club, the authors curated systematic reviews and published search strategies, suggested focus areas for appraisal such as research question formats, term selection, synonyms, and tags and proposed the research topic for building a collaborative search strategy.

Conclusion: Due to the pandemic, our meetings didn’t occur as frequently as planned. Our meetings have included librarians who were not responsible for research support as a way of enhancing their understanding of comprehensive search skills and workflows. Our current goal is to create a toolkit designed to meet the unique needs of small health sciences universities, with the expected outcomes of: -Enhancing workflows and communications around literature reviews involving comprehensive searches -Increasing awareness of review article methodologies for students and faculty -Creating time savings through improved methodology and search practices -Reducing duplicated training efforts by our institutions -Increasing the visibility and credibility of our respective libraries -Relationship building among libraries colleagues

Sharing Our Super Power: Promoting Health Literacy Month at a Cancer Hospital

Practice Area: Information Services

Adela V. Justice AHIP - Senior Librarian, MD Anderson Cancer Center/The Learning Center, Houston, Texas

Julie Bradford MA - Associate Director, Patient Education, MD Anderson Cancer Center, Houston, Texas

Jennifer Fonseca MPH, CHES - Health Education Specialist, Patient Education MD Anderson Cancer Center, Houston, Texas

Background: In October 2020, the Patient Education (PE) staff at a large cancer hospital promoted national Health Literacy Month to the hospital’s clinicians and staff using several methods. The aim of this promotion was to build institutional awareness of the importance of health literacy, health numeracy and plain language. This was also a dedicated effort to brand PE as the “go to” source for health literacy, since health literacy is a unique skill of the PE health education and library staff. This month-long promotion also marked PE’s renewed effort to promote health literacy every October going forward.

Description: In August 2020, PE staff health educators and librarians were solicited for ideas on how to effectively promote Health Literacy Month. Twelve suggestions were presented, which were then divided into actions that could be implemented in October 2020, or which could be done in the future. Beginning in September 2020, PE staff met to formulate a promotion plan and implement separate task forces for the ideas that received the most votes as being feasible. These ideas were: a targeted email campaign, crafting weekly email signatures for PE staff to use throughout the month, tying in the library’s LibGuides, developing two infographics, one for health literacy and one for health numeracy, and promoting the infographics on social media and at institutional meetings. This resulted in several task forces that were successful in bringing these ideas to fruition.

Conclusion: The impact/outcome for all activities was measured when possible. Metrics were collected on Tweet impressions, when/where the infographics were shared, and how many times the email campaign was sent out. Some additional avenues of promotion were also able to be utilized, such as the PE institutional Intranet page, one of the hospital’s Facebook groups, and an institution-wide newsletter. Thousands of the hospital’s clinicians were reached via the various promotions, and several accolades and thank you notes were received from faculty and staff. The ideas that were able to be realized will be repeated, while those unable to be enacted upon will be evaluated for future use and, if applicable, pursued for 2021’s Health Literacy Month.

The Status of Scholarly Efforts of Librarians in Health Literacy: A Bibliometric Analysis

Practice Area: Innovation & Research Practice

Alexandria Q. Wilson - Assistant Professor/Research & Learning Services Librarian, Preston Medical Library, University of Tennessee Graduate School of Medicine, Knoxville, Tennessee

Courtney Wombles - Graduate Research Assistant, Preston Medical Library, University of Tennessee Graduate School of Medicine

Robert E. Heidel PhD, Stat - Associate Professor of Biostatistics, Surgery, Knoxville, Tennessee

Kelsey L. Grabeel AHIP - Assistant Director, Preston Medical Library / Health Information Center, University of Tennessee Graduate School of Medicine / University of Tennessee Medical Center, Knoxville, Tennessee

Objectives: Over the past 20 years, health literacy has increasingly impacted patient care. Recently, Healthy People 2030 updated the definition to include personal health literacy as well as organizational health literacy. As information professionals, librarians can be leaders of helping to improve health literacy through their roles in the classroom, public library, or in the healthcare environment. In order to determine the scholarly impact librarians may have had on health literacy, researchers examined the status and characteristics of health literacy (HL) publications authored by librarians from 2000 to 2019.

Methods: Bibliometrics, the statistical analysis of publications, is used to assess the indicators of productivity, collaboration, and impact or visibility of librarians in health literacy-related research. Data was collected using the multidisciplinary database Scopus and articles were screened for inclusion criteria using Rayyan. Through bibliometrics, quantitative analysis will be performed, including examination of number of citations, number of publications per year and per country, the most prolific authors and their affiliations, most prolific journals and journal impact factors, number of articles published in library science-related and non-library science-related journals, the origin of the first author’s country, number of articles per research type, etc. The itsa (Interrupted Time Series analysis) procedure in Stata was used to assess the trends in publications pre- and post-2013.

Results: Of 321 search results, 219 unique references met the inclusion criteria of librarian authorship. Before 2013, the rate of HL publications increased at a rate of 1.30 annually (95% CI 0.97–1.62, $p<0.001$). After 2013, the number of HL publications increased at a rate of 4.18 publications (95% CI 3.71–4.65, $p<0.001$) annually with the peak in 2019 (n=32). Journal of Consumer Health on the Internet was the most prolific journal. The majority of included references were authored by at least two authors with a collaboration index of 2.58. In terms of impact, 79% of included articles were cited at least once.

Conclusions: In the last two decades, librarian involvement in HL publications has exponentially increased, most markedly in the years following 2013. Librarian authors are highly collaborative, but few have published on health literacy multiple times. The visibility, volume, and collaboration efforts show how engaged librarians are in health literacy scholarship. Further research is needed to examine the research and impactful efforts made by librarians that aren’t published.

Stress and Coping among Nursing Home Staff during the COVID Pandemic

Practice Area: Innovation & Research Practice

Amy G. Lyons AHIP, FMLA - Associate Librarian, University at Buffalo, Health Sciences at Abbott Library, Buffalo, New York

Audrieanna E. Raciti - Family Nurse Practitioner , University at Buffalo , New York Mills, New York

Margaret Doerzbacher - Clinical Instructor, University at Buffalo School of Nursing, Buffalo, New York

Yanjun Zhow - University at Buffalo

Cristina de Rosa - Graduate Research Assistant, University at Buffalo, Bismarck, North Dakota

Chia-Hui Chen - University at Buffalo

Yu-Ping Chang - University at Buffalo

Yanjun Zhou - Graduate assistant, School of Nursing, University at Buffalo SUNY, Amherst, New York

Objectives: The purpose of this study is to explore the stressors and coping mechanisms of staff working in a nursing home environment during the COVID-19 pandemic. Nursing homes house our most aged and vulnerable populations, contributing to the high levels of Coronavirus illness and death. Healthcare staff who are often emotionally engaged and working in such close proximity, pose an increased risk to these highly susceptible populations in nursing homes. Since there is still much to learn related to COVID-19 we turned our attention to investigating the emotional toll and impact of healthcare staff working with those residing in nursing homes.

Methods: Institutional Review Board (IRB) approval was received to conduct a qualitative study using a semi-structured survey for administering individual interviews. A series of the same 12 open ended questions were posed to each staff member. Participants were nursing home staff currently employed or were employed by one of two New York State long term care

facilities during the COVID-19 pandemic. Interview data were transcribed verbatim and then analyzed using Braun and Clarke’s Reflexive Thematic analysis. Thematic analysis results in explaining people’s experiences, perceptions, views, or representations of a given topic.

Results: Results focus on twelve nursing home staff participating in the study. These included 2 nurse practitioners, 7 registered nurses, 1 executive director, 1 social worker and 1 physical therapist. Of the participants 11 were female and 1 male. Collectively, their experience working within a long-term care facility ranged from 7 months to 25 years. Based upon the data, four main stressors were identified and five helpful coping strategies were shared by study participants.

Conclusions: Staff of both nursing homes describe their experiences as exhausting, frustrating, and constantly uncertain, as demands change daily. Several describe that counseling services, leadership, colleague support and family support enabled them to address and manage the constant stress they experience. Of particular concern is the coverage and shortage of staff available to address patient needs resulting from the COVID-19 pandemic. Furthermore, staff articulated worry about a second wave. From these findings, the researchers believe they help to understand the impact COVID-19 has on staff located in nursing homes. Healthcare staff working in direct patient care require ongoing support to address stress and prevent staff turnover. Further research needs to identify stress reduction interventions to assist nursing home staff.

Substance use disorders and the evidence for technology-based tools and resources: A Content analysis

Practice Area: Clinical Support

Zach Sneed - Assistant Professor/Program Director, Clinical Counseling and Mental Health/Psychiatry, Lubbock, Texas

Amy Faltinek MLS, CAS, AHIP - Senior Director, Technical Services, TTUHSC Preston Smith Library of the Health Sciences/Technical Services, Lubbock, Texas

Regina Baronia MD, M.Ed. - Research Associate, Department of Psychiatry, Lubbock, Texas

Susan E. Bergeson PhD - Professor, Cell Biology & Biochemistry, Lubbock, Texas

Peggy J. Edwards - Unit Manager - Informationist/Medical Librarian, Texas Tech Univ. HSC, Lubbock, Texas

Objectives: With increasing sophistication and access to Internet technology, there is a corresponding increase in web-based treatments for substance use disorders (SUD). The use of technology can also greatly increase the kinds and amount of data useful to researchers. Our objectives were to explore, collect and categorize the various presentations of evidence

regarding technology-based tools and resources for SUD examining: how widespread are technology-based SUD treatments and the types of evidence available to support the use of technology. How strong and complete is the literature base for technology-based treatment for SUD?

Methods: There is a gap in understanding and using the knowledge base around technology-based SUD treatment interventions. There are existing studies spanning this literature; however our study took a more in-depth review of a wider range of publications. We conducted a content analysis via systematic review methods using PubMed, Embase, Cochrane, CINAHL, Ageline, PsycInfo, Rural Health Information Hub, WorldWideScience.org, ClinicalTrials.gov, Scopus and Web of Science. This process returned 3,222 references which were imported into Covidence for review. Removing 396 duplicates left 2,825 studies. Next, 2,102 irrelevant articles were excluded, leading to 720 full text reviews. This phase included interlibrary loans, interrater reliability, and conflict resolution strategies excluding 301 articles. Final results are based on the full-text review of 410 studies.

Results: The literature base is wider than ever before. There is evidence that technology can be used to deliver and augment treatment or recovery-based services; however more can be done to strengthen and organize the literature. Final results include a wide array of information such as article focus (research or conceptual); type of research (clinical trial, group experiment, single-subject, systematic reviews); and a summary of the included studies’ research questions, keywords and/or specific aims. We accomplished this via a mixture of charts, graphs, and images.

Conclusions: Technology has the ability to impact clinical implications/outcomes related to SUD. A significant variety of technology-based SUD tools and resources exist; however much of the recent literature struggles to use common definitions necessary for progress. Some products have existed for years and possess a substantial evidence base; however other research presented in final form is lacking typical components of published work related to methodology and efficacy. A variety of opportunities exist to expand or augment clinical services and research. Our presentation provides a methodologically rigorous and relevant synopsis. This information can be helpful to health science librarians by coalescing and parsing a diverse and complex area.

Through the Eyes of Our Researchers: Enhancing the Collaborative Role and Value of Librarians Within the Systematic Review Team

Practice Area: Innovation & Research Practice

Donna S. Gibson - Director of Library Services, Memorial Sloan Kettering Library, New York, New York

Kendra Godwin - Research Informationist II, Memorial Sloan Kettering Library, New York, New York

Robin O’Hanlon - Associate Librarian, User Services, Memorial Sloan Kettering Library, Astoria, New York

Celine Soudant - Research Informationist II, Memorial Sloan Kettering Library, New York, New York

Konstantina Matsoukas - Research Informationist III, Medical Library, Memorial Sloan Kettering Cancer Center, New York, New York

Objectives: Systematic review (SR) services based in a health care environment can benefit from ongoing assessment and evaluation. By performing regular assessments, librarians can uncover opportunities to add value to the SR process and research team. To determine areas for improvement, understand our researchers’ perceptions, and where librarians might further assist, we compared responses from two similar client satisfaction surveys (administered at a 3-year interval) sent to researchers who used the service. As SR services grow and evolve, so too should the tools used to evaluate them to better inform plans for service enhancements and crafting marketing messages.

Methods: In 2017 and 2020, client surveys were distributed to measure satisfaction with our Library’s SR service. Several questions from the 2017 survey were repurposed and administered to a similar audience, researchers who had submitted a request for support and published a SR in collaboration with their assigned librarian. Based on responses from the original survey, three new questions were added to the second survey to further explore our current SR process and identify tasks within the SR process where the researchers felt the librarian had the greatest impact or where they could have benefited from more support. A selection of first time and repeat service users were identified for interviews to tease out additional information about the librarian’s role. An analysis of both surveys and interviews reveals action items to enhance our service.

Results: The 2020 survey response rate was slightly larger, with 27 completed surveys (22 in 2017). Feedback was similar regarding client satisfaction, service recommendation, knowledge and quality of work completed by the librarian, and benefits from using the service. Both surveys’ findings revealed that some clients thought the SR process was slow, exacerbated by staff turnover or competing responsibilities. A new 2020 question highlighting researchers’ selection of where they saw the greatest impact of the librarian demonstrated that traditional librarian functions topped the list. Eleven client interviews were completed, uncovering other practical ways in which librarians could support SR teams.

Conclusion: The findings from both surveys and the interviews confirmed overall service value and benefits of working with an assigned librarian. The interviews provided specific ways that librarians could increase their collaboration on the SR team: proactively scheduling brief progress check-ins, setting expectations for obtaining full-text content (article management), ongoing identification/sharing of cutting-edge SR tools, developing a SR team task checklist, and simplifying the SR request process.

“Together We Learn”: Collaborating with Medical Students to Build an Online Anti-racism Collection

Practice Area: Education

Gary S. Atwood - Education Librarian & Library Assistant Professor, Dana Medical Library, Burlington, Vermont

Kate E. Bright - Assistant to the Director, Dana Medical Library, Burlington, Vermont

Background: In the summer of 2020, students from the Larner College of Medicine's Social Justice Coalition (SJC) asked if the library could support their project to build a digital collection of anti-racism resources. Specifically, they asked if the library could host the collection and purchase relevant materials when possible. The project presented interesting policy and technical issues related to LibGuides, the platform we eventually selected. It also provided educational opportunities for the medical students, staff, and the library. After six months of hard work, we launched the collection, titled “Together We Learn: Resources for Our Journeys in Justice & Inclusion” in December 2020.

Description: While, initially, it appeared that this would be a simple project to complete, we quickly discovered unique challenges. For example, LibGuides was chosen as the technical platform to use, but there was a consensus among the poster authors, students, and their advisor that the students should retain editorial control over the content. This ran counter to library policy and we had to submit a request for an exemption so that the SJC could act as an editor, which was granted. The project team also worked closely on design. This involved educating the students and their advisor about how LibGuides operates, which impacted how content appeared. We also had to collaborate with the library's Collections Development team to address issues related to purchasing materials. Finally, we had to develop new workflows to address issues like reviewing and updating guide content. This required another adjustment to established library policy. The final guide includes a rich mix of resources, such as books and videos, that cover a variety of crucial topics.

Conclusion: The guide was launched in December 2020. The initial reception was enthusiastic and supportive with hundreds of views that month. Like many end-of-year projects, however, it was soon overshadowed by the holidays, the January 2021 spike in COVID-19 infections, and events in Washington D.C. Our goal for the spring 2021 semester is to reintroduce the guide to the public. The medical students and their advisor are developing a plan to accomplish that goal. We also need to grow the guide and we have already started to receive suggestions via an embedded “Recommend a Resource” form. As a result, the guide remains an ongoing project. We are honored to continue working with our medical students on something they are so passionate about.

The Top-Cited Articles in Osteopathic Medical Education: a Bibliometric Analysis

Practice Area: Information Services

Adrienne Brodie - Liaison Librarian, A.T. Still Memorial Library, Mesa, Arizona

Harold S. Bright AHIP, IV - University Library Director, Library, Mesa, Arizona

Laura M. Lipke AHIP - Medical Librarian, A. T. Still Memorial Library, Kirksville, Missouri

Lori A. Fitterling - University Library Director, Kansas City University, Kansas City, Missouri

Sloane G. Kelley MS - Library Director, NYITCOM at A-State Library, State University, Arkansas

Kyle D. Robinson MLIS - Electronic and Technical Services Librarian, California Health Science University, Coarsegold, CA

Objectives: This study identifies the 50 top-cited articles pertaining to osteopathic medical education from the Web of Science database and from seven prominent journals not indexed in this database. Guided by previous bibliometric research, we systematically examined the scientific literature about osteopathic medical education and captured their bibliometrics. The end goal was to learn about recurrent topics and authors associated with Colleges of Osteopathic Medicine.

Methods: Three search strategies were used to extract articles for this project: (1) Combined search terms for “osteopathic medicine” and “medical education”; (2) Combined search #1 with a search of authors from osteopathic medical schools; and (3) 7 journals which are known to publish osteopathic articles and are not indexed in the Web of Science database, were searched with the combined terms for search #1. Bibliometrics for the top articles were gathered through Google Scholar. Inclusion and exclusion criteria for all articles were identified and applied to the final results.

Results: After examining over 191,250 articles, 50 articles were identified that met the search criteria and were listed according to the number of times cited metric. Common themes identify what specific osteopathic topics are published, and where there is a gap in the literature on osteopathic medical education. The top educational main topics were medical education and anatomy education. One third of the articles discussed medical topics, such as anatomy, empathy, interprofessional education, osteopathic, surgery and technology.

Conclusions: Bibliometric analysis provides a way to analyze the landscape of scholarly research and authorship in scientific publishing and this study concludes that osteopathic medical education is a vibrant topic in scientific literature.

Transforming EBP Workshops: Transitioning from In-Person to Online Using MS Teams

Practice Area: Education

Beverly Murphy, MLS AHIP, FMLA - Assistant Director, Web Content & Development; DUHS Hospital Nursing Liaison: Watts CON Liaison, Duke Medical Center Library & Archives, Durham, North Carolina

Virginia M. Carden, MLS AHIP - Administrative Research Librarian, Duke University Medical Center Library, Durham, North Carolina

Deborah Allen, PhD, RN, CNS, FNP-BC, AOCNP - Nurse Scientist, Duke University Health System, Durham, North Carolina

Joan Cahill, RN, BNS, OCN, CCRP - Nursing Research Program Coordinator, Duke University Health System, Durham, North Carolina

Background: This poster describes the transformation of an in-person evidence-based practice (EBP) workshop series to an online format using MS Teams. Due to the ongoing pandemic, it became necessary to transform “Mitigating the Madness,” a three-part series of hands-on workshops (#1: Clinical Inquiry, #2: Project Development, and #3: Project Dissemination) incorporating didactic presentations, roundtable discussions, literature searching, and writing, to a format that would be virtually accessible. These workshops involve the interprofessional partnership of medical librarians, clinical nursing leaders, and nursing faculty.

Description: When faced with the inability to meet in person due to the pandemic, the Mitigating the Madness Planning Committee quickly pivoted to develop a user-friendly workshop using an online platform. They investigated several options for implementation and decided to use MS Teams due to its accessibility and functionality. Simultaneous work to develop and implement the transformation centered in two areas. Clinical nurse educators and the medical librarian developed content for resource files, recorded presentations, and organized coaching assignments for asynchronous learning. Within MS Teams, the medical librarian and nurse researcher created the workshop virtual site and loaded content for access by participants and coaches. Across the workshops, 17 recorded presentations and 14 coaching assignments were created. Upon completion of a workshop module, a 2-hour virtual, touchpoint session was held where all participants and coaches were present.

Conclusion: Since transitioning to the online format, we have held four workshops with an average attendance of 10-12 people including participants and coaches. During the touchpoint session, participants and coaches were able to share experiences and give feedback for improving the process. Robust discussions centered around PICO question

formulation, database searching, literature appraisal, and project design. Feedback from participants has been positive with minimal challenges indicated. Issues included lack of familiarity with Teams, following instructions, and timeliness of completing assignments. Coaches continue to explore methods to promote participant engagement through the online platform. The transition from in-person EBP workshops to online using MS Teams has been well received by participants, coaches, and presenters.

Transforming Evidence: Measuring the Prevalence of Preprint Inclusion in Systematic Reviews

Practice Area: Innovation & Research Practice

Elizabeth G. Hinton MSIS, AHIP - Associate Professor/Research & Instruction Librarian, Rowland Medical Library/University of Mississippi Medical Center, Jackson, MS

Objectives: In the wake of COVID-19, submission of health sciences preprints has grown exponentially. While grey literature should be included in any comprehensive systematic review, some preprint servers emphasize that preprints should not be relied on to guide clinical practice. This could be considered in direct opposition to the goal of evidence synthesis. Considering the rigor of a systematic review and meta-analysis, this exploratory study aimed to discover how often preprints are included in systematic reviews.

Methods: PubMed, Web of Science, the JBI Library, and the Cochrane Library were searched from inception to April 2021 for systematic reviews that included bioRxiv, medRxiv, or ChemRxiv in their information sources. Systematic review protocols were not included in the search. Results were imported into EndNote to remove duplicates and errata. The bibliographies of the remaining citations were searched for inclusion of preprint citations in the final review. Results were then analyzed by discipline, journal, country, and type of review to determine any publication trends.

Results: A total of 212 systematic reviews were included in the analysis. The reviews were published from 2019-2021 and 98% were related to COVID-19. A sample of the results indicates that general internal medicine was the most common discipline with 39% of the citations. Out of 154 publications, PLOS One was represented the most frequently (seven reviews), and six of the reviews were published in medRxiv. Reviews with at least one author from the United States were the most common (32%), and 85% of the citations followed the methodology of a quantitative systematic review. Other systematic review methodologies included living, rapid, scoping, and, remarkably, one umbrella review.

Conclusions: Owing to medRxiv’s relatively recent inception and its role in pushing COVID-19 research forward, the number of reviews concerning COVID-19 is to be expected. While most of the analyzed reviews included only one or two preprint citations, several included a significant number of preprints. When accounting for the lack of evidence published through

traditional channels during the COVID-19 pandemic, the synthesis of preprints is understandable; however, basing clinical practice off of non-refereed publications should be done with an abundance of caution. As this was an exploratory analysis, future data collection would certainly capture reviews beyond COVID-19 and offer further insight into the impact of preprint inclusion in evidence synthesis.

Using a Citation Manager to Add Levels of Evidence to a Literature Search Results Template

Practice Area: Clinical Support

Mary Pat Gage, MLIS AHIP - Medical Librarian, Library & Research Support, Miami, Florida

Devica Samsundar AHIP - Director, Library & Research Support, Library & Research Support, Miami, Florida

Jessica Salinas, MSI - Medical Librarian, Library & Research Support, Homestead, Florida

Joana Fernandez, MLIS - Medical Librarian, Library & Research Support, Miami, Florida

Background: The American Nurses Credentialing Center Hospital Magnet Recognition requires nurses to understand and apply research that improves patient care, safety, and costs. This happens when they identify the highest levels of evidence and translate them into practice. Research designs have the best evidence based on rigor, population size, validity, and randomization. To support Magnet Recognition efforts, librarians at Baptist Health South Florida updated their search results template to include levels of evidence with the citation, abstract, PMID and database link. To enhance understanding between levels of evidence, the Dartmouth College and Yale University EBM pyramid was added to the form.

Description: We customized our EndNote Output Style by inserting the **“Type of Article”** field, into the Journal Article Bibliography template using the command “Link Adjacent Text” with the following syntax: **`Type of Article:`**°Type of Article. This translated to Word like this example: Type of Article: Review. The “Publication Type” metadata from Scopus and OVID Medline landed seamlessly in Endnote. PubMed required import option “MEDLINE (OVID SP).” CINAHL required the citation file to be exported as “Generic bibliographic management software,” and then use the import option “CINAHL (EBSCO).” We acquired copyright clearance to use the EBM pyramid in our search results form and linked it to an infographic on appraisal of evidence on the last page.

Conclusion: This enhancement added about eight minutes to processing a results list of twenty-five references. The benefit to our patrons outweighed the increased time for the librarians. We are also applying this output style to the Excel Systematic Review Search Results Template. A REDCap survey, to evaluate our patrons’ satisfaction with the new format, will be distributed to all of those who used the search service since the update.

Using an Email Template to Increase Library Class Registrations

Practice Area: Education

Kristen DeSanto AHIP - Clinical Librarian, Associate Professor, Strauss Health Sciences Library / University of Colorado Anschutz Medical Campus, Castle Rock, Colorado

Background: At this academic health sciences library, librarians teach an average of 12 classes each month. In Q1 2020, there was an average of 13 registrations per class. The library wanted to increase this number and thought a different marketing strategy could help. Aside from posting class schedules on the library homepage, emails are sent to the campus listserv, which are seen by all students, faculty, and staff. There was no consistency to the emails regarding content or format, so we proposed creating a streamlined but eye-catching template for emails in order to increase registrations.

Description: In developing the email template, four guiding principles were followed: Library brand reinforcement: The subject line begins with the library name followed by class name. The body of the email begins with the library logo and includes an image of the building; Consistency: This template is used for all library classes; Accessibility: All images contain alt text, and URLs are hyperlinked. Text is written in black sans serif font on a white background with a 12-point minimum; and Simplicity: The most important information is placed at the beginning in larger font: class name, date, time, and a link to the registration page. A brief class description is included, but learning objectives are not. Supplementary information is placed at the bottom: course instructor and email, and links to the library chat service and complete class schedule.

Results: During the 6-month period since the email template debuted in October 2020, average registrations per class increased 50%, from 16 (Q1 and Q3 2020) to 24 (Q4 2020 and Q1 2021). Q2 2020 was not included due to a large increase in registrations in April and May 2020 when library classes moved to an all-virtual format, which was considered an outlier. In relation to the date the email was sent, 52% of registrations were received on the day the email was sent, and 72% on or after the day the email was sent, demonstrating the impact of the listserv.

Conclusion: After implementing an email template built on four guiding principles, average registrations per class increased by 50%. Half of the registrations were received the day the email was sent to the listserv, suggesting that students, faculty, and staff were noticing the emails and being enticed to register for a class. A limitation is that increases in registrations could be due to factors unrelated to the email format.

Using Google Scholar Alerts as Outreach

Practice Area: Information Services

JJ Pionke - Applied Health Sciences Librarian, University of Illinois at Urbana-Champaign, Champaign, Illinois

Background: The purpose of the research publication response email outreach project was to use an innovative way to reach out to publishing professors about library resources, including specialized librarians.

Description: Being at a major research institution, the bulk of faculty are actively working researchers who have Google Scholar accounts even if they might not have other types of accounts like ORCID. Using a faculty list provided by the college that I liaison to, I created Google Scholar Alerts for each faculty member to be sent to my work email. From there, I created a spreadsheet with faculty names on the vertical and topics of emails on the horizontal. I created a series of "canned" emails that highlight a single library resource. When I receive a Google Alert, I check the spreadsheet and send the next email in the sequence. There is no current assessment initiative for this project, though that is the next phase.

Conclusion: This project is ongoing but has not been formally assessed. Informal assessment via email feedback when faculty have received an outreach email has been very positive and has led to faculty using more library resources. The next phase of this project is to create an assessment instrument to more formally measure the efficacy of this outreach initiative.

A Video is Worth a 1000 Words: Creating Videos to Support a Consumer Health Information Page

Practice Area: Information Services

Elizabeth Irish AHIP - Associate Professor, Schaffer Library of Health Sciences, Albany Medical College, Albany, New York

Kara Burke - Director for Community Engagement, Division of Community Outreach and Medical Education Albany Medical College, Albany, New York

Enid Geyer AHIP - Associate Dean for Information Resources and Technology, Albany Medical College, Schaffer Library of Health Sciences, Albany, New York

Ingrid Allard - Associate Dean for Community Outreach and Medical Education, Albany Medical College Division of Community Outreach and Medical Education, Albany, New York

Background: In order to better engage visitors to a "For Health Consumers" website, an academic health sciences library, an academic community engagement office, a public library,

and a community-based organization pooled their strengths to develop supporting videos. Videos combined practical patient empowerment techniques with information finding skills. The four topics were: “Finding a doctor”, “preparing for an appointment”, “talking with your doctor”, and “help from a public library”. The intent was to provide an additional method for sharing the educational information presented on the website in order to engage varied learning styles and empower patients to actively participate in their healthcare.

Description: Project development included pre-production, production, and post-production. Financial support was NNLM/MAR grant funded. In pre-production the scripts were developed collaboratively by librarians and community outreach faculty. Actors were recruited from the standardized patient pool and the public library. A local film crew was hired. Filming locations, a hotel suite and public library, were selected. Rough cuts were filmed to preview to two focus groups. Scripts were revised based on their input. During the production phase, the film crew, actors, and project leadership spent 1.5 days filming. In post-production, the team reviewed the final mastered cuts prior to incorporating the videos on the website. Planned promotional efforts included community screening at the public library and linking to the academic site by community partners. Final evaluation was to include a short survey distributed at a community screening and monitoring video website views.

Conclusion: This project’s strength rests in the community collaboration process. It is an example of how academic and public librarians can work together with academic community outreach professionals and community-based organizations to develop patient empowerment projects. Unfortunately, the evaluation and promotion of the videos was significantly impacted by COVID-19. The community screening and all promotional activities were paused as libraries shutdown and re-imagined services. There have been preliminary discussions to reschedule the community screening when public libraries re-open. Although views have not reached initial expectations, patients have been postponing doctor appointments, reducing the current need. As partners continue to adapt their workflows to the ongoing pandemic, the team plans to resume promotional efforts, including linking the videos to the partners’ websites.

What to Do When Getting in Small Groups and Playing with LEGO™ is the Worst Idea: Preserving Gameful Learning in a Pandemic

Practice Area: Education

Nena Schvaneveldt AHIP - Assistant Librarian, Eccles Health Sciences Library, Salt Lake City, Utah

Donna Baluchi - Library Supervisor, Spencer S. Eccles Health Sciences Library, Salt Lake City, Utah

Brandon Patterson - Technology Engagement Librarian, Eccles Health Sciences Library, Salt Lake City, Utah

Background: One library in the Intermountain West has had success integrating gameful learning and interactive elements into library instruction. However, as instruction shifted online due to the pandemic, we were faced with the challenge of maintaining engaging, meaningful learning in an online environment. We had developed and adapted lessons to use gameful learning principles to teach plagiarism, citation, the research process, and database searching. In Fall 2020 and Spring 2021 in response to the COVID-19 pandemic, the library did all instruction virtually, which made previous games, some of which included physical pieces, very difficult to modify.

Description: The online environment necessitated some changes. Some games, such as a plagiarism activity involving LEGO, were, unfortunately, transitioned to lecture format. A game involving putting citations together like puzzles was moved into a quiz in LibWizard; however, students found the tool’s rigidity frustrating. Team games were easier to facilitate as individual games. A team-based database searching game was successfully converted to an individual format, then play-tested with a small group of library colleagues, who provided feedback to enhance the game. A group activity based on a board game about the research process was converted into a Choose Your Own Adventure story with a reflection prompt at the end of the lesson.

Conclusion: Some of the format changes for the online environment improved the games overall: for example, providing links is far easier over Zoom, and can ensure students are looking at the correct resource. However, further testing and development is warranted for some games, especially those around citation and plagiarism. Student feedback for these games remains overwhelmingly positive, as it was in-person. Students appreciate the interactivity of the games. Online modules developed, such as the Choose Your Own Adventure module, may be easier to incorporate in instruction as they can be completed individually on the students’ own timeframes. The development of these games will be an enhancement to the library’s gameful learning curricula, rather than a replacement to the in-person games.

Who is COUNTING? A publishing company support team’s quest to understand the diverse ways librarians acquire, analyze, and disseminate usage statistics

Practice Area: Information Management

Dana Thimons - *Manager, Customer Success, McGraw Hill*, *Aberdeen, North Carolina*

Ashley Weinheimer MLIS - *Customer Success Librarian, McGraw Hill Professional Group, Pennsylvania*

Wesley Holloway, MLS - *Customer Success Librarian, McGraw Hill, New York, New York*

Lauren Weiner MA, BSEd - *Customer Success Coordinator, McGraw Hill*

Background: As librarians on a support team at a publishing company, we provide usage statistics for customers and basic assistance analyzing the data. The recent transition from COUNTER 4 to COUNTER 5 left us confused about COUNTER 5 and more aware of our need to gain a better understanding of usage statistics in general. We sought insight into how well librarians understand COUNTER 5, which usage reports librarians are using, and how publishing companies can better support librarians’ analysis of usage data.

Description: The team began by creating a usage statistics task force to interview librarians at various institutions. We recruited volunteers for 30-minute interviews to discuss which usage reports they pull, what they consider “good usage,” if they share usage statistics with internal or external stakeholders, and how we can better support them throughout this process. While helpful, interviews require a significant time commitment from the librarians and our team. To gather more information, we created a survey that we plan to distribute to several library listservs. The survey builds upon the interview questions and asks how librarians are calculating cost-per-use, if they are using pivot tables or other tools to manipulate data, and if they are defining good usage differently this year than they did prior to the pandemic or seeing any other pandemic-related trends.

Conclusion: While we do not have the ability to change how our company reports usage, our goal was to gain insight into ways our team can better support librarians throughout the statistics collection process. From the librarians we interviewed and surveyed, there was a wide range of responsibilities surrounding usage statistics, as well as a range in how deeply librarians are analyzing data. Based upon what we learned from this project, we created Excel templates that enable librarians to easily graph their usage statistics to share data with leadership and other stakeholders. We also identified key actions we can take, such as implementing proactive outreach to provide login information for users to download their own usage reports for our products

WTF While WFH: A “What the Fun” Committee Focused on Having Fun While Working from Home

Practice Area: Professionalism & Leadership

Adela V. Justice AHIP - Senior Librarian, MD Anderson Cancer Center/The Learning Center, Houston, Texas

Background: In March 2020 a hospital’s Patient Education (PE) staff transitioned to working remotely full-time due to the pandemic. This included the consumer health/patient library staff, since the library is part of the PE department and the library was closed temporarily. With the

staff working from home, the pandemic caused several social activities to be cancelled such as the staff retreat, birthday breakfasts, holiday parties, and more. When it was announced that staff would keep working from home into 2021, some members formed a committee that would focus on implementing ways to have fun together during work, while at home.

Description: After PE staff supervisors approved the new committee, all 15 PE staff members were petitioned for interest, with five ultimately joining the committee. Coined the “WTF: What the Fun” committee, the first task was to brainstorm ideas that could connect staff in fun ways remotely and virtually, synchronously and asynchronously. Several ideas were implemented, with the first being an October pumpkin decorating contest. In December an online pet show was launched, and in January it was PE staff favorite book quotes plus a "fun hat" department Zoom meeting. The asynchronous activities were enabled by submitted photos and text to shared Powerpoints. Future activities include a favorite movie quotes Powerpoint, Mardi Gras mask/beads department Zoom meeting, online coffee catchups, and others. The goal is to have one or more activities per month while staff continues to work from home through 2021.

Conclusion: The WTF committee made it clear from the beginning that participation in any of these activities would be completely voluntary. Of the 15 PE staff, all have participated in one or more activities. The shared Powerpoints on the staff common drive have been an easy way to implement asynchronous activities. The hospital provides full Zoom access to employees, so activities occurring on Zoom are not encumbered by time limits. The committee’s future ideas for work from home fun will hopefully include some that appeal to all PE staff until everyone can return to the office and resume team-building and social activities in person.

Zoom is Exhausting: Intentionally Going Asynchronous

Practice Area: Education

Nena Schvaneveldt AHIP - Assistant Librarian, Eccles Health Sciences Library, Salt Lake City, Utah

Donna Baluchi - Library Supervisor, Spencer S. Eccles Health Sciences Library, Salt Lake City, Utah

Background: Librarians at one intermountain west academic health center are embedded in a pre-health professions course for first-year college students. The goal of this partnership is, in part, to form positive relationships with future health professions students at their entry to higher education. During each semester, librarians meet with each section five times in total: four in-class sessions and one small group meeting with students. Previously, the in-class sessions mixed lecture, activities, and discussion; however, when the class moved to a blended format in Fall 2020, the librarians assessed whether synchronous or asynchronous learning would work best for all parties.

Description: In Fall 2020, the librarians delivered most instruction synchronously. This included a game, although most sessions were lecture-focused. While the sessions went well, teaching over Zoom is difficult with a lack of nonverbal feedback. Additionally, feedback from students indicated that long Zoom presentations were not desired. With this in mind, the librarians chose to conduct all Spring 2021 sessions asynchronously. The recorded lectures were intentionally delivered in a more approachable format, e.g. using memes and animated GIFs. Students’ assignments focused on reflecting and intellectually engaging with content over rote memorization. The librarians will still schedule a synchronous virtual meeting with each team of students to offer personalized help. In response to student feedback, the librarians do not require students to turn on their cameras.

Conclusion: Student feedback on this design has not yet been received, but will be prior to May 2021. Developing intentionally asynchronous courses for Spring 2020 has been less exhausting for the librarians, who tried to maintain a level of engagement and enthusiasm through lectures. Preserving our ability to teach is vital to the primary goal: to foster a positive relationship with the students to enable them to better grasp the library’s utility in their studies.

RESEARCH TRAINING INSTITUTE POSTERS

An Evaluation of Point of Care Tools by Nurses: How Do They Compare?

Annie Nickum AHIP - Information Services and Liaison Librarian, University of Illinois-Chicago, Chicago, Illinois

Objective: To answer clinical questions quickly at bedsides, healthcare practitioners need point-of-care tools (PoCTs). This study compared three PoCTs from a nursing perspective to determine which resource best met the unique information needs of practicing registered nurses at a large midwestern university hospital.

Methods: The PoCTs selected for review were ClinicalKey for Nursing, Nursing Reference Center Plus, and UpToDate. After informed consent was obtained prior to the start of the survey, participants were asked to choose one of three categories for clinical questions: guidelines and understanding of disease; assessment and diagnosis; and nursing interventions and medication information. Then they were given three clinical questions to answer using the PoCT, spending approximately three minutes per question. After they completed their searches, respondents returned to the survey to rate their experience using the PoCT in areas such as currency, layout, navigation, and use of filters. They also were asked to indicate familiarity with each PoCT, their overall opinions, and demographic information The authors recruited registered nurses at BLINDED through nursing hospital listservs and flyers in common areas asking them to complete the survey. The survey was open for the last six weeks of 2020. Participants were told the survey would take approximately half an hour. To encourage participation, nurses would have the option to enter a drawing for gift cards.

Results: Participants ranged in experience from less than five years to over thirty with the majority being employed full-time as inpatient nurses. Approximately half of participants held a Bachelor’s, a third had a Masters, and the remainder either an Associate’s or a doctoral degree. Participants were most familiar with UpToDate, but average scores for for most areas were similar across all three PoCTs. The exception was filters in which UpToDate was favored. Answers to open ended questions suggest brand-recognition is a factor.

Conclusion: The perceptions of nurses towards PoCTs were markedly different than what had been anticipated by librarians. There is a clear need for education on both sides – nurses can better learn how to utilize these PoCTs and librarians can learn more about what type of information nurses are seeking.

Bibliotherapy for Stress Management: A Wellness Intervention for First Year Medical Students

Rebecca A. Morin - Head of Research & Instruction, Hirsh Health Sciences LIbrary, Boston, Massachusetts

Amy E. LaVertu MLS - Associate Librarian, Hirsh Health Sciences Library/Research & Instruction, Boston, MA

Bibliotherapy, loosely defined as reading for therapeutic effect, has its modern roots in the Library War Service of the First World War. In collaboration with physicians, librarians “prescribed” books to soldiers recovering from illness or injury, in efforts to boost the spirits and bolster the mental health of invalids in military hospitals. In the 21st century, bibliotherapy has been deployed in clinical settings and in a public library-led community self-help context. Particularly popular in the UK, these bibliotherapeutic interventions often take the form of a book group or community discussion of a traditional self-help text. Medical students were determined to be an ideal population to examine applicability of bibliotherapy in an academic setting. Recent studies indicate a high prevalence of “high emotional exhaustion, high depersonalization, and burnout” among medical students, as well as a greater likelihood of depression and fatigue as compared to other US college graduates aged 22-32. The COVID-19 pandemic has introduced further disruptions in personal and academic life, with greater than 40% of respondents to a recent survey indicating elevated adverse mental health conditions. Cognition researchers theorize that consumption of tragic/horror narratives enable readers to mentally prepare for challenging situations, a concept seemingly supported by the 2020 reappearance of pandemic novels such as Stephen King’s The Stand and Albert Camus’ La Peste, on best-seller lists, and the spring 2020 appearance of Steven Soderbergh’s 2011 film Contagion at the top of Netflix’s most-streamed list. Building on this concept, this study aims to determine if bibliotherapy, in the form of short excerpts of fiction and nonfiction related to plagues and pandemics, can serve as an effective intervention to improve perceptions of stress and anxiety among first year medical students. Due to severe demands on the time and attention of medical students, as well as the physical distancing required due to COVID-19, this intervention will replace the conventional self-help book with short readings (reading time ranging from 11 to 32 minutes) and an independent, self-directed structure. Due to the limited number of data points that can be gathered during an academic semester, the study is not expected to yield statistically significant results but will serve as a model for future research and potential wellness programming. As the study is still in progress, this poster reports trends unfolding in real time, not a complete analysis of results.

Evaluation of Distiller’s AI Function for Title/Abstract Screening in Systematic Reviews

Kearin Reid AHIP - Medical Librarian, College of American Pathologists, Buffalo Grove, Illinois

Objectives: Systematic reviews (SRs) are time-intensive, and the use of artificial intelligence (AI) has the potential to reduce the time required for the systematic review process. Our objective is to determine if the AI function of DistillerSR is comparable to the conventional dual review by human subject matter experts during the title/abstract phase of the SR process.

Methods: This analytical comparative study analyzes the AI function of DistillerSR for the title/abstract review of references during SRs. We performed a retrospective review of two SRs using our conventional method of dual review by human subject matter experts. To determine the equivalency of DistillerSR’s AI function, we created new projects using the same pool of references as the original projects and created an AI training set using the historical data from the original review. Then, we applied the DistillerSR’s AI tool to the remaining references and reviewed and compared the outcomes of the review methods to investigate equivalency. We calculated the sensitivity and specificity of the AI function and assessed the similarities and differences in the results obtained.

Results: To determine sensitivity and specificity, we compared the articles included at the title/abstract screening stage with the final pool of evidence included in the published guidelines, as that is of primary importance to our subject matter experts. Our pilot project sensitivity calculations for DistillerSR’s AI tool do not currently meet our acceptability threshold to allow the inclusion of this AI tool into our guideline development process.

Conclusions: Further review is planned to continue evaluating the individual projects for possible reasons for the discrepancies. These projects vary in size and scope and this may significantly impact the tool’s performance as well as the literature search itself. There is potential that AI can be combined with human review in order to maximize the AI tool’s capabilities while incorporating the expertise of human subject matter experts, but it cannot be used as a single reviewer.

Experience Required: A Survey of MLIS Student Experiences with Graduate Employment in Libraries

Christi Piper AHIP - Informationist, University of Colorado Anschutz Medical Campus Strauss Health Sciences Library, Aurora, Colorado

Samantha Wilairat - Graduate Assistant, University of Colorado Anschutz Medical Campus, Lakewood, Colorado

Objectives: Little research has been done to assess MLIS students’ knowledge of or interest in graduate employment jobs in health sciences libraries. Scherrer (2010) reported that 87% of the former academic librarian residents they interviewed stated that professional experience in an academic library was important to them, while 75% stated that professional experiences in health sciences was important to them. Goodsett and Koziura (2016) found that information professionals and LIS students value hands-on experience. It remains to be seen why health sciences libraries have a difficult time recruiting graduate students when hands on experience is highly valued by students and information professionals alike.

Methods: The study population is MLIS students currently enrolled in an ALA accredited graduate program within the United States. To answer the research questions, we have administered a Qualtrics survey that asks students about whether or not they are employed during their graduate program and how they decided on the position they did. If students are not employed, we will ask them about factors that impacted their lack of employment in libraries. Following the survey, participants were asked to volunteer for a follow-up interview that looked specifically at understanding health sciences librarianship. This poster will only report on the survey results of the project.

Results: The poster will include a summary of the survey results and discuss reasons why graduate students do or do not hold graduate employment positions in academic libraries. The results will include information about what aspects of a library position are most important to students and what are factors that hinder a student from having graduate employment. Results will be stratified by gender, race/ethnicity, and caregiving responsibilities to offer insight into barriers and expectations for different student groups.

Conclusions: There are a number of different challenges and opportunities academic librarians may face when recruiting graduate students for employment positions during their MLIS programs. We hope to understand what graduate students are looking for in graduate employment and how we can best support future librarians in their career goals.

Listening to Voces: Exploring Oral Histories to Identify Health Literacy Gaps in the Latinx Community

Ana Corral - Medical and Health Sciences Librarian, University of Houston Libraries, Houston, Texas

Background: Low health literacy and access to reliable health information serve as barriers to good health, both of which have been brought into stark relief during the COVID- 19 pandemic. Health literacy skills in the United States are assessed using questionnaires or measurement tools supported by the Agency for Healthcare Research and Quality (AHRQ). However, these metrics are dependent on reading skills and an understanding of English-language medical terms, an overreliance which ignores the sociolinguistic factors and nuances that affect health literacy skills of communities that have been historically marginalized; in this case, the Latinx community.

Methods: This project will reuse existing oral history data to explore the intersection of health literacy, health information access, and language in the U.S. Latinx community during the COVID-19 pandemic. Employing a social ecological model of health information behavior, combined with critical librarianship theory, and the concept of Nepantla, I will analyze data from the Voces of a Pandemic oral history project from the Voces Oral History Center, University of Texas atAustin. Purposive sampling with be conducted from the current data sample of 252 English- and Spanish-language oral history interviews.

Nurses, Empathy, and Graphic Medicine: a recipe for success?

Melanie Sorsby - Medical Librarian, Covenant Health Center, Lubbock, Texas

Objectives: This study sets out to determine whether reading examples of graphic medicine which depict patient/caregiver perspectives and experiences with illness can increase empathy levels in registered nurses in the acute care setting.

Background: Empathy is an essential trait in nursing yet it is one that can be negatively impacted for a variety of reasons, such as time in service, exposure, burnout, and compassion fatigue; however, the converse can also be true, in that burnout, compassion fatigue, etc. can affect nurses ability to show empathy. “Graphic Medicine” is defined as “the intersection between the medium of comics and the discourse of healthcare” on the graphicmedicine.org website. Multiple studies have been conducted to show that exposure to Graphic Medicine can increase empathy in medical students, residents, and physicians; however, very little research has been done with nurses.

Method: This mixed methods study will use a qualitative focus-group methodology to determine whether there is a change in empathy in registered nurses after three monthly book club type meetings using graphic medicine titles. A series of generic prompts will be available to aid in discussion. Comments will be categorized by theme. Investigators will measure the registered nurses’ empathy using the Interpersonal Reactivity Index (IRI) scale (28 items on a 5-point Likert scale, with four subscales), both before and after reading the three books and participating in the focus-groups. Results: This research project is still in the early stages, but the investigators hope to show a positive association between graphic medicine and nurse empathy.

Conclusions: TBD

Preferred Professional Development Methods of Health Science Librarians

Maggie Shawcross - Health Sciences Library & Assistant Professor, University of Northern Colorado, Greeley, Colorado

Participating in professional development (continuing education) is an important component of librarianship. This is especially important in the health sciences where the amount and complexity of new health information can be overwhelming. Library professionals who specialize in the health sciences need to have a deep understanding of the unique needs of health sciences to support faculty research, enrich student learning and develop and manage comprehensive health collections. These library professionals rely on a variety of professional development resources to develop their skills in the field of health science librarianship. This research project seeks to learn the preferred methods of professional development by health science library professionals. A survey was created using Qualtrics and seven instruction modalities were identified and included on the survey. The survey was sent out through various listservs used by health science librarians and the researcher is awaiting results. This poster documents the process of a novice researcher as she embarks on her first major research project with the guidance of the Medical Library Association Research Training Institute (MLARTI). The researcher will discuss the rapidly changing landscape of health information, her process in developing the survey tool, and how the current COVID-19 Pandemic, which forced her and other researchers to work remotely and change her instruction and services to an online format, affected her research process.

Research Data Services and Health Sciences Graduate Students: Preparation, Services, and Populations Served

Stacy Winchester - Research Data Librarian, University of South Carolina, Columbia, South Carolina

Jenessa Marie McElfresh AHIP - Health Sciences Librarian, Clemson University, Clemson, South Carolina

Megan Sheffield - Data Services Librarian, Clemson University, Easley, South Carolina

This project captures a snapshot of the work librarians providing Research Data Services (RDS) to health sciences graduate students are doing. Information about career preparation paths, work environments, services offered, and specific populations being served are of interest to members of the data librarian community. This research project utilized an anonymous survey of data librarians about a number of components of their work and areas of expertise.

Systematic, Interactive Teamwork in the Development of a Delphi Study: Advancing Knowledge through an Underutilized Methodology in Library and Information Science

Stacy F. Posillico MLS, JD - Senior Librarian, Northwell Health Libraries, Hempstead, NY

Background: Evidence-based research is essential to improving the state of the Library and Information Science profession. Librarians who develop brilliant ideas for research may lack the skills necessary to conduct a study on the topic. Without a meaningful understanding of available research methodologies and study design, Librarians may create projects that are inadequate for the study of complex processes and policies.

Description: Acceptance as an MLA Research Training Institute (RTI) Fellow and participation in the five-day RTI intensive workshop to develop research skills led to my discovery of an underutilized study methodology in Library and Information Science: The Delphi Method. This method, a type of mixed methods research, is often used in the Health Sciences to create guidelines and models for patient care and service delivery. Teaching and mentorship by the RTI Faculty led to a deeper understanding of the Delphi methodology and the determination that the Delphi Method was the most applicable means for studying a complicated research question. Furthermore, this type of research cannot be performed without a talented team and a commitment to allocate time devoted to scholarly research activities. The RTI Fellowship led to the recognition of opportunities for assembling the right team and finding the time to do the work, and in this case, led to the development of a research protocol paper to evaluate an important issue in hospital library leadership and management.

Conclusion: Developing research competencies as an MLA RTI Fellow has had a positive impact on my ability to conduct research, and our team has successfully developed a research protocol paper that will be presented at the 2021 MLA Annual Conference. The discovery of underutilized research methodologies during the RTI training, together with guidance as to how to establish a research team and dedicate time to scholarly activity, leads to a measurable increase in research confidence and capability.

Teaching the teachers: comparing MLIS curricula to entry-level job descriptions

Caitlin Meyer MLIS - Research and Education Librarian, Cushing/Whitney Medical Library at Yale, New Haven, CT

Note: This poster presentation is about work in progress for the presenter's MLA RTI project and does not have final results.

Objectives: The presenter entered the RTI program curious about the relationship between MLIS curricula and required skills in entry level jobs. Throughput the RTI program, this was

narrowed down to two questions: Are MLIS programs adequately preparing graduates for the academic librarian job market, particularly in regards to skills and knowledge around teaching? Are MLIS programs’ curricula reflective of skills and experience expected in practice?

Methods: The presenter conducted a multifaceted and interdisciplinary literature review to find appropriate theoretical frameworks and design the project. The course catalogs for all ALA accredited MLIS programs were collected and analyzed using structured content analysis. Due to the pandemic disrupting the frequency of job postings, this part of the project was delayed and is being redesigned. RESULTS: The presenter will share preliminary data from evaluating the course catalogs of all currently ALA accredited MLIS programs.

Conclusions: TBD pending completion of the second part of the data collection and analysis.

The Publication Fate of Abstracts Presented at the 2012 and 2014 Medical Library Association Conferences

Rachel J. Hinrichs AHIP - Health Sciences Librarian, IUPUI University Library, Indianapolis, Indiana

Objective: We sought to determine how many abstracts presented at the 2012 and 2014 Medical Library Association (MLA) annual conferences were later published as full-text journal articles and which features of the abstract and author influence the likelihood of future publication. To do so, we replicated a previous study on MLA conference abstracts presented in 2002 and 2003. The secondary objective was to compare the publication rates between the prior and current study.

Methods: Presentations and posters delivered at the 2012 and 2014 MLA meetings were coded to identify factors associated with publication. Post-conference publication of abstracts as journal articles was determined using a literature search and survey sent to first authors. Chi-squared tests were used to assess differences in the publication rate, and logistic regression was used to assess the influence of abstract factors on publication.

Results: The combined publication rate for the 2012 and 2014 meetings was 21.8% (137/628 abstracts), which is a statistically significant decrease compared to the previously reported rate for 2002 and 2003 (27.6%, 122/442 abstracts). The odds that an abstract would later be published as a journal article increased if the abstract was multi-institutional or if it was research, specifically surveys or mixed methods research.

Conclusions: The lower publication rate of MLA conference abstracts may be due to an increased number of program or non-research abstracts that were accepted or a more competitive peer review process for journals. MLA could increase the publication rate by encouraging and enabling multi-institutional research projects among its members.

Transmutations in a Research Curriculum: Seeking to Encourage Enthusiasm for the Research Process in a Psychiatry Residency

Ellen M. Justice AHIP - Clinical & Research Librarian, UNC Health Sciences at MAHEC (Mountain Area Health Education Center), Asheville, North Carolina

Kimberly Leach

Stephen Buie - MAHEC

Kathy Foley - Faculty, MAHEC, Tryon, North Carolina

Objective: Assess the Psychiatry Residency Research Curriculum’s effectiveness in encouraging community-based residents’ enthusiasm for and developing confidence in research knowledge and skills.

Methods: Initial and post- surveys created in RedCap®. Initial survey had response rate of 88%, n=15; post-survey a response rate of 53%, n=9. Surveys were analyzed and interpreted to provide insights into the strengths and gaps in the curriculum.

Results: Initial survey indicated sixty percent thought it was important to have protected research time. Over half indicated receiving guidance from a mentor was important. The post-survey suggested a trend toward both increased unenthusiasm and increased enthusiasm for research. An open-ended question asked what could increase their enthusiasm and yielded: want to identify project that is meaningful contribution; need a dedicated mentor; and nothing could increase enthusiasm. The post-survey indicated that over half felt “somewhat confident” with their research skills while twenty-two percent said they were “somewhat unconfident.” A limitation to being able to provide direct comparisons of the surveys is that of a small cohort (n=17), fewer responded to the post-survey (n=9) compared to the initial survey (n=15).

Throughout the years, faculty have been responsive to the needs of the residents; therefore, several transmutations were made to the curriculum since 2018. Included are decrease in number of sessions from nineteen to thirteen. During 2020-21, the faculty instituted that instead of launching a research project, residents write up a psychiatric case report for which residents also provide blinded peer review. This provides opportunity to both learn to write a case report and practice peer review. Further to increase accountability in discussions, pre-assignments are now required. This has also been helpful in increasing engagement since the curriculum had to move online because of the COVID-19 pandemic. The course is now pass/fail.

Conclusions: This evaluation has allowed the research faculty to better understand how to meet both accreditation requirements for the residency as well as the needs of residents who have specifically chosen a community-based residency instead of a residency based at an academic research institution. Although research is not everyone’s “cup of tea,” we do think the curriculum provided more experience with critical analysis and encouraged informed consumption of studies. It introduced the peer review process which is important in publication

of robust literature. Cultivating enthusiasm for research is elusive; however, we do believe that the curriculum instilled an appreciation for the rigor that is needed in meaningful research.

Ultimately Better Healthcare Through Interactions of an Interprofessional Journal Club

Helen-Ann B. Epstein MLS, MS, AHIP, FMLA - Informationist, Virtua Health Sciences Library/Clinical Learning Team, Mt Laurel, NJ

See poster for further information.
